# Photoinduced EDA-complex-mediated late-stage C–H iodination of arenes using aryldibenzothiophenium salts under air

**DOI:** 10.1039/d6ra06512k

**Published:** 2026-10-05

**Authors:** Yan Ni, Pingyang Sun, Miaoting Wang, Wenwen Sun, Ping Zhang

**Affiliations:** a Key Laboratory of Green and Precise Synthetic Chemistry and Applications, Ministry of Education, School of Chemistry and Chemical Engineering, Huaibei Normal University Huaibei Anhui 235000 P. R. China zping90950@126.com

## Abstract

We report a visible-light-driven, metal-free iodination of arenes using aryldibenzothiophenium (Ar-DBT) salts and potassium iodide. This transformation operates efficiently under air with KI as the optimized iodide source, forming an electron donor–acceptor (EDA) complex that generates aryl radicals upon visible-light irradiation. This protocol exhibits broad functional group tolerance across electronically diverse arenes, heterocycles, and sterically congested substrates. Notably, the synthetic utility of this protocol is demonstrated through the late-stage diversification of a wide array of structurally complex bioactive, agrochemical molecules, and functional molecules, as well as the efficient synthesis of dihalogenated xantphos derivatives.

## Abstract 2

A visible-light-induced, metal-free protocol for arene C–H iodination using Ar-DBT salts and KI, enabling late-stage functionalization of complex bioactive compounds and synthesis of xantphos-based ligand precursor under air.

## Graphical abstract

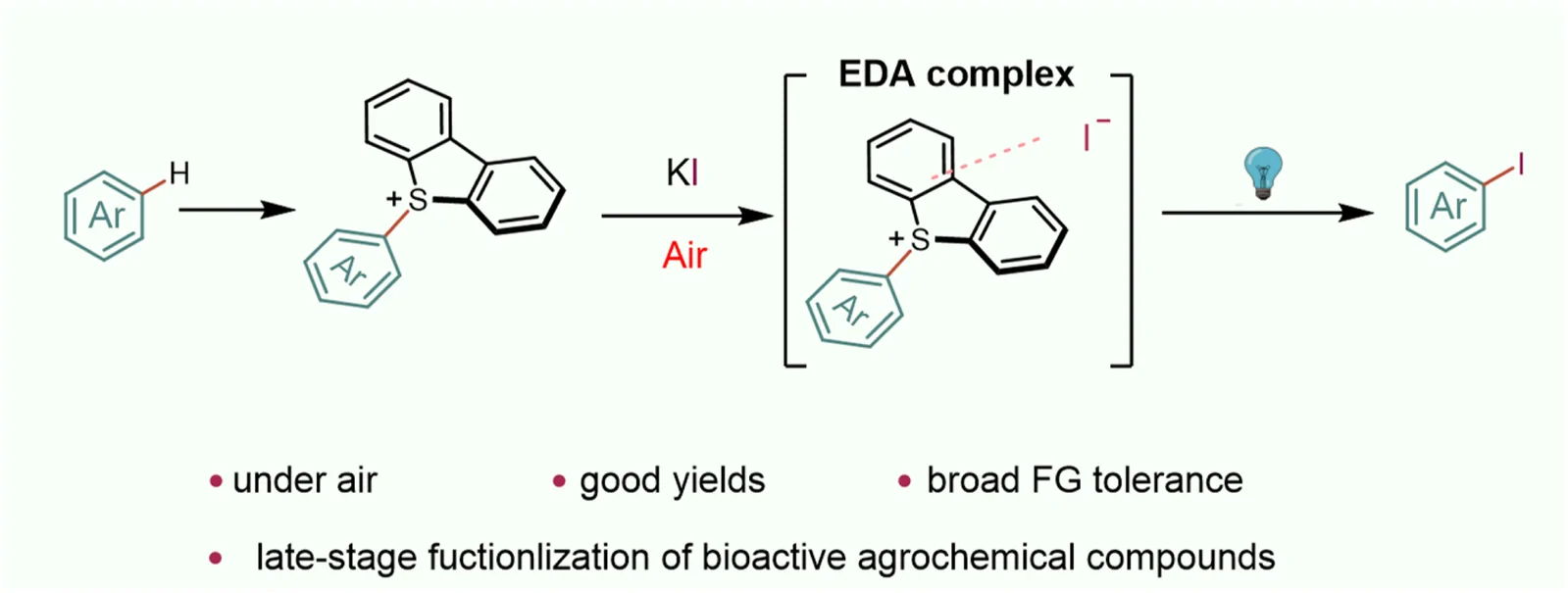

Aryl iodides are pivotal building blocks in synthetic organic chemistry, extensively employed to construct carbon–carbon^1^ (C–C) and carbon–heteroatom^2^ (*e.g.*, C–O, C–N, *etc.*) bonds *via* transition metal-catalyzed cross-coupling reaction (Scheme 1A). The formation of C–C bonds is well-established, as exemplified by the Nobel Prize-honored Heck, Negishi, and Suzuki reactions.^1*a*–*c*^ In parallel, the development of C–X bond coupling has also seen transformative advances exemplified by two landmark protocols: on the one hand, the palladium-catalyzed Buchwald–Hartwig amination,^2*a*–*d*^ which has progressed rapidly through the continuous development of phosphine ligands; on the other hand, copper-catalyzed Ma's Ullmann-type reactions,^2*e*,*f*^ where systematic ligand optimization—from amino acids to oxalamides—has enabled progress under milder conditions, and facilitated industrial-scale pharmaceutical production. While, aryl iodides are commonly synthesized *via* the direct iodination of arene C–H bonds,^3^ typically employing iodine (I_2_) or *N*-iodosuccinimide (NIS) serving as the iodinating agents in the presence of strong oxidants or strong acids (Scheme 1B).^4,5^ These methods inherently suffer from limitations, including unpredictable site-selectivity, poor functional group tolerance, and the generation of stoichiometric waste. To address these issues, significant advances have been made in recent years. For example, metal-catalyzed C–H iodination using directing groups has enabled highly regioselective transformations,^6^ while photochemical approaches employing iodine radicals under mild conditions have emerged as complementary strategies.^7^ Electrochemical iodination has also been developed as a sustainable alternative.^8^ Alternatively, Jiao and co-workers developed DMSO-based oxidative halogenation and Lewis base catalysis,^9^ while Tang and co-workers established an alloxan/ascorbic acid biomimetic system for aerobic oxidative halogenation.^10^ Despite these promising developments, many of these methods still require transition-metal catalysts, external oxidants, or specialized apparatus, highlighting the need for a mild, metal-free, and operationally simple iodination protocol.

**Scheme 1:**
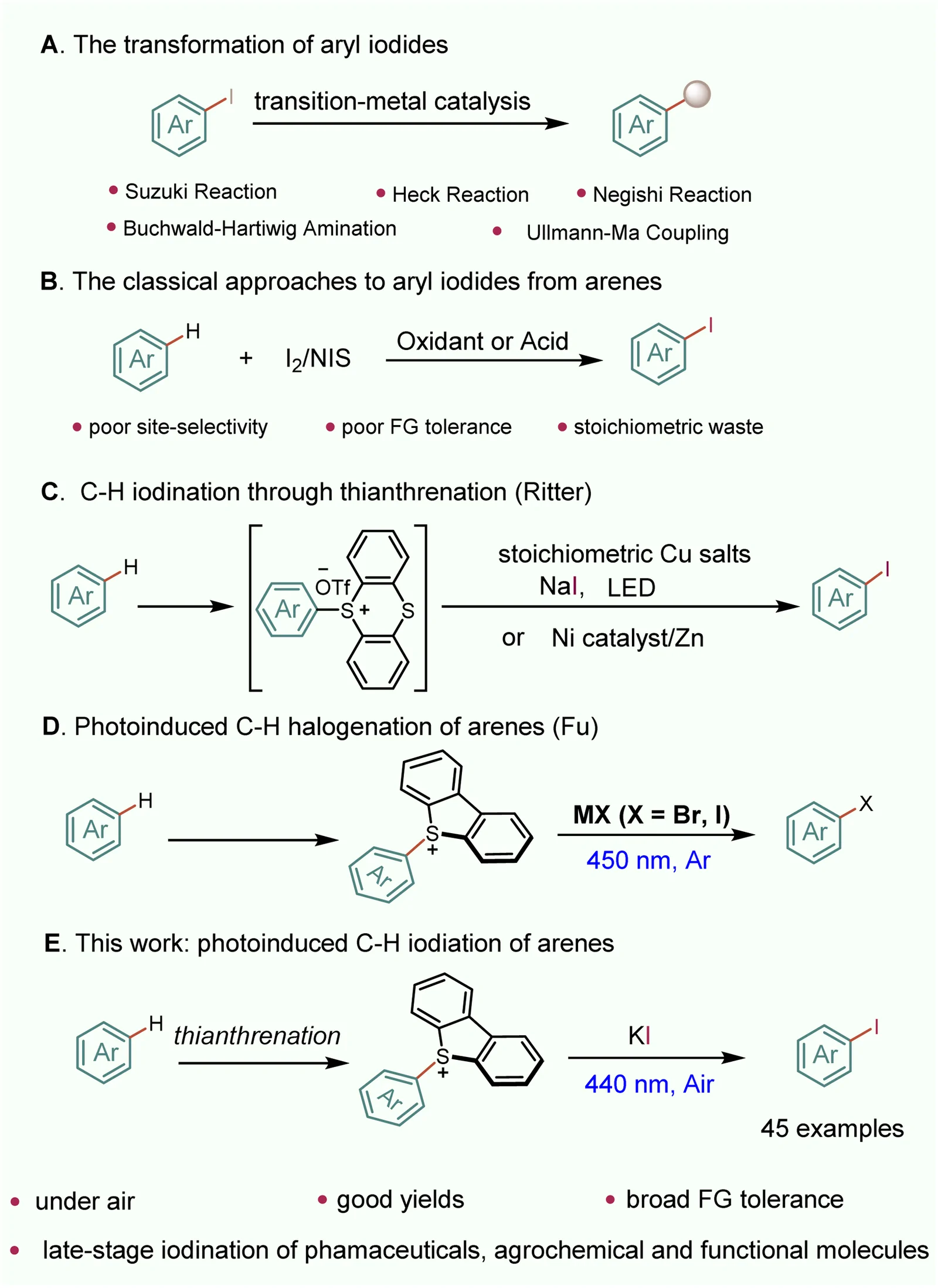
(A) The application of aryl iodides. (B) The classical approaches to aryl iodides from arenes. (C) C–H iodination through thianthrenation. (D) Photoinduced halogen anion-mediated C–H halogenation of arenes. (E) This work: photoinduced late-stage C–H iodiation of arenes.

In recent years, electron donor–acceptor (EDA) complexes have emerged as a powerful platform for photochemical transformations without the need for external photocatalysts.^11^ Upon visible-light irradiation, an EDA complex formed between an electron-rich donor and an electron-deficient acceptor undergoes single-electron transfer (SET) to generate radical species under mild conditions.^12^ This strategy has been successfully applied to diverse transformations, including C–C and C–heteroatom bond formations,^11*a*,*f*,11*i*,*j*^ radical additions,^11*a*–*e*^ and arylations.^11*a*^ The inherent advantages of EDA-mediated photoreactions—namely, catalyst-free conditions, operational simplicity, and compatibility with air and moisture—make them highly attractive for sustainable synthetic chemistry.

Over the past decade, organosulfonium salts have gained increasing attention as versatile intermediates for arene C–H functionalization, offering unique advantages in site-selectivity and reaction mildness.^13^ Among these, thianthrenium (Ar-TT^+^) salts stand out for their dual reactivity as both electrophiles and radical precursors. A key advance came from Ritter and co-workers, who established a site-selective arene C–H thianthrenation protocol that enables the preparation of Ar-TT^+^ salts from complex arenes with excellent regiocontrol.^7*c*,13*a*,*b*^ These salts have since enabled a range of transformations, including arylation, alkylation, and halogenation, *via* transition-metal catalysis or photoredox processes (Scheme 1C). In parallel, the Wang group has further enriched the chemistry of sulfonium salts through copper-mediated thianthrenation and phenoxathiination of arylborons, as well as *para*-selective borylation of monosubstituted arenes using a transient mediator.^14^ More recently, Procter and co-workers demonstrated that aryldibenzothiophenium (Ar-DBT^+^) salts can serve as effective acceptors in EDA complexes for visible-light-driven C–H functionalization of arenes *via* radical pathways.^11*a*,*c*^ Given the synthetic potential of Ar-DBT^+^ salts and our ongoing interest in sulfonium-based photochemistry,^11*a*–*c*,13*g*,*h*,15^ we envisioned that these readily accessible salts could serve as aryl radical precursors for C–H iodination under visible-light irradiation.

Of note, during the course of our research, Fu and co-workers reported an elegant EDA complex-mediated halogenation of arenes using Ar-DBT salts (Scheme 1D).^16^ While their work established the feasibility of this transformation, our protocol offers complementary advantages, including enhanced functional group compatibility, operation under air, and applicability to a broader range of structurally diverse substrates (Scheme 1E).

Initially, the reaction was conducted employing aryl dibenzothiophenium salt (Ar-DBT, 1) prepared from corresponding arene as the model substrate and KI as the iodine source under irradiation by 440 nm LEDs, as shown in Table S1 (see Section 2.1 in the SI for details). Initial investigation using THF as the solvent afforded the iodinated product 2 in 57% yield, accompanied by the protonated by-product 2′ in 35% yield (entry 1, Table S1). To suppress the formation of 2′, a series of solvent screening was conducted. Among the solvents screened, such as acetone, DMF, DMA, DMSO, DCM, 1,4-dioxane, EA, H_2_O, and CH_3_CN, CH_3_CN was found to be optimal, affording the target product 2 in 80% yield and suppressing the formation of 2′ to 12% (entries 2–11, Table S1). Notably, replacement of KI with lithium iodide (LiI), sodium iodide (NaI) or tetrabutylammonium iodide (TBAI) led to a diminished reaction yield compared to the results achieved with KI (entries 1–4, Table 1). This observation indicates that the countercation plays an active role. It may be attributed to the moderate Lewis acidity and optimal ionic radius of K^+^, which facilitate the formation of a compact and ordered EDA complex with the Ar-DBT^+^ cation and I^−^, promoting efficient single-electron transfer upon light irradiation.^17^

**Table 1:** Optimization of the reaction conditions^a^^,^^b^

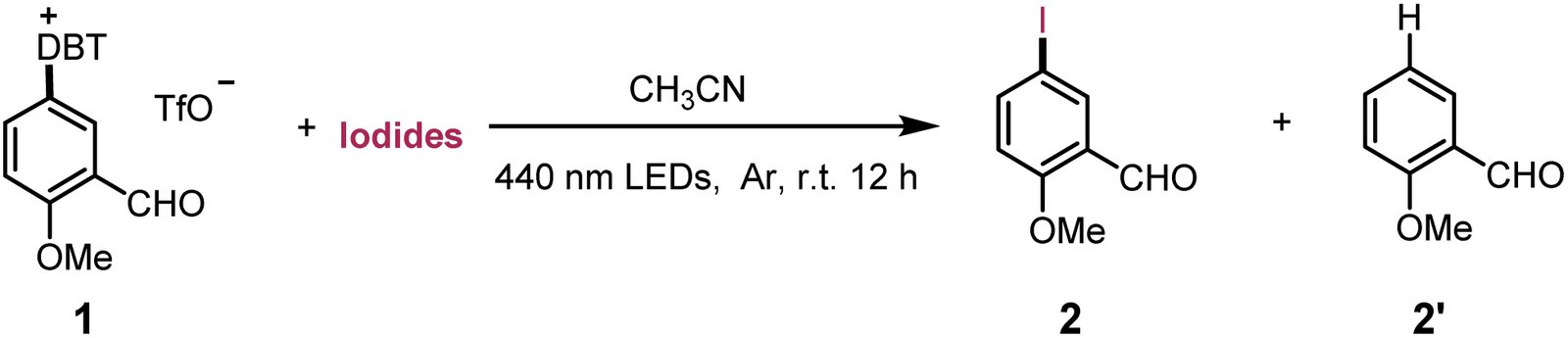				
Entry	Iodides	Iodides (*x* equiv.)	2 (%)^b^	2′ (%)^b^
1	Lil	2.0	70	17
2	Nal	2.0	74	16
3	KI	2.0	80	11
4	*n*-Bu_4_NI	2.0	72	16
5	KI	1.0	74	13
6	KI	1.5	86	7
7	KI	2.5	76	10
8	KI	3.0	75	10
9^c^	KI	1.5	74	6
10^d^	KI	1.5	84	9
11^e^	KI	1.5	84	8
12^f^	KI	1.5	66	14

a Reaction conditions: aryl dibenzothiophenium salt 1 (0.2 mmol, 1.0 equiv.), KI (2.0 equiv.) in solvent (2 mL) under argon atmosphere and LEDs irradiation for 12 h at room temperature.

b The yield was determined by ^1^H NMR analysis of crude product using 1,3,5-trimethoxybenzene as an internal standard.

c Reaction time for 9 h.

d Reaction time for 15 h.

e Under air.

f O_2_.

Subsequently, the loading of KI was optimized. Screening from 1.0 to 3.0 equivalents revealed that 1.5 equivalents delivered the optimal outcome, elevating the yield of the desired product 2 to 86% with a reduction of 2′ to 7% (entries 3, 5–8, Table 1). The reaction conditions were further optimized (entries 6, 9–10, Table 1). Time-course studies showed that the yield reached a maximum of 86% at 12 h. Examination of the irradiation wavelength identified 440 nm as optimal (entries 2–3, Table S4). To our delight, the reaction proceeded smoothly under an air atmosphere (entries 11 and 12, Table 1), affording the desired product 2 in 84% yield comparable to that obtained under an inert argon atmosphere. In view of the similar efficiency and the significantly enhanced operational simplicity, air was chosen as the optimal atmosphere for this transformation. A control experiment conducted in the dark confirmed that light irradiation is essential for the reaction (entry 8, Table S4). Accordingly, the optimized reaction conditions were established as follows: 1 (0.2 mmol), KI (1.5 equiv.) in CH_3_CN (2.0 mL) irradiated by 440 nm LEDs under air for 12 h.

Building upon the optimized conditions, we next investigated the generality of the transformation using a series of substituted Ar-DBT salts prepared *in situ* from the corresponding arenes (Table 2). The catalytic system displayed broad functional-group tolerance, smoothly accommodating both electron-donating groups (*e.g.*, methyl, *tert*-butyl, isopropyl, alkyl, alkoxy, and amide) and electron-withdrawing substituents (*e.g.*, fluoro, chloro, bromo, cyano, ester, nitro, and ketone). Initially, the scope of *para*-substituted substrates was examined. A range of DBT salts bearing diverse functional groups, including *tert*-butyl (5), isopropyl (4), methoxy (8), alkoxy ether/ester motifs (6 and 7), propargyl ether (9), amide (10, 11), hydrogen (3), and halogens (F, Cl, Br; 12–14), were.

**Table 2:** Scope of Ar-DBT salts^a^^b^

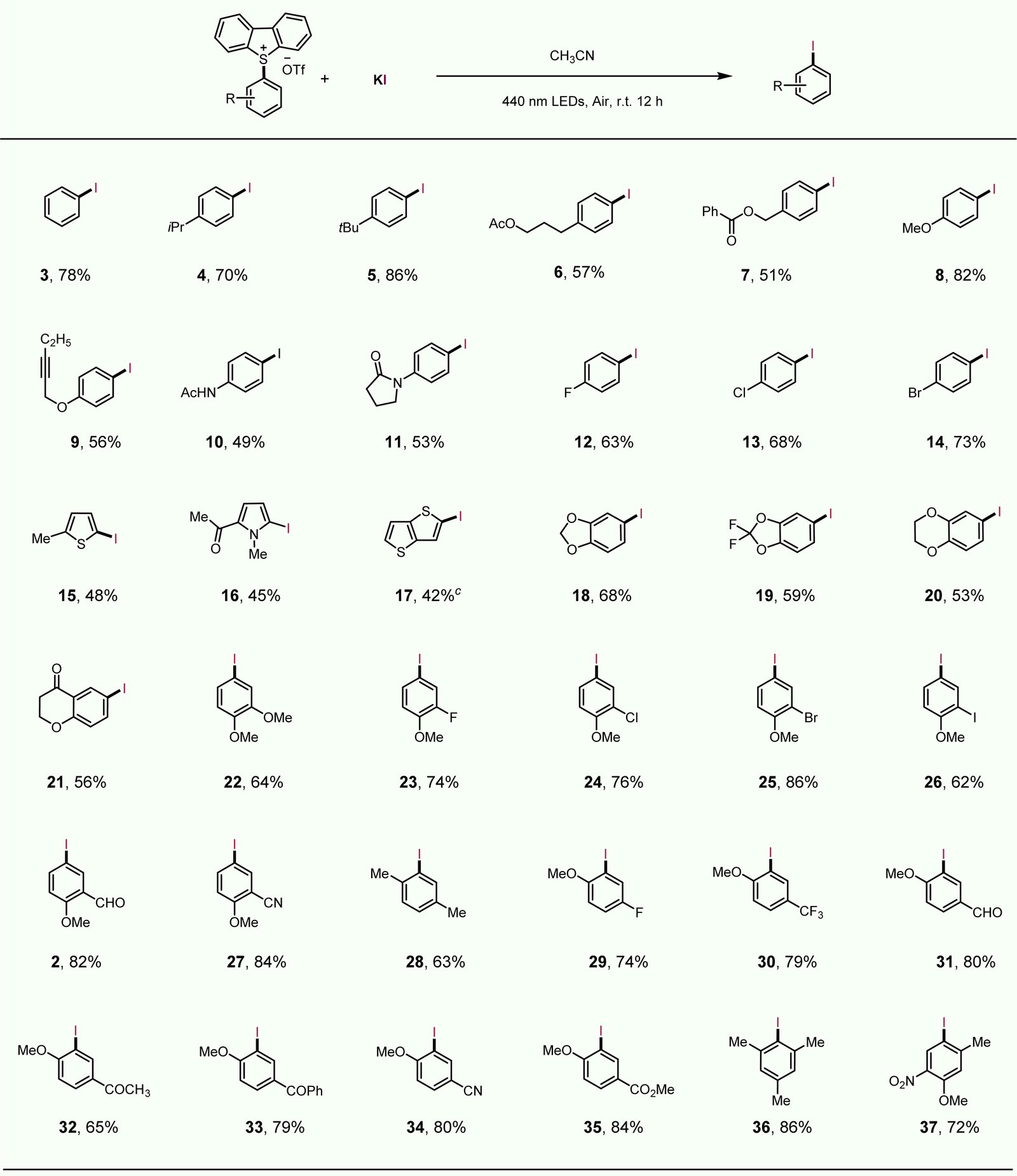

a Reaction conditions: aryl dibenzothiophenium salt (0.2 mmol), KI (1.5 equiv.) in CH_3_CN (2 mL) under air atmosphere and LEDs irradiation for 12 h at room temperature.

b Isolated yields.

c 18 h.

Smoothly converted to the corresponding aryl iodides in moderate to good yields (49–86%). The method also proved applicable to heterocyclic systems, such as thiophene (15, 17) and pyrrole derivatives (16), albeit in somewhat lower yields. Notably, the protocol was successfully extended to disubstituted arenes, encompassing both *ortho*- and *para*-substituted arenes (2, 18–35). Aryl DBT salts derived from 1,3-benzodioxle derivatives (18, 19) and 1,4-benzodioxan (20) furnished the corresponding products in 68%, 59%, and 53% yields, respectively. Likewise, an isochromanone-derived aryl DBT salt could also be transformed into compound 21 with a yield of 56%. To further evaluate functional-group compatibility, a variety of substituted anisole-based aryl DBT salts were explored. Substrates bearing *ortho*-methoxy along with additional *ortho*-substituents (OMe, F, Cl, Br, CHO, and CN) proved compatible, yielding the corresponding disubstituted aryl iodides in 62–86% yields (2, 22–27). Moreover, aryl DBT salts featuring *para-*electron-withdrawing groups (F, CF_3_, CHO, COCH_3_, COPh, CN, and CO_2_Me) relative to methoxy delivered the iodinated products in excellent yields (28–35). Remarkably, the system also demonstrated high reactivity toward sterically congested trisubstituted arenes, affording the desired products (36, 37) in excellent yields without compromising efficiency, underscoring the robustness and versatility of the method.

This transformation proved to be a versatile platform for late-stage diversification of bioactive molecules as well as organic optoelectronic materials (Scheme 2). Thus, a diverse array of aryl iodides derivatives was efficiently synthesized in moderate to good yields (Scheme 2A), encompassing anti-inflammatory agents (xanthone 38, diclofenac 42, indomethacin 46), hypolipidemic drugs (clofibrate 39, fenofibrate 44, gemfibrozil 45), thiazepin-derived antipsychotic agents 43, and dibenzothiophene-based optoelectronic π-conjugated precursor 47.^18^ Notably, the method also enabled access to iodinated derivatives of phytohormone 40, highlighting its potential in agrochemical research. Furthermore, its applicability extended to structurally distinct scaffolds, as demonstrated by the synthesis of triptycene-based iodide 41, which possesses a rigid three-dimensional architecture with promising functional properties. Beyond pharmaceutical modification, the protocol also served as a mild chemo-selective entry to functional molecular building blocks with orthogonal halogenation sites. The preparation of dihalogenated xantphos precursors showcases the access to valuable bromo-ido scaffolds inaccessible *via* conventional NBS-based bromination, underscoring the broad applicability of this iodination strategy for diverse molecular synthesis (Scheme 2B).

**Scheme 2:**
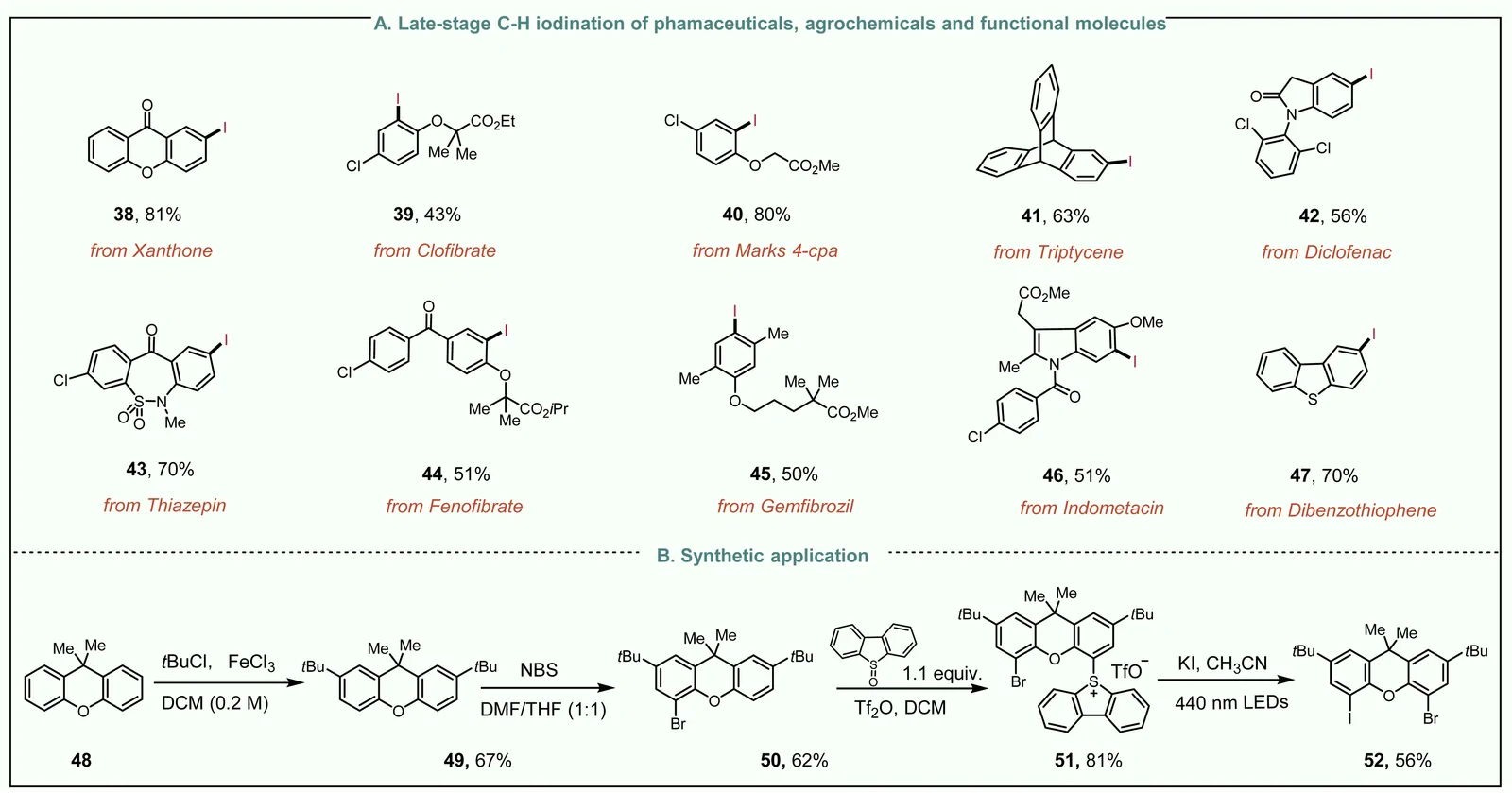
(A) Late-stage C–H iodination of phamaceuticals, agrochemical and functional molecules. (B) Synthetic application.

On a 5 mmol scale, treating Ar-DBT salt 1 with KI successfully afforded 2 in 81% yield under standard conditions. This result demonstrated the practical utility of this synthetic method (see Section 2.3 in the SI). To elucidate the reaction pathway, a series of preliminary mechanistic investigations was carried out. Initially, the reaction of Ar-DBT salt 1 and KI was completely suppressed in the absence of KI or light, and no iodinated product was observed, and the Ar-DBT salt remained largely intact, indicating no detectable background homolysis under 440 nm irradiation (Scheme 3a). Further insight into the mechanism was gained through radical trapping experiments (Scheme 3b). 3 equiv. of 2,2,6,6-tetramethyl-1-piperidinyloxyl (TEMPO), 5,5-dimethyl-1-pyrroline *N*-oxide (DMPO) or 1,1-diphenylethylene as radical scavengers were added to the reaction mixture under standard conditions. The reaction was severely inhibited by radical inhibitors in presence of TEMPO, demonstrating that may proceed *via* a radical process.^19^ Additionally, the adducts 52–54 were successfully detected by high-resolution mass spectrometry (HRMS). The light on/off experiments indicated that continuous irradiation is essential for the reaction to proceed (Scheme 3c), and the quantum yield of 0.28 (*Φ* < 1) is inconsistent with a radical chain propagation pathway (see Section 2.5.7 in the SI). Furthermore, UV/vis absorption spectra of the reaction components and their mixture revealed a new charge-transfer band, suggesting the formation of an EDA complex between Ar-DBT salt 1 and KI (Scheme 3d).^20^ This assignment was further supported by Job's plot analysis, which established a 1 : 1 binding stoichiometry between the Ar-DBT cation and I^−^ (Scheme 3e).

**Scheme 3:**
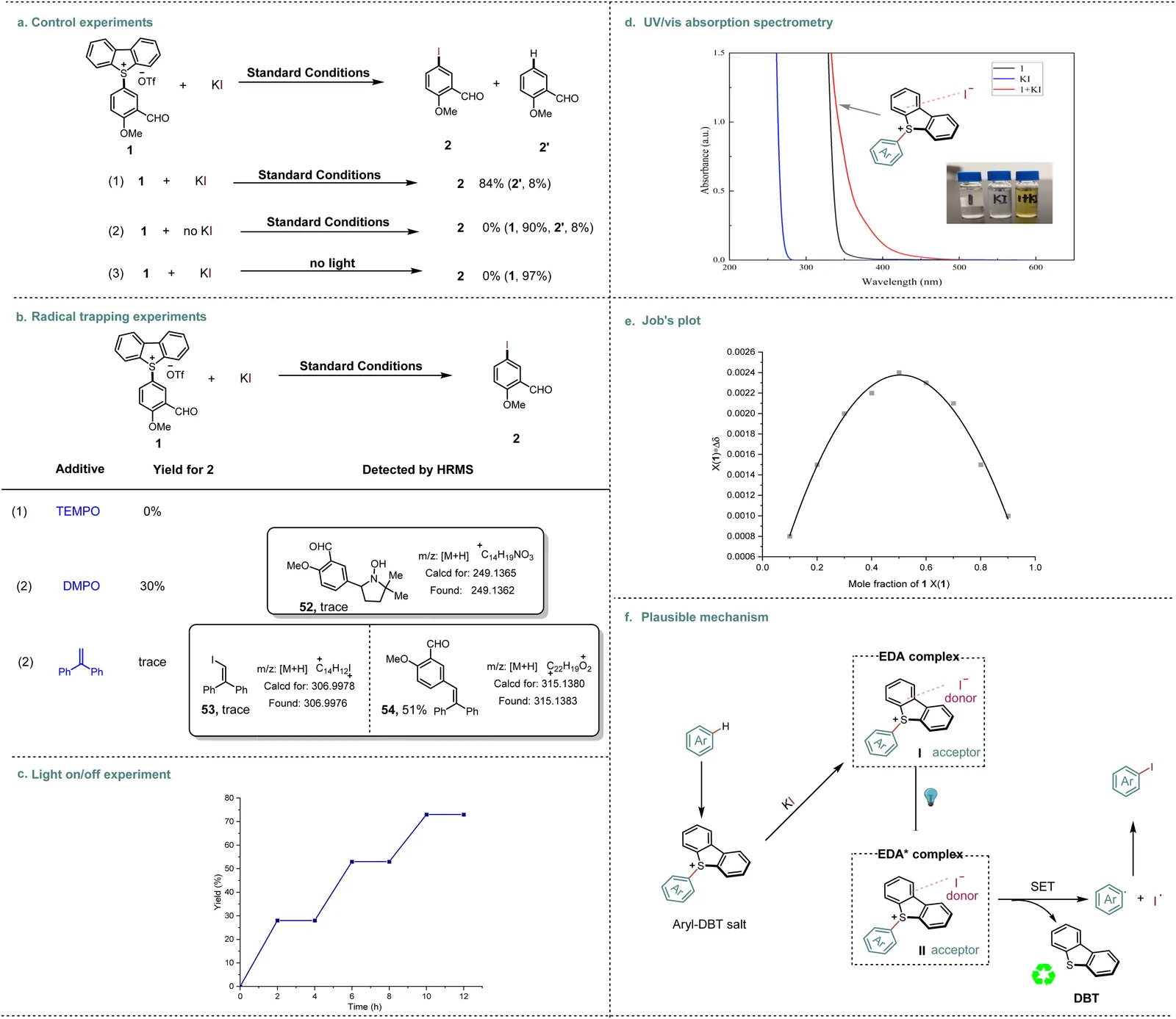
(a) Control experiments. (b) Radical trapping experiments. (c) Light on/off experiment. (d) UV/vis absorption spectrometry. (e) Job's plot. (f) Plausible mechanism.

Based on the experimental observations and in line with previous reports,^11*a*–*c*,21^ a plausible reaction mechanism is proposed (Scheme 3f). Initially, the Ar-DBT salt forms an electron donor–acceptor (EDA) complex (I) with KI. Upon visible-light irradiation, this complex is promoted to its excited state (EDA* complex II), then undergoes a single-electron transfer (SET) process, generating aryl radical and iodide radical, along with the by-product DBT—which can be readily recovered and reused as a precursor for the synthesis of arylsulfonium salts. Subsequently radical–radical coupling furnishes functionalized aryl iodides.

In summary, we have developed a practical, visible-light-driven, metal-free arene C–H iodination using Ar-DBT salts and KI. The reaction operates efficiently under air with KI as the optimal iodide source, providing a broad range of aryl iodides in good to excellent yields. The mild conditions enable late-stage iodination of complex bioactive molecules and gram-scale synthesis. This protocol offers a complementary, operationally simpler alternative to existing EDA-mediated iodination methods.

## Conflicts of interest

The authors declare no competing financial interest.

## Supplementary Material

RA-OLF-D6RA06512K-s001

## Data Availability

The data supporting this article have been included as part of the supplementary information (SI), and experimental data have been provided as the SI. Supplementary information: experimental details and characterization data of all new compounds. See DOI: https://doi.org/10.1039/d6ra06512k.
