# Supplementary material for: Photoinduced EDA-complex-mediated late-stage C–H iodination of arenes using aryldibenzothiophenium salts under air
Source: RSC Adv. 2026 Oct 5;16(46):59941–8. doi: 10.1039/d6ra06512k (PMC13635897; doi:10.1039/d6ra06512k)
Supplement: Supplementary file 1 [file RA-016-D6RA06512K-s001.pdf]

Supplementary Information (SI) for RSC Advances.
This journal is © The Royal Society of Chemistry

Supporting Information

Photoinduced EDA-Complex-Mediated Late-stage C-H Iodination of Arenes using Aryldibenzothiophenium Salts Under Air

Yan Ni[a], Pingyang Sun[a], Miaoting Wang[a], Wenwen Sun[a], Ping Zhang[a, *]

[a]Key Laboratory of Green and Precise Synthetic Chemistry and Applications, Ministry of Education, School of Chemistry and Chemical Engineering, Huaibei Normal University, Huaibei, Anhui 235000, P. R. China

Email: zping90950@126.com

Table of content

1. General Information 3

2. Experimental Section 3

2.1. Optimization Studies 3
2.2. Material and Product Preparation Procedure 5
2.2.1 Preparation for Aryl dibenzothiophenium salts[1] 5
2.2.2 General Procedure for Aryl iodides 6
2.2.3 Reaction Setup 6
2.3. Gram-Scale Synthesis for Products 2 6
2.4. Synthetic Application 7
2.5. Mechanism Studies 9
2.5.1 Control Experiments 9
2.5.2 Radical- trapping with TEMPO 10
2.5.3 Radical- trapping with DMPO 10
2.5.4 Radical trapping with 1,1-Diphenylethylene 11
2.5.5 Light on-off experiment 11
2.5.6 UV-vis absorption spectra 12
2.5.7 Quantum yield measurement 12
2.5.8 Determination of the binding by Job's plot 13
2.6. Characterization Date of All Products 14
2.7. References 32
3. ^{1}H, ^{13}C and ^{19}F spectra of All Products 36

1. General Information

Nuclear magnetic resonance spectra were recorded on Bruker 600 MHz instruments internally referenced to TMS (0.0 ppm) or residue of $CDCl_3$ (7.26 ppm for ^{1}H, and 77.00 ppm for ^{13}C) signal. The following abbreviations were used to explain multiplicities: s = singlet, d = doublet, t = triplet, q = quartet, m = multiplet, br = broad. High-resolution mass spectra (HRMS) were conducted at Micromass Q-T of instrument (ESI). All reagents were purchased at the highest commercial quality and used without further purification unless otherwise stated. Solvents were distilled using standard techniques.

2. Experimental Section

2.1. Optimization Studies

Table S1. Screening of Solvents[a]

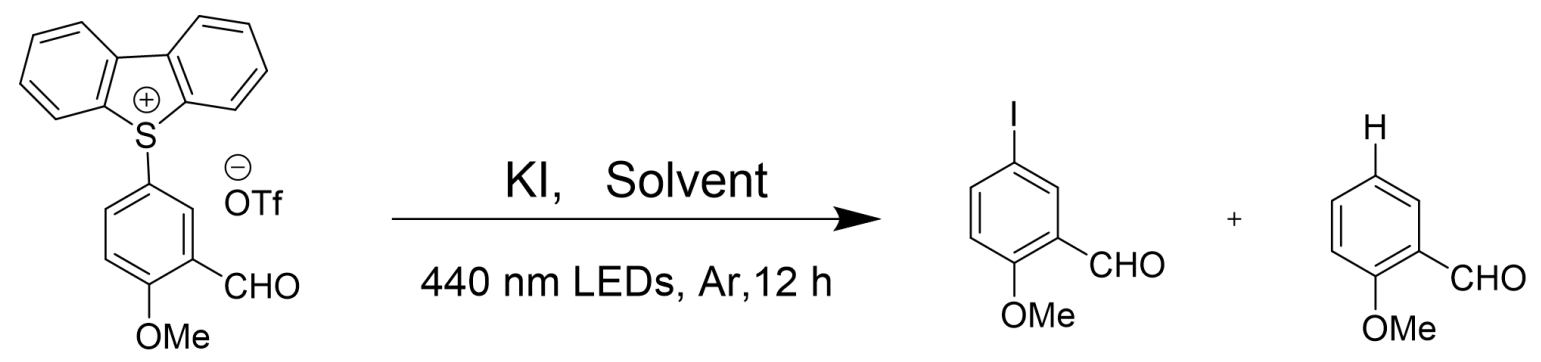

1 **2** **2'**

Entry	Solvent	**2** (%)[b]	**2'** (%)[b]
1	THF	56	35
2	DCM	75	16
3	DMSO	52	12
4	DMA	59	30
5	DMF	57	32
6	Acetone	73	20
7	1,4-dioxane	54	25
8	EA	74	18
9	H_2O	5	85
10	CH_3CN/H_2O	14	76
11	CH_3CN	80	11

[a] Reaction Conditions: Aryl dibenzothiophenium salt **1** (0.2 mmol, 1.0 equiv.), KI (2.0 equiv.) in solvent (2 mL) under argon atmosphere and LED irradiation for 12 h at room temperature. [b] The yield was determined by ^{1}H NMR analysis of crude product using 1,3,5-trimethoxybenzene as an internal standard.

Table S2. Screening of Iodides[a]

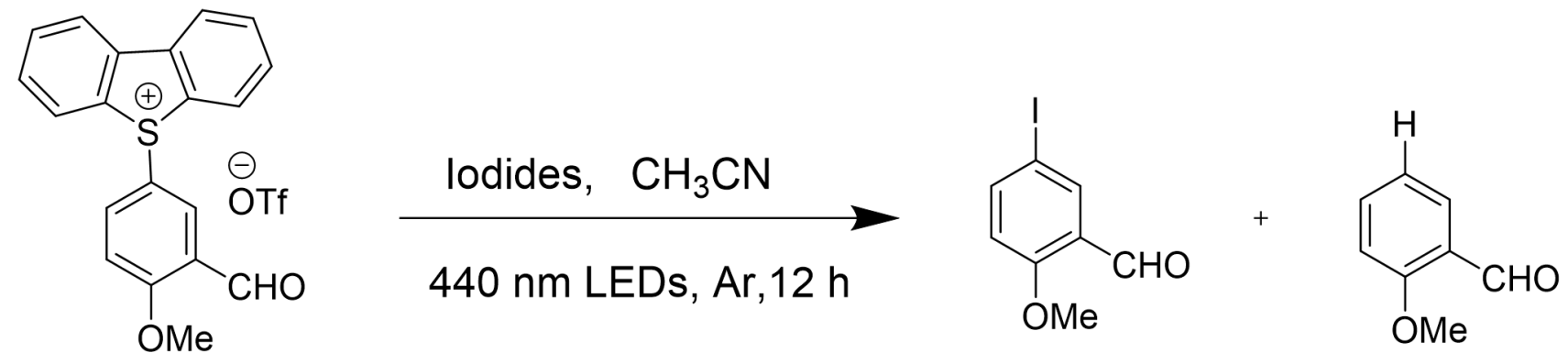

1 2 2'

Entry	Iodides	2 (%)[b]	2' (%)[b]
1	LiI	70	17
2	NaI	74	16
3	KI	80	11
4	TBAI	72	16

[a] Reaction Conditions: Aryl dibenzothiophenium salt **1** (0.2 mmol, 1.0 equiv.), Iodides (2.0 equiv.) in CH_3CN (2 mL) under argon atmosphere and LED irradiation for 12 h at room temperature. [b] The yield was determined by ^{1}H NMR analysis of crude product using 1,3,5-trimethoxybenzene as an internal standard.

Table S3. Screening of KI equivalents[a]

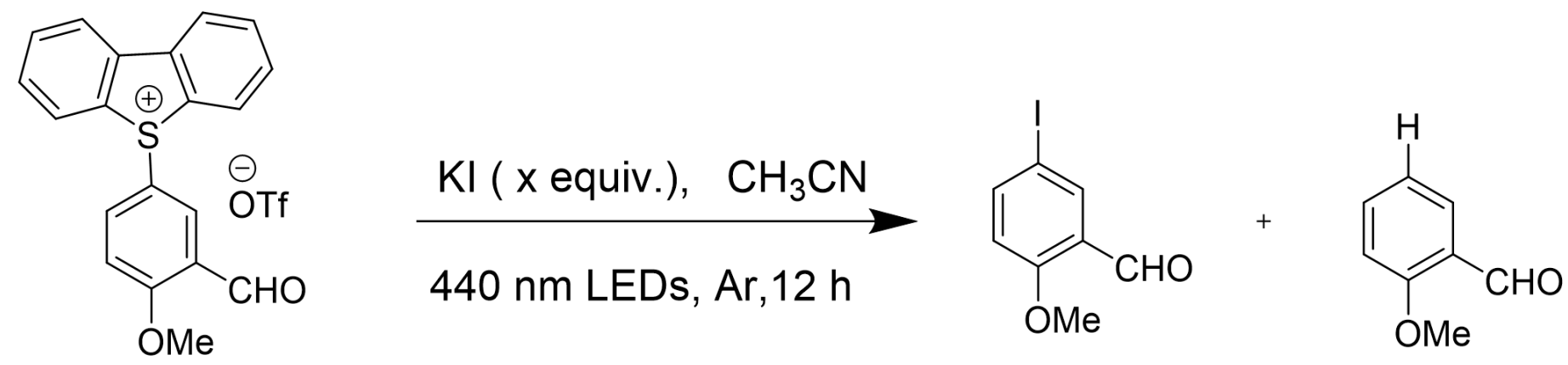

1 2 2'

Entry	KI (x equiv.)	2 (%)[b]	2' (%)[b]
1	1.0	74	13
2	1.5	86	7
3	2.0	80	11
4	2.5	76	10
5	3.0	75	10

[a] Reaction Conditions: Aryl dibenzothiophenium salt **1** (0.2 mmol, 1.0 equiv.), KI (x equiv.) in CH_3CN (2 mL) under argon atmosphere and LED irradiation for 12 h at room temperature. [b] The yield was determined by ^{1}H NMR analysis of crude product using 1,3,5-trimethoxybenzene as an internal standard.

Table S4. Control Experiments[a]

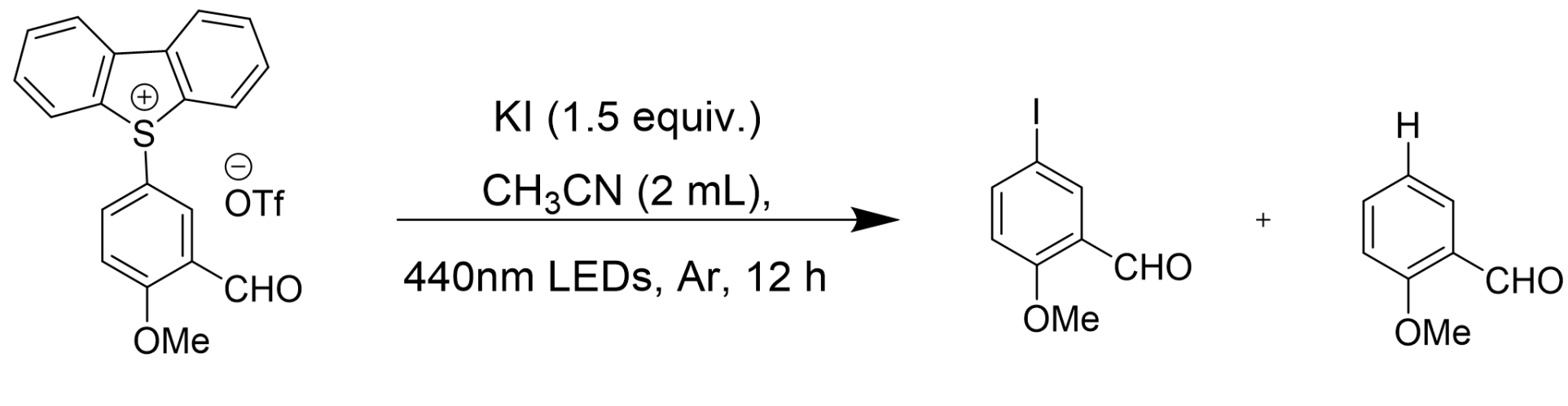

1 2 2'

Entry	Variation from conditions	**2** (%)[b]	**2'** (%)[b]
1	none	86	7
2	reaction time for 9 h	74	6
3	reaction time for 15 h	84	9
4	427 nm LEDs	80	11
5	456 nm LEDs	73	12
6	under air	84	8
7	O_2	66	14
8	No light	0	0

[a] Reaction Conditions: Aryl dibenzothiophenium salt **1** (0.2 mmol, 1.0 equiv.), KI (1.5 equiv.) in CH_3CN (2 mL) under argon atmosphere and LED irradiation for 12 h at room temperature. [b] The yield was determined by ^1H NMR analysis of crude product using 1,3,5-trimethoxybenzene as an internal standard.

2.2. Material and Product Preparation Procedure

2.2.1 Preparation for Aryl dibenzothiophenium salts[1]

To a solution of dibenzothiopheneoxide (1.1 equiv.) and arene (10.0 mmol, 1.0 equiv.) in dichloromethane (50 mL) in -78 °C was added trifluoromethanesulfonic anhydride (1.2 equiv.) dropwise, and after the addition was complete, the reaction was continued for an additional hour at this temperature. The mixture was concentrated and filtered with a short column washing with 5% CH_3OH/ dichloromethane. The filtrate was concentrated and used for the next step without purification.

2.2.2 General Procedure for Aryl iodides

The aryl DBT salt (0.2 mmol, 1.0 equiv.) and KI (0.3 mmol, 1.5 equiv.) were added to a solution in CH_3CN, then stirred under irradiation by LEDs at room temperature for 12 h. Upon completion, the reaction mixture was filtered through a pad of Celite. The filtrate was concentrated in vacuo to afford crude products, which were purified by flash column chromatography on silica gel to afford the pure products.

2.2.3 Reaction Setup

All photochemical reactions were carried out using a Kessil PR160L-440 nm LED lamp as the light source, with the distance between the lamp and the reaction vial fixed at 1.5 cm.

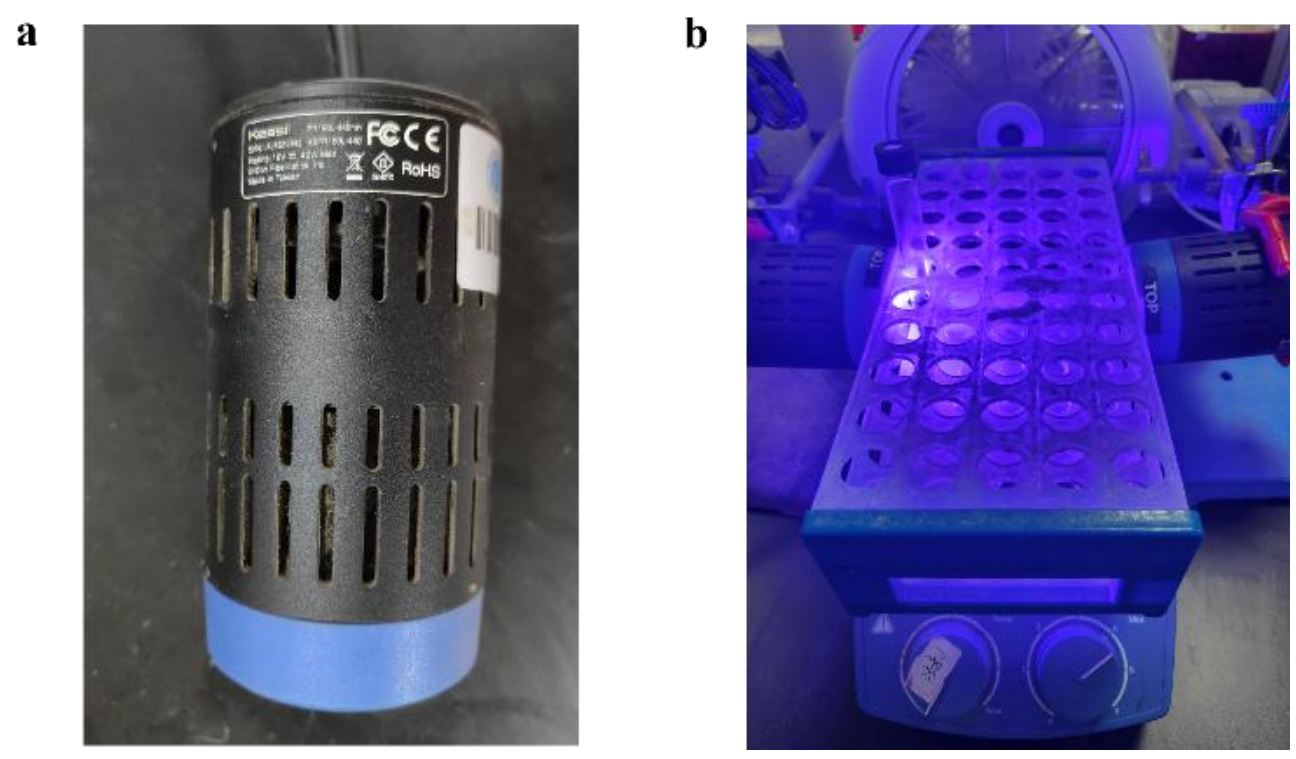

Figure S1. a. Kessil PR160L-440 nm LED light source. **b**. Reaction vials irradiated under the LED lamp, with the distance between light source and reaction vial fixed at 1.5 cm.

2.3. Gram-Scale Synthesis for Products 2

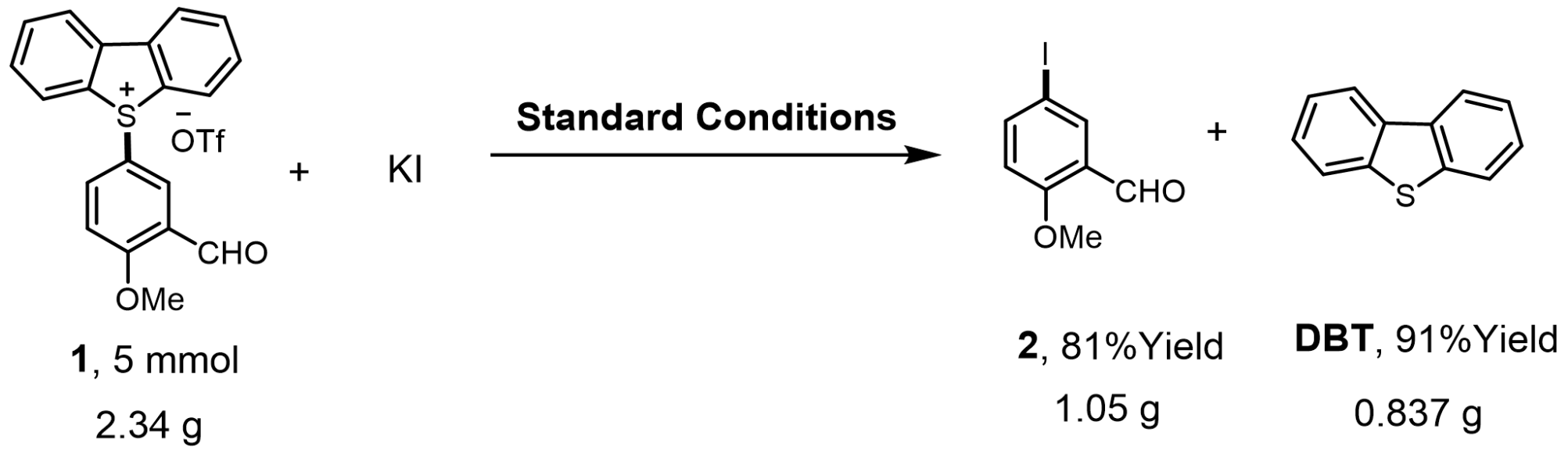

Compound **2** was synthesized following the **General Procedure for Aryl iodides**. The reaction of the corresponding aryl DBT salt **1** (2.34 g, 5.0 mmol, 1.0 equiv.), KI (1.25

g, 7.5 mmol, 1.5 equiv.) in CH_3CN (50.0 mL) delivered **2** (1.05g, 81%) as a white solid after purification via silica gel column chromatography with 10% ethyl acetate/hexanes (R_f = 0.5, 10% ethyl acetate/hexanes).

2.4. Synthetic Application

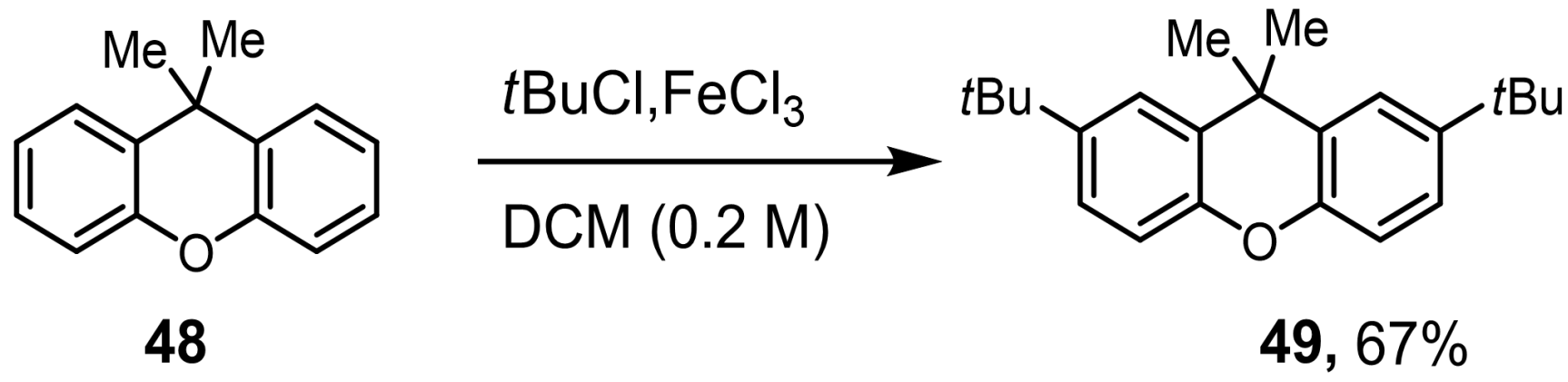

Compound **48** (1.0 g, 4.76 mmol, 1.0 equiv.) $FeCl_3$ (0.038 g, 0.24 mmol, 2.45 equiv.) in DCM 0°C was added *t*BuCl (1.3 mL, 11.65 mmol, 2.45 equiv.) dropwise, and the mixture was stirred for 3 h, and then for an additional 15 h at room temperature. The mixture was concentrated, poured into saturated aqueous NaCl solution (15 mL). The organic layers were separated, and the water was extracted with ethyl dichloromethane (15 mL), dried over Na_2SO_4. The residue was purified to give **49** (1.03 g, 67%) as a white solid after purification via silica gel column chromatography with 1% ethyl acetate/hexanes (Rf = 0.7, 1% ethyl acetate/hexanes). **^{1}H NMR** (600 MHz, $CDCl_3$) δ 7.41 (d, *J* = 2.4 Hz, 2H), 7.21 (dd, *J* = 2.4, 2.4 Hz, 2H), 6.96 (d, *J* = 8.4 Hz, 2H), 1.65 (s, 6H), 1.34 (s, 18H); **^{13}C NMR** (150 MHz, $CDCl_3$) δ 148.3, 145.4, 129.2, 124.3, 122.6, 115.6, 34.4, 34.3, 32.5, 31.6; **HRMS (ESI$^+$)** Calcd for $C_{23}H_{30}O$ $[M+H]^+$ 323.2369, found 266.1752.

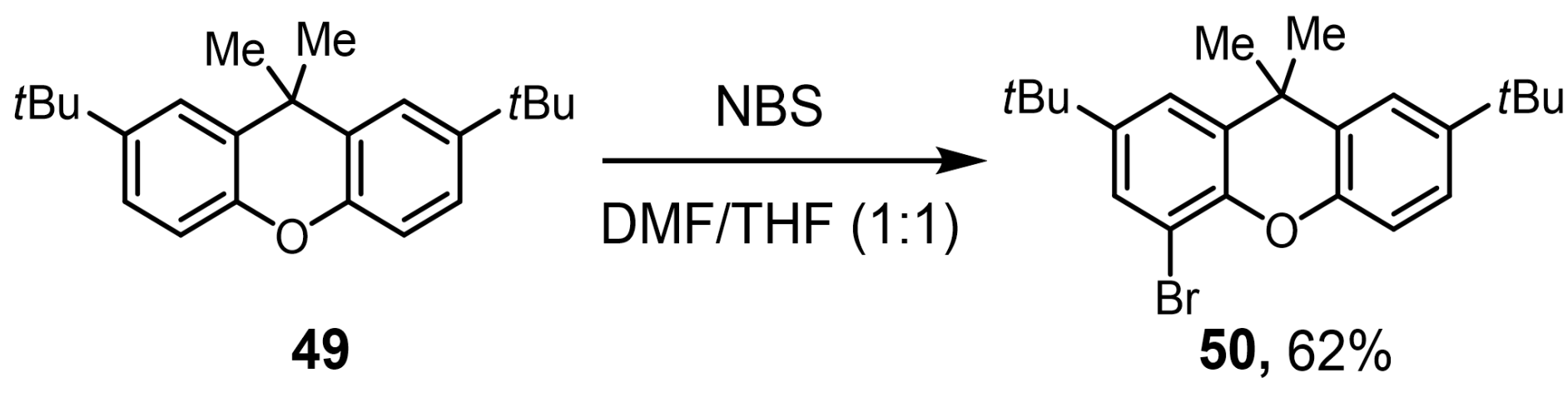

Compound **49** (0.5 g, 1.55 mmol, 1.0 equiv.) and NBS (2.49 g, 13.97 mmol, 9.0 equiv.) in DMF/THF (1:1=15mL:15mL), and the mixture was stirred at 30 °C overnight. The residue was purified to give **50** (0.95 g, 62%) as a white solid after purification via silica gel column chromatography with 1% ethyl acetate/hexanes (Rf = 0.8, 1% ethyl

acetate/hexanes). **^{1}H NMR** (600 MHz, $CDCl_3$) δ 7.44 (s, 1H), 7.38 (s, 1H), 7.34 (s, 1H), 7.23 (d, *J* = 8.4 Hz, 1H), 7.08 (d, *J* = 8.4 Hz, 1H), 1.63 (s, 6H), 1.33 (s, 9H), 1.32 (s, 9H); **^{13}C NMR** (150 MHz, $CDCl_3$) δ 148.1, 146.4, 146.1, 145.3, 131.3, 129.0, 128.2, 124.4, 122.2, 121.9, 116.0, 110.0, 35.0, 34.5, 34.5, 32.2, 31.5, 31.4; **HRMS (ESI$^+$)** Calcd for $C_{23}H_{29}BrO$ $[M+H]^+$ 401.1475, found 340.1911.

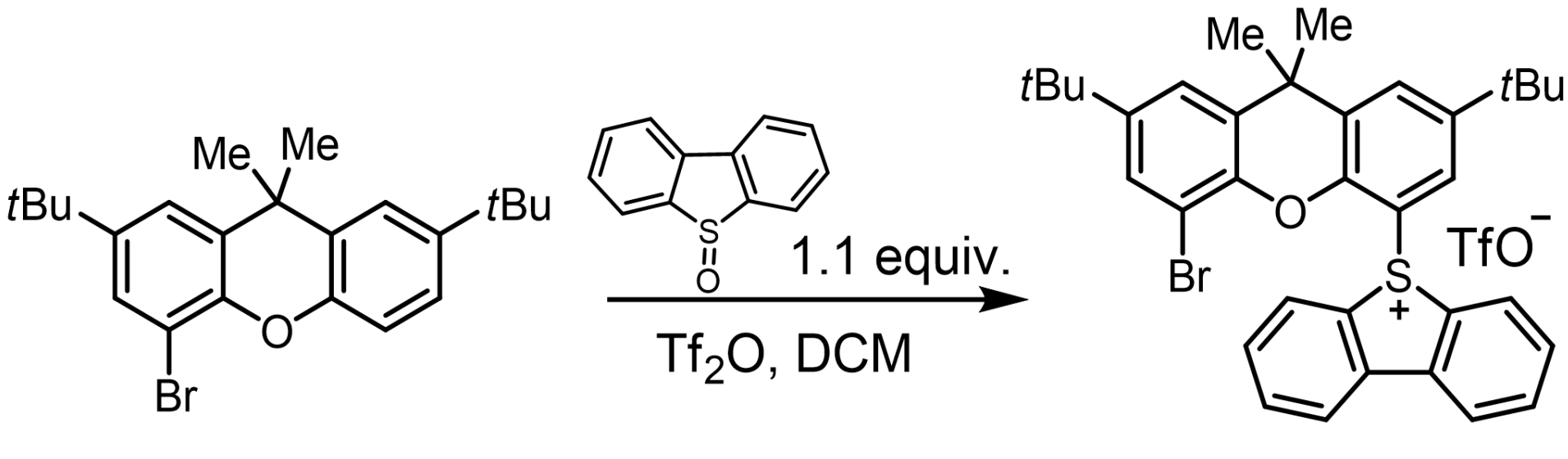

50 **51,** 81%

To a solution of dibenzothiopheneoxide (0.28 g, 1.38 mmol, 1.1 equiv.) and **50** (0.5g, 1.25 mmol, 1.0 equiv.) in dry dichloromethane (10 mL) at -40 °C, was added trifluoromethanesulfonic anhydride (0.423 g, 1.5 mmol, 1.2 equiv.) dropwise, and the mixture was stirred at this temperature for 1 h. The reaction was being warmed to room temperature, and stirred at this temperature for another 1 h. The mixture was concentrated, diluted with 15 mL dichloromethane, and poured into water (30 mL). The organic layers were separated, and the water was extracted with DCM (15 mL*2). The combined organic layer was washed with a saturated aqueous $NaHCO_3$ solution (15 mL), dried over Na_2SO_4, filtered and concentrated. The crude residue was dissolved in 2 mL dichloromethane, precipitated with ethyl ether. The suspension was decanted, and the solid was dried to give the dibenzothiophenium salt **51** (0.741 g, 81%).**^{1}H NMR** (600 MHz, $CDCl_3$) δ 8.40 (d, *J* = 7.8 Hz, 2H), 8.31 (d, *J* = 7.8Hz, 2H), 7.97 (t, *J* = 7.2 Hz, 2H), 7.74 (t, *J* = 7.8 Hz, 2H), 7.69 (s, 1H), 7.56 (s, 1H), 7.43 (s, 1H), 6.41 (s, 1H), 1.71 (s, 6H), 1.35 (s, 9H), 1.06 (m, 9H); **^{13}C NMR** (150 MHz, $CDCl_3$) δ 149.6, 149.2, 148.1, 143.2, 139.5, 132.9, 131.8, 130.6, 130.3, 129.1, 128.6, 128.5, 125.3, 122.5, 121.9, 120.7, 119.7, 113.1, 109.9, 60.3, 53.4, 35.6, 35.1, 34.8, 32.5, 31.2, 30.8, 20.9, 14.1; **HRMS (ESI$^+$)** Calcd for $C_{36}H_{36}BrOF_3O_4S_2$ $[M+H]^+$ 733.1263, found 340.1911.

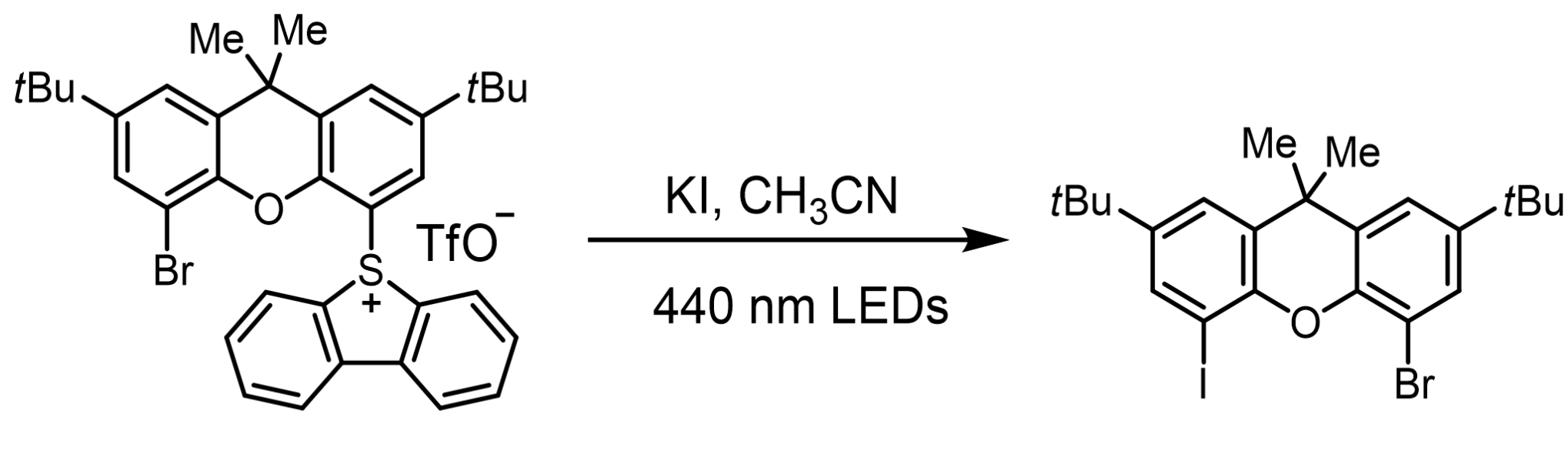

51 **52,** 56%

Compound **52** was synthesized following the **General Procedure for Aryl iodides** The reaction of the dibenzothiophenium salt **51** (0.146 g, 0.200 mmol, 1.0 equiv.), KI (49.8 mg, 0.300 mmol, 1.5 equiv.) in CH_3CN (2.0 mL) delivered **52** (58.9 mg, 56%) as a white solid after purification via silica gel column chromatography with hexanes (R_f = 0.7, hexanes). **^{1}H NMR** (600 MHz, $CDCl_3$) δ 7.70 (s, 1H), 7.49 (s, 1H), 7.37 (s, 1H), 7.33 (s, 1H), 1.62 (s, 6H), 1.32 (s, 18H); **^{13}C NMR** (150 MHz, $CDCl_3$) δ 147.9, 147.7, 147.2, 145.7, 134.6, 131.3, 130.4, 128.4, 122.7, 121.5, 110.3, 84.4, 35.8, 34.6, 34.4, 31.8, 31.4, 31.4; **HRMS (ESI$^+$)** Calcd for $C_{22}H_{26}BrIO$ $[M+H]^+$ 513.0284, found 340.1911.

2.5. Mechanism Studies

2.5.1 Control Experiments

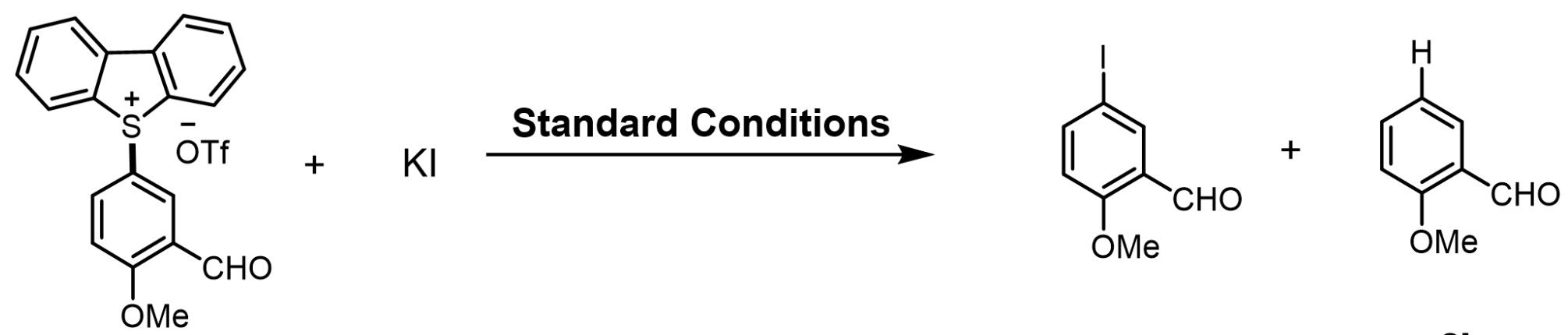

1 **2** **2'**

Entry	Condition	Yield (%)
**1**	none	84% (**2'**, 8%)
**2**	No KI	0 (**1**, 93%, **2'**, 6%)
**3**	no light	0 (**1**, 97%)

2.5.2 Radical- trapping with TEMPO

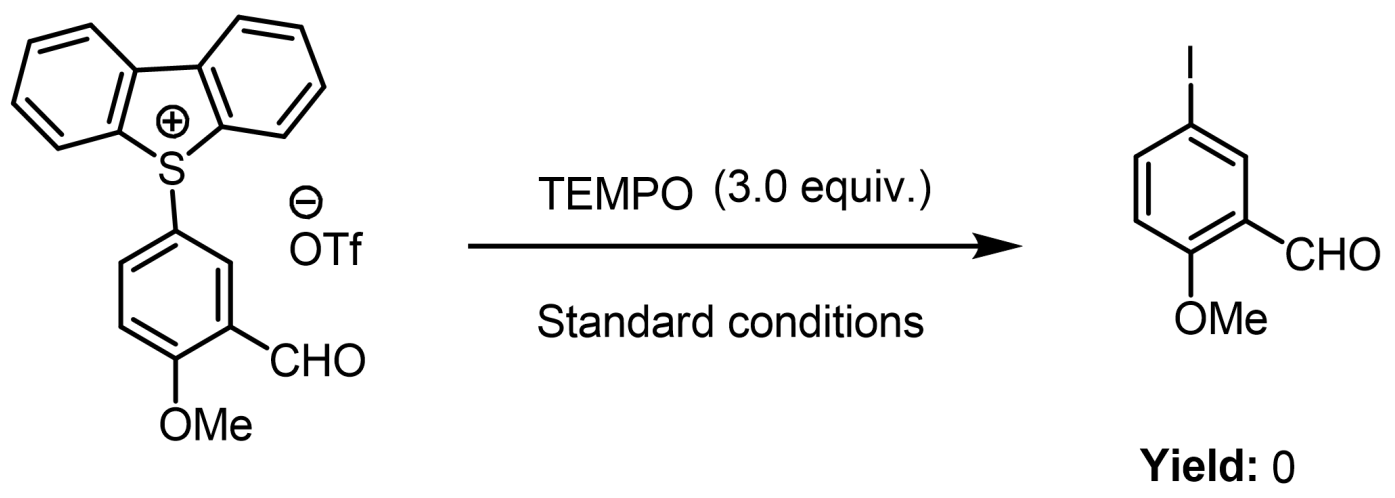

1 **2**

The dibenzothiophenium salt **1** (93.6 mg, 0.200 mmol, 1.0 equiv.), KI (49.8 mg, 0.300 mmol, 1.5 equiv.) and TEMPO (3.0 equiv.) were added, and CH_3CN (2.0 mL) were added and the mixture was stirred for 12 h irradiated by the LEDs at room temperature. After the reaction is complete, the reaction mixture was filtered through a pad of Celite. The diluted filtrate was analyzed by ^{1}HNMR and HRMS, and products **2** was not observed, the addut was also not detected by HRMS.

2.5.3 Radical- trapping with DMPO

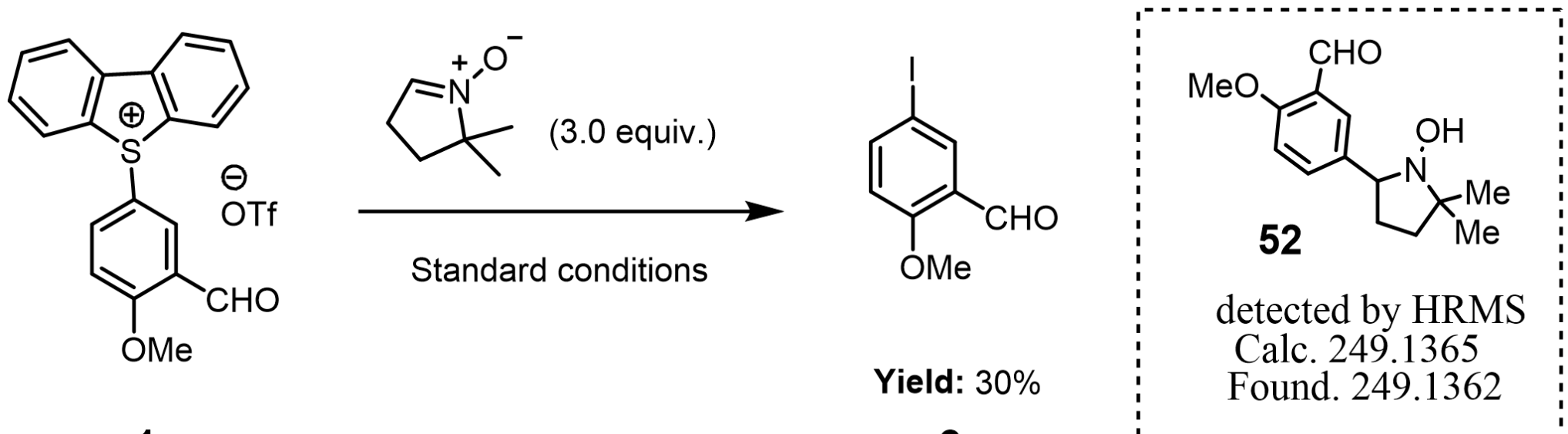

1 **2**

The dibenzothiophenium salt **1** (93.6 mg, 0.200 mmol, 1.0 equiv.), KI (49.8 mg, 0.300 mmol, 1.5 equiv.) and DMPO (3.0 equiv.) were added, and CH_3CN (2.0 mL) were added and the mixture was stirred for 12 h irradiated by the LEDs at room temperature. After the reaction is complete, the reaction mixture was filtered through a pad of Celite. The diluted filtrate was analyzed by ^{1}HNMR and HRMS, and products **2** was formed in 30% yield, meanwhile, radical intermediates **52** were detected by HRMS.

2.5.4 Radical trapping with 1,1-Diphenylethylene

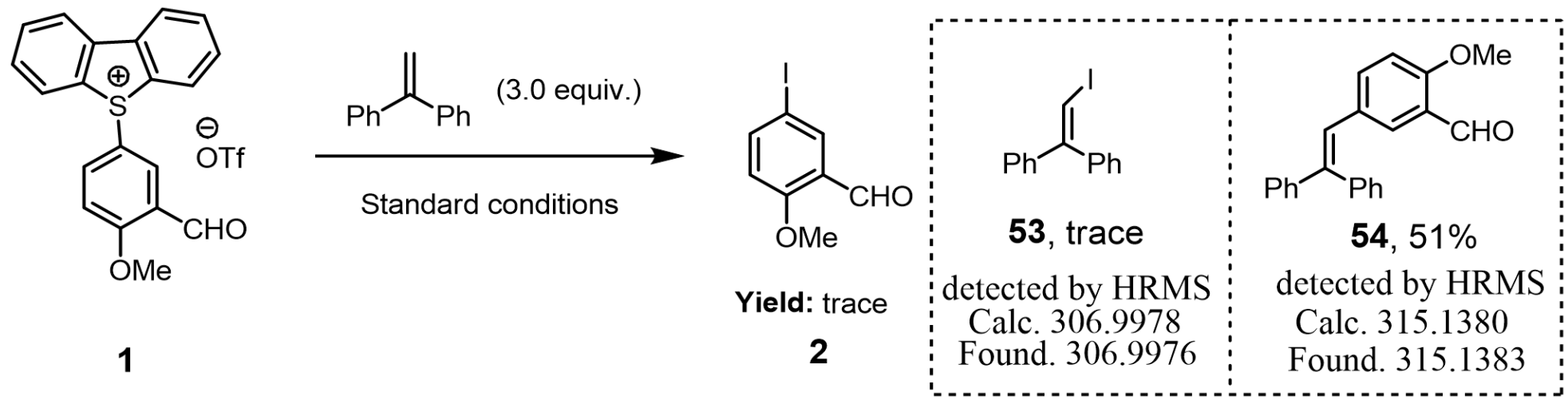

The dibenzothiophenium salt **1** (93.6 mg, 0.200 mmol, 1.0 equiv.), KI (49.8 mg, 0.300 mmol, 1.5 equiv.) and 1,1-diphenylethene (3.0 equiv.) were added, and CH_3CN (2.0 mL) were added and the mixture was stirred for 12 h irradiated by the LEDs at room temperature. After the reaction is complete, the reaction mixture was filtered through a pad of Celite. The diluted filtrate was analyzed by ^{1}HNMR and HRMS, and products **2** were detected, meanwhile, radical intermediates **53** and **54** also were detected.

2.5.5 Light on-off experiment

Light on-off experiments were carried out by following the procedure below. The dibenzothiophenium salt **1** (93.6 mg, 0.200 mmol, 1.0 equiv.), KI (49.8 mg, 0.300 mmol, 1.5 equiv.) were added, and CH_3CN (2.0 mL) were added and the mixture was stirred at room temperature alternating intervals of irradiation by blue LEDs and two-hour dark, during a total of 12 hours. The GC-MS yield of the product was monitored with dodecane as internal standard after each interval.

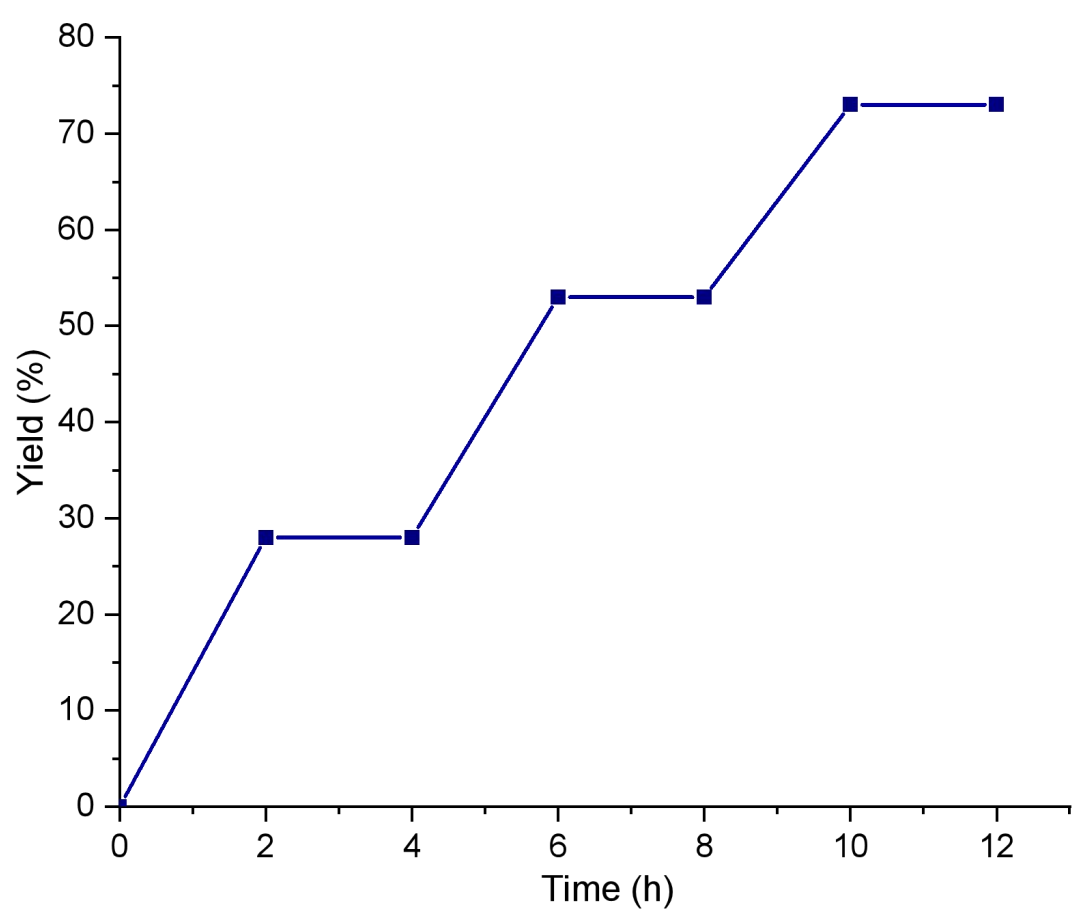

Figure S2. Light on-off experiment.

2.5.6 UV-vis absorption spectra

UV-Vis absorption spectra were recorded on a UNIC 4802 UV/VIS double-beam spectrophotometer using a 1 cm quartz cuvette cell. The spectra of Ar-DBT salt **1**, KI, and their mixture were measured in CH_3CN at a concentration of $5*10^{-4}$ M for each component. The mixed sample was freshly prepared and allowed to stand in the dark for 30 minutes prior to measurement to ensure complete equilibration of the EDA complex while minimizing light-induced decomposition. All measurements were performed under air at room temperature.

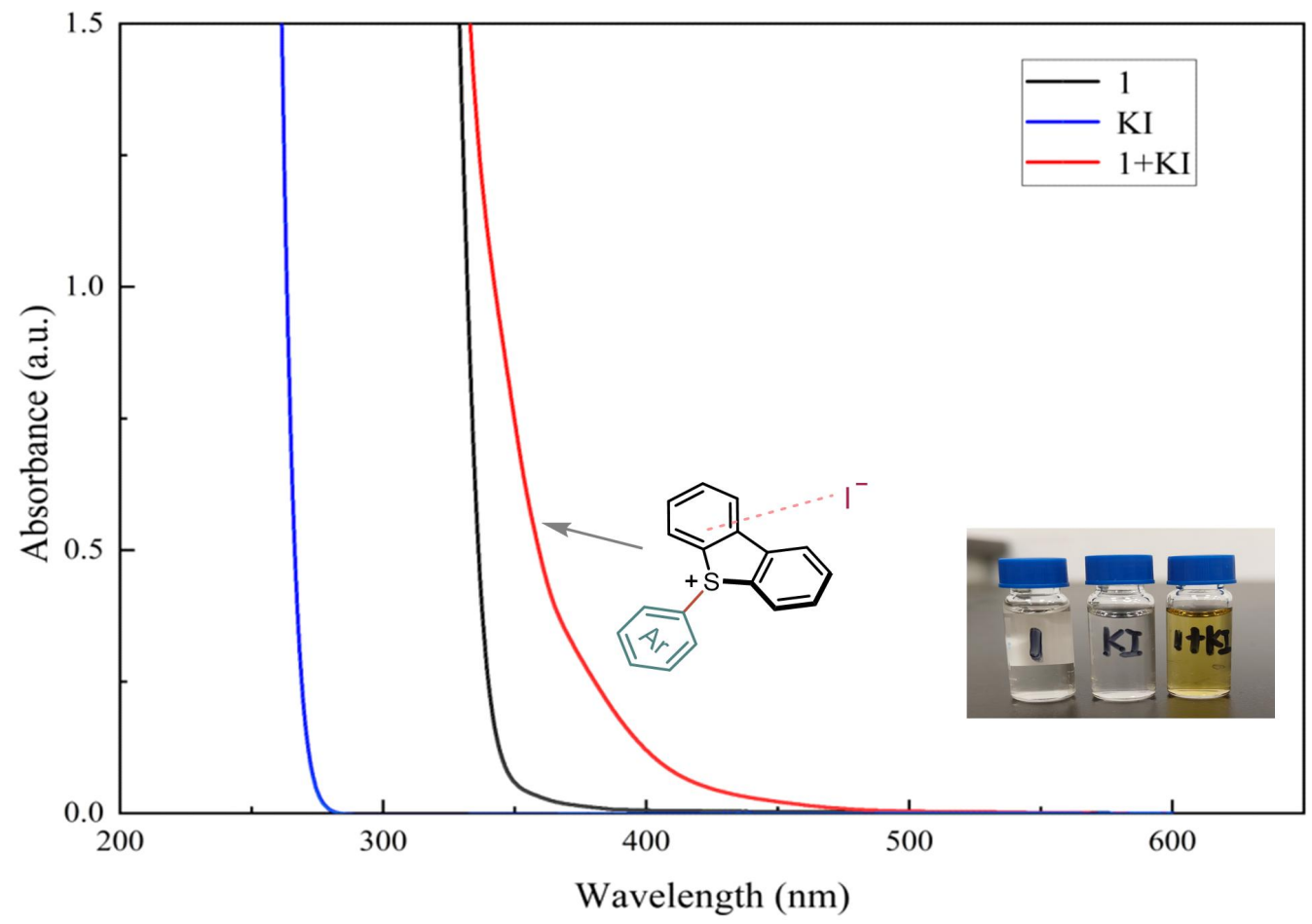

Figure S3. UV-vis absorption spectra in CH_3CN.

2.5.7 Quantum yield measurement

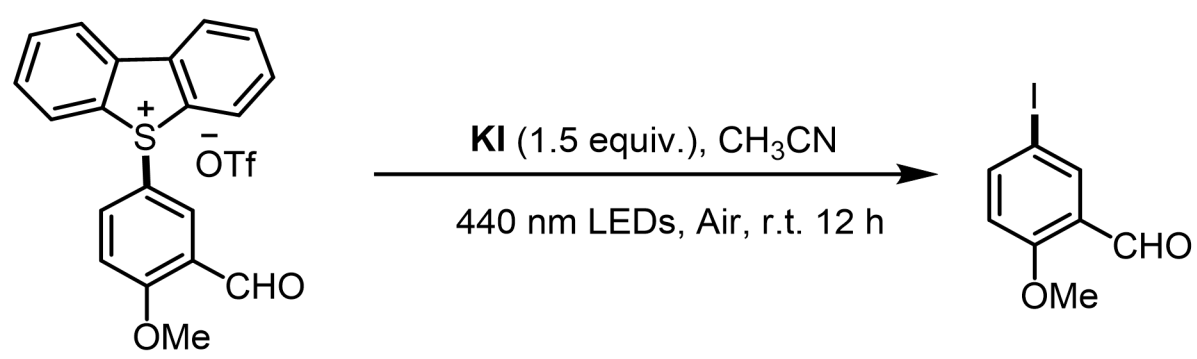

1 **2**

The aryl DBT salt **1** (0.2 mmol, 1.0 equiv.) and KI (0.3 mmol, 1.5 equiv.) were added to a solution in CH_3CN, then stirred under irradiation (λ = 440 nm, with intensity of 5.32 mW/cm^2) by LEDs at room temperature for 7200 s. Upon completion, the yield

of product **2** was determined by ^{1}HNMR based on 1,3,5-trimethoxybenzene standard. The quantum yield Φ was determined as follows.

$$\emptyset = \frac{n_2 N_A/t}{fP\lambda/hc}$$

Notes: n_2: the mole number (5.6×10^{-5} mol) of the product **2**; *t*: reaction time (7200 s); N_A: 6.02×10^{23}/mol; *f*: $1\text{-}10^{-A}$ (440 nm, A =0.28); P: P = E*S (E = 5.32 mW/cm^2, S: the area that irradiated S = 2.98 cm^2); λ: wavelength (4.4×10^{-7} m); *h*: planck constant (6.626×10^{-34} J*s); c: speed of light (3×10^8 m/s).

$$\emptyset = 0.28$$

2.5.8 Determination of the binding by Job's plot

Job's plot was performed according to the literature method.[27] The aryl DBT salt **1** (0.3 mmol) was dissolved in CD_3CN (3.0 mL) to give a stock solution at 0.1 mol/L concentration. Similarly, TBAI (0.3 mmol) was added to CD_3CN (3.0 mL) solution to provide a 0.1 mol/L solution. Then, the solutions of **1** and TBAI were added to the NMR tube based on the specific ratio in Table S5. The 1H(Ar-H) chemical shift of free **1** solution (0.1 mol/L) is 7.2944 ppm. The job's plot had a maximum when X (**1**) equals to 0.5, suggesting that the binding stoichiometry between **1** and TBAI is 1:1.

Table S5. ^{1}H NMR experimental data for Job's plot

Entry	V (**1**, mL)	V (TBAI, mL)	X	δ(^{1}H(Ar-H), ppm)	Δδ	Δδ*X (**1**)
1	0.45	0.05	0.9	7.2956	0.0012	0.00108
2	0.40	0.10	0.8	7.2963	0.0019	0.00152
3	0.35	0.15	0.7	7.2974	0.0030	0.00210
4	0.30	0.20	0.6	7.2983	0.0039	0.00234
5	0.25	0.25	0.5	7.2992	0.0048	0.00240
6	0.20	0.30	0.4	7.2997	0.0053	0.00212
7	0.15	0.35	0.3	7.3012	0.0068	0.00204
8	0.10	0.40	0.2	7.3019	0.0075	0.00150

9	0.05	0.45	0.1	7.3025	0.0081	0.00081

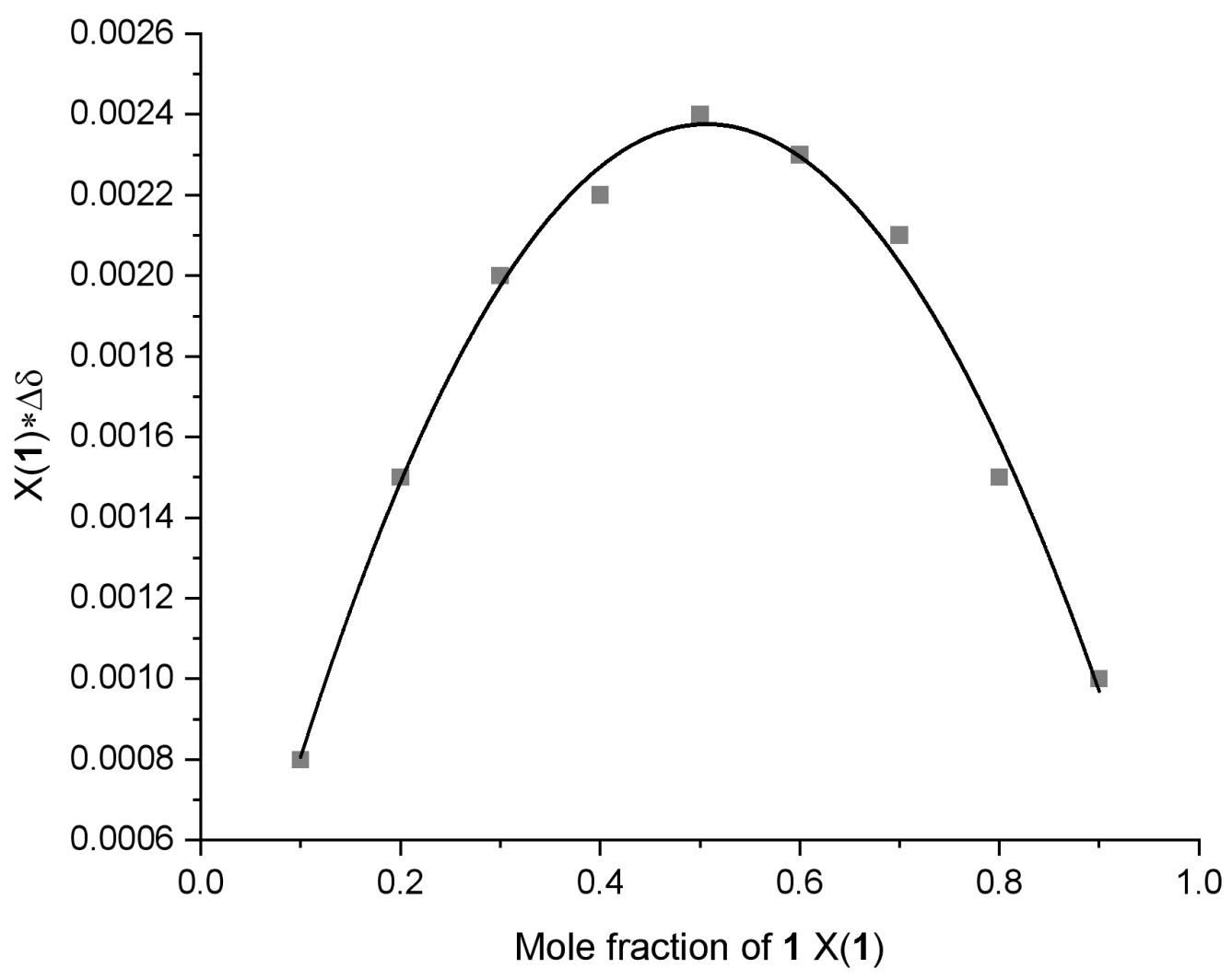

Figure S3. Job's plot.

2.6. Characterization Date of All Products

iodobenzene

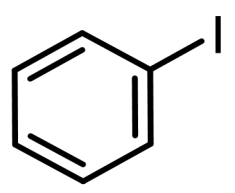

Compound **3**[2] was synthesized following the **General Procedure for Aryl iodides**. The reaction of the corresponding aryl DBT salt (82 mg, 0.200 mmol, 1.0 equiv.), KI (49.8 mg, 0.300 mmol, 1.5 equiv.) in CH_3CN (2.0 mL) delivered **3** (31.8 mg, 78%) as a white solid after purification via silica gel column chromatography with hexanes (R_f = 0.9, hexanes). **^{1}H NMR** (600 MHz, $CDCl_3$) δ 7.71 (d, *J* = 7.8 Hz, 2H), 7.33 (t, *J* = 7.2 Hz, 1H), 7.11 (t, *J* = 7.8 Hz, 2H); **^{13}C NMR** (150 MHz, $CDCl_3$) δ 137.5, 130.2, 127.4, 94.4.

1-iodo-4-isopropylbenzene

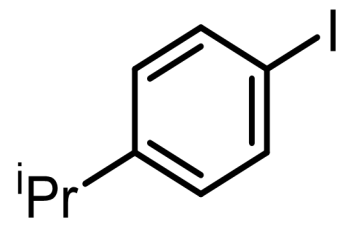

Compound **4**[2] was synthesized following the **General Procedure for Aryl iodides.** The reaction of the corresponding aryl DBT salt (90 mg, 0.200 mmol, 1.0 equiv.), KI (49.8 mg, 0.300 mmol, 1.5 equiv.) in CH_3CN (2.0 mL) delivered **4** (34.4 mg, 70%) as a white solid after purification via silica gel column chromatography with hexanes (R_f = 0.8,

hexanes). **^{1}H NMR** (600 MHz, $CDCl_3$) δ 7.60 (d, *J* = 7.8 Hz, 2H), 6.98 (d, *J* = 7.8 Hz, 2H), 2.92-2.79 (m, 1H), 1.23 (d, *J* = 6.6 Hz, 6H); **^{13}C NMR** (150 MHz, $CDCl_3$) δ 148.5, 137.3, 128.6, 90.6, 33.7, 23.8.

1-(tert-butyl)-4-iodobenzene

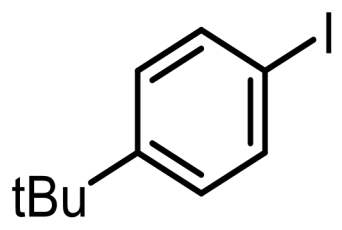

Compound **5**[3] was synthesized following the **General Procedure for Aryl iodides.** The reaction of the corresponding aryl DBT salt (93 mg, 0.200 mmol, 1.0 equiv.), KI (49.8 mg, 0.300 mmol, 1.5 equiv.) in CH_3CN (2.0 mL) delivered **5** (44.7 mg, 86%) as a white solid after purification via silica gel column chromatography with hexanes (R_f = 0.8, hexanes). **^{1}H NMR** (600 MHz, $CDCl_3$) δ 7.62 (s, *J* = 8.4 Hz, 2H), 7.14 (d, *J* = 8.4 Hz, 2H), 1.30 (s, 9H); **^{13}C NMR** (150 MHz, $CDCl_3$) δ 150.8, 137.0, 127.6, 90.6, 34.6, 31.1.

3-(4-iodophenyl)propyl acetate

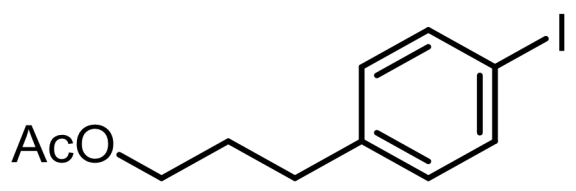

Compound **6** was synthesized following the **General Procedure for Aryl iodides.** The reaction of the corresponding aryl DBT salt (0.102 g, 0.200 mmol, 1.0 equiv.), KI (49.8 mg, 0.300 mmol, 1.5 equiv.) in CH_3CN (2.0 mL) delivered **6** (34.7 mg, 57%) as a white solid after purification via silica gel column chromatography with 5% ethyl acetate/hexanes (R_f = 0.5, 5% ethyl acetate/hexanes). **^{1}H NMR** (600 MHz, $CDCl_3$) δ 7.60 (d, *J* = 7.8 Hz, 2H), 6.93 (d, *J* = 8.4 Hz, 2H), 4.06 (t, *J* = 6.0 Hz, 2H), 2.63 (t, *J* = 7.8 Hz, 2H), 2.04 (s, 3H), 1.94-1.90 (m, 2H); **^{13}C NMR** (150 MHz, $CDCl_3$) δ 171.0, 140.8, 137.4, 130.5, 91.0, 63.5, 31.7, 29.9, 20.9; **HRMS (ESI$^+$)** Calcd for $C_{11}H_{13}IO_2$ $[M+Na]^+$ 326.9852, found 326.9850.

4-iodobenzyl benzoate

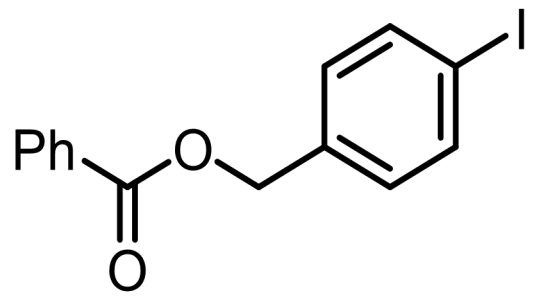

Compound **7**[14] was synthesized following the **General Procedure for Aryl iodides.** The reaction of the corresponding aryl DBT salt (0.109 g, 0.200 mmol, 1.0 equiv.), KI

(49.8 mg, 0.300 mmol, 1.5 equiv.) in CH_3CN (2.0 mL) delivered **7** (34.5 mg, 51%) as a white solid after purification via silica gel column chromatography with 10% ethyl acetate/hexanes (R_f = 0.5, 10% ethyl acetate/hexanes). **^{1}H NMR** (600 MHz, $CDCl_3$) δ 8.06 (d, *J* = 7.8 Hz, 2H), 7.72 (d, *J* = 7.8 Hz, 2H), 7.57 (t, *J* = 7.8 Hz, 1H), 7.44 (t, *J* = 7.8 Hz, 2H), 7.20 (d, *J* = 7.8 Hz, 2H), 5.30 (s, 2H); **^{13}C NMR** (150 MHz, $CDCl_3$) δ 166.3, 137.7, 135.7, 133.2, 130.0, 129.9, 129.7, 128.4, 93.9, 65.9.

1-iodo-4-methoxybenzene

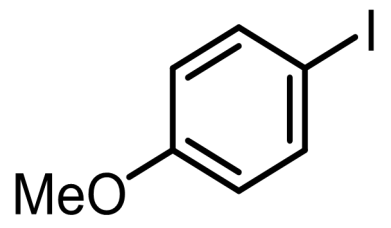

Compound **8**[4] was synthesized following the **General Procedure for Aryl iodides.** The reaction of the corresponding aryl DBT salt (88 mg, 0.200 mmol, 1.0 equiv.), KI (49.8 mg, 0.300 mmol, 1.5 equiv.) in CH_3CN (2.0 mL) delivered **8** (38.3 mg, 82%) as a white solid after purification via silica gel column chromatography with hexanes (R_f = 0.9, hexanes). **^{1}H NMR** (600 MHz, $CDCl_3$) δ 7.56 (d, *J* = 8.4 Hz, 2H), 6.68 (d, *J* = 8.4 Hz, 2H), 3.78 (s, 3H); **^{13}C NMR** (150 MHz, $CDCl_3$) δ 159.4, 138.2, 116.3, 82.7, 55.3.

1-iodo-4-(pent-2-yn-1-yloxy)benzene

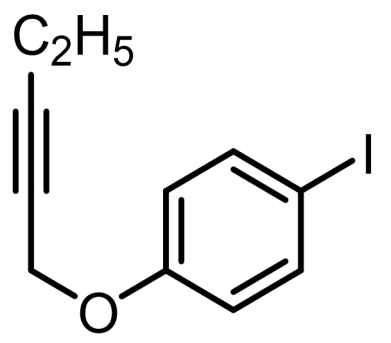

Compound **9**[22] was synthesized following the **General Procedure for Aryl iodides.** The reaction of the corresponding aryl DBT salt (98 mg, 0.200 mmol, 1.0 equiv.), KI (49.8 mg, 0.300 mmol, 1.5 equiv.) in CH_3CN (2.0 mL) delivered **9** (32.0 mg, 56%) as a white solid after purification via silica gel column chromatography with 10% ethyl acetate/hexanes (R_f = 0.5, 10% ethyl acetate/hexanes). **^{1}H NMR** (600 MHz, $CDCl_3$) δ 7.56 (d, *J* = 7.8 Hz, 2H), 6.75 (d, *J* = 7.8 Hz, 2H), 4.63 (s, 2H), 2.23 (q, *J* = 7.2 Hz, 2H), 1.13 (t, *J* = 7.8 Hz, 3H); **^{13}C NMR** (150 MHz, $CDCl_3$) δ 157.7, 138.1, 117.3, 90.0, 83.4, 73.7, 56.5, 13.6, 12.4.

N-(4-iodophenyl)acetamide

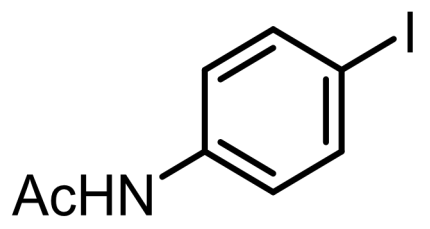

Compound **10**[25] was synthesized following the **General Procedure for Aryl iodides.** The reaction of the corresponding aryl DBT salt (93 mg, 0.200 mmol, 1.0 equiv.), KI (49.8 mg, 0.300 mmol, 1.5 equiv.) in CH_3CN (2.0 mL) delivered **10** (25.5 mg, 49%) as a white solid after purification via silica gel column chromatography with 10% ethyl acetate/hexanes (R_f = 0.5, 10% ethyl acetate/hexanes). **^{1}H NMR** (600 MHz, $CDCl_3$) δ 7.61 (d, *J* = 7.8 Hz, 2H), 7.29 (d, *J* = 8.4 Hz, 2H), 7.21 (s, 1H), 2.17 (s, 3H); **^{13}C NMR** (150 MHz, $CDCl_3$) δ 168.2, 137.9, 137.6, 121.6, 87.4, 24.6.

1-(4-iodophenyl)pyrrolidin-2-one

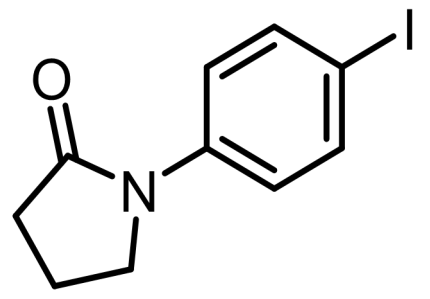

Compound **11**[21] was synthesized following the **General Procedure for Aryl iodides.** The reaction of the corresponding aryl DBT salt (99 mg, 0.200 mmol, 1.0 equiv.), KI (49.8 mg, 0.300 mmol, 1.5 equiv.) in CH_3CN (2.0 mL) delivered **11** (30.4 mg, 53%) as a white solid after purification via silica gel column chromatography with 10% ethyl acetate/hexanes (R_f = 0.5, 10% ethyl acetate/hexanes). **^{1}H NMR** (600 MHz, $CDCl_3$) δ 7.65 (d, *J* = 7.8 Hz, 2H), 7.41 (d, *J* = 7.8 Hz, 2H), 3.82 (t, *J* = 6.6 Hz, 2H), 2.60 (t, *J* = 8.4 Hz, 2H), 2.20-2.12 (m, 2H); **^{13}C NMR** (150 MHz, $CDCl_3$) δ 174.2, 139.2, 137.7, 121.5, 87.9, 48.4, 32.7, 17.8.

1-fluoro-4-iodobenzene

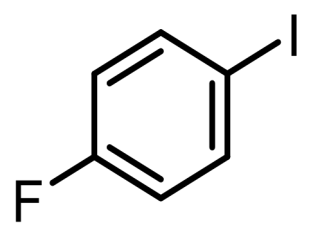

Compound **12**[13] was synthesized following the **General Procedure for Aryl iodides.** The reaction of the corresponding aryl DBT salt (86 mg, 0.200 mmol, 1.0 equiv.), KI (49.8 mg, 0.300 mmol, 1.5 equiv.) in CH_3CN (2.0 mL) delivered **12** (28.0 mg, 63%) as a white solid after purification via silica gel column chromatography with hexanes (R_f = 0.9, hexanes). **^{1}H NMR** (600 MHz, $CDCl_3$) δ 7.63 (t, *J* = 6.6 Hz, 2H), 6.84 (t, *J* = 8.4

Hz, 2H); **^{13}C NMR** (150 MHz, $CDCl_3$) δ 163.6, 161.9, 139.0, 138.9, 117.9, 117.7, 86.9, 86.9; **^{19}F NMR** (565 MHz, $CDCl_3$) δ -114.4.

1-chloro-4-iodobenzene

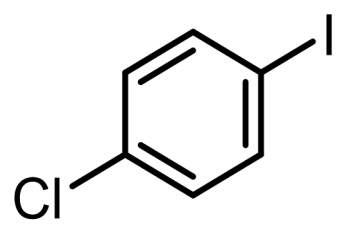

Compound **13**[12] was synthesized following the **General Procedure for Aryl iodides.** The reaction of the corresponding aryl DBT salt (89 mg, 0.200 mmol, 1.0 equiv.), KI (49.8 mg, 0.300 mmol, 1.5 equiv.) in CH_3CN (2.0 mL) delivered **13** (32.4 mg, 68%) as a white solid after purification via silica gel column chromatography with hexanes (R_f = 0.9, hexanes). **^{1}H NMR** (600 MHz, $CDCl_3$) δ 7.61 (d, J = 7.8 Hz, 2H), 7.09 (d, J = 7.8 Hz, 2H); **^{13}C NMR** (150 MHz, $CDCl_3$) δ 138.7, 134.2, 130.5, 91.1.

1-bromo-4-iodobenzene

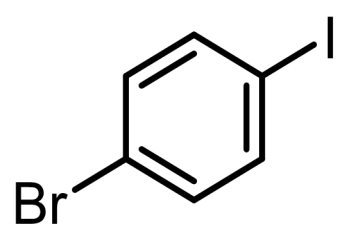

Compound **14**[11] was synthesized following the **General Procedure for Aryl iodides.** The reaction of the corresponding aryl DBT salt (98 mg, 0.200 mmol, 1.0 equiv.), KI (49.8 mg, 0.300 mmol, 1.5 equiv.) in CH_3CN (2.0 mL) delivered **14** (41.2 mg, 73%) as a white solid after purification via silica gel column chromatography with hexanes (R_f = 0.9, hexanes). **^{1}H NMR** (600 MHz, $CDCl_3$) δ 7.54 (d, J = 7.8 Hz, 2H), 7.23 (d, J = 7.8 Hz, 2H); **^{13}C NMR** (150 MHz, $CDCl_3$) δ 139.0, 133.4, 122.2, 92.0.

5-iodo-2-methylthiophene

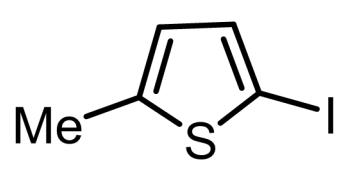

Compound **15**[10] was synthesized following the **General Procedure for Aryl iodides.** The reaction of the corresponding aryl DBT salt (86 mg, 0.200 mmol, 1.0 equiv.), KI (49.8 mg, 0.300 mmol, 1.5 equiv.) in CH_3CN (2.0 mL) delivered **15** (21.5 mg, 48%) as a white solid after purification via silica gel column chromatography hexanes (R_f = 0.9, hexanes). **^{1}H NMR** (600 MHz, $CDCl_3$) δ 7.02 (s, 1H), 6.46 (s, 1H), 7.48 (s, 3H); **^{13}C NMR** (150 MHz, $CDCl_3$) δ 145.9, 136.8, 127.0, 69.3, 15.3.

1-(5-iodothiophen-2-yl)ethan-1-one

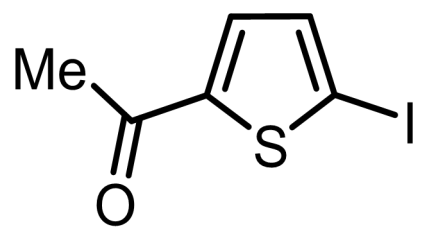

Compound **16**[17] was synthesized following the **General Procedure for Aryl iodides.** The reaction of the corresponding aryl DBT salt (92 mg, 0.200 mmol, 1.0 equiv.), KI (49.8 mg, 0.300 mmol, 1.5 equiv.) in CH_3CN (2.0 mL) delivered **16** (22.4 mg, 45%) as a white solid after purification via silica gel column chromatography with 5% ethyl acetate/hexanes (R_f = 0.5, 5% ethyl acetate/hexanes). **^{1}H NMR** (600 MHz, $CDCl_3$) δ 7.00 (s, 1H), 6.83 (s, 1H), 3.92 (s, 3H), 2.40 (s, 3H); **^{13}C NMR** (150 MHz, $CDCl_3$) δ 187.8, 135.0, 132.6, 126.0, 58.7, 37.7, 27.1.

2-iodothieno[3,2-b]thiophene

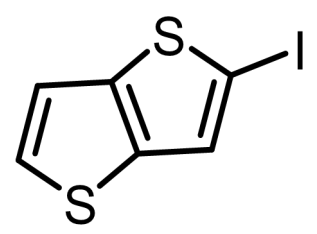

Compound **17**[25] was synthesized following the **General Procedure for Aryl iodides.** The reaction of the corresponding aryl DBT salt (94 mg, 0.200 mmol, 1.0 equiv.), KI (49.8 mg, 0.300 mmol, 1.5 equiv.) in CH_3CN (2.0 mL) delivered **17** (22.3 mg, 42%) as a white solid after purification via silica gel column chromatography with hexanes (R_f = 0.9, hexanes). **^{1}H NMR** (600 MHz, $CDCl_3$) δ 7.42 (s, 1H), 7.40 (d, J = 4.8 Hz, 1H), 7.19 (d, J = 5.4 Hz, 1H); **^{13}C NMR** (150 MHz, $CDCl_3$) δ 143.9, 139.4, 128.3, 127.0, 118.6, 74.9.

5-iodobenzo[d][1,3]dioxole

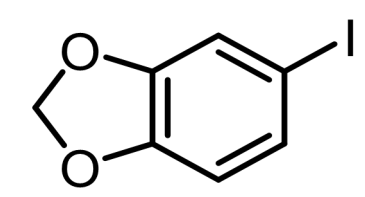

Compound **18**[3] was synthesized following the **General Procedure for Aryl iodides.** The reaction of the corresponding aryl DBT salt (91 mg, 0.200 mmol, 1.0 equiv.), KI (49.8 mg, 0.300 mmol, 1.5 equiv.) in CH_3CN (2.0 mL) delivered **18** (33.7 mg, 68%) as a white solid after purification via silica gel column chromatography with hexanes (R_f = 0.7, 10% ethyl acetate/hexanes). **^{1}H NMR** (600 MHz, $CDCl_3$) δ 7.18-7.09 (m, 2H), 6.59 (d, J = 7.8 Hz, 1H), 5.95 (s, 2H); **^{13}C NMR** (150 MHz, $CDCl_3$) δ 148.7, 147.8, 130.6, 117.7, 110.4, 101.4, 82.2.

2,2-difluoro-5-iodobenzo[d][1,3]dioxole

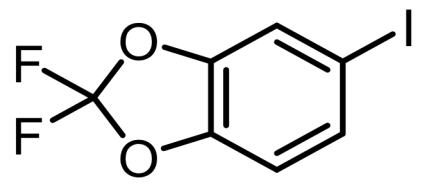

Compound **19**[18] was synthesized following the **General Procedure for Aryl iodides.** The reaction of the corresponding aryl DBT salt (98 mg, 0.200 mmol, 1.0 equiv.), KI (49.8 mg, 0.300 mmol, 1.5 equiv.) in CH_3CN (2.0 mL) delivered **19** (33.5 mg, 59%) as a white solid after purification via silica gel column chromatography with hexanes (R_f = 0.8, hexanes). **^{1}H NMR** (600 MHz, $CDCl_3$) δ 7.46-7.35 (m, 2H), 6.84 (d, J = 8.4 Hz, 1H); **^{13}C NMR** (150 MHz, $CDCl_3$) δ 144.4, 143.8, 133.0, 132.7, 131.3, 129.6, 118.7, 111.4, 84.6; **^{19}F NMR** (565 MHz, $CDCl_3$) δ -49.9.

6-iodo-2,3-dihydrobenzo[b][1,4]dioxine

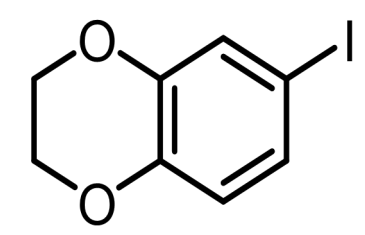

Compound **20**[23] was synthesized following the **General Procedure for Aryl iodides.** The reaction of the corresponding aryl DBT salt (94 mg, 0.200 mmol, 1.0 equiv.), KI (49.8 mg, 0.300 mmol, 1.2 equiv.) in CH_3CN (2.0 mL) delivered **20** (27.8 mg, 53%) as a white solid after purification via silica gel column chromatography with hexanes (R_f = 0.8, hexanes). **^{1}H NMR** (600 MHz, $CDCl_3$) δ 7.18 (s, 1H), 7.12 (d, J = 8.4 Hz, 1H), 6.61 (d, J = 8.4 Hz, 1H), 4.23 (s, 4H); **^{13}C NMR** (150 MHz, $CDCl_3$) δ 144.6, 143.7, 130.0, 126.0, 119.1, 82.4, 64.2.

7-iodochroman-4-one

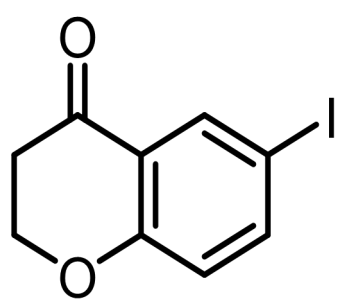

Compound **21**[3] was synthesized following the **General Procedure for Aryl iodides.** The reaction of the corresponding aryl DBT salt (96 mg, 0.200 mmol, 1.0 equiv.), KI (49.8 mg, 0.300 mmol, 1.5 equiv.) in CH_3CN (2.0 mL) delivered **21** (30.6 mg, 56%) as a white solid after purification via silica gel column chromatography with 2% ethyl acetate/hexanes (R_f = 0.5, 2% ethyl acetate/hexanes). **^{1}H NMR** (600 MHz, $CDCl_3$) δ 8.19 (s, 1H), 7.72 (d, J = 9.0 Hz, 1H), 6.76 (d, J = 9.0 Hz, 1H), 4.53 (t, J = 6.6 Hz, 2H),

2.80 (t, J = 6.6 Hz, 2H); ^{13}**C NMR** (150 MHz, $CDCl_3$) δ 190.4, 161.4, 144.2, 135.8, 123.1, 120.3, 83.7, 67.0, 37.3.

4-iodo-1,2-dimethoxybenzene

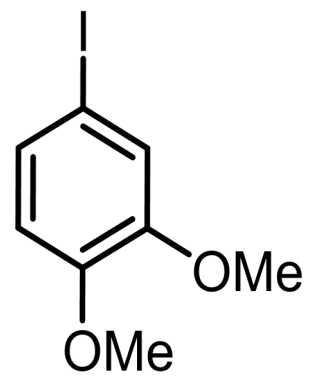

Compound **22**[24] was synthesized following the **General Procedure for Aryl iodides.** The reaction of the corresponding aryl DBT salt (94 mg, 0.200 mmol, 1.0 equiv.), KI (49.8 mg, 0.300 mmol, 1.5 equiv.) in CH_3CN (2.0 mL) delivered **22** (33.8 mg, 64%) as a white solid after purification via silica gel column chromatography with hexanes (R_f = 0.8, hexanes). ^{1}**H NMR** (600 MHz, $CDCl_3$) δ 7.23 (d, 1H), 7.12 (s, 1H), 6.62 (d, J = 8.4 Hz, 1H), 3.86 (s, 3H), 3.85 (s, 3H); ^{13}**C NMR** (150 MHz, $CDCl_3$) δ 149.8, 149.2, 129.8, 120.4, 113.2, 82.3, 56.1, 55.9.

2-fluoro-4-iodo-1-methoxybenzene

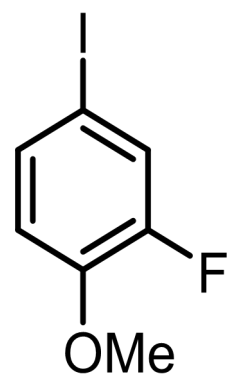

Compound **23**[3] was synthesized following the **General Procedure for Aryl iodides.** The reaction of the corresponding aryl DBT salt (92 mg, 0.200 mmol, 1.0 equiv.), KI (49.8 mg, 0.300 mmol, 1.5 equiv.) in CH_3CN (2.0 mL) delivered **23** (37.3 mg, 74%) as a white solid after purification via silica gel column chromatography with hexanes (R_f = 0.8, hexanes). ^{1}**H NMR** (600 MHz, $CDCl_3$) δ 7.44-7.34 (m, 2H), 6.71 (t, J = 8.4 Hz, 1H), 3.86 (s, 3H); ^{13}**C NMR** (150 MHz, $CDCl_3$) δ 153.2, 151.5, 147.9 (d, J = 10.5 Hz), 133.3, 133.3, 125.2, 125.0, 115.2, 80.9 (d, J = 7.5 Hz), 56.3; ^{19}**F NMR** (565 MHz, $CDCl_3$) δ -132.3.

2-chloro-4-iodo-1-methoxybenzene

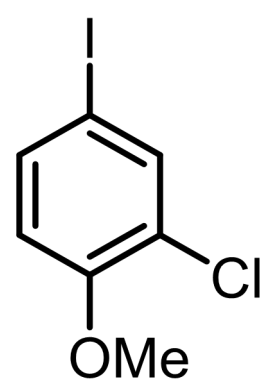

Compound **24**[3] was synthesized following the **General Procedure for Aryl iodides.** The reaction of the corresponding aryl DBT salt (95 mg, 0.200 mmol, 1.0 equiv.), KI (49.8 mg, 0.300 mmol, 1.5 equiv.) in CH_3CN (2.0 mL) delivered **24** (40.7 mg, 76%) as a white solid after purification via silica gel column chromatography with hexanes (R_f = 0.8, hexanes). **^{1}H NMR** (600 MHz, $CDCl_3$) δ 7.65 (s, 1H), 7.51 (d, J = 8.4 Hz, 1H), 6.68 (d, J = 8.4 Hz, 1H), 3.88 (s, 3H); **^{13}C NMR** (150 MHz, $CDCl_3$) δ 155.1, 138.2, 136.6, 123.8, 113.9, 81.8, 56.2.

2-bromo-4-iodo-1-methoxybenzene

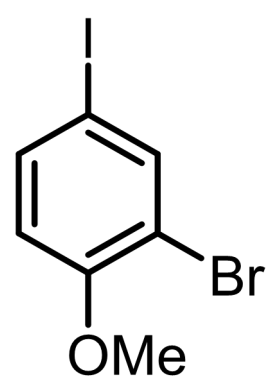

Compound **25**[3] was synthesized following the **General Procedure for Aryl iodides.** The reaction of the corresponding aryl DBT salt (103 mg, 0.200 mmol, 1.0 equiv.), KI (49.8 mg, 0.300 mmol, 1.5 equiv.) in CH_3CN (2.0 mL) delivered **25** (53.6 mg, 86%) as a white solid after purification via silica gel column chromatography with hexanes (R_f = 0.8, hexanes). **^{1}H NMR** (600 MHz, $CDCl_3$) δ 7.82 (s, 1H), 7.55 (d, J = 9.0 Hz, 1H), 6.66 (d, J = 8.4 Hz, 1H), 3.87 (s, 3H); **^{13}C NMR** (150 MHz, $CDCl_3$) δ 156.0, 141.0, 137.2, 113.8, 112.9, 82.4, 56.3.

2,4-diiodo-1-methoxybenzene

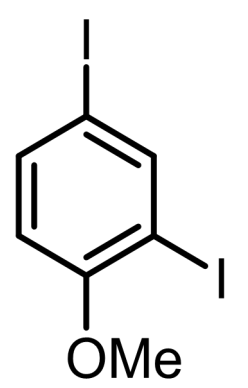

Compound **26**[8] was synthesized following the **General Procedure for Aryl iodides.** The reaction of the corresponding aryl DBT salt (0.113 g, 0.200 mmol, 1.0 equiv.), KI (49.8 mg, 0.300 mmol, 1.5 equiv.) in CH_3CN (2.0 mL) delivered **26** (44.5 mg, 62%) as a white solid after purification via silica gel column chromatography with hexanes (R_f

= 0.8, hexanes). **^{1}H NMR** (600 MHz, $CDCl_3$) δ 8.03 (d, *J* = 1.8 Hz, 1H), 7.57 (dd, *J* = 1.8 Hz, *J* =8.4 Hz, 1H), 6.58 (d, *J* = 9.0 Hz, 1H), 3.85 (s, 3H); **^{13}C NMR** (150 MHz, $CDCl_3$) δ 158.1, 146.6, 138.2, 112.8, 87.3, 83.2, 56.4.

5-iodo-2-methoxybenzaldehyde

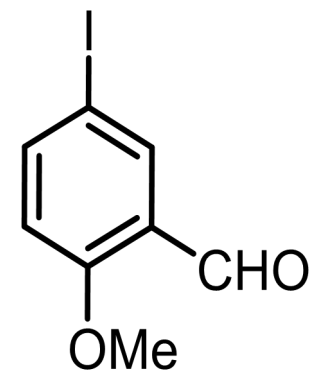

Compound **2**[15] was synthesized following the **General Procedure for Aryl iodides.** The reaction of the corresponding aryl DBT salt (94 mg, 0.200 mmol, 1.0 equiv.), KI (49.8 mg, 0.300 mmol, 1.5 equiv.) in CH_3CN (2.0 mL) delivered **2** (43.0 mg, 82%) as a white solid after purification via silica gel column chromatography with 10% ethyl acetate/hexanes (R_f = 0.5, 10% ethyl acetate/hexanes). **^{1}H NMR** (600 MHz, $CDCl_3$) δ 10.34 (s, 1H), 8.09 (s, 1H), 7.81 (d, *J* = 8.4Hz, 1 H), 6.78 (d, *J* = 9.0 Hz, 1H), 3.92 (s, 3H); **^{13}C NMR** (150 MHz, $CDCl_3$) δ 188.2, 161.4, 144.1, 137.1, 126.5, 114.1, 83.0, 55.8.

5-iodo-2-methoxybenzonitrile

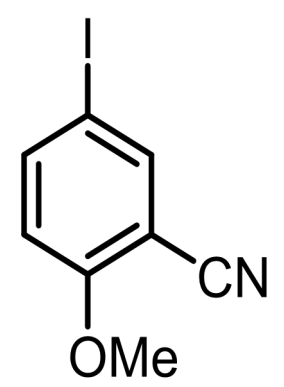

Compound **27**[19] was synthesized following the **General Procedure for Aryl iodides.** The reaction of the corresponding aryl DBT salt (93 mg, 0.200 mmol, 1.0 equiv.), KI (49.8 mg, 0.300 mmol, 1.5 equiv.) in CH_3CN (2.0 mL) delivered **27** (43.5 mg, 84%) as a white solid after purification via silica gel column chromatography with 10% ethyl acetate/hexanes (R_f = 0.5, 10% ethyl acetate/hexanes). **^{1}H NMR** (600 MHz, $CDCl_3$) δ 7.85-7.76 (m, 2H), 6.75 (d, *J* = 8.4Hz, 1H), 3.92 (s, 3H); **^{13}C NMR** (150 MHz, $CDCl_3$) δ 161.0, 143.0, 141.6, 114,8, 113.4, 104.2, 81.2, 56.2.

2-iodo-1,4-dimethylbenzene

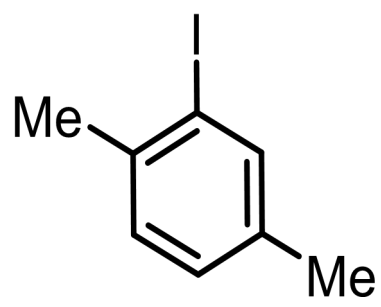

Compound **28**[5] was synthesized following the **General Procedure for Aryl iodides.** The reaction of the corresponding aryl DBT salt (88 mg, 0.200 mmol, 1.0 equiv.), KI (49.8 mg, 0.300 mmol, 1.5 equiv.) in CH_3CN (2.0 mL) delivered **28** (29.2 mg, 63%) as a white solid after purification via silica gel column chromatography with hexanes (R_f = 0.8, hexanes). **^{1}H NMR** (600 MHz, $CDCl_3$) δ 7.65 (s, 1H), 7.11 (d, *J* = 7.8 Hz, 1H), 7.04 (d, *J* = 7.8 Hz, 1H), 2.38 (s, 3H), 2.27 (s, 3H); **^{13}C NMR** (150 MHz, $CDCl_3$) δ 139.3, 138.1, 137.2, 129.3, 129.0, 101.0, 27.5, 20.3.

4-fluoro-2-iodo-1-methoxybenzene

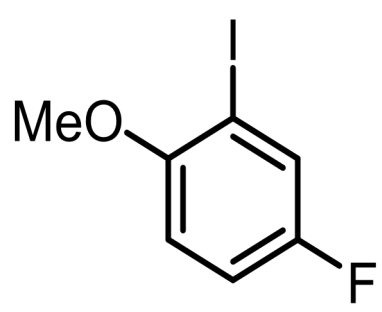

Compound **29**[3] was synthesized following the **General Procedure for Aryl iodides.** The reaction of the corresponding aryl DBT salt (92 mg, 0.200 mmol, 1.0 equiv.), KI (49.8 mg, 0.300 mmol, 1.5 equiv.) in CH_3CN (2.0 mL) delivered **29** (37.3 mg, 74%) as a white solid after purification via silica gel column chromatography with hexanes (R_f = 0.8, hexanes). **^{1}H NMR** (600 MHz, $CDCl_3$) δ 7.50 (d, *J* = 7.2 Hz, 1H), 7.03 (t, *J* = 8.4 Hz, 1H), 6.75 (q, *J* = 4.8 Hz, 1H), 3.85 (s, 3H); **^{13}C NMR** (150 MHz, $CDCl_3$) δ 157.5, 155.9, 154.8 (d, *J* = 3.0 Hz), 126.2, 126.1, 115.6, 115.5, 111.0, 110.9, 85.2 (d, *J* = 9.0 Hz), 56.9; **^{19}F NMR** (565 MHz, $CDCl_3$) δ -122.5.

2-iodo-1-methoxy-4-(trifluoromethyl)benzene

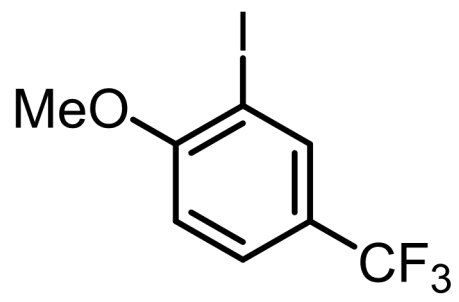

Compound **30**[8] was synthesized following the **General Procedure for Aryl iodides.** The reaction of the corresponding aryl DBT salt (102 mg, 0.200 mmol, 1.0 equiv.), KI (49.8 mg, 0.300 mmol, 1.5 equiv.) in CH_3CN (2.0 mL) delivered **30** (47.7 mg, 79%) as a white solid after purification via silica gel column chromatography with 2% ethyl acetate/hexanes (R_f = 0.5, 2% ethyl acetate/hexanes). **^{1}H NMR** (600 MHz, $CDCl_3$) δ

8.01 (s, 1H), 7.58 (d, *J* = 8.4 Hz, 1H), 6.86 (d, *J* = 8.4 Hz, 1H), 3.93 (s, 3H); **^{13}C NMR** (150 MHz, $CDCl_3$) δ 160.6, 136.6 (q, *J* = 3.0 Hz), 127.0 (q, *J* = 4.5 Hz), 124.8, 124.6, 124.4, 124.2 (d, *J* = 6.0 Hz), 122.35, 110.4, 56.6; **^{19}F NMR** (565 MHz, $CDCl_3$) δ -61.6.

3-iodo-4-methoxybenzaldehyde

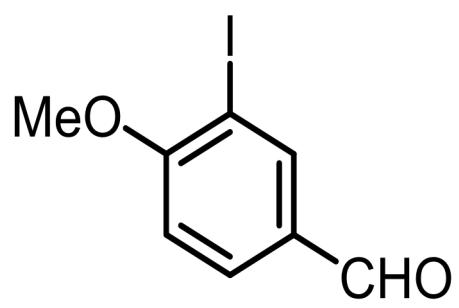

Compound **31**[4] was synthesized following the **General Procedure for Aryl iodides.** The reaction of the corresponding aryl DBT salt (94 mg, 0.200 mmol, 1.0 equiv.), KI (49.8 mg, 0.300 mmol, 1.5 equiv.) in CH_3CN (2.0 mL) delivered **31** (41.9 mg, 80%) as a white solid after purification via silica gel column chromatography with 10% ethyl acetate/hexanes (R_f = 0.5, 10% ethyl acetate/hexanes). **^{1}H NMR** (600 MHz, $CDCl_3$) δ 9.82 (s, 1H), 8.30 (s, 1H), 7.85 (d, *J* = 7.8 Hz, 1H), 6.92 (d, *J* = 8.4 Hz, 1H), 3.97 (s, 3H); **^{13}C NMR** (150 MHz, $CDCl_3$) δ 189.3, 162.7, 141.0, 132.0, 131.4, 110.5, 86.4, 56.7.

1-(3-iodo-4-methoxyphenyl)ethan-1-one

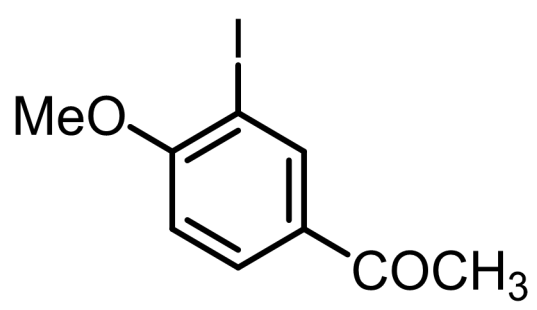

Compound **32**[4] was synthesized following the **General Procedure for Aryl iodides.** The reaction of the corresponding aryl DBT salt (96 mg, 0.200 mmol, 1.0 equiv.), KI (49.8 mg, 0.300 mmol, 1.5 equiv.) in CH_3CN (2.0 mL) delivered **32** (35.9 mg, 65%) as a white solid after purification via silica gel column chromatography with 10% ethyl acetate/hexanes (R_f = 0.5, 10% ethyl acetate/hexanes). **^{1}H NMR** (600 MHz, $CDCl_3$) δ 8.32 (d, *J* = 1.8 Hz, 1H), 7.88 (dd, *J* = 1.8, 8.4 Hz, 1H), 6.78 (d, *J* = 8.4 Hz, 1H), 3.88 (s, 3H), 2.48 (s, 3H); **^{13}C NMR** (150 MHz, $CDCl_3$) δ 195.5, 161.7, 140.1, 131.8, 130.5, 110.0, 85.8, 56.6, 26.3.

(3-iodo-4-methoxyphenyl)(phenyl)methanone

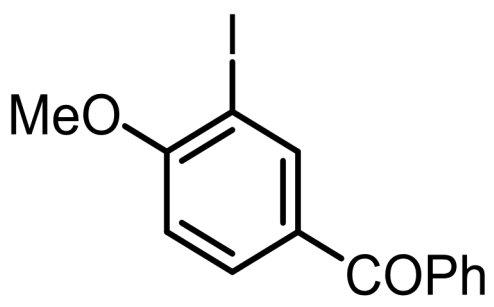

Compound **33**[6] was synthesized following the **General Procedure for Aryl iodides.** The reaction of the corresponding aryl DBT salt (0.109 g, 0.200 mmol, 1.0 equiv.), KI (49.8 mg, 0.300 mmol, 1.5 equiv.) in CH_3CN (2.0 mL) delivered **33** (53.4 mg, 79%) as a white solid after purification via silica gel column chromatography with 10% ethyl acetate/hexanes (R_f = 0.5, 10% ethyl acetate/hexanes). **^{1}H NMR** (600 MHz, $CDCl_3$) δ 8.29 (d, *J* = 1.8 Hz, 1H), 7.82 (dd, *J* = 2.4, 9.0 Hz, 1H), 7.74 (d, *J* = 7.2 Hz, 2H), 7.58 (t, *J* = 7.2 Hz, 1H), 7.48 (t, *J* = 7.8, 2H), 6.87 (d, *J* = 8.4 Hz, 1H), 3.96 (s, 3H); **^{13}C NMR** (150 MHz, $CDCl_3$) δ 194.1, 161.4, 141.6, 137.6, 132.5, 132.2, 131.8, 129.7, 128.3, 109.8, 85.6, 56.6.

3-iodo-4-methoxybenzonitrile

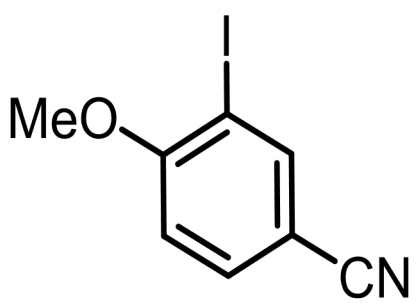

Compound **34**[3] was synthesized following the **General Procedure for Aryl iodides.** The reaction of the corresponding aryl DBT salt (93 mg, 0.200 mmol, 1.0 equiv.), KI (49.8 mg, 0.300 mmol, 1.5 equiv.) in CH_3CN (2.0 mL) delivered **34** (41.4 mg, 80%) as a white solid after purification via silica gel column chromatography with 10% ethyl acetate/hexanes (R_f = 0.5, 10% ethyl acetate/hexanes). **^{1}H NMR** (600 MHz, $CDCl_3$) δ 8.03 (d, *J* = 2.4 Hz, 1H), 7.62 (dd, *J* = 1.8, 8.4 Hz, 1H), 6.65 (d, *J* = 8.4 Hz, 1H), 3.94 (s, 3H); **^{13}C NMR** (150 MHz, $CDCl_3$) δ 161.5, 142.7, 134.0, 117.5, 110.7, 105.8, 86.0, 56.7.

methyl 3-iodo-4-methoxybenzoate

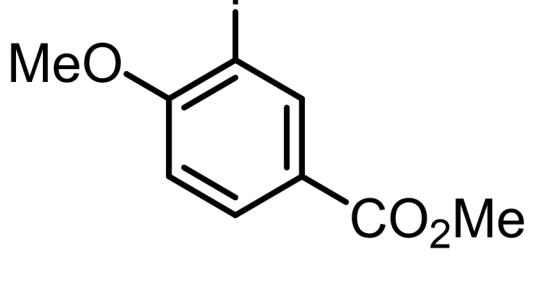

Compound **35**[5] was synthesized following the **General Procedure for Aryl iodides.** The reaction of the corresponding aryl DBT salt (100 mg, 0.200 mmol, 1.0 equiv.), KI (49.8 mg, 0.300 mmol, 1.5 equiv.) in CH_3CN (2.0 mL) delivered **35** (49.0 mg, 84%) as

a white solid after purification via silica gel column chromatography with 10% ethyl acetate/hexanes (R_f = 0.5, 10% ethyl acetate/hexanes). **^{1}H NMR** (600 MHz, $CDCl_3$) δ 8.46 (d, *J* = 2.4 Hz, 1H), 8.02 (dd, *J* = 2.4, 9.0 Hz, 1H), 6.83 (d, *J* = 9.0 Hz, 1H), 3.94 (s, 3H), 3.89 (s, 3H); **^{13}C NMR** (150 MHz, $CDCl_3$) δ 165.5, 161.6, 141.0, 131.6, 124.3, 109.9, 85.3, 56.6, 52.1.

2-iodo-1,3,5-trimethylbenzene

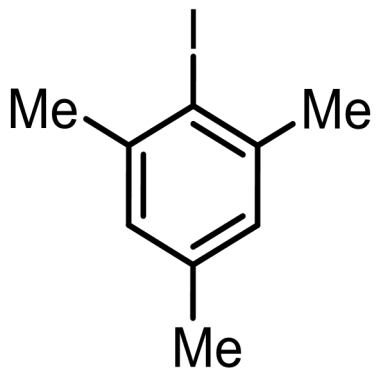

Compound **36**[9] was synthesized following the **General Procedure for Aryl iodides.** The reaction of the corresponding aryl DBT salt (90 mg, 0.200 mmol, 1.0 equiv.), KI (49.8 mg, 0.300 mmol, 1.5 equiv.) in CH_3CN (2.0 mL) delivered **36** (42.3 mg, 86%) as a white solid after purification via silica gel column chromatography with hexanes (R_f = 0.8, hexanes). **^{1}H NMR** (600 MHz, $CDCl_3$) δ 6.90 (s, 2H), 2.44 (s, 6H), 2.25 (s, 3H); **^{13}C NMR** (150 MHz, $CDCl_3$) δ 141.7, 137.3, 127.9, 104.2, 29.5, 20.6.

1-iodo-4-methoxy-2-methyl-5-nitrobenzene

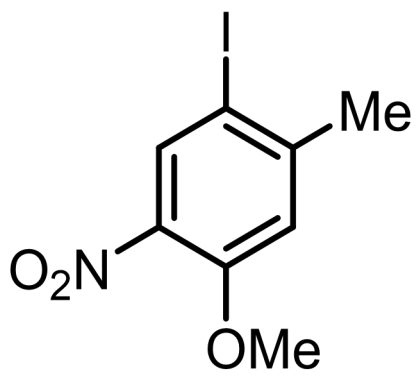

Compound **37** was synthesized following the **General Procedure for Aryl iodides.** The reaction of the corresponding aryl DBT salt (100 mg, 0.200 mmol, 1.0 equiv.), KI (49.8 mg, 0.300 mmol, 1.5 equiv.) in CH_3CN (2.0 mL) delivered **37** (42.2 mg, 72%) as a white solid after purification via silica gel column chromatography with 10% ethyl acetate/hexanes (R_f = 0.5, 10% ethyl acetate/hexanes). **^{1}H NMR** (600 MHz, $CDCl_3$) δ 8.27 (s, 1H), 6.97 (s, 1H), 3.94 (s, 3H), 2.48 (s, 3H); **^{13}C NMR** (150 MHz, $CDCl_3$) δ 153.1, 148.9, 137.7, 135.4, 114.6, 87.5, 56.6, 28.7; **HRMS (ESI$^+$)** Calcd for $C_8H_8INO_3$ $[M+H]^+$ 293.9622, found 293.9622.

2-iodo-9H-xanthen-9-one

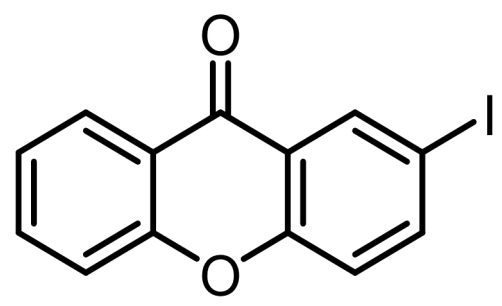

Compound **38**[7] was synthesized following the **General Procedure for Aryl iodides.** The reaction of the corresponding aryl DBT salt (0.106 g, 0.200 mmol, 1.0 equiv.), KI (49.8 mg, 0.300 mmol, 1.5 equiv.) in CH_3CN (2.0 mL) delivered **38** (52.2 mg, 81%) as a white solid after purification via silica gel column chromatography with 5% ethyl acetate/hexanes (R_f = 0.5, 5% ethyl acetate/hexanes). **^{1}H NMR** (600 MHz, $CDCl_3$) δ 8.615 (s, 1H), 8.23 (d, *J* = 7.8 Hz, 1H), 7.94 (d, *J* = 8.4 Hz, 1H), 7.72 (t, *J* = 7.8 Hz, 1H), 7.46 (d, *J* = 8.4 Hz, 1H), 7.38 (t, *J* = 7.2 Hz, 1H), 7.24 (d, *J* = 8.4 Hz, 1H); **^{13}C NMR** (150 MHz, $CDCl_3$) δ 175.8, 156.0, 155.6, 143.2, 135.5, 135.2, 126.8, 124.3, 123.5, 121.6, 120.2, 118.1, 87.2.

Ethyl 2-(4-chloro-2-iodophenoxy)-2-methylpropanoate

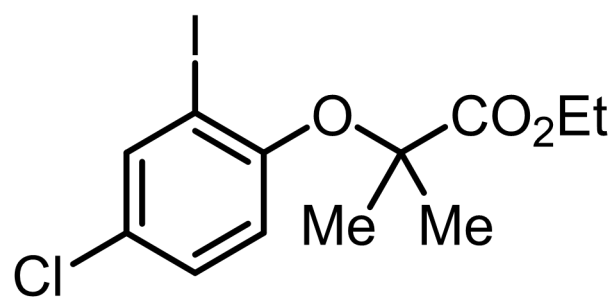

Compound **39**[3] was synthesized following the **General Procedure for Aryl iodides.** The reaction of the corresponding aryl DBT salt (115 mg, 0.200 mmol, 1.0 equiv.), KI (49.8 mg, 0.300 mmol, 1.5 equiv.) in CH_3CN (2.0 mL) delivered **39** (31.6 mg, 43%) as a white solid after purification via silica gel column chromatography with 10% ethyl acetate/hexanes (R_f = 0.5, 10% ethyl acetate/hexanes). **^{1}H NMR** (600 MHz, $CDCl_3$) δ 7.75 (s, 1H), 7.17 (d, *J* = 9.0 Hz, 1H), 6.69 (d, *J* = 8.4 Hz, 1H), 4.24 (q, *J* = 6.6 Hz, 2H), 1.63 (s, 6H), 1.26 (t, 3H); **^{13}C NMR** (150 MHz, $CDCl_3$) δ 173.8, 154.0, 138.7, 128.8, 127.9, 118.0, 91.4, 81.2, 61.4, 25.3, 14.1.

Methyl 2-(4-chloro-2-iodophenoxy)acetate

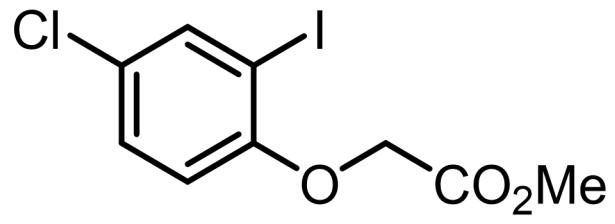

Compound **40**[20] was synthesized following the **General Procedure for Aryl iodides**. The reaction of the corresponding aryl DBT salt (106 mg, 0.200 mmol, 1.0 equiv.), KI 49.8 (49.8 mg, 0.300 mmol, 1.5 equiv.) in CH_3CN (2.0 mL) delivered **40** (52.1 mg, 80%)

as a white solid after purification via silica gel column chromatography with 10% ethyl acetate/hexanes (R_f = 0.5, 10% ethyl acetate/hexanes). **^{1}H NMR** (600 MHz, $CDCl_3$) δ 7.73 (s, 1H), 7.38 (s, 4H), 7.32 (d, *J* = 7.2 Hz, 1H), 7.13 (d, *J* = 7.2 Hz, 1H), 7.01 (s, 4H), 5.37 (d, *J* = 8.4 Hz, 2H); **^{13}C NMR** (150 MHz, $CDCl_3$) δ 147.8, 145.2, 144.7, 144.4, 134.0, 132.5, 125.4, 125.4, 123.8, 123.7, 89.9, 53.6, 53.5.

(9r,10r)-2-iodo-9,10-dihydro-9,10-[1,2]benzenoanthracene

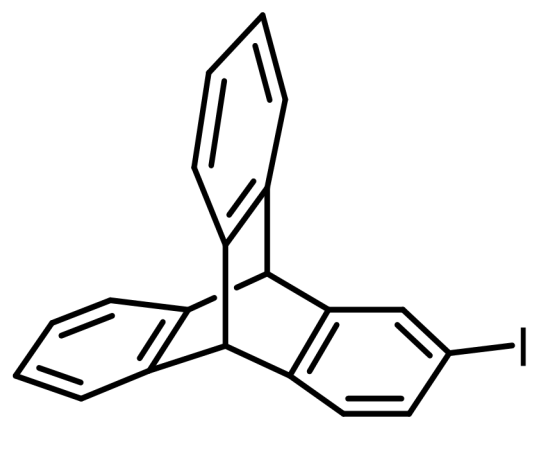

Compound **41** was synthesized following the **General Procedure for Aryl iodides**. The reaction of the corresponding aryl DBT salt (0.117 g, 0.200 mmol, 1.0 equiv.), KI 49.8 (49.8 mg, 0.300 mmol, 1.5 equiv.) in CH_3CN (2.0 mL) delivered **41** (47.9 mg, 63%) as a white solid after purification via silica gel column chromatography with hexanes (R_f = 0.5, hexanes). **^{1}H NMR** (600 MHz, $CDCl_3$) δ 7.73 (s, 1H), 7.38 (s, 4H), 7.32 (d, *J* = 7.2 Hz, 1H), 7.13 (d, *J* = 7.2 Hz, 1H), 7.01 (s, 4H), 5.37 (d, *J* = 8.4 Hz, 2H); **^{13}C NMR** (150 MHz, $CDCl_3$) δ 147.8, 145.2, 144.7, 144.4, 134.0, 132.5, 125.4, 125.4, 123.8, 123.7, 89.9, 53.6, 53.5; **HRMS (ESI$^+$)** Calcd for $C_{20}H_{13}I$ $[M+H]^+$ 381.0135, found 381.0135.

1-(2,6-dichlorophenyl)-5-iodoindolin-2-one

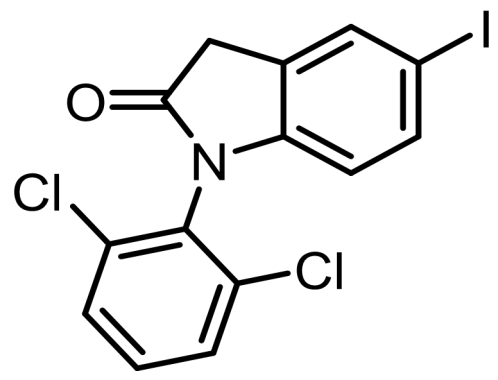

Compound **42**[16] was synthesized following the **General Procedure for Aryl iodides.** The reaction of the corresponding aryl DBT salt (122 mg, 0.200 mmol, 1.0 equiv.), KI (49.8 mg, 0.300 mmol, 1.5 equiv.) in CH_3CN (2.0 mL) delivered **42** (45.1 mg, 56%) as a white solid after purification via silica gel column chromatography with 10% ethyl acetate/hexanes (R_f = 0.5, 10% ethyl acetate/hexanes). **^{1}H NMR** (600 MHz, $CDCl_3$) δ 7.65 (s, 1H), 7.55-7.48 (c, 3H), 7.39 (t, *J* = 7.8 Hz, 1H), 6.19 (d, *J* = 7.8 Hz, 1H), 3.76

(s, 2H); **^{13}C NMR** (150 MHz, $CDCl_3$) δ 172.6, 143.0, 136.8, 135.4, 133.6, 131.0, 129.9, 129.1, 126.6, 111.1, 85.6, 35.2.

3-chloro-9-iodo-6-methyldibenzo[c,f][1,2]thiazepin-11(6H)-one 5,5-dioxide

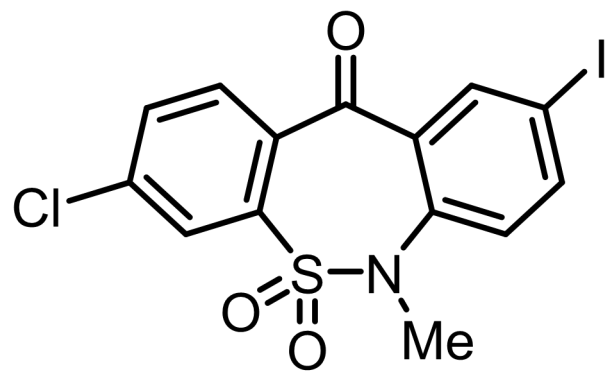

Compound **43** was synthesized following the **General Procedure for Aryl iodides.** The reaction of the corresponding aryl DBT salt (0.128 g, 0.200 mmol, 1.0 equiv.), KI (49.8 mg, 0.300 mmol, 1.5 equiv.) in CH_3CN (2.0 mL) delivered **43** (60.6 mg, 70%) as a white solid after purification via silica gel column chromatography with 10% ethyl acetate/hexanes (R_f = 0.5, 10% ethyl acetate/hexanes). **^{1}H NMR** (600 MHz, $CDCl_3$) δ 8.61 (s, 1H), 7.96-7.87 (m, 3H), 7.69 (d, *J* = 8.4 Hz, 1H), 7.08 (d, *J* = 8.4 Hz, 1H), 3.34 (s, 3H); **^{13}C NMR** (150 MHz, $CDCl_3$) δ 188.2, 143.4, 141.0, 140.6, 139.0, 138.3, 133.9, 133.5, 133.3, 132.0, 126.0, 125.4, 90.2, 38.8; **HRMS (ESI$^+$)** Calcd for $C_{14}H_9ClINO_3S$ $[M+H]^+$ 433.9109, found 433.9109.

Isopropyl 2-(4-(4-chlorobenzoyl)-2-iodophenoxy)-2-methylpropanoate

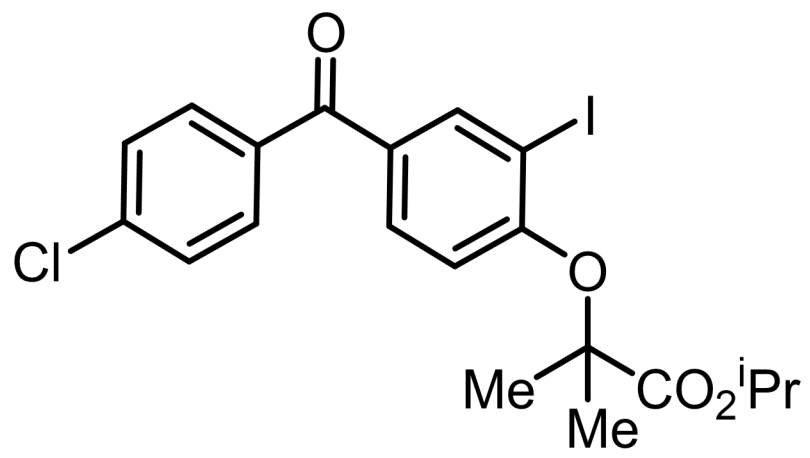

Compound **44** was synthesized following the **General Procedure for Aryl iodides.** The reaction of the corresponding aryl DBT salt (138 mg, 0.200 mmol, 1.0 equiv.), KI (49.8 mg, 0.300 mmol, 1.5 equiv.) in CH_3CN (2.0 mL) delivered **44** (49.6 mg, 51%) as a white solid after purification via silica gel column chromatography with 10% ethyl acetate/hexanes (R_f = 0.5, 10% ethyl acetate/hexanes). **^{1}H NMR** (600 MHz, $CDCl_3$) δ 8.25 (d, *J* = 8.25 Hz, 1H), 7.69 (d, *J* = 8.4 Hz, 2H), 7.66 (dd, *J* = 1.8, 8.4 Hz, 1H), 6.72 (d, *J* = 8.4 Hz, 2H), 5.22-4.97 (m, 1H), 1.71 (s, 6H), 1.21 (d, *J* = 6.6 Hz, 6H); **^{13}C NMR** (150 MHz, $CDCl_3$) δ 192.9, 172.7, 158.7, 141.7, 138.8, 135.8, 132.0, 131.2, 131.1,

128.7, 115.0, 89.5, 81.3, 69.5, 25.4, 21.5; **HRMS (ESI$^+$)** Calcd for $C_{20}H_{20}ClIO_4$ $[M+H]^+$ 487.0168, found 487.0165.

Methyl 5-(4-iodo-2,5-dimethylphenoxy)-2,2-dimethylpentanoate

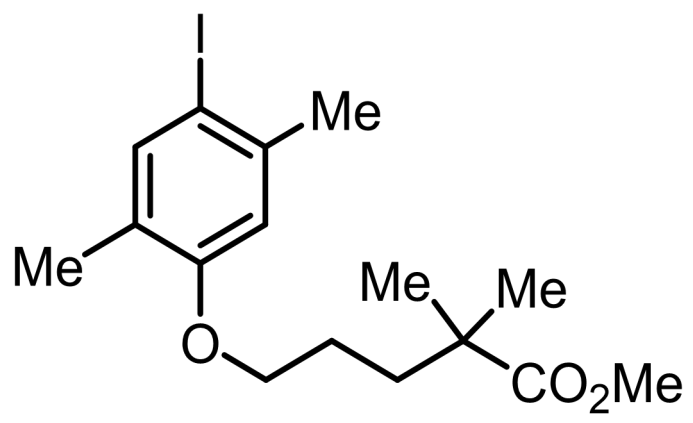

Compound **45**[3] was synthesized following the **General Procedure for Aryl iodides.** The reaction of the corresponding aryl DBT salt (119 mg, 0.200 mmol, 1.0 equiv.), KI (49.8 mg, 0.300 mmol, 1.5 equiv.) in CH_3CN (2.0 mL) delivered **45** (39.0 mg, 50 %) as a white solid after purification via silica gel column chromatography with 10% ethyl acetate/hexanes (R_f = 0.5, 10% ethyl acetate/hexanes). **^{1}H NMR** (600 MHz, $CDCl_3$) δ 7.51 (s, 1H), 6.67 (s, 1H), 3.89 (t, *J* = 5.4 Hz, 2H), 3.66 (s, 3H), 2.37 (s, 3H), 2.13 (s, 3 H), 1.74-1.66 (m, 4H), 1.22 (s, 6H); **^{13}C NMR** (150 MHz, $CDCl_3$) δ 178.2, 157.3, 139.9, 139.3, 126.6, 112.7, 89.0, 68.0, 51.7, 42.0, 37.0, 28.0, 25.2, 25.0, 15.2.

Methyl-2-(1-(4-chlorobenzoyl)-6-iodo-5-methoxy-2-methyl-1H-indol-3-yl)acetate

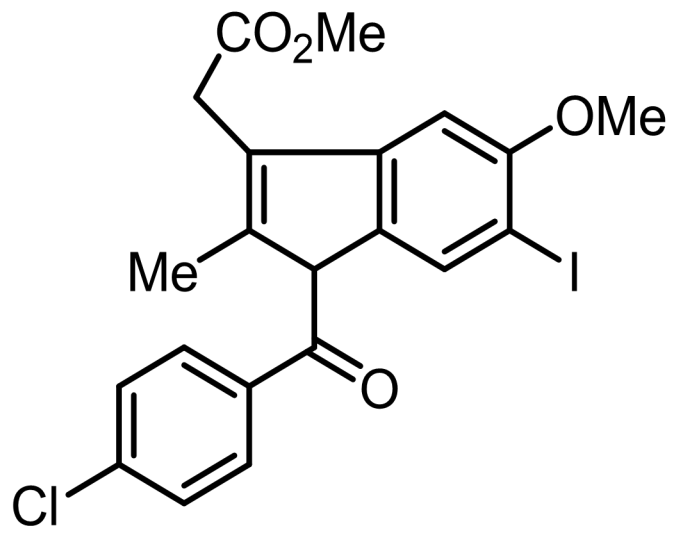

Compound **46**[3] was synthesized following the **General Procedure for Aryl iodides.** The reaction of the corresponding aryl DBT salt (141 mg, 0.200 mmol, 1.0 equiv.), KI (49.8 mg, 0.300 mmol, 1.5 equiv.) in CH_3CN (2.0 mL) delivered **46** (50.7 mg, 51%) as a white solid after purification via silica gel column chromatography with 10% ethyl acetate/hexanes (R_f = 0.5, 10% ethyl acetate/hexanes). **^{1}H NMR** (600 MHz, $CDCl_3$) δ 7.65 (d, *J* = 7.8 Hz, 2H), 7.58 (s, 1H), 7.49 (d, *J* = 7.8 Hz, 2H), 6.91 (s, 1H), 3.92 (s, 3H), 3.70 (s, 3H), 3.65 (s, 2H), 2.28 (m, 3H); **^{13}C NMR** (150 MHz, $CDCl_3$) δ 171.1, 168.0, 154.2, 139.6, 135.8, 133.4, 131.8, 131.2, 130.7, 129.2, 124.7, 112.3, 99.4, 81.1, 56.7, 52.2, 30.1, 13.5.

2-iododibenzo[*b,d*]thiophene

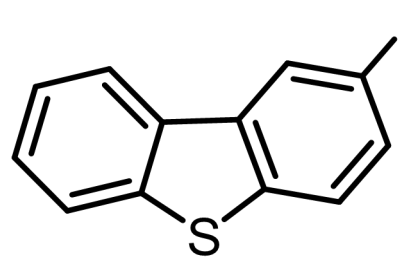

Compound **47**[26] was synthesized following the **General Procedure for Aryl iodides.** The reaction of the corresponding aryl DBT salt (103.2 mg, 0.200 mmol, 1.0 equiv.), KI (49.8 mg, 0.300 mmol, 1.5 equiv.) in CH_3CN (2.0 mL) delivered **47** (43.3 mg, 70%) as a white solid after purification via silica gel column chromatography with 10% ethyl acetate/hexanes (R_f = 0.5, 10% ethyl acetate/hexanes). **^{1}H NMR** (600 MHz, $CDCl_3$) δ 8.48f (d, *J* = 1.2 Hz, 1H), 8.12 -8.09 (m, 1H), 7.86 -7.83 (m, 1H), 7.72 (dd, *J* = 8.4 Hz, *J* = 1.8 Hz, 1H), 7.60 (d, *J* = 8.4 Hz, 1H), 7.50 -7.45 (m, 2H).

2.7. References

1. Aukland, M. H.; Šiaučiulis, M.; West, A.; Perry, G. J. P.; Procter, D. J. Metal-free photoredox-catalysed formal C–H/C–H coupling of arenes enabled by interrupted Pummerer activation. *Nat. Catal*. **2020**, *3*, 163.

2. Niu, L.; Zhang, H.; Yang, H.; Fu, H. Metal-free Iodination of Arylboronic Acids and the Synthesis of Biaryl Derivatives. *Synlett.* **2014**, *25*, 995. DOI: 10.1055/s-0033-1340871.

3.Yan, L. Y.; Gao, A.; Ma, C.; Zhang, Q.; Fu, M. C. Photoinduced Halogen Anion-Mediated Arene C–H Functionalization through Arylsulfonium Salts via Electron Donor-Acceptor Complexes. *Sci. China Chem*. **2025**, *68*. DOI: 10.1007/s11426-025-2682-y.

4. Leboeuf, D.; Ciesielski, J.; Frontier, A. J. Gold(I)-Catalyzed Iodination of Arenes. *Synlett*. **2014**, *25*, 399. DOI: 10.1055/s-0033-1340321.

5. Zhou, C. Y.; Li, J.; Peddibhotla, S.; Romo, D. Mild Arming and Derivatization of Natural Products via an $In(OTf)_3$-Catalyzed Arene Iodination. *Org. Lett*. **2010**, *12*, 2104. DOI: 10.1021/ol100587j.

6. Tang, J.; Zhao, S.; Wei, Y.; Quan, Z.; Huo, C. CBr_4 Promoted Intramolecular Aerobic Oxidative Dehydrogenative Arylation of Aldehydes: Application in the Synthesis of

Xanthones and Fluorenones. *Org. Biomol. Chem.* **2017**, *15*, 1589. DOI: 10.1039/c7ob00080d.

7. Tuerkmen, Y. E.; Rawal, V. H. Exploring the Potential of Diarylacetylenediols as Hydrogen Bonding Catalysts. *J. Org. Chem.* **2013**, *78*, 8340. DOI: 10.1021/jo400926n.

8. Hamamoto, H.; Hattori, S.; Takemaru, K.; Miki, Y. Hypervalent Iodine (III)-LiX Combination in Fluoroalcohol Solvent for Aromatic Halogenation of Electron-Rich Arenecarboxylic Acids. *Synlett.* **2011**, *11*, 1563. DOI: 10.1055/s-0030-1260791.

9. Mukhopadhyay, S.; Batra, S. Direct Transformation of Arylamines to Aryl Halides via Sodium Nitrite and N-Halosuccinimide. *Chem. Eur. J.* **2018**, *24*, 14622. DOI: 10.1002/chem.201803347.

10. Linshoeft, J.; Baum, E. J.; Hussain, A.; Gates, P. J.; Näther, C.; Staubitz, A. Highly Tin-Selective Stille Coupling: Synthesis of a Polymer Containing a Stannole in the Main Chain. *Angew. Chem. Int. Ed.* **2014**, 53, 12916. DOI: 10.1002/anie.201407377.

11. Huang, J.; Xue, M.; Shan, Z.; Zhang, S.; Lin, T.; Li, Y. Cu (II)-crosslinked carboxymethylcellulose entrapped in-situ generated Cu_2O nanoparticles composite beads: a highly efficient catalyst for ipso-iodination of arylboronic acids. *Res. Chem. Intermed.* **2025**, *51*, 6087. DOI: 10.1007/s11164-025-05722-z.

12. Fu, F.; Gurung, L.; Czaun, M.; Mathew, T.; Prakash, G. K. S. ipso-Bromination/Iodination of Arylboronic Acids: Poly (4-vinylpyridine)-Br_2/I_2 Complexes as Safe and Efficient Reagents. *Tetrahedron Lett.* **2019**, 60, 151020. DOI: 10.1016/j.tetlet.2019.151020.

13. Yang, H.; Li, Y.; Jiang, M.; Wang, J.; Fu, H. General Copper-Catalyzed Transformations of Functional Groups from Arylboronic Acids in Water. *Chem. Eur. J.* **2011**, 17, 5652. DOI: 10.1002/chem.201003711.

14. Yukawa, Y.; Tsuno, Y.; Sawada, M. *Bull. Chem. Soc. Jpn.* **1972**, 45, 1198. DOI: 10.1246/bcsj.45.1198.

15. Leboeuf, D.; Ciesielski, J.; Frontier, A. J. Gold(I)-Catalyzed Iodination of Arenes. *Synlett.* **2014**, *25*, 399. DOI: 10.1055/s-0033-1340321.

16. Wang, L.; Zhang, M.; Jin, Y.-Y.; Lu, Q.; Fang, Q. Three 1-phenylindolin-2-one derivatives displaying different molecular dipole moments and different

crystallographic symmetries. *Acta Crystallogr. Sect. C: Struct. Chem.* **2015**, *71*, 69. DOI: 10.1107/S2053229614026874.
17. Prebil, R.; Stovber, S. Aerobic oxidative O-iodination of carbonyl compounds using molecular iodine activated by a nitrate-based catalytic system. *Tetrahedron Lett.* **2014**, 55, 5643. DOI: 10.1016/j.tetlet.2014.08.055.
18. Gorecka, J.; Leroux, F.; Schlosser, M. Deprotonation-Triggered Heavy-Halogen Migrations as a Key to the Structural Elaboration of 2,2-Difluoro-1,3-benzodioxole. *Eur. J. Org. Chem.* **2004**, *1*, 64. DOI: 10.1002/ejoc.200300553.
19. Matsuoka, J.; Yano, Y.; Hirose, Y.; Mashiba, K.; Sawada, N.; Nakamura, A.; Maegawa, T. Elemental Sulfur-Mediated Aromatic Halogenation. *J. Org. Chem.* **2024**, *89*, 770. DOI: 10.1021/acs.joc.3c02259.
20. Mirk, D.; Kataeva, O.; Froehlich, R.; Waldvogel, S. R. The First Direct Chlorination of Iodoarenes - A Side-chain Directed Process. *Synthesis* **2003**, *15*, 2410. DOI: 10.1055/s-2003-42412.
21. Wu, J.; Wang, Z.; Chen, X.-Y.; Wu, Y.; Wang, D.; Peng, Q.; Wang, P. Para-selective borylation of monosubstituted benzenes using a transient mediator. *Sci. China Chem.* **2020**, *63*, 336. DOI: 10.1007/s11426-019-9652-x.
22. Zhang, L.; Si, X.; Rominger, F.; Hashmi, A. S. K. Visible-Light-Induced Radical Carbo-Cyclization/gem-Diborylation through Triplet Energy Transfer between a Gold Catalyst and Aryl Iodides. *J. Am. Chem. Soc.* **2020**, *142*, 10485. DOI: 10.1021/jacs.oc03197.
23. Kong, Q.; Jiang, C.; Lyu, B.; Li, Z.; Jiao, N.; Song, S. Denitrative Iodination of Nitroarenes with Hydroiodic Acid. *Chin. Chem. Lett.* **2025**, *36*, 111444. DOI: 10.1016/j.cclet.2025.111444.
24. Hajipour, A. R.; Arbabian, M.; Ruohon, A. E. Tetramethylammonium dichloroiodate: An efficient and environmentally friendly iodination reagent for iodination of aromatic compounds under mild and solvent-free conditions. *J. Org. Chem.* **2002**, *67*, 8622. DOI: 10.1021/jo0264628.
25. Weber, V.; Arnaud, L.; Dukic-Stefanovic, S.; Wenzel, B.; Roux, V.; Chezel, J.-M.; Lai, T.-H.; Deuther-Conrad, W.; Maisonial-Besset, A. Novel Radioiodinated and

Radiofluorinated Analogues of FT-2102 for SPECT or PET Imaging of mIDH1 Mutant Tumours. *Molecules* **2022**, *27*, 3766. DOI: 10.3390/molecules27123766.
26. Padniuk, I.; Stoica, O.; Zuzak, R.; Blieck, R.; Krawiec, M.; Godlewski, S.; Echavarren, A. M. On surface synthesis of an eleven-ringsulfur-doped nonacene. *Chem. Commun*., **2024**, *60*, 858. DOI: 10.1039/d3cc05486a.
27. Lu, Y.; Liu, Q.; Wang, Z. X.; Chen, X. Y. Alkynyl Sulfonium Salts Can Be Employed as Chalcogen-Bonding Catalysts and Generate Alkynyl Radicals under Blue-Light Irradiation. *Angew. Chem. Int. Ed*. **2022**, *61*, e202116071.

3. ^1H, ^{13}C and ^{19}F spectra of All Products

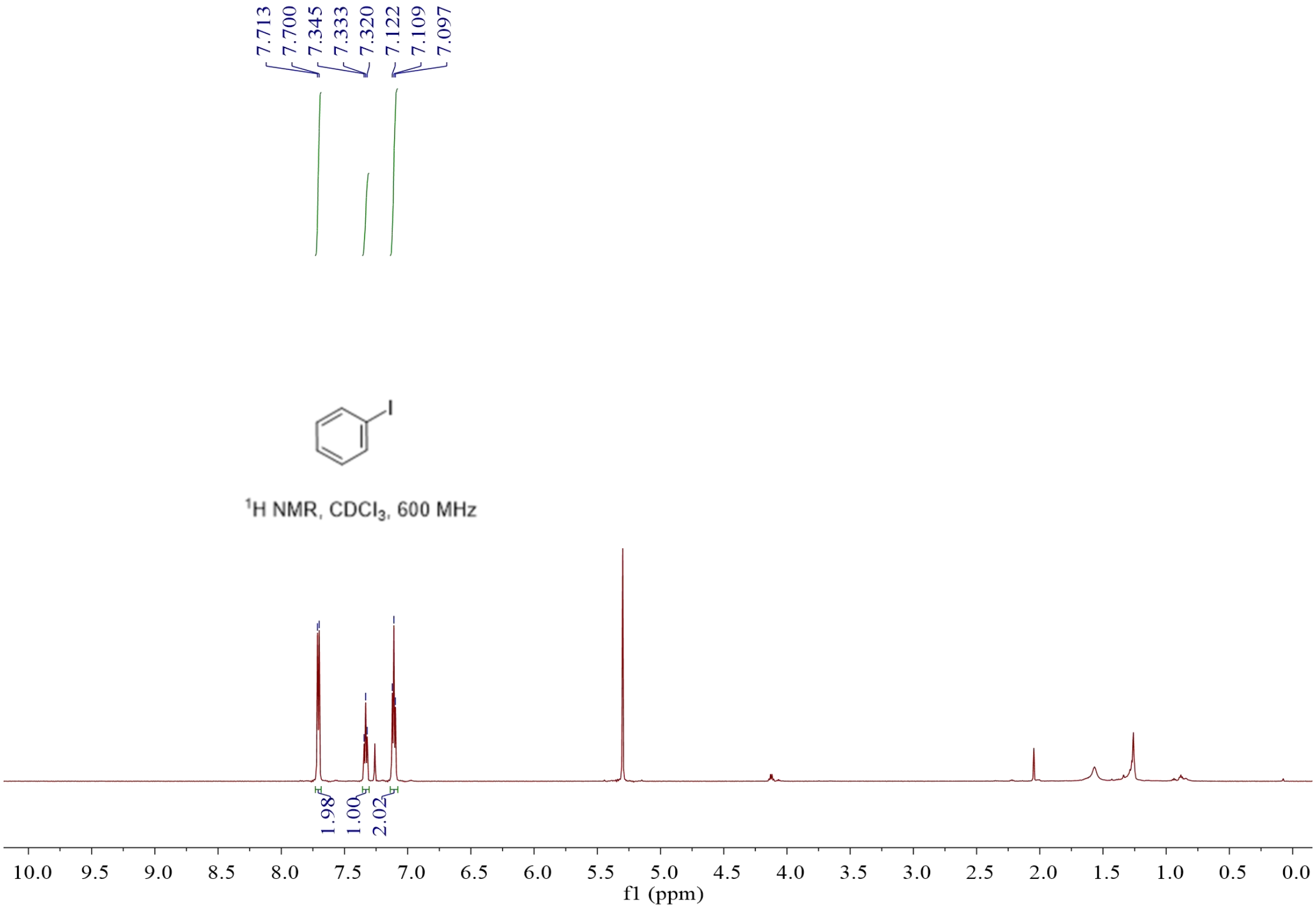

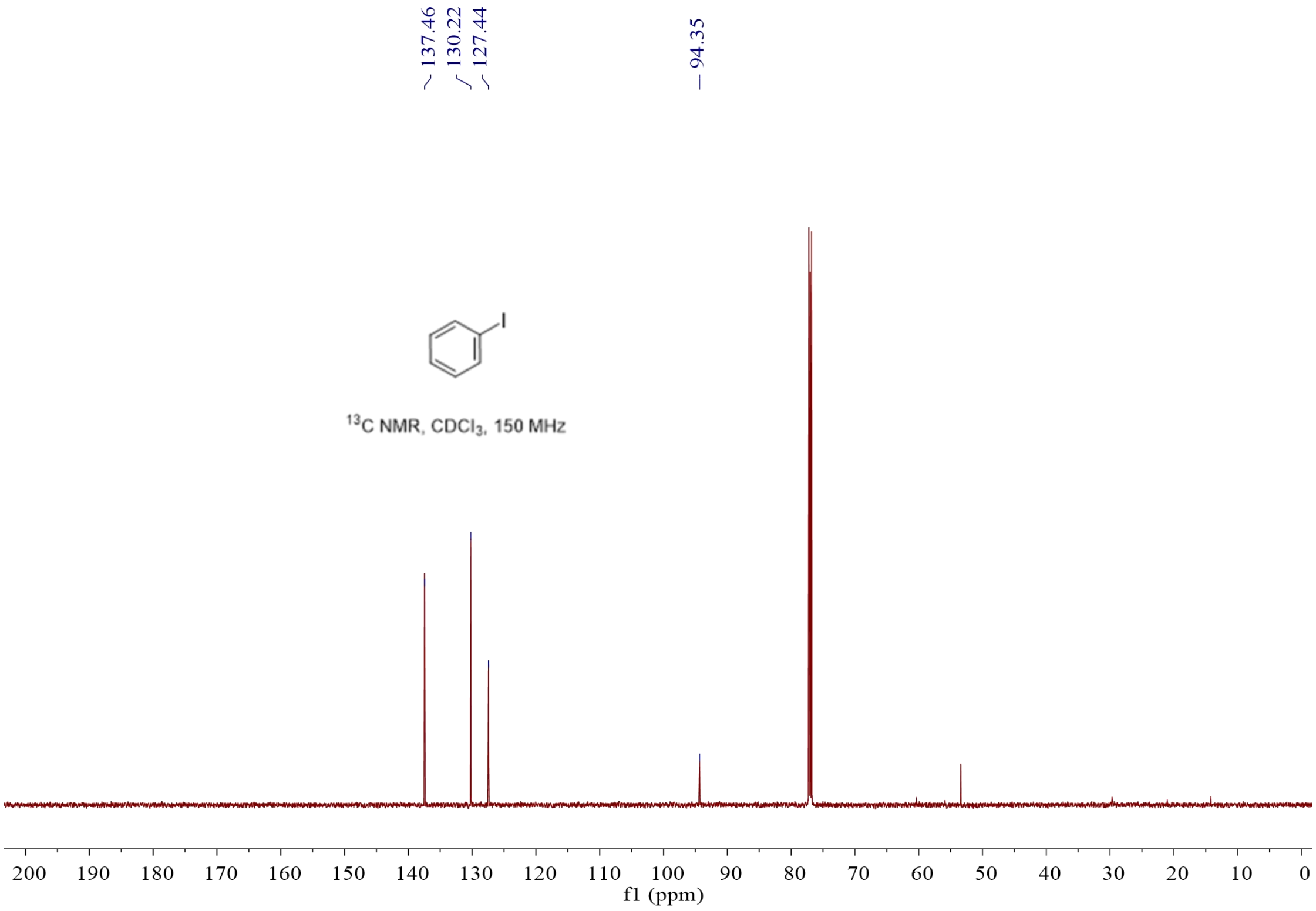

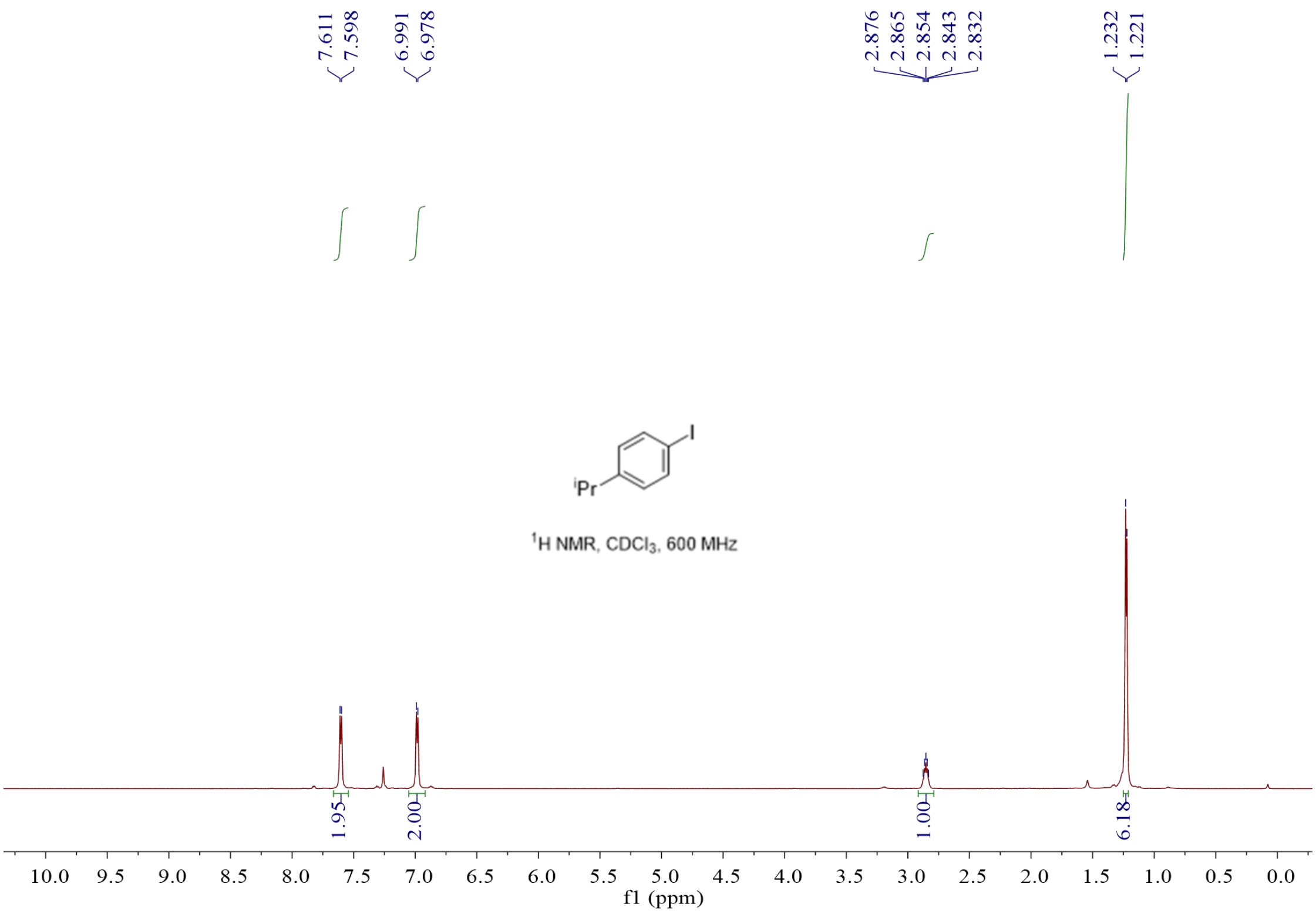

7.611
7.598
6.991
6.978
2.876
2.865
2.854
2.843
2.832
1.232
1.221
iPr
I
1H NMR, CDCl3, 600 MHz
1.95
2.00
1.00
6.18
10.0 9.5 9.0 8.5 8.0 7.5 7.0 6.5 6.0 5.5 5.0 4.5 4.0 3.5 3.0 2.5 2.0 1.5 1.0 0.5 0.0
f1 (ppm)

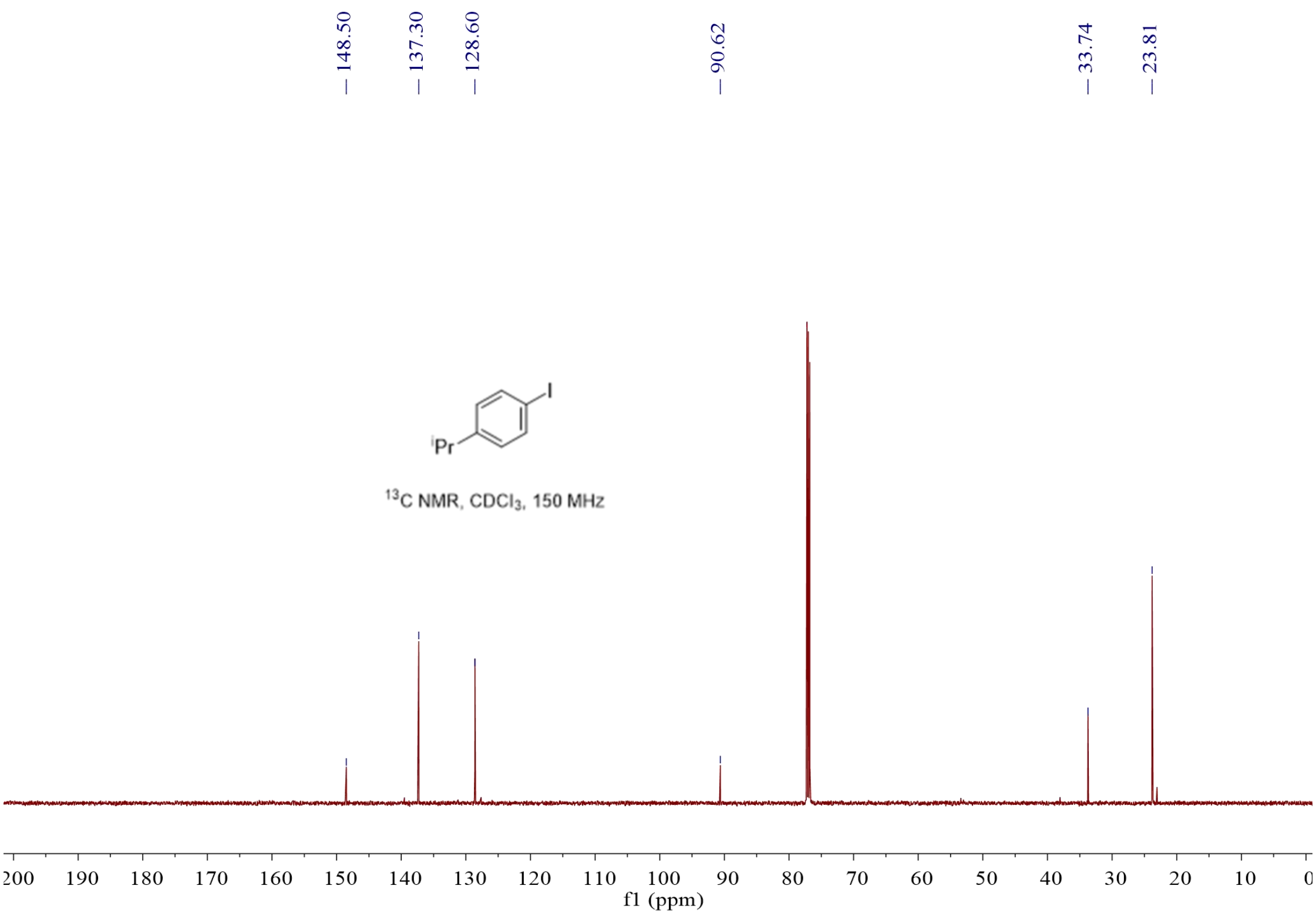

148.50
137.30
128.60
90.62
33.74
23.81
iPr
I
13C NMR, CDCl3, 150 MHz
200 190 180 170 160 150 140 130 120 110 100 90 80 70 60 50 40 30 20 10 0
f1 (ppm)

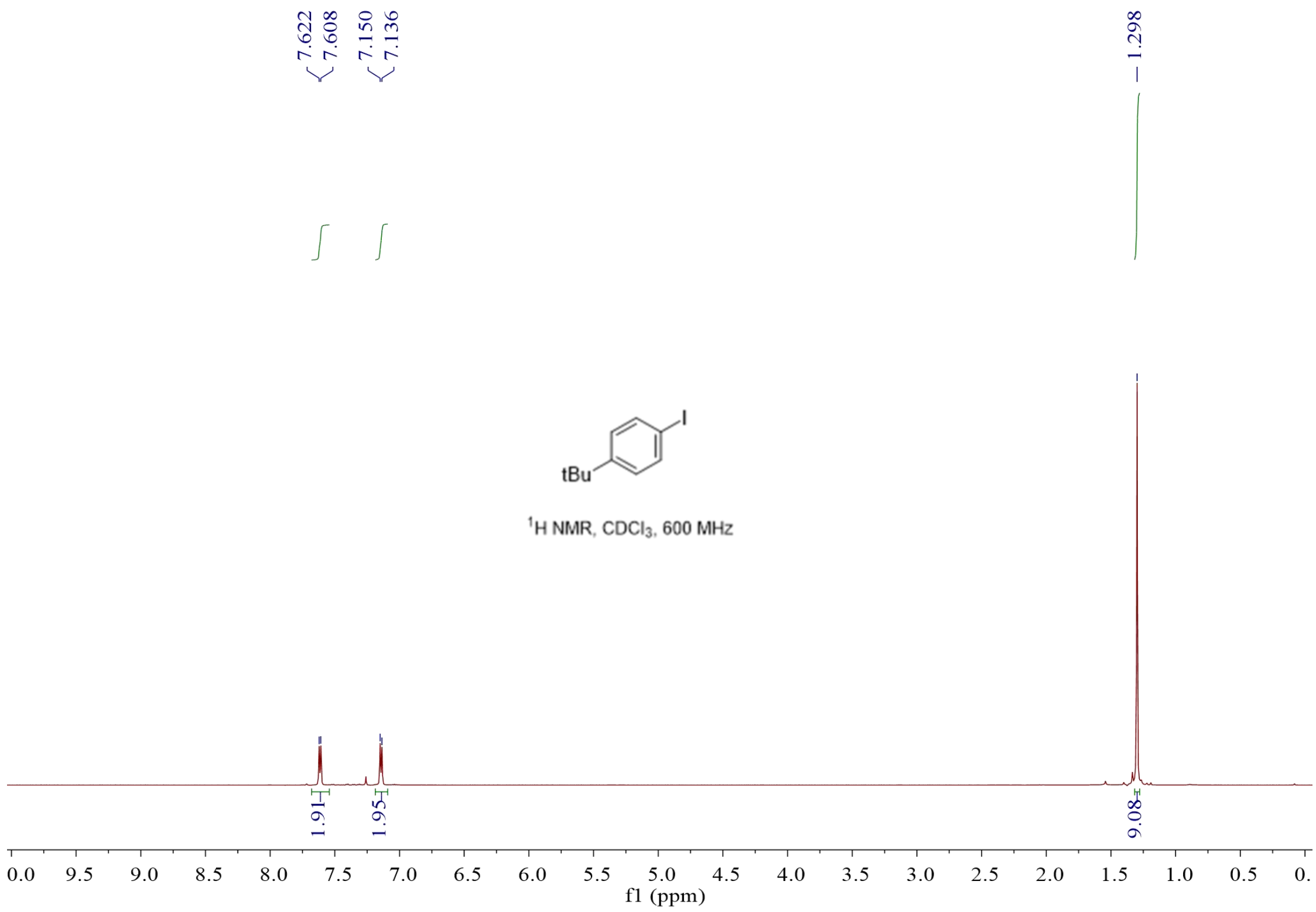
7.622
7.608
7.150
7.136
1.298
tBu
I
^{1}H NMR, CDCl$_3$, 600 MHz
1.91
1.95
9.08
0.0 9.5 9.0 8.5 8.0 7.5 7.0 6.5 6.0 5.5 5.0 4.5 4.0 3.5 3.0 2.5 2.0 1.5 1.0 0.5 0.
f1 (ppm)

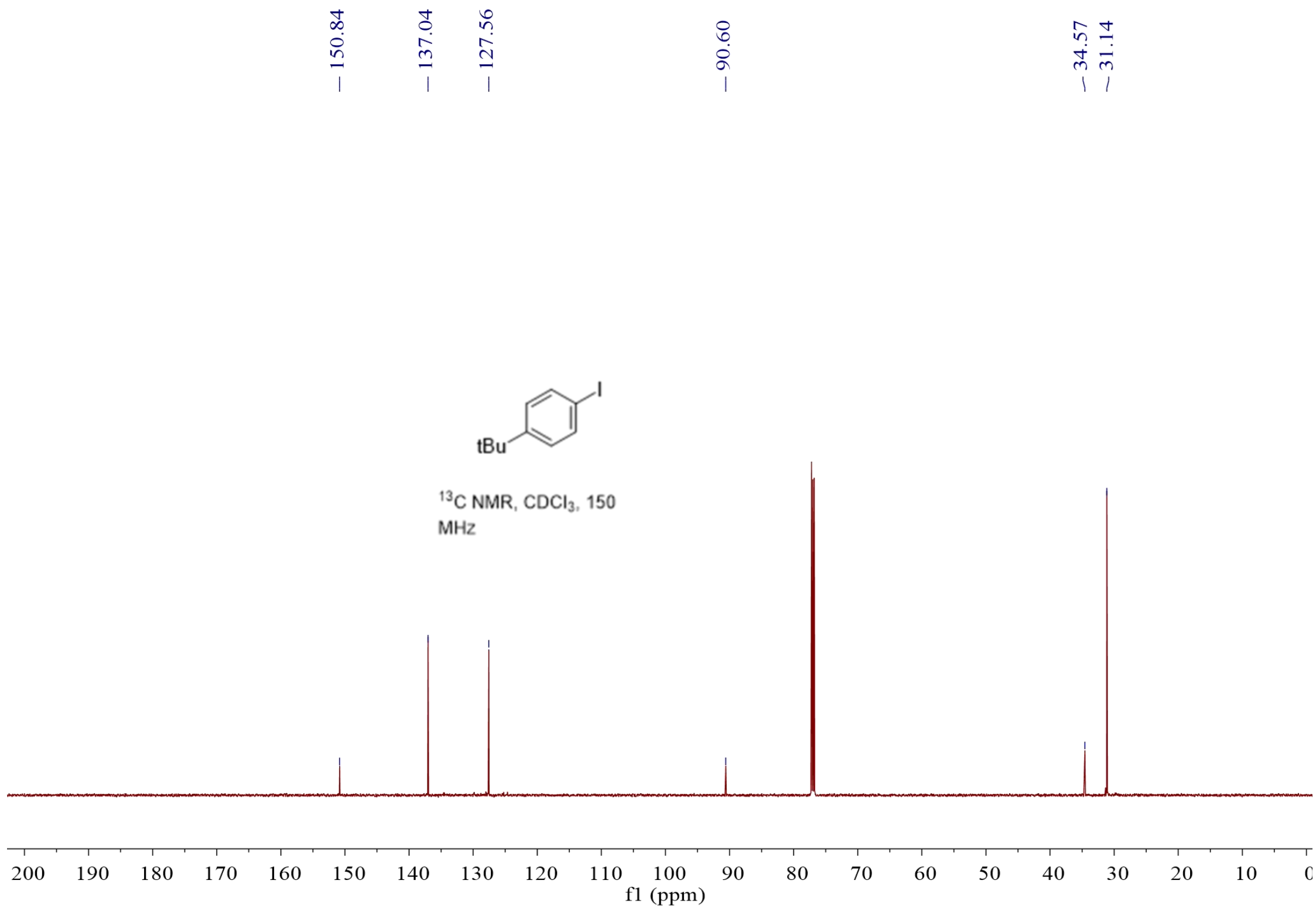
150.84
137.04
127.56
90.60
34.57
31.14
tBu
I
^{13}C NMR, CDCl$_3$, 150 MHz
200 190 180 170 160 150 140 130 120 110 100 90 80 70 60 50 40 30 20 10 0
f1 (ppm)

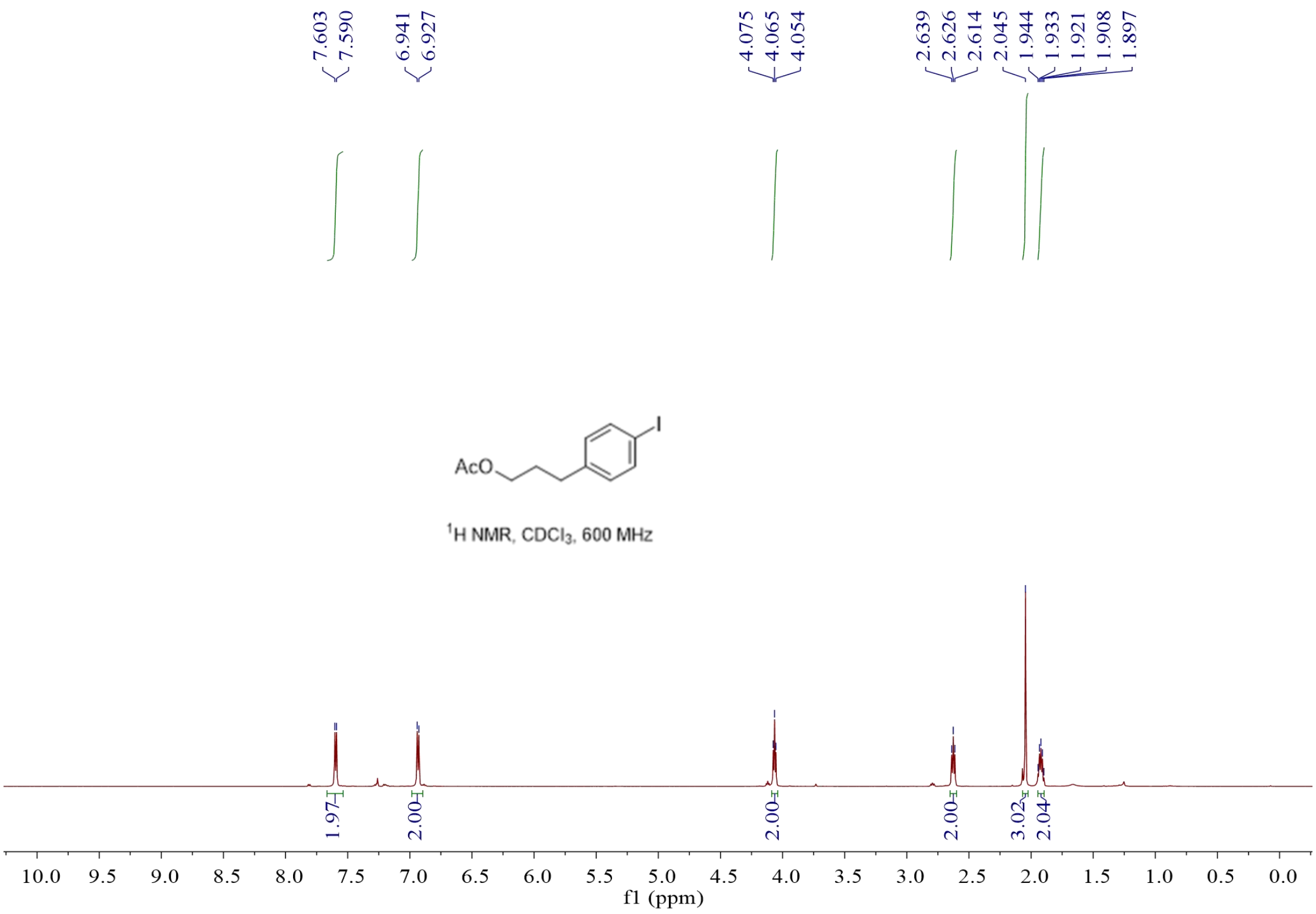
7.603
7.590
6.941
6.927
4.075
4.065
4.054
2.639
2.626
2.614
2.045
1.944
1.933
1.921
1.908
1.897
AcO
I
¹H NMR, CDCl₃, 600 MHz
1.97
2.00
2.00
2.00
3.02
2.04
10.0 9.5 9.0 8.5 8.0 7.5 7.0 6.5 6.0 5.5 5.0 4.5 4.0 3.5 3.0 2.5 2.0 1.5 1.0 0.5 0.0
f1 (ppm)

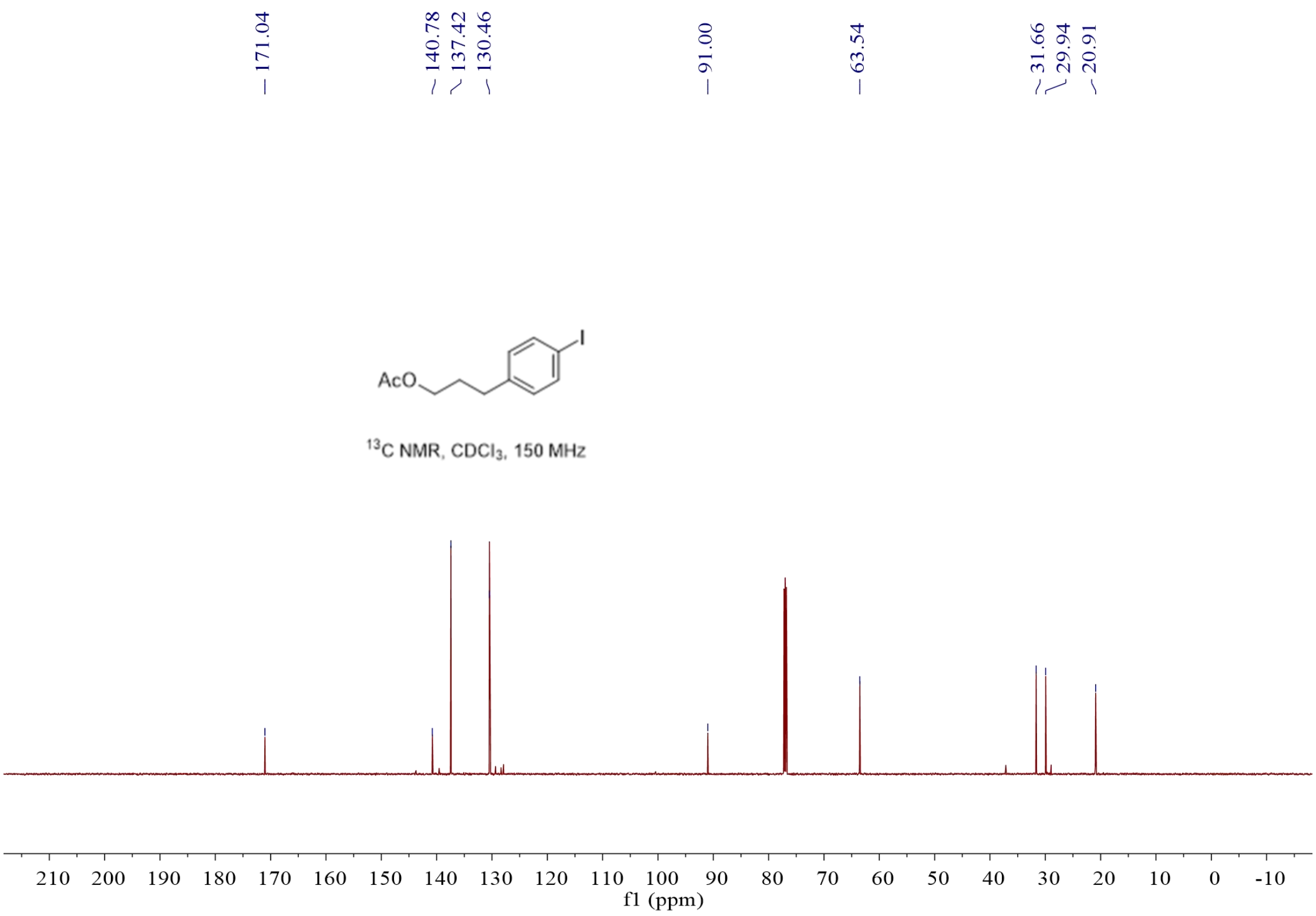
171.04
140.78
137.42
130.46
91.00
63.54
31.66
29.94
20.91
AcO
I
¹³C NMR, CDCl₃, 150 MHz
210 200 190 180 170 160 150 140 130 120 110 100 90 80 70 60 50 40 30 20 10 0 -10
f1 (ppm)

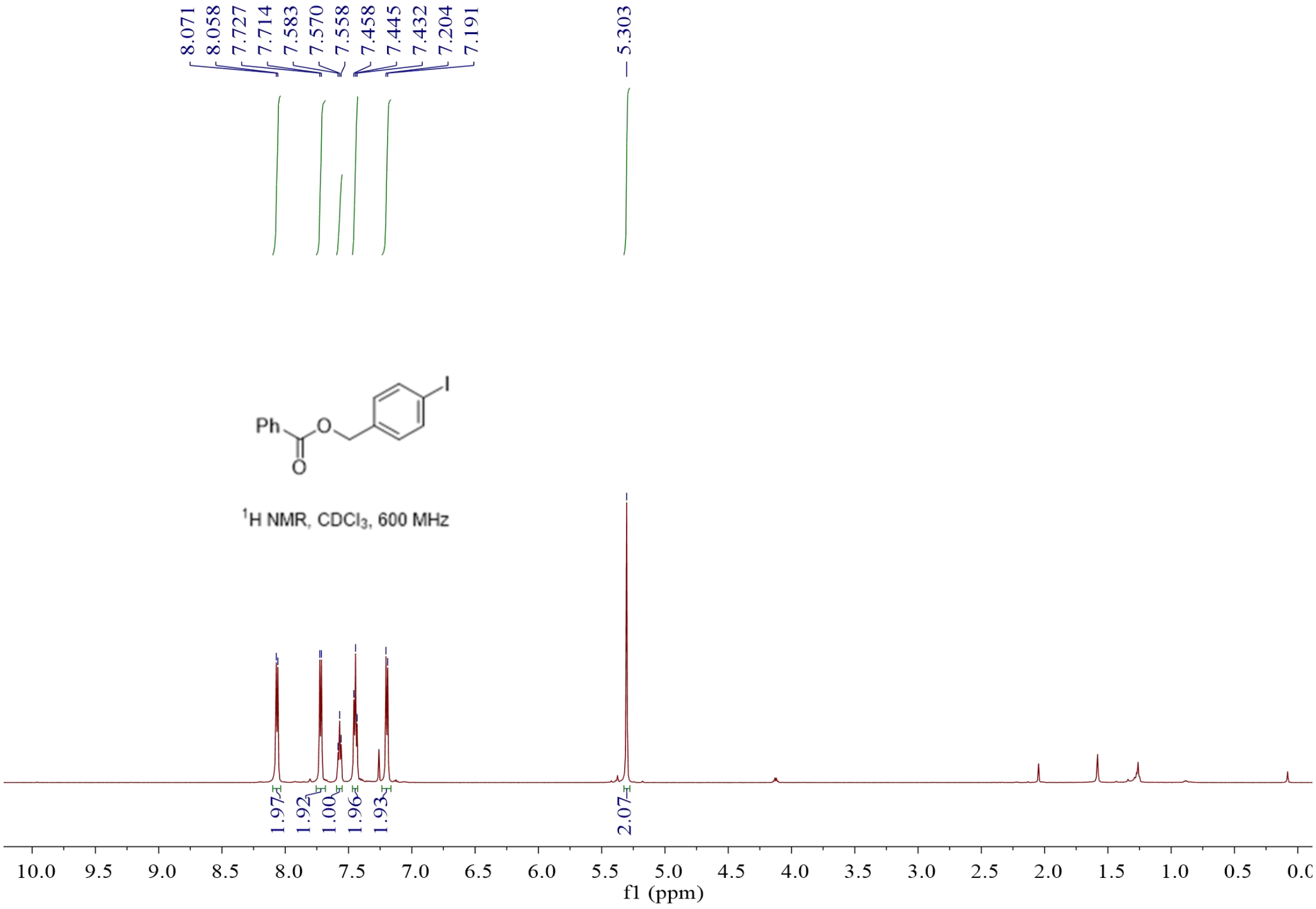
8.071
8.058
7.727
7.714
7.583
7.570
7.558
7.458
7.445
7.432
7.204
7.191
5.303
Ph
O
O
I
^{1}H NMR, CDCl$_3$, 600 MHz
1.97
1.92
1.00
1.96
1.93
2.07
f1 (ppm)

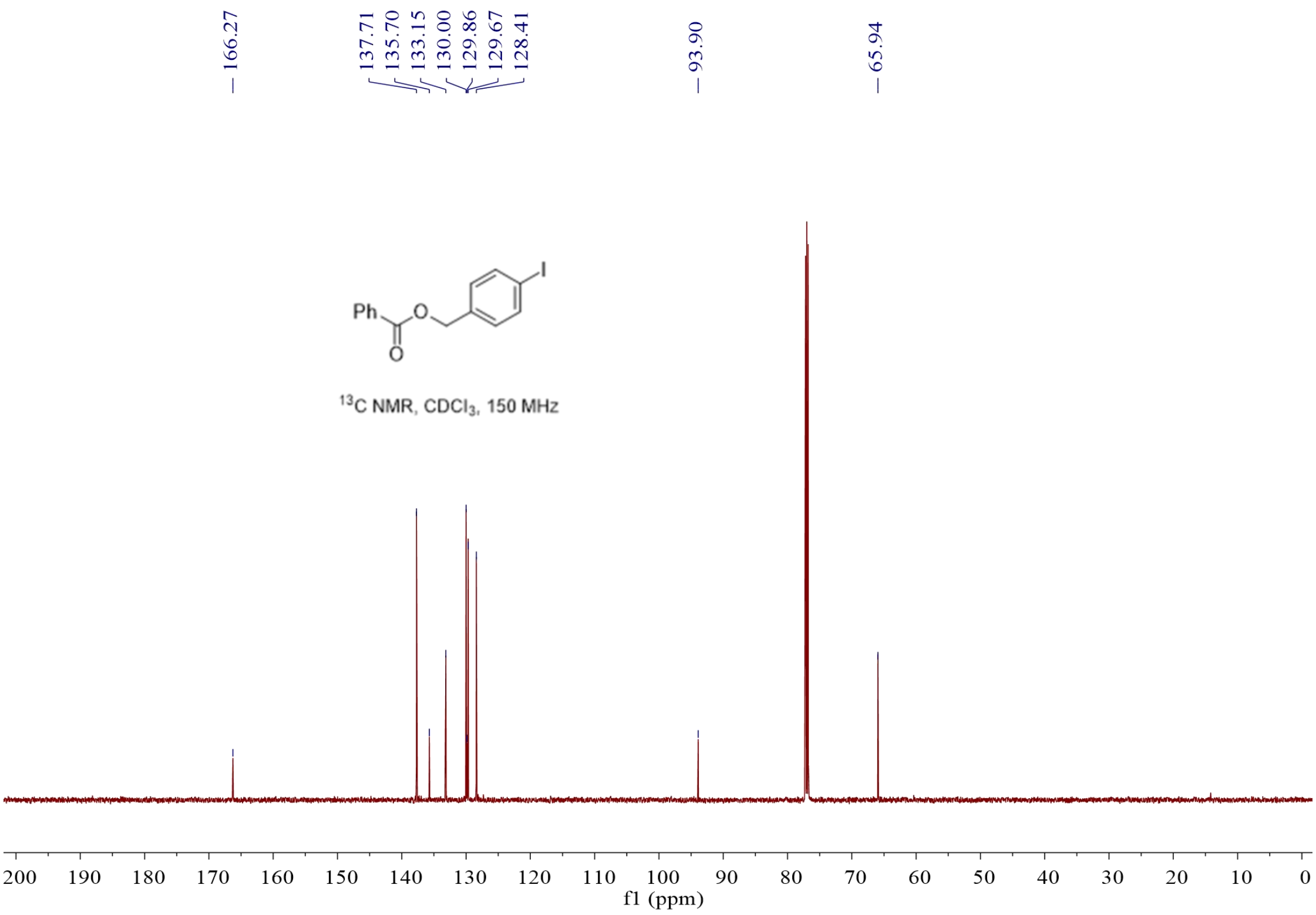
166.27
137.71
135.70
133.15
130.00
129.86
129.67
128.41
93.90
65.94
Ph
O
O
I
^{13}C NMR, CDCl$_3$, 150 MHz
f1 (ppm)

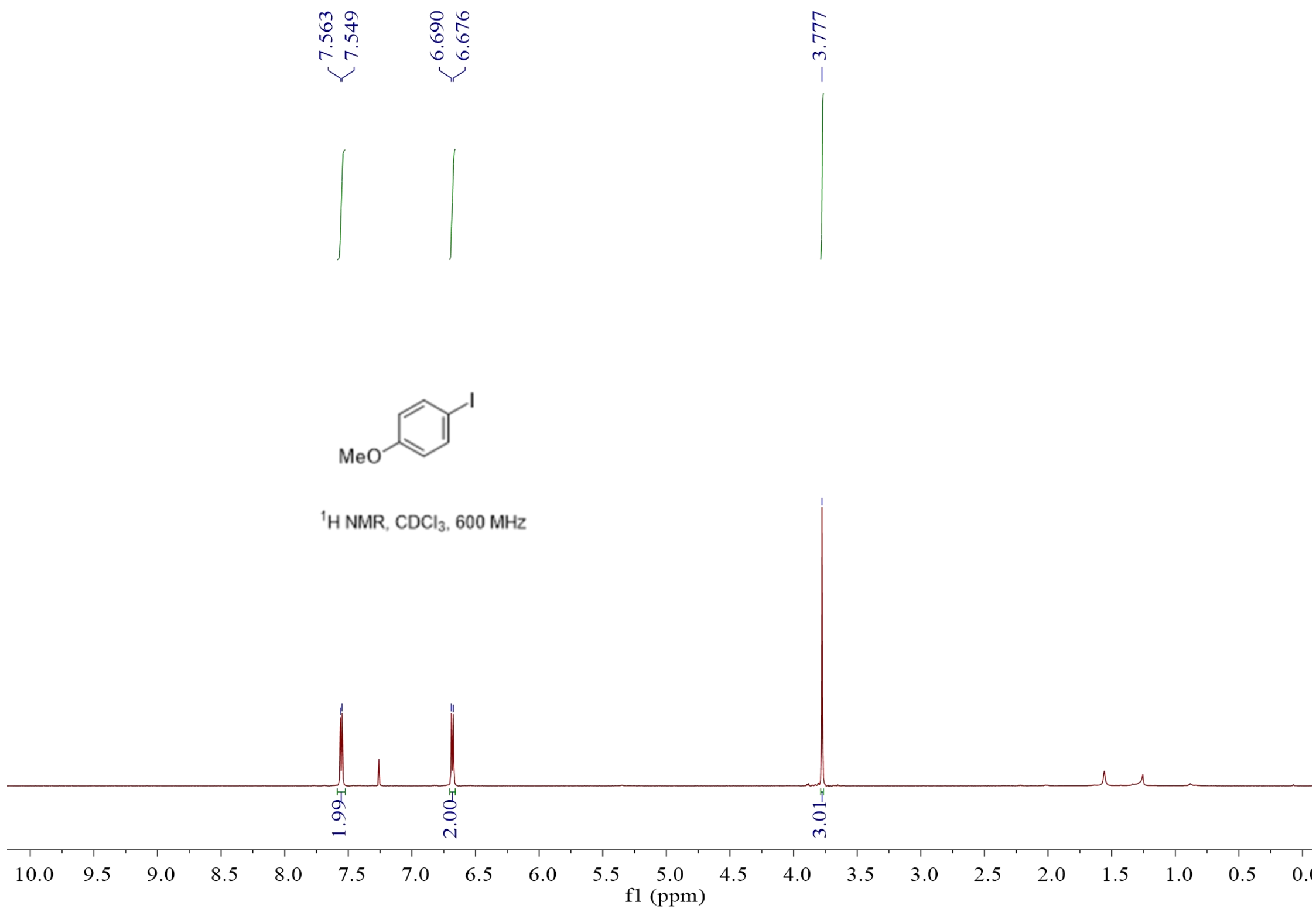
7.563
7.549
6.690
6.676
3.777
MeO
I
1H NMR, CDCl3, 600 MHz
1.99
2.00
3.01
10.0 9.5 9.0 8.5 8.0 7.5 7.0 6.5 6.0 5.5 5.0 4.5 4.0 3.5 3.0 2.5 2.0 1.5 1.0 0.5
f1 (ppm)

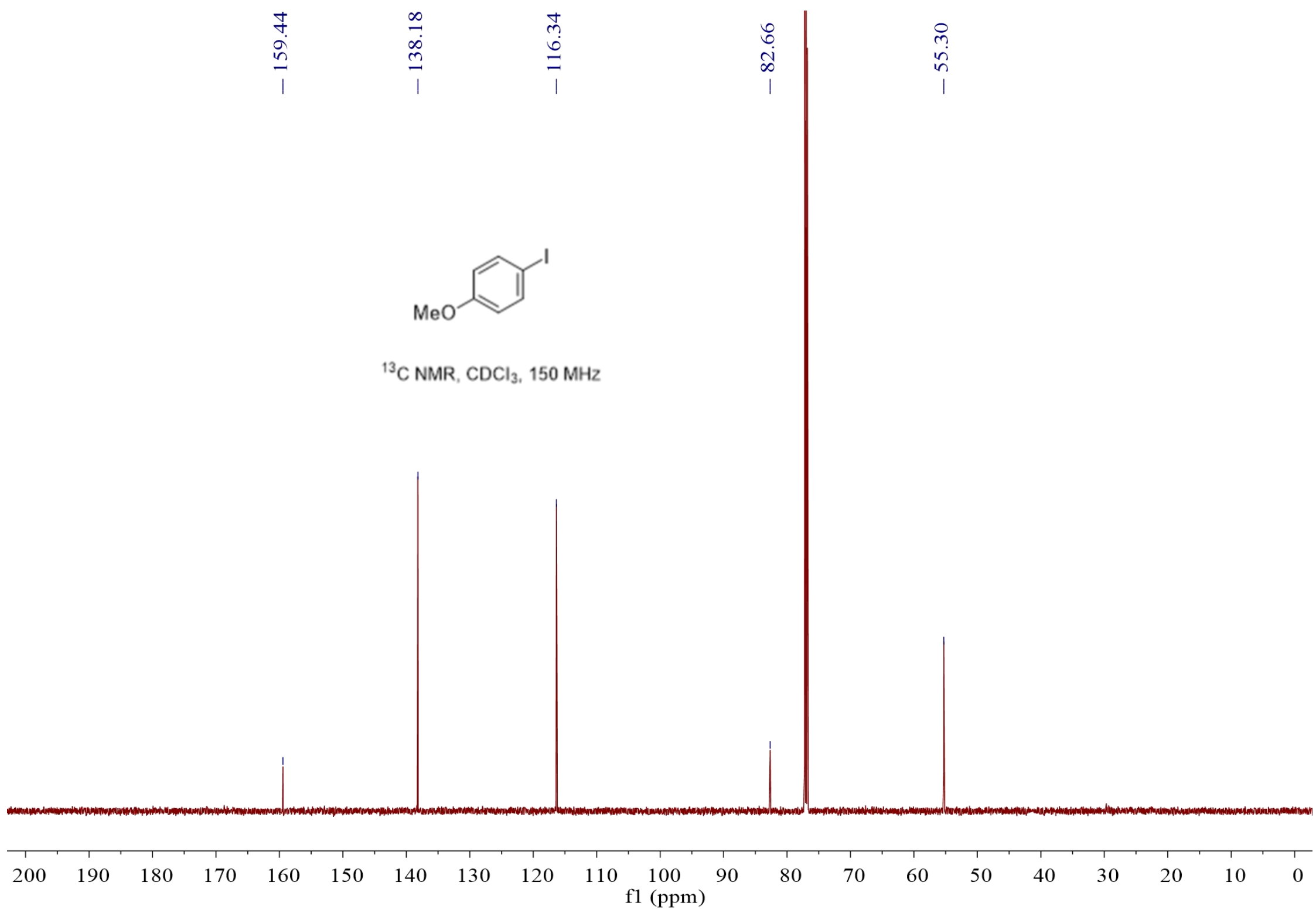
159.44
138.18
116.34
82.66
55.30
MeO
I
13C NMR, CDCl3, 150 MHz
200 190 180 170 160 150 140 130 120 110 100 90 80 70 60 50 40 30 20 10 0
f1 (ppm)

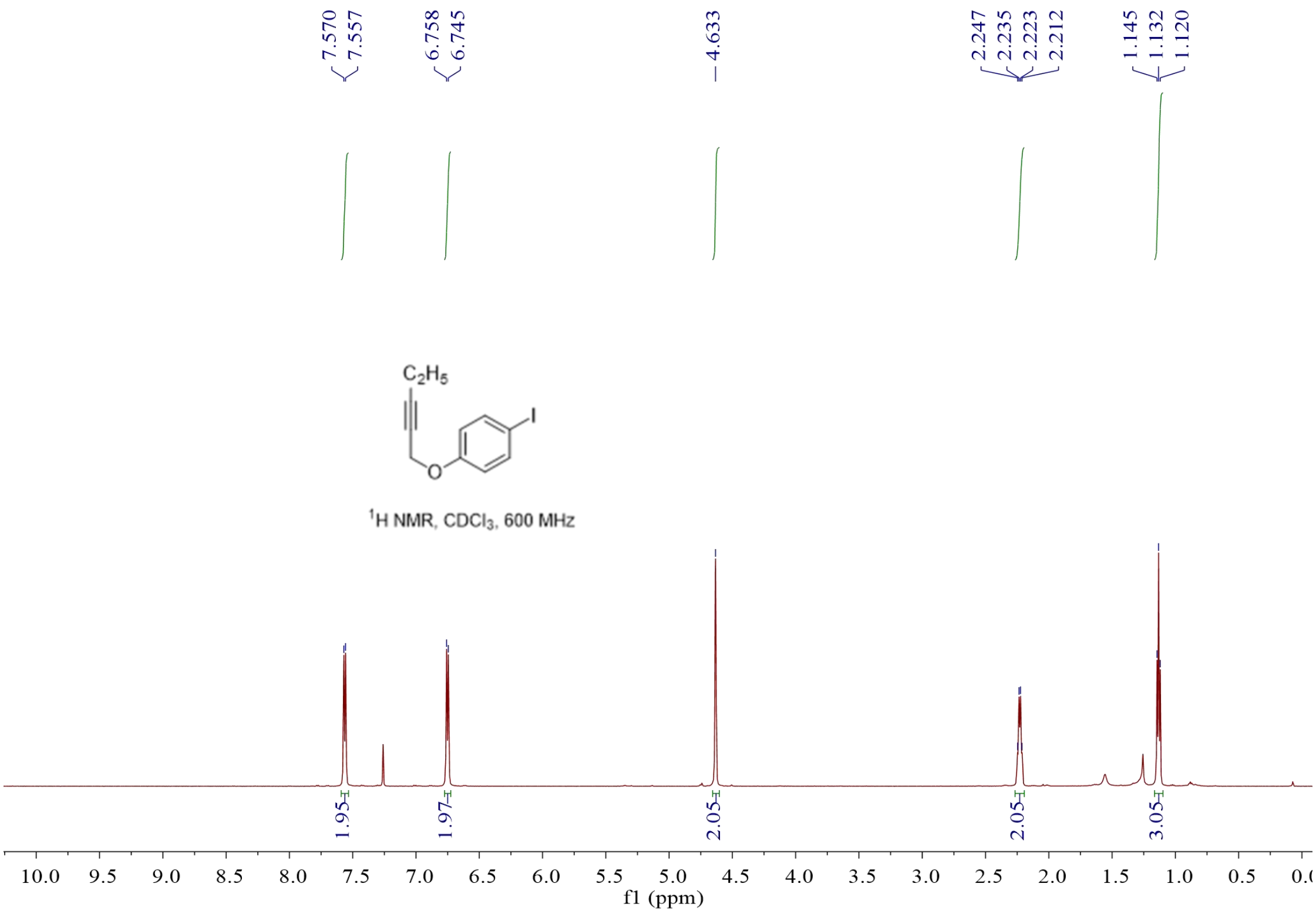
7.570
7.557
6.758
6.745
4.633
2.247
2.235
2.223
2.212
1.145
1.132
1.120
C2H5
I
O
1H NMR, CDCl3, 600 MHz
1.95
1.97
2.05
2.05
3.05
10.0 9.5 9.0 8.5 8.0 7.5 7.0 6.5 6.0 5.5 5.0 4.5 4.0 3.5 3.0 2.5 2.0 1.5 1.0 0.5
f1 (ppm)

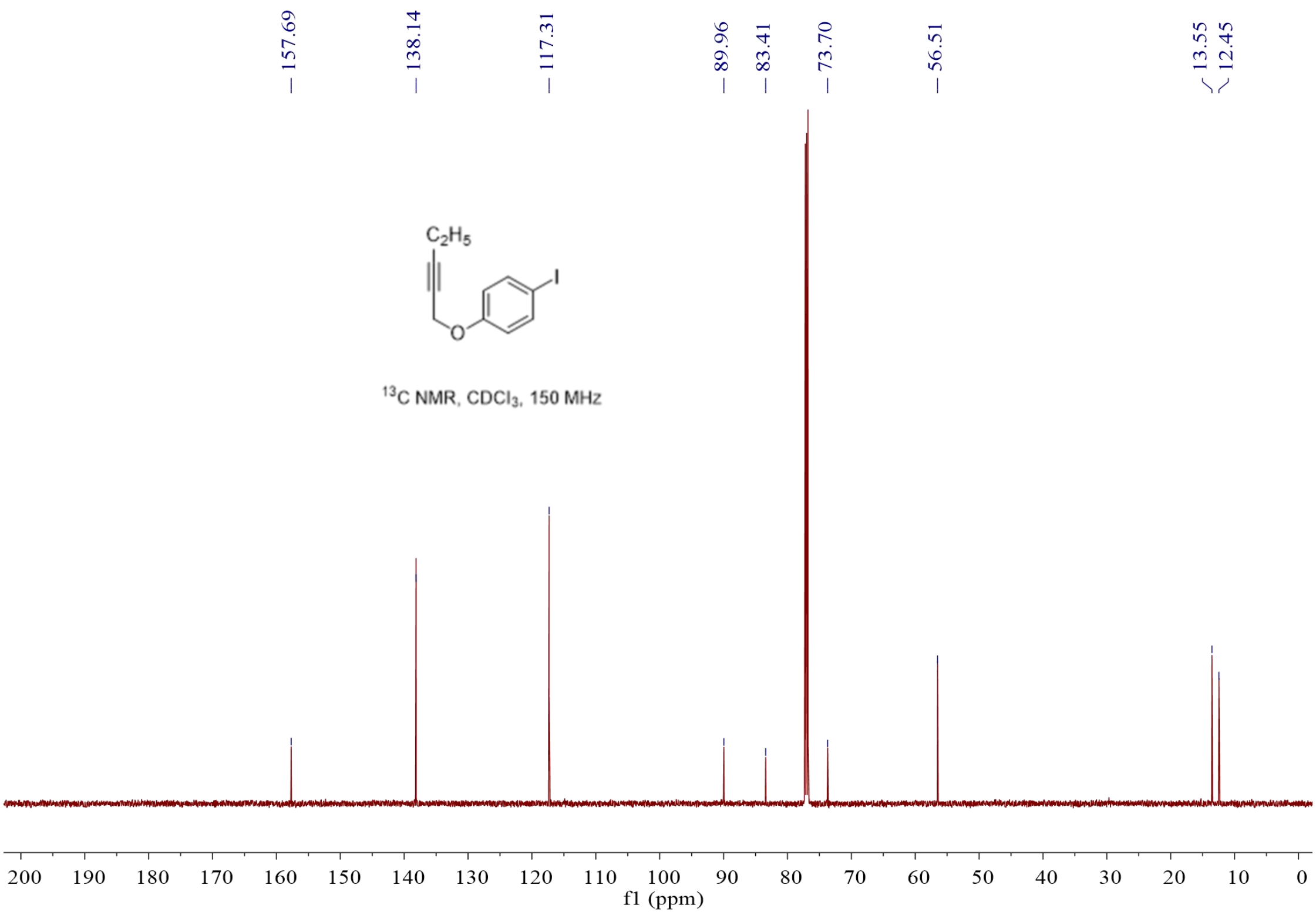
157.69
138.14
117.31
89.96
83.41
73.70
56.51
13.55
12.45
C2H5
I
O
13C NMR, CDCl3, 150 MHz
200 190 180 170 160 150 140 130 120 110 100 90 80 70 60 50 40 30 20 10 0
f1 (ppm)

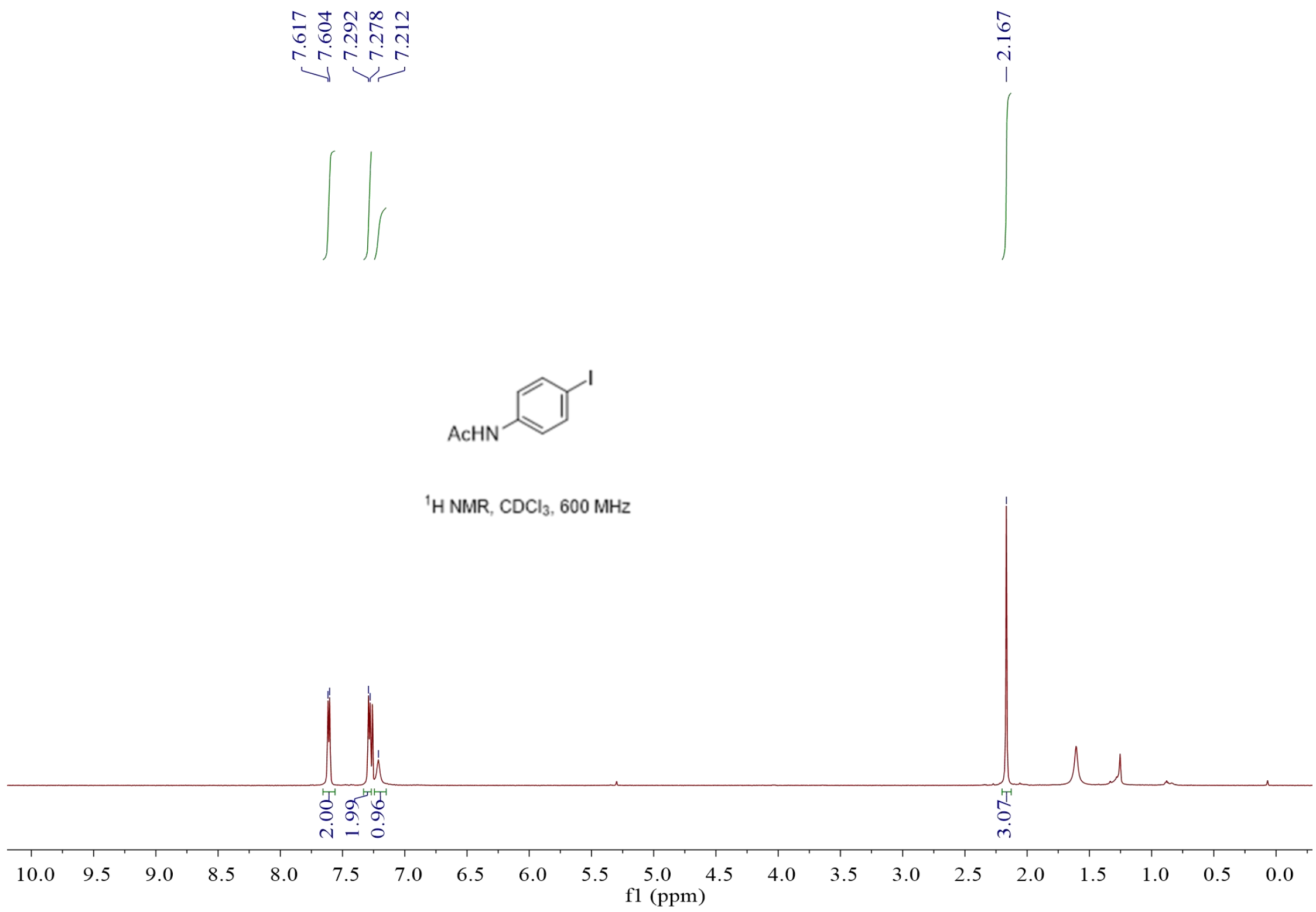
7.617
7.604
7.292
7.278
7.212
2.167
AcHN
I
1H NMR, CDCl3, 600 MHz
2.00
1.99
0.96
3.07
10.0 9.5 9.0 8.5 8.0 7.5 7.0 6.5 6.0 5.5 5.0 4.5 4.0 3.5 3.0 2.5 2.0 1.5 1.0 0.5 0.0
f1 (ppm)

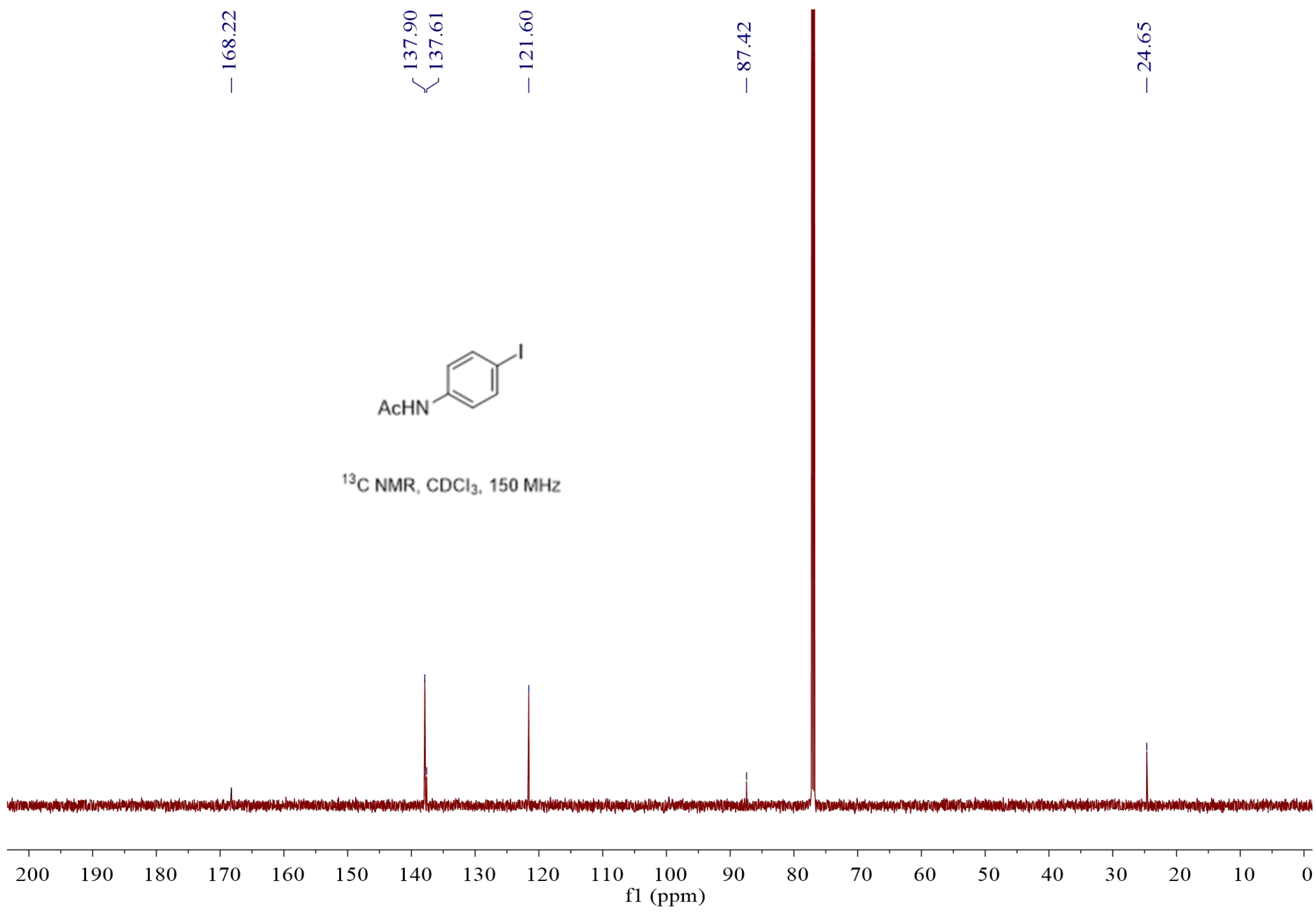
168.22
137.90
137.61
121.60
87.42
24.65
AcHN
I
13C NMR, CDCl3, 150 MHz
200 190 180 170 160 150 140 130 120 110 100 90 80 70 60 50 40 30 20 10 0
f1 (ppm)

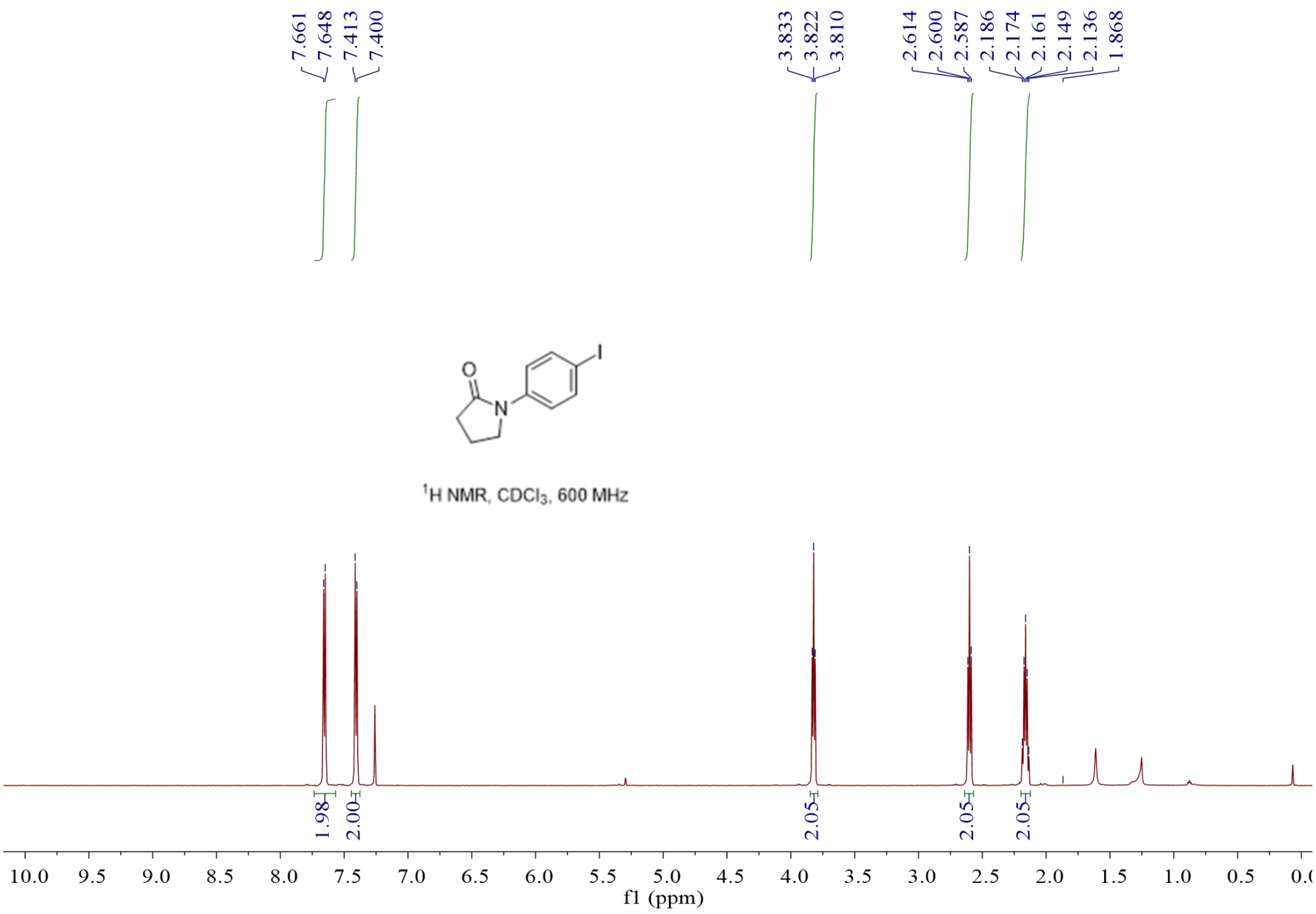
7.661
7.648
7.413
7.400
3.833
3.822
3.810
2.614
2.600
2.587
2.186
2.174
2.161
2.149
2.136
1.868
O
N
I
1H NMR, CDCl3, 600 MHz
1.98
2.00
2.05
2.05
2.05
10.0 9.5 9.0 8.5 8.0 7.5 7.0 6.5 6.0 5.5 5.0 4.5 4.0 3.5 3.0 2.5 2.0 1.5 1.0 0.5
f1 (ppm)

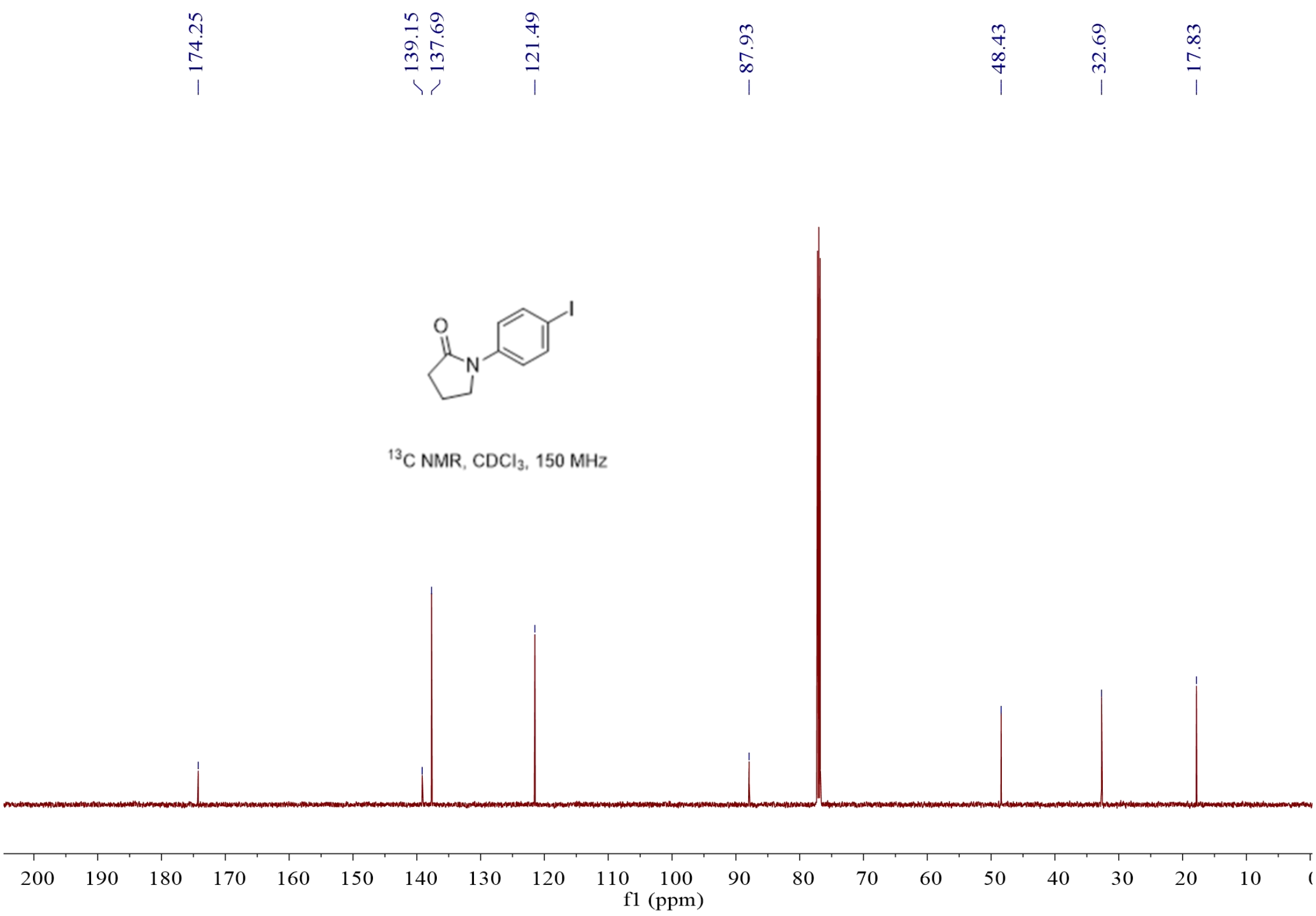
174.25
139.15
137.69
121.49
87.93
48.43
32.69
17.83
O
N
I
13C NMR, CDCl3, 150 MHz
200 190 180 170 160 150 140 130 120 110 100 90 80 70 60 50 40 30 20 10
f1 (ppm)

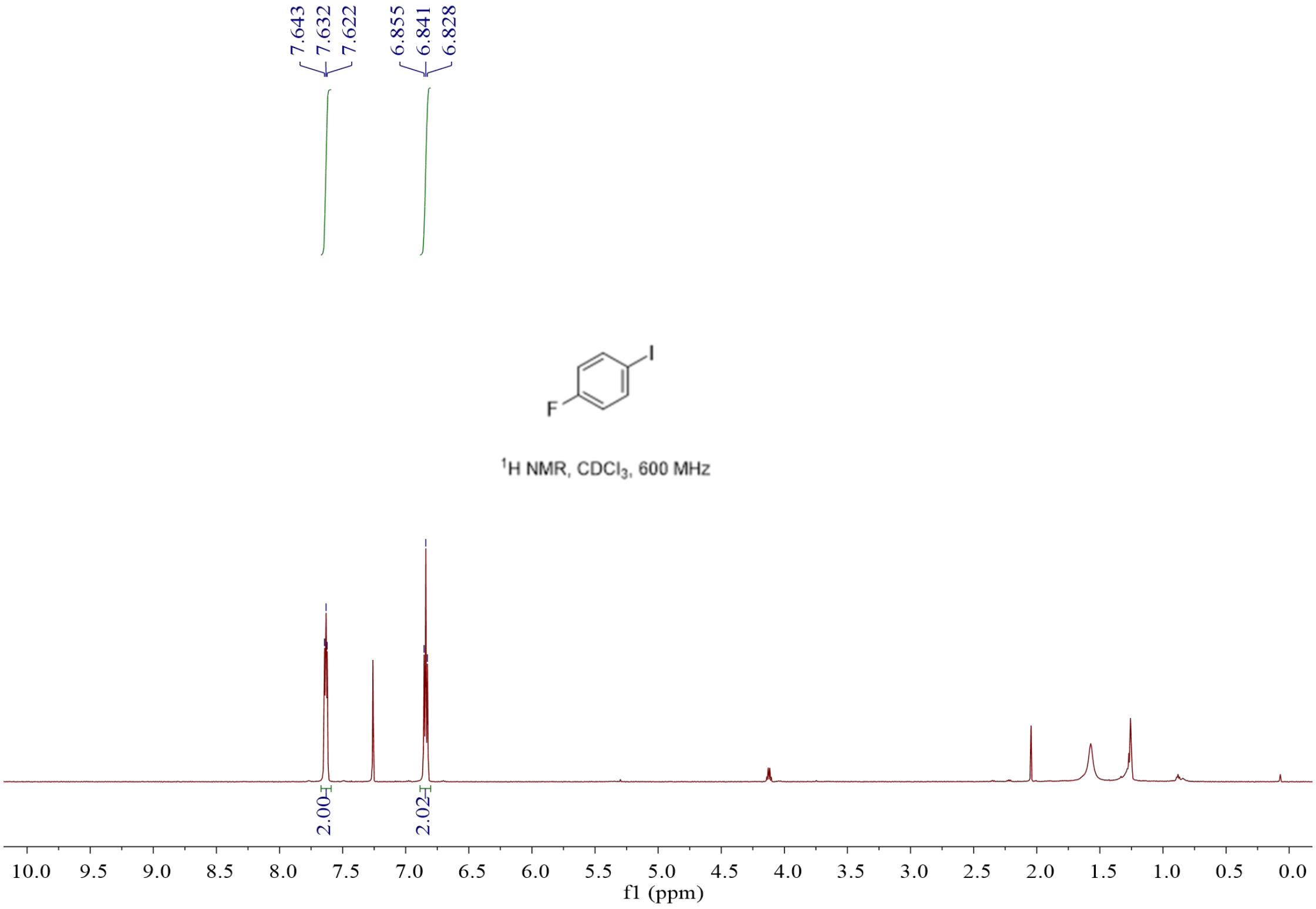
7.643
7.632
7.622
6.855
6.841
6.828
F
I
^{1}H NMR, CDCl$_3$, 600 MHz
2.00
2.02
10.0 9.5 9.0 8.5 8.0 7.5 7.0 6.5 6.0 5.5 5.0 4.5 4.0 3.5 3.0 2.5 2.0 1.5 1.0 0.5 0.0
f1 (ppm)

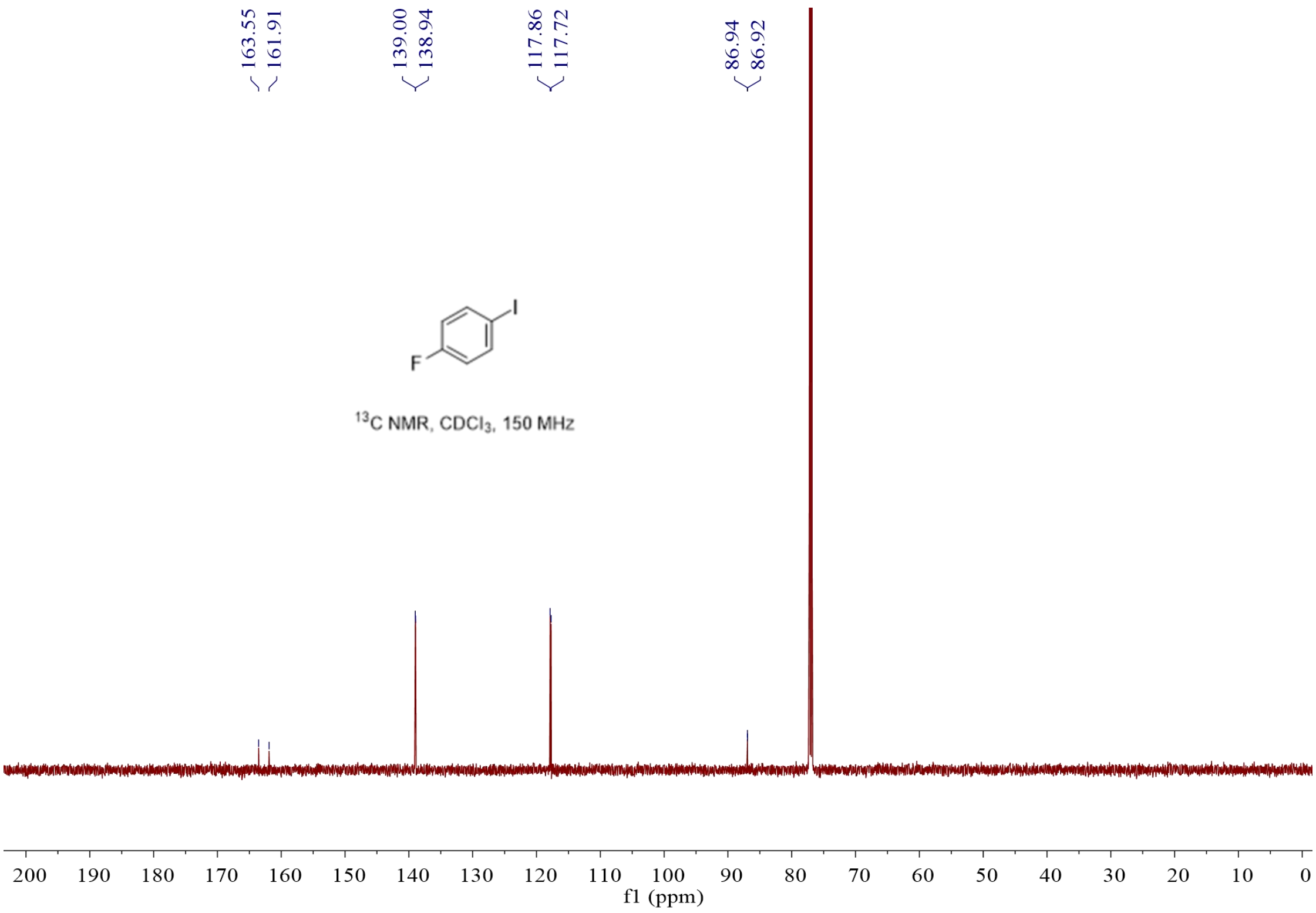
163.55
161.91
139.00
138.94
117.86
117.72
86.94
86.92
F
I
^{13}C NMR, CDCl$_3$, 150 MHz
200 190 180 170 160 150 140 130 120 110 100 90 80 70 60 50 40 30 20 10 0
f1 (ppm)

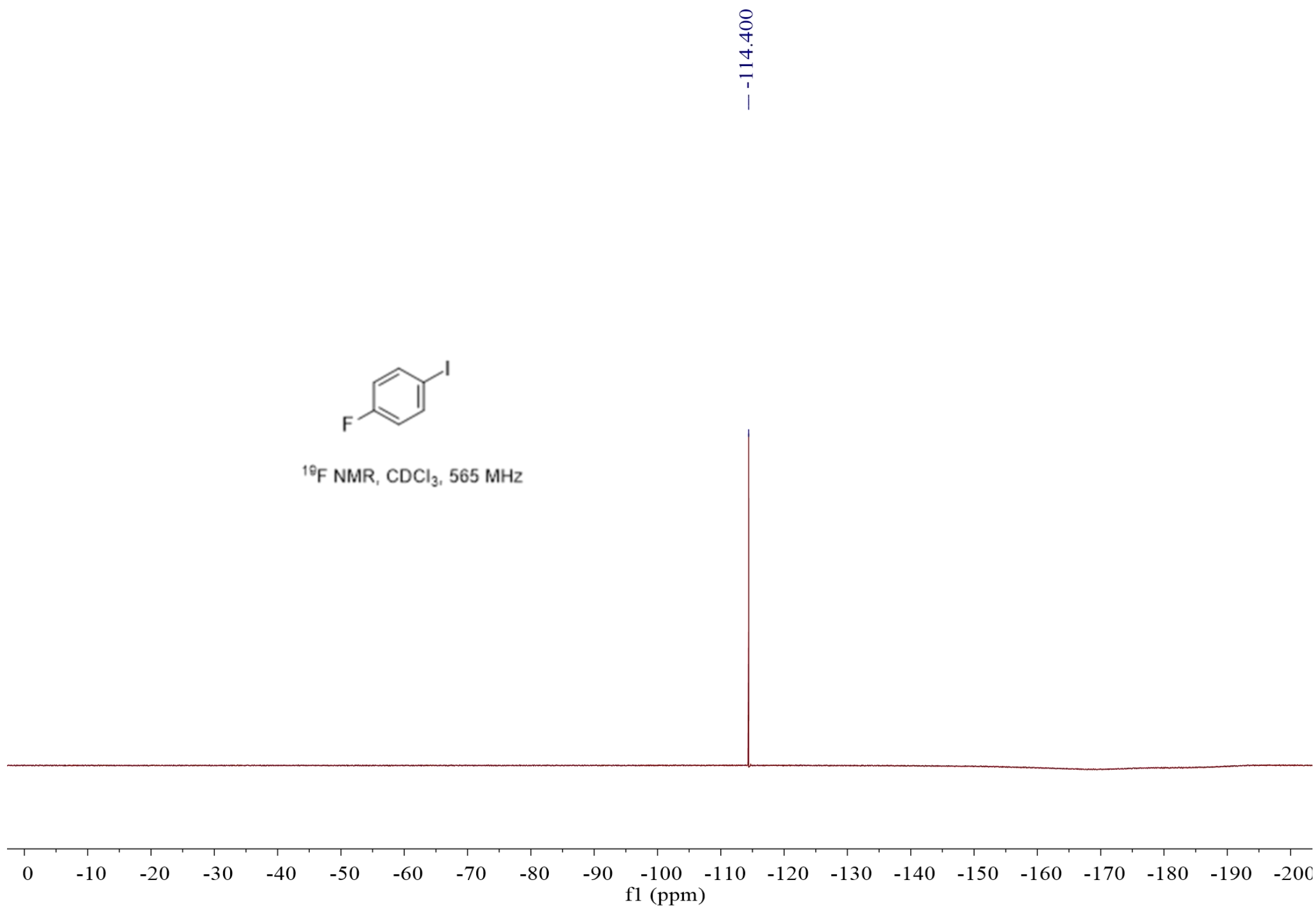

-114.400
F
I
19F NMR, CDCl3, 565 MHz
0 -10 -20 -30 -40 -50 -60 -70 -80 -90 -100 -110 -120 -130 -140 -150 -160 -170 -180 -190 -200
f1 (ppm)

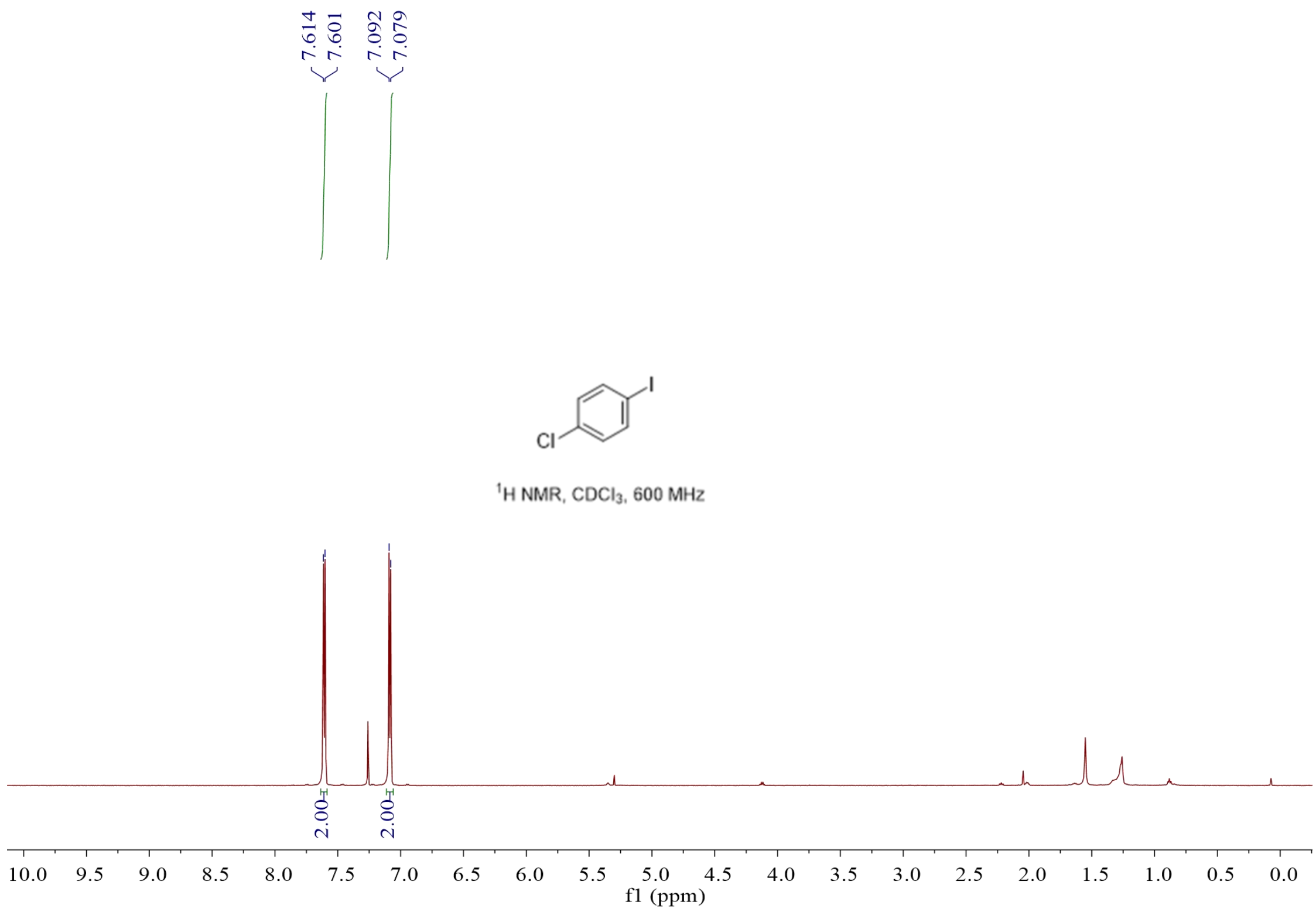

7.614
7.601
7.092
7.079
Cl
I
1H NMR, CDCl3, 600 MHz
2.00
2.00
10.0 9.5 9.0 8.5 8.0 7.5 7.0 6.5 6.0 5.5 5.0 4.5 4.0 3.5 3.0 2.5 2.0 1.5 1.0 0.5 0.0
f1 (ppm)

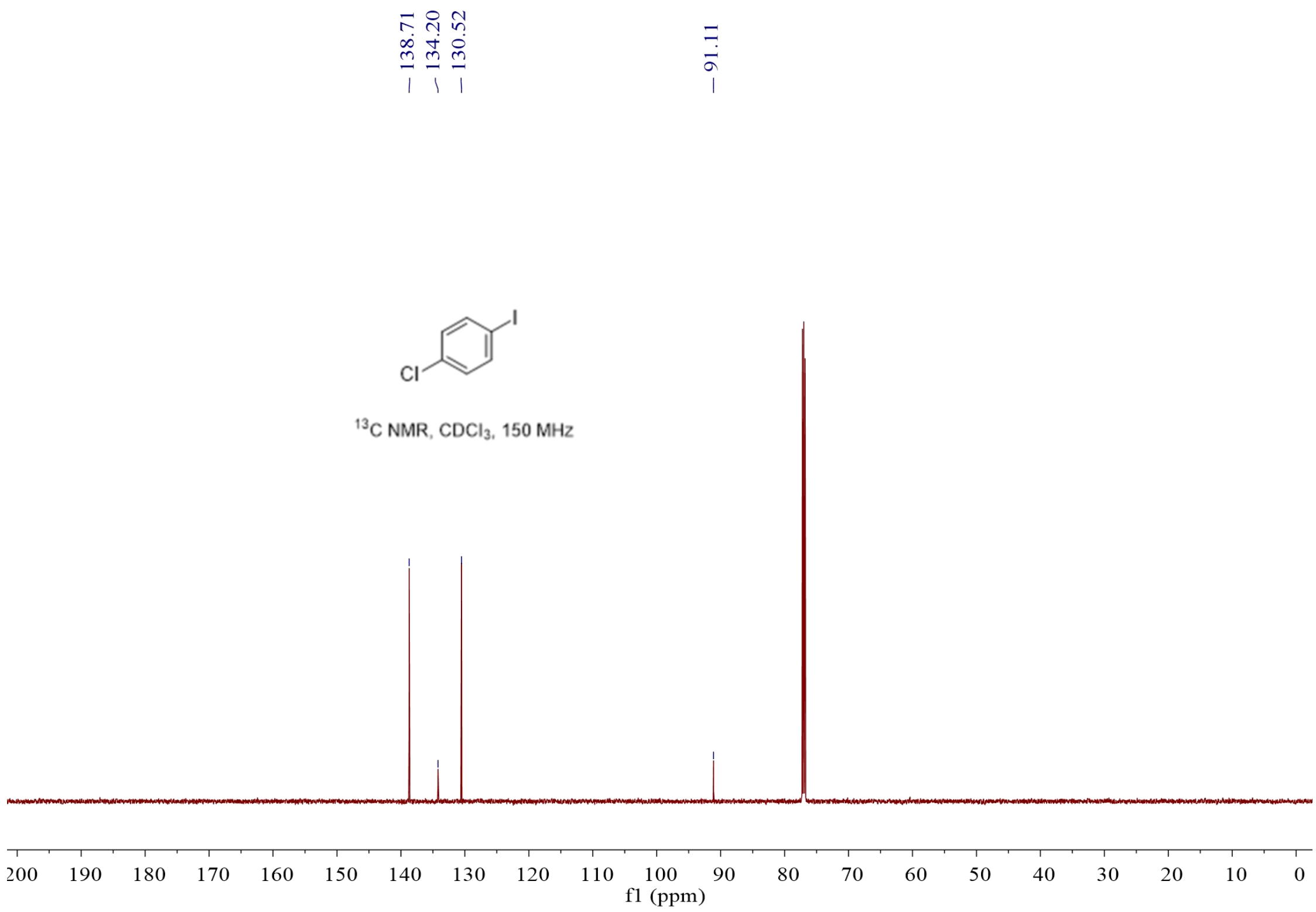
138.71
134.20
130.52
91.11
Cl
I
13C NMR, CDCl3, 150 MHz
200 190 180 170 160 150 140 130 120 110 100 90 80 70 60 50 40 30 20 10 0
f1 (ppm)

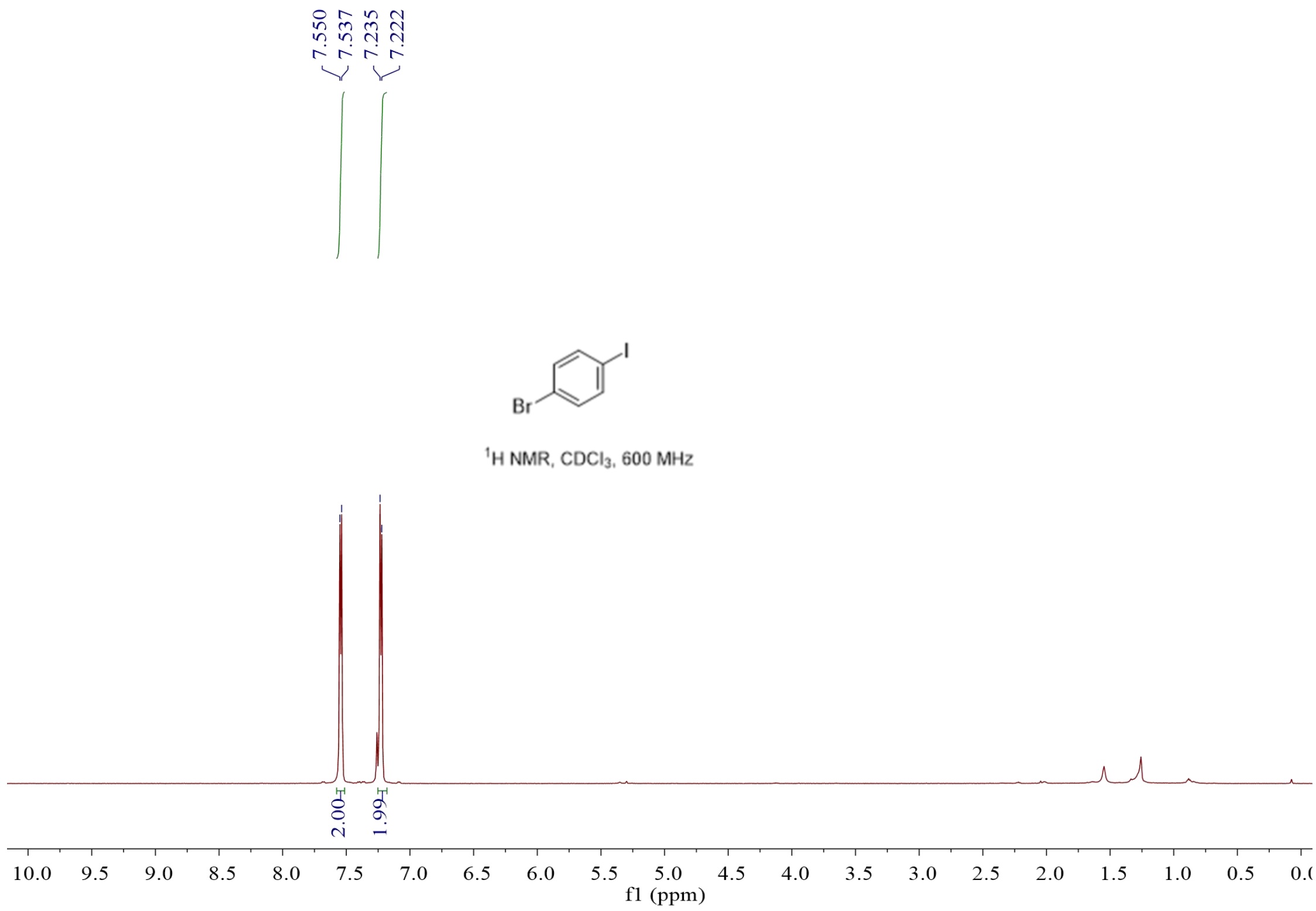
7.550
7.537
7.235
7.222
Br
I
1H NMR, CDCl3, 600 MHz
2.00
1.99
10.0 9.5 9.0 8.5 8.0 7.5 7.0 6.5 6.0 5.5 5.0 4.5 4.0 3.5 3.0 2.5 2.0 1.5 1.0 0.5 0.0
f1 (ppm)

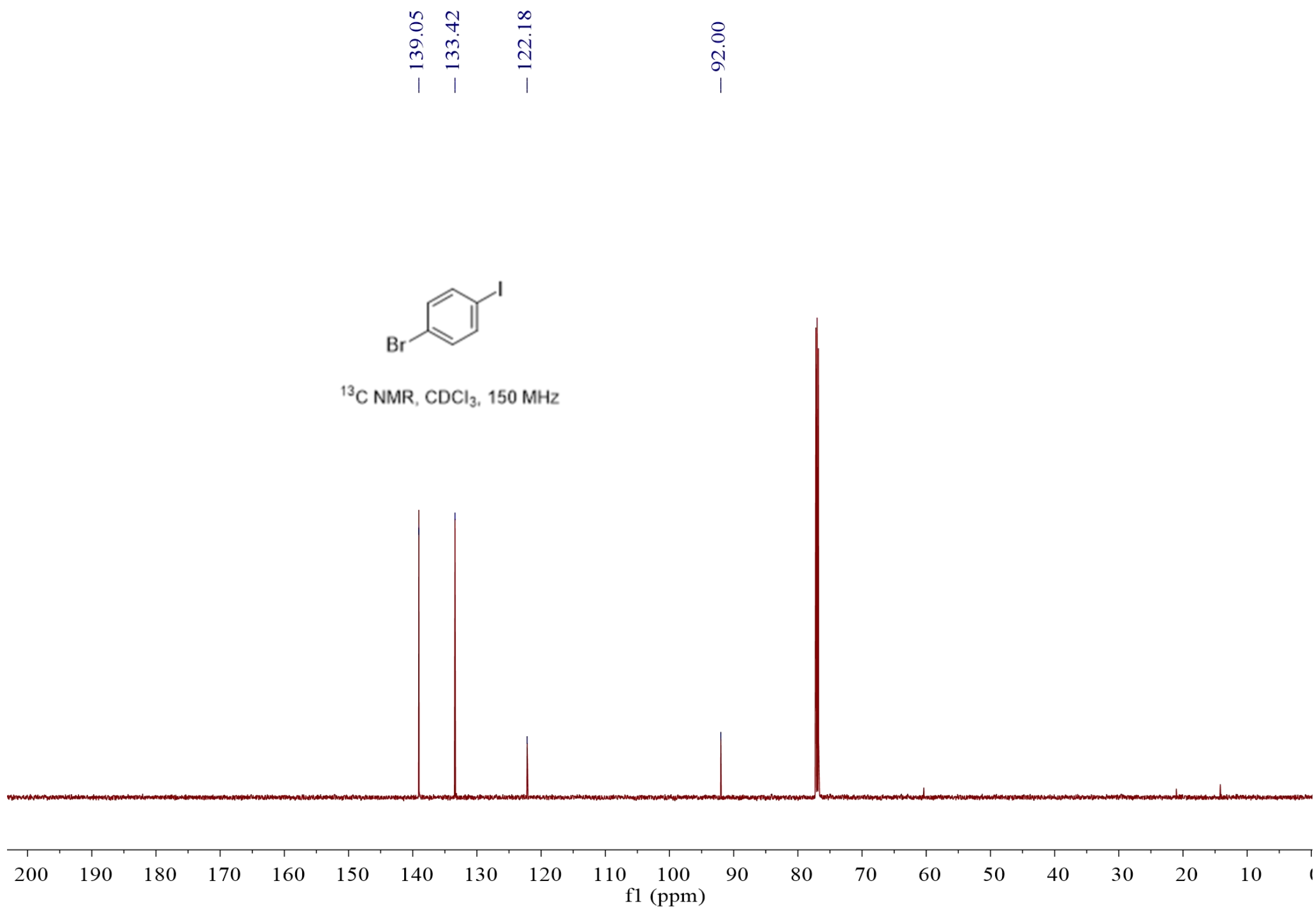
139.05
133.42
122.18
92.00
Br
I
^{13}C NMR, $CDCl_3$, 150 MHz
200 190 180 170 160 150 140 130 120 110 100 90 80 70 60 50 40 30 20 10
f1 (ppm)

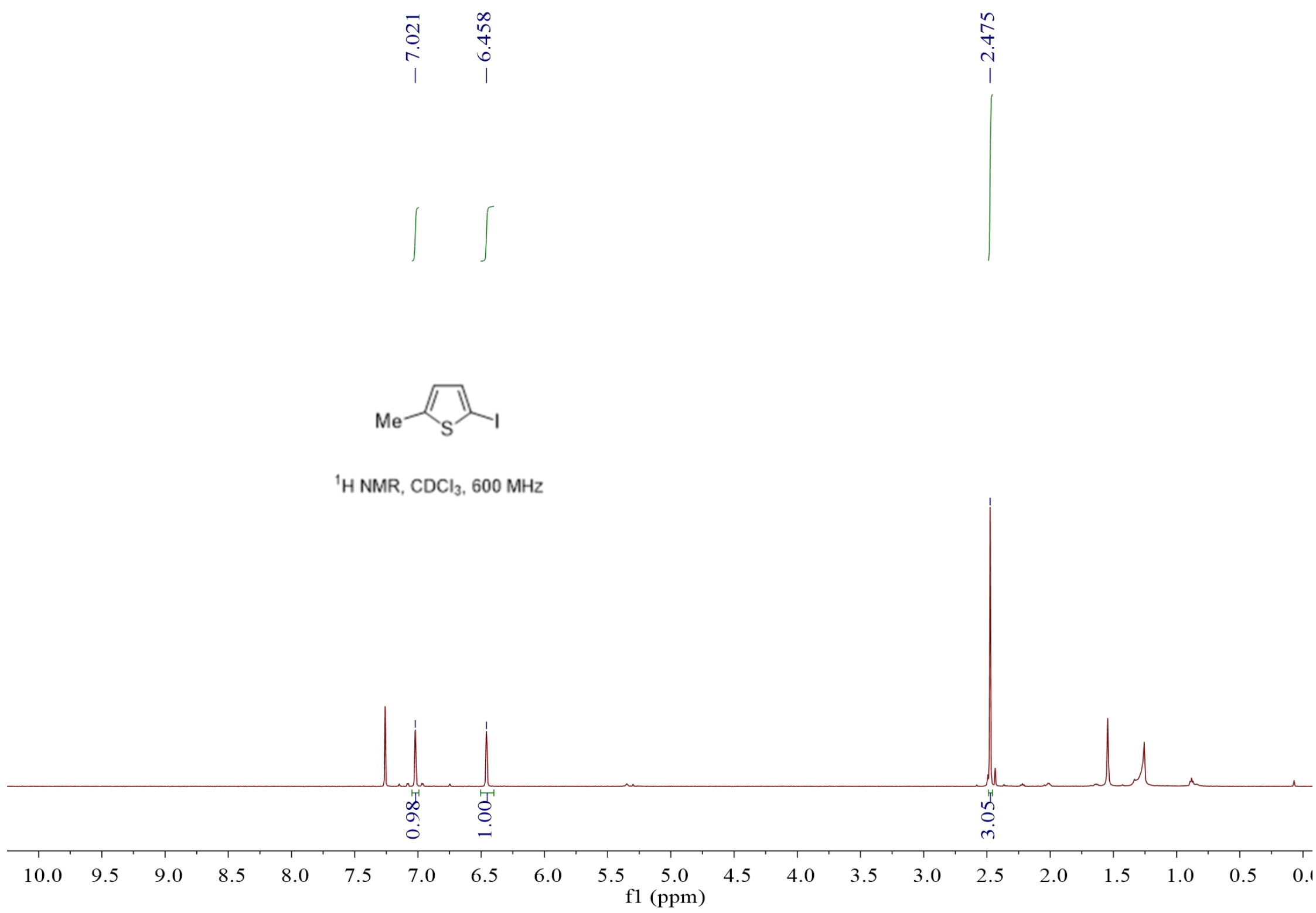
7.021
6.458
2.475
Me
S
I
^1H NMR, $CDCl_3$, 600 MHz
0.98
1.00
3.05
10.0 9.5 9.0 8.5 8.0 7.5 7.0 6.5 6.0 5.5 5.0 4.5 4.0 3.5 3.0 2.5 2.0 1.5 1.0 0.5
f1 (ppm)

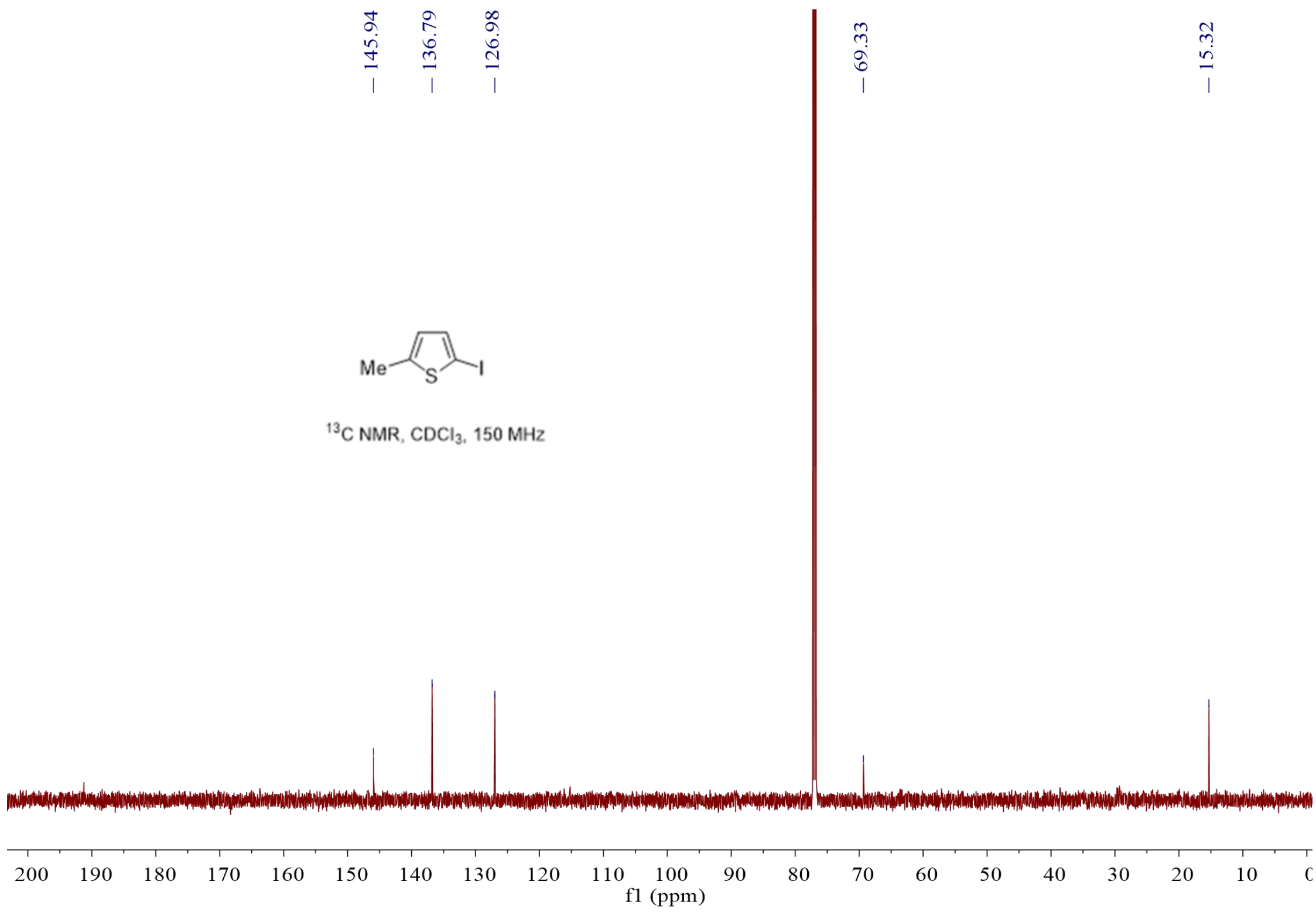
145.94
136.79
126.98
69.33
15.32
Me
S
I
13C NMR, CDCl3, 150 MHz
200 190 180 170 160 150 140 130 120 110 100 90 80 70 60 50 40 30 20 10 0
f1 (ppm)

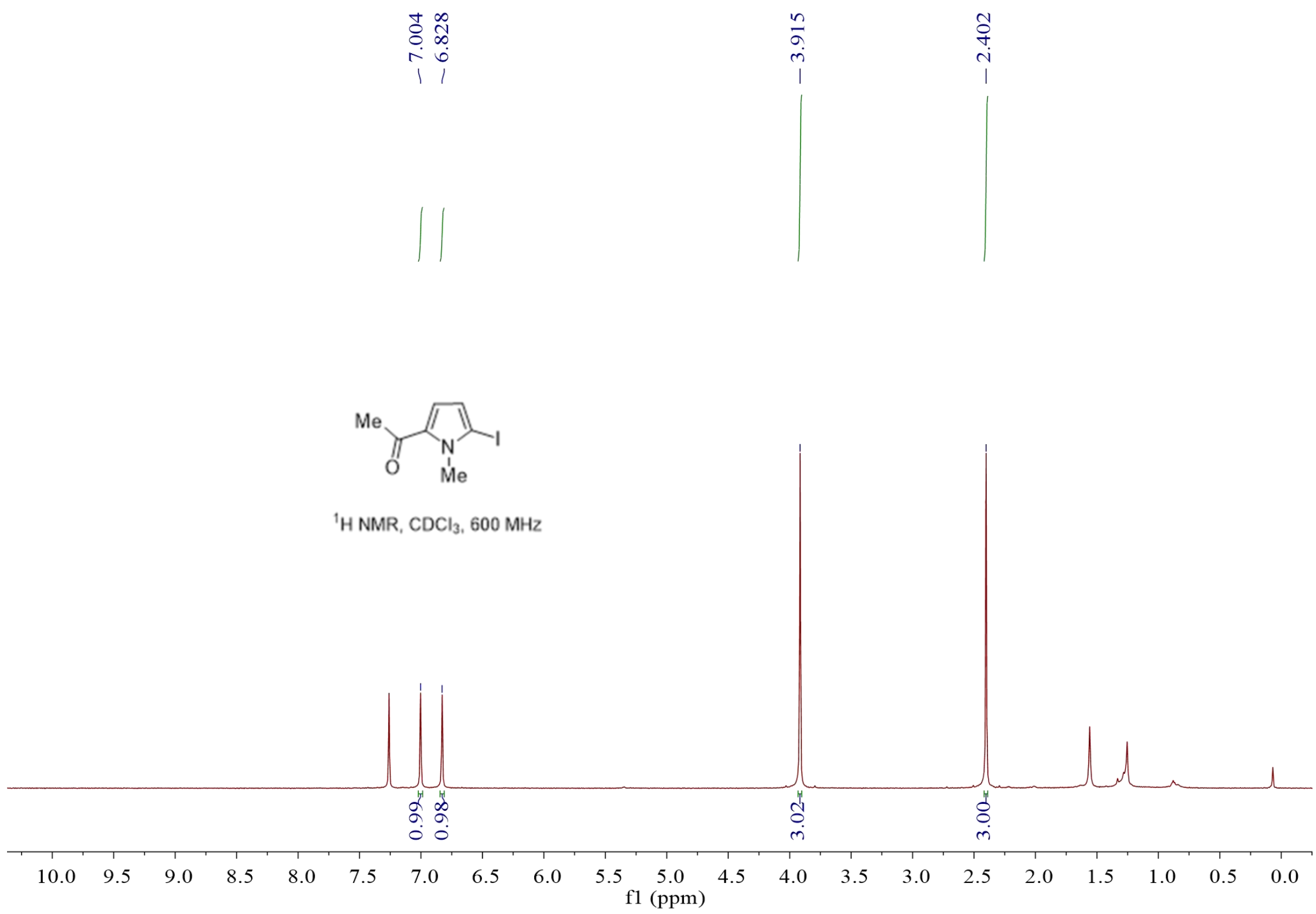
7.004
6.828
3.915
2.402
Me
O
N
Me
I
1H NMR, CDCl3, 600 MHz
0.99
0.98
3.02
3.00
10.0 9.5 9.0 8.5 8.0 7.5 7.0 6.5 6.0 5.5 5.0 4.5 4.0 3.5 3.0 2.5 2.0 1.5 1.0 0.5 0.0
f1 (ppm)

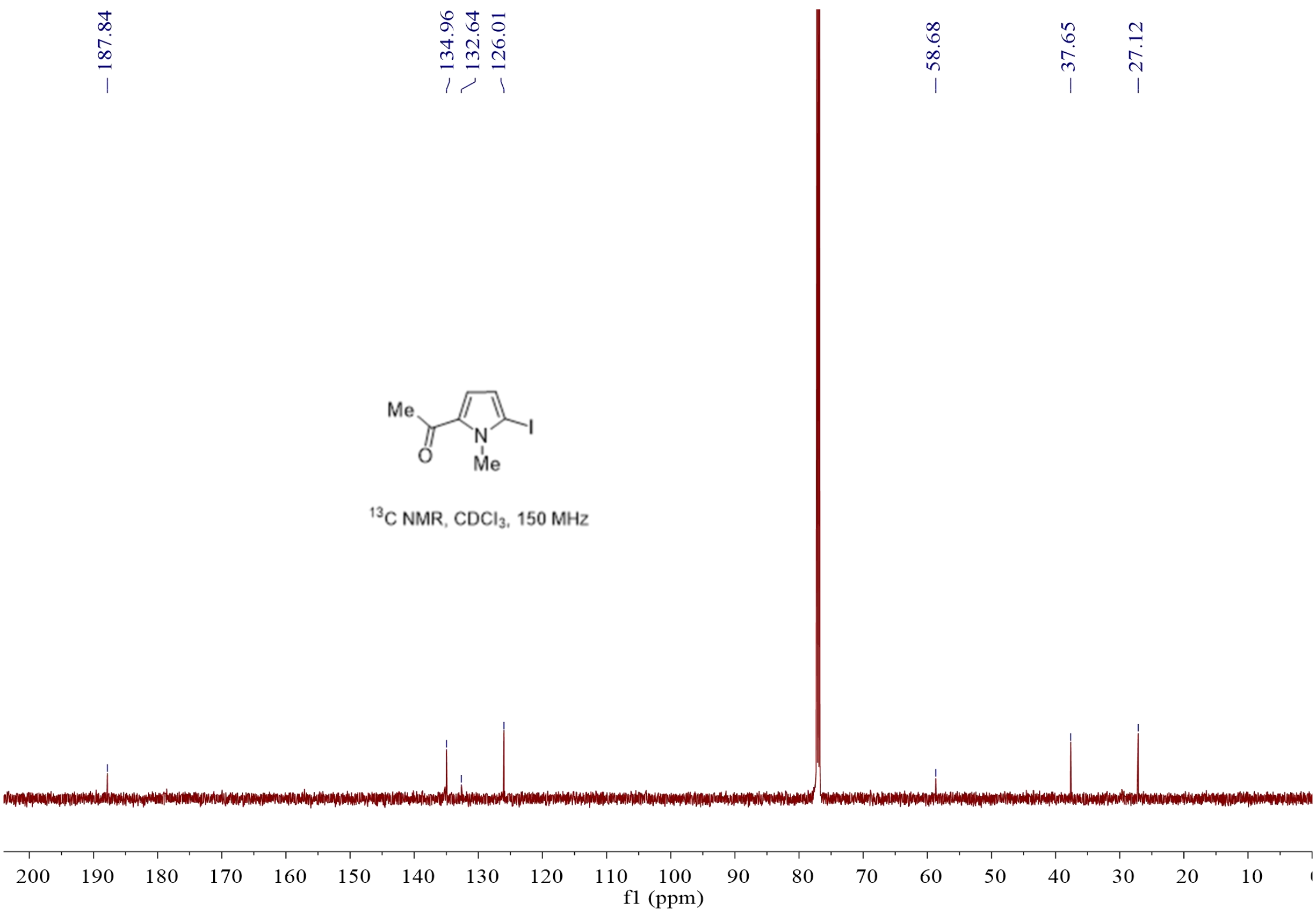
187.84
134.96
132.64
126.01
58.68
37.65
27.12
Me
O
N
Me
I
13C NMR, CDCl3, 150 MHz
200 190 180 170 160 150 140 130 120 110 100 90 80 70 60 50 40 30 20 10
f1 (ppm)

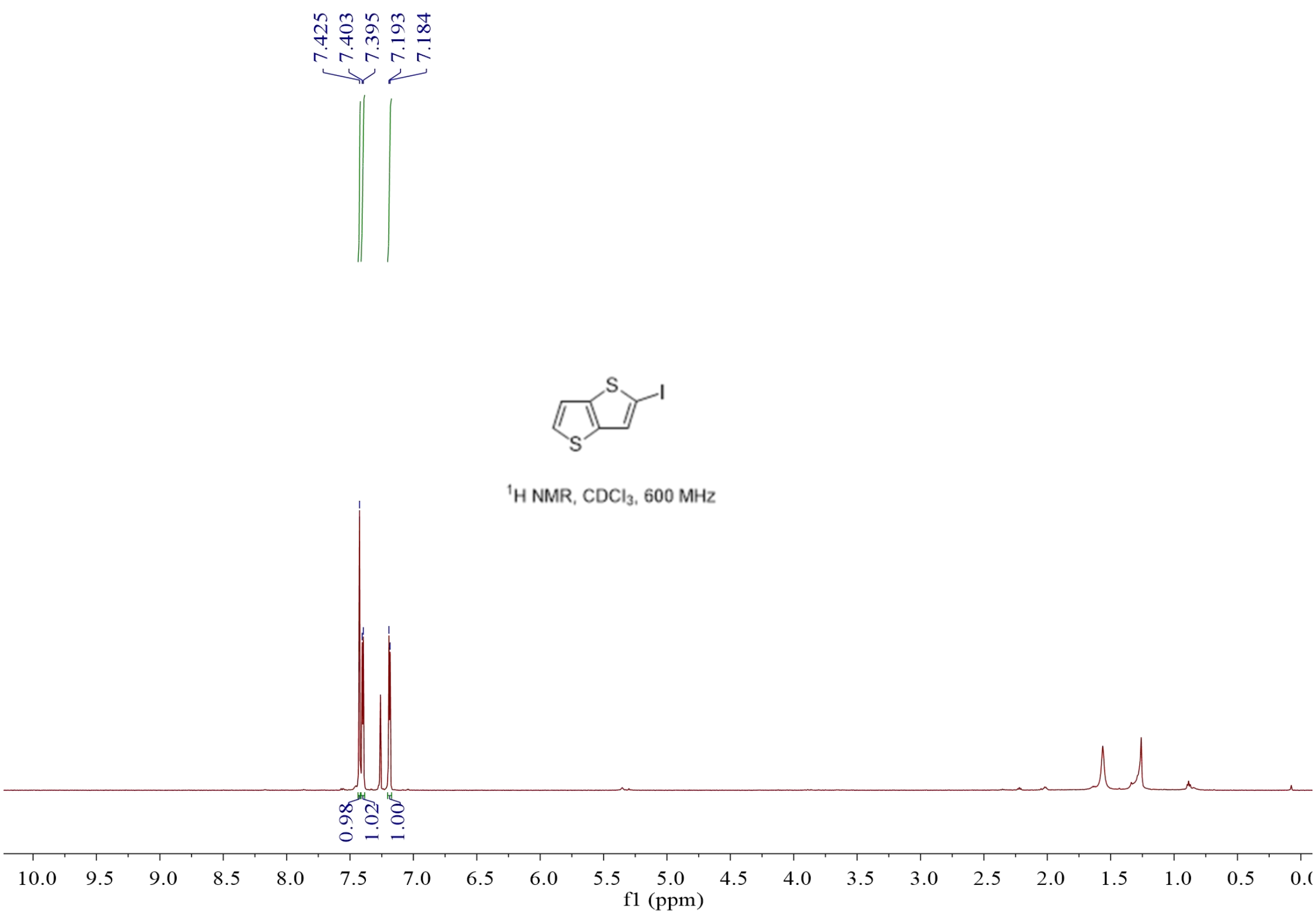
7.425
7.403
7.395
7.193
7.184
S
S
I
1H NMR, CDCl3, 600 MHz
0.98
1.02
1.00
10.0 9.5 9.0 8.5 8.0 7.5 7.0 6.5 6.0 5.5 5.0 4.5 4.0 3.5 3.0 2.5 2.0 1.5 1.0 0.5
f1 (ppm)

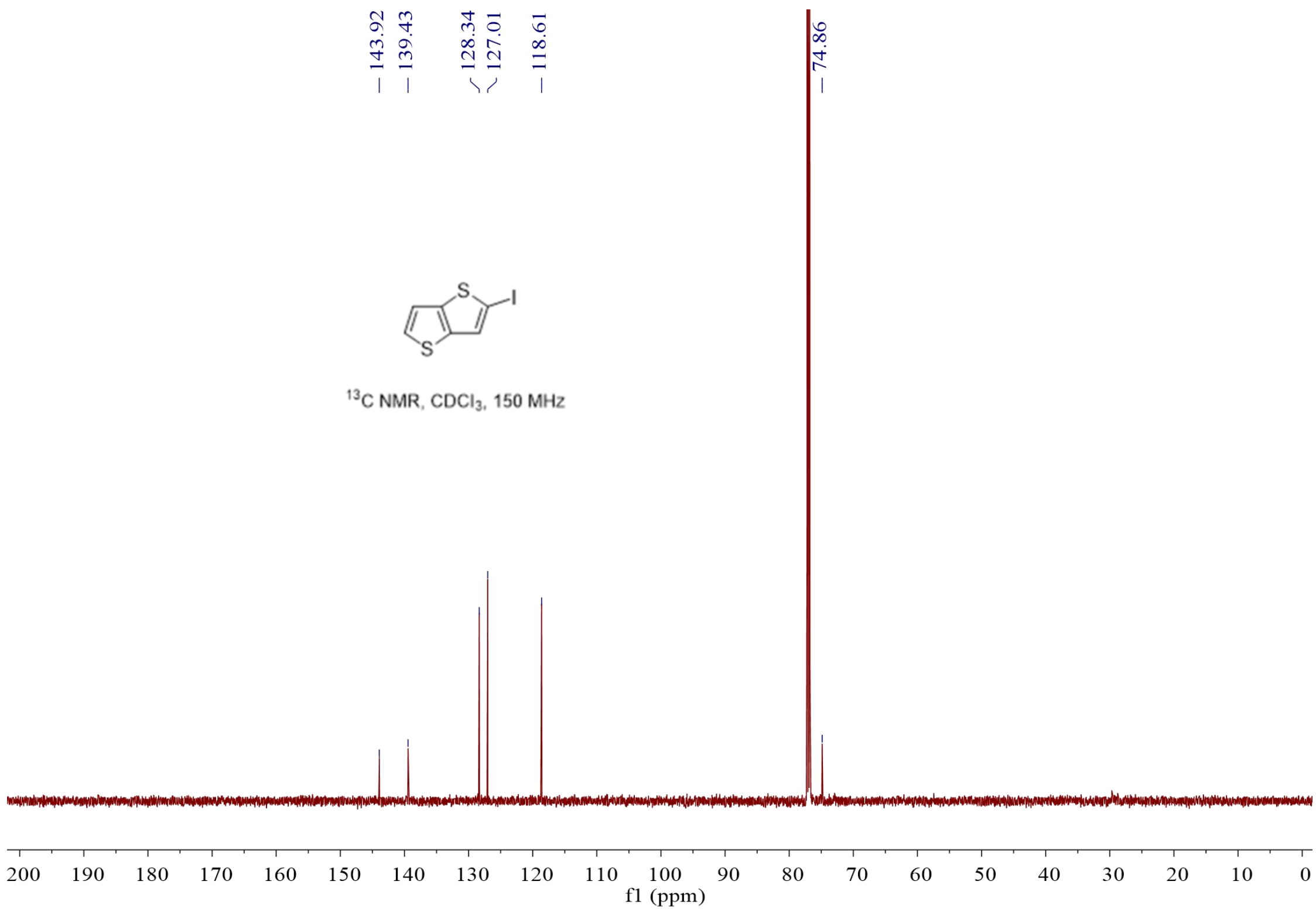
143.92
139.43
128.34
127.01
118.61
74.86
S
S
I
^{13}C NMR, $CDCl_3$, 150 MHz
200 190 180 170 160 150 140 130 120 110 100 90 80 70 60 50 40 30 20 10 0
f1 (ppm)

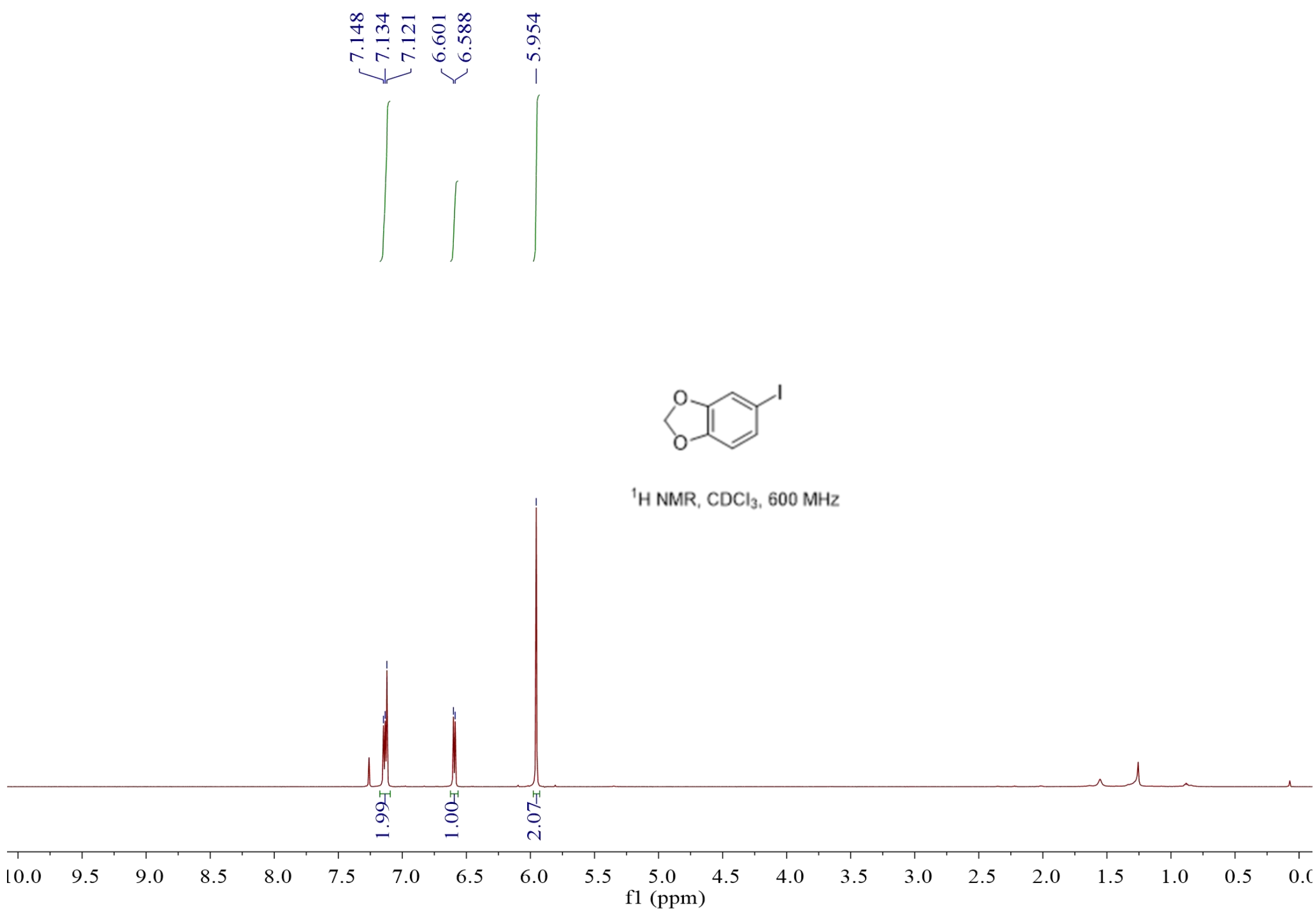
7.148
7.134
7.121
6.601
6.588
5.954
O
O
I
^{1}H NMR, $CDCl_3$, 600 MHz
1.99
1.00
2.07
10.0 9.5 9.0 8.5 8.0 7.5 7.0 6.5 6.0 5.5 5.0 4.5 4.0 3.5 3.0 2.5 2.0 1.5 1.0 0.5 0.0
f1 (ppm)

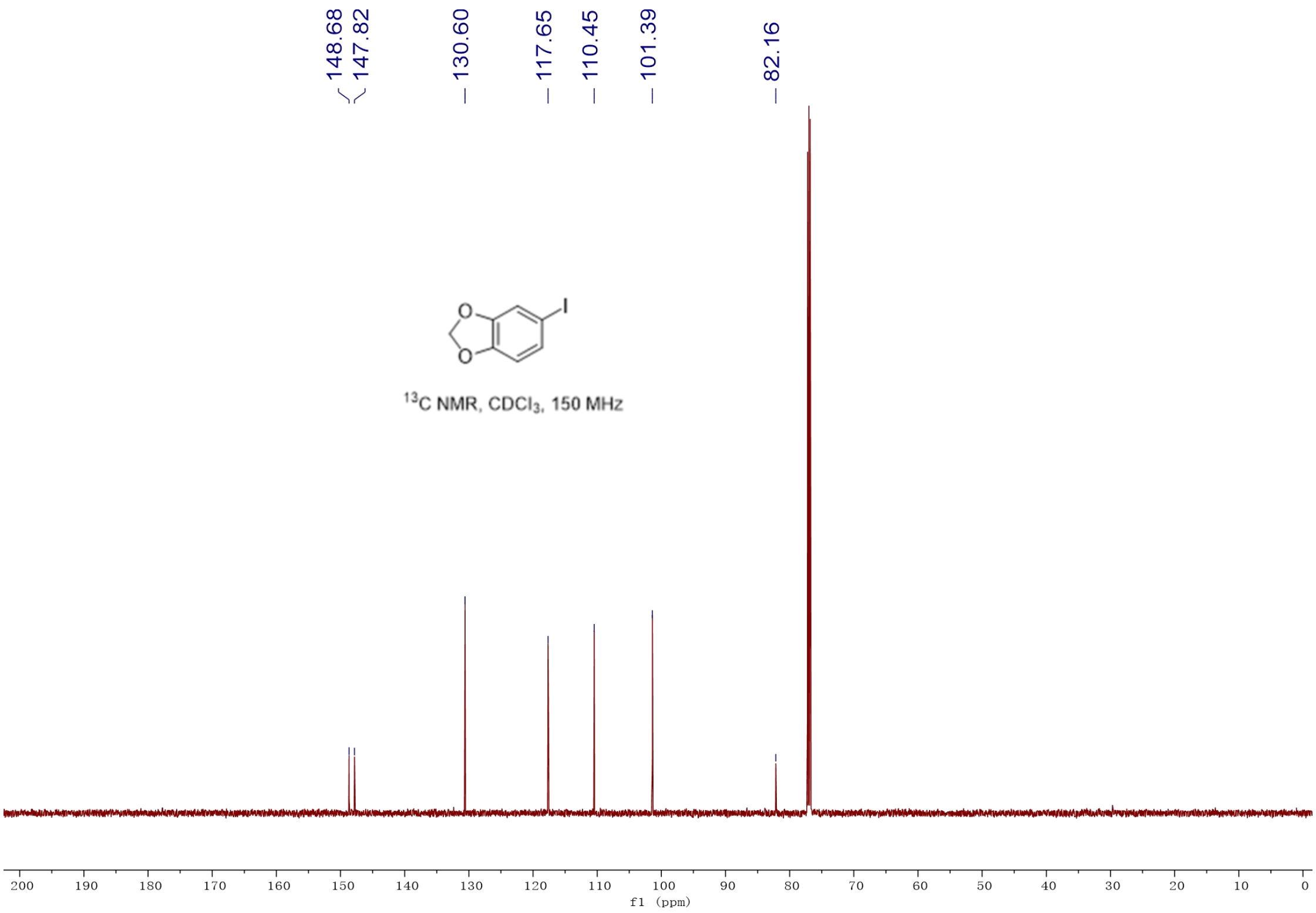

148.68
147.82
130.60
117.65
110.45
101.39
82.16
O
O
I
13C NMR, CDCl3, 150 MHz
200 190 180 170 160 150 140 130 120 110 100 90 80 70 60 50 40 30 20 10 0
f1 (ppm)

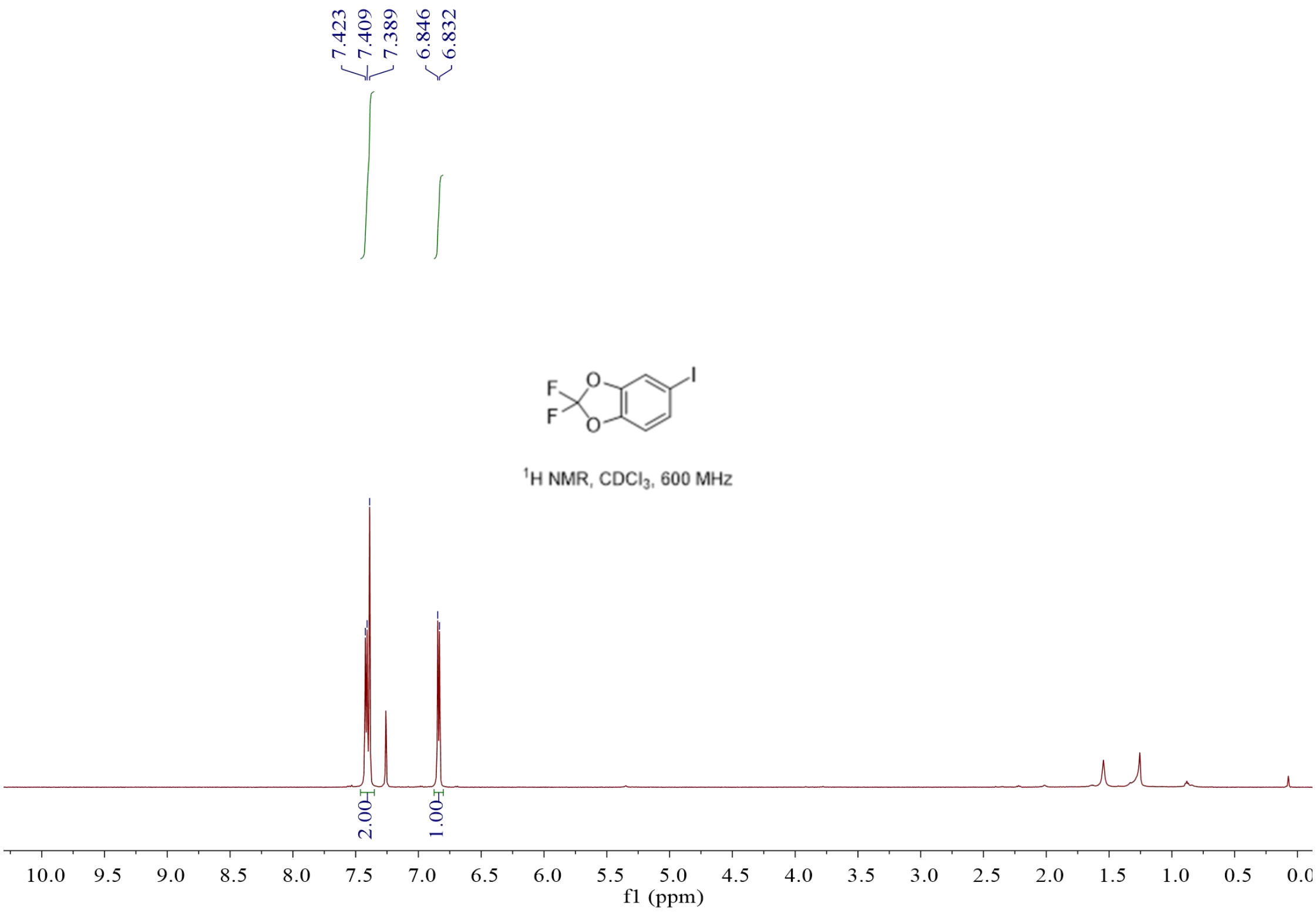

7.423
7.409
7.389
6.846
6.832
F
F
O
O
I
1H NMR, CDCl3, 600 MHz
2.00
1.00
10.0 9.5 9.0 8.5 8.0 7.5 7.0 6.5 6.0 5.5 5.0 4.5 4.0 3.5 3.0 2.5 2.0 1.5 1.0 0.5 0.0
f1 (ppm)

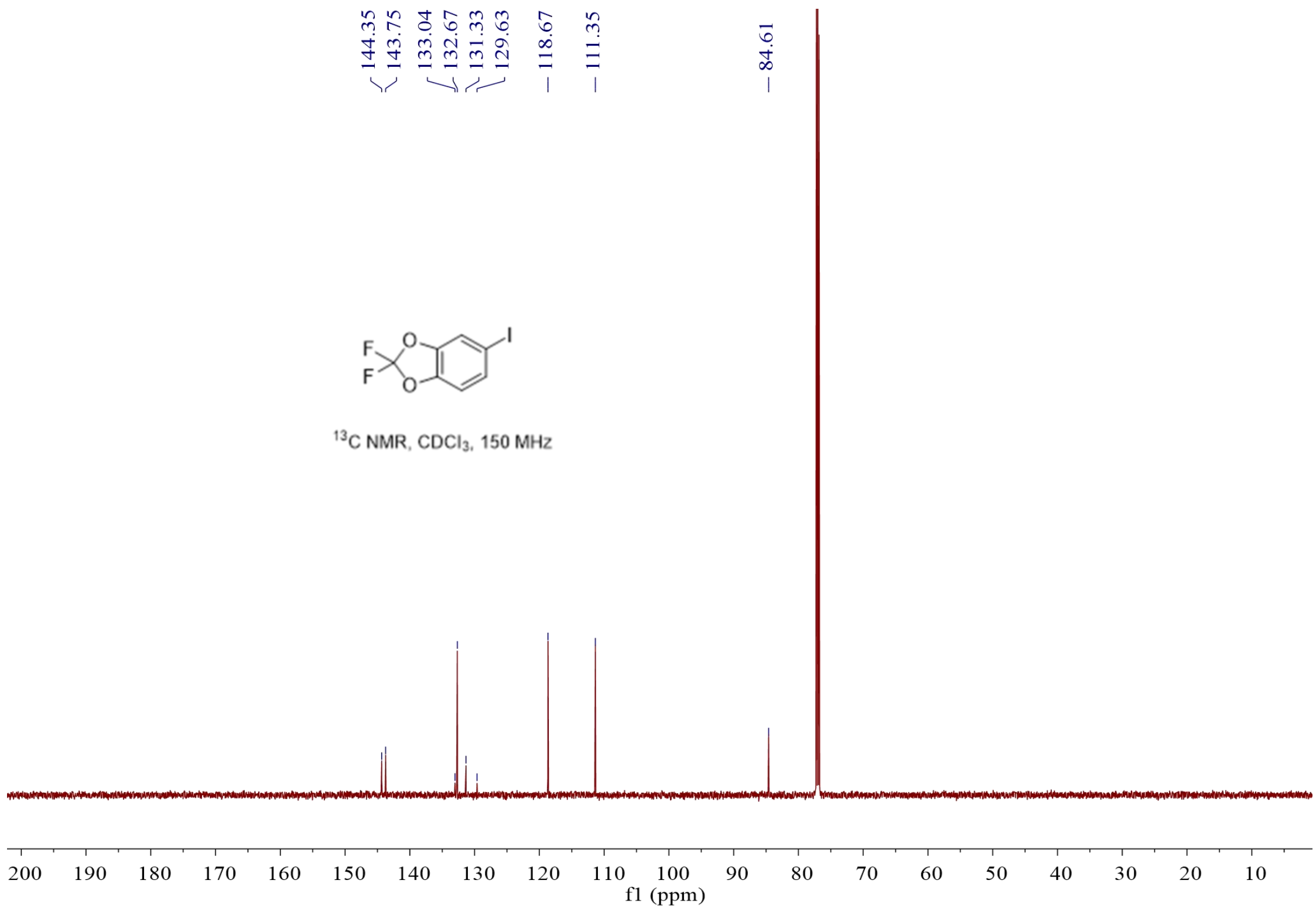
144.35
143.75
133.04
132.67
131.33
129.63
118.67
111.35
84.61
F
F
O
O
I
13C NMR, CDCl3, 150 MHz
200 190 180 170 160 150 140 130 120 110 100 90 80 70 60 50 40 30 20 10
f1 (ppm)

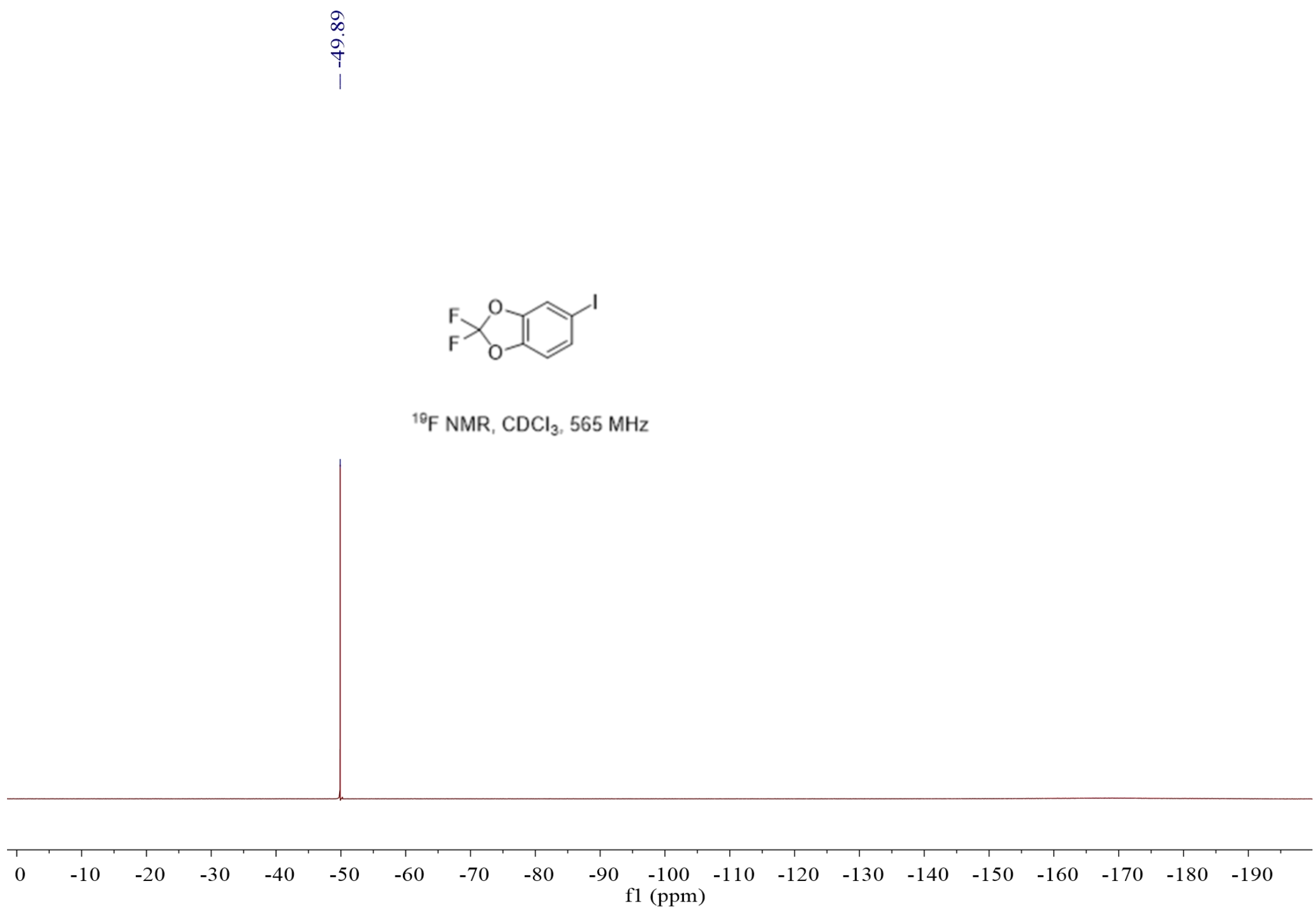
-49.89
F
F
O
O
I
19F NMR, CDCl3, 565 MHz
0 -10 -20 -30 -40 -50 -60 -70 -80 -90 -100 -110 -120 -130 -140 -150 -160 -170 -180 -190
f1 (ppm)

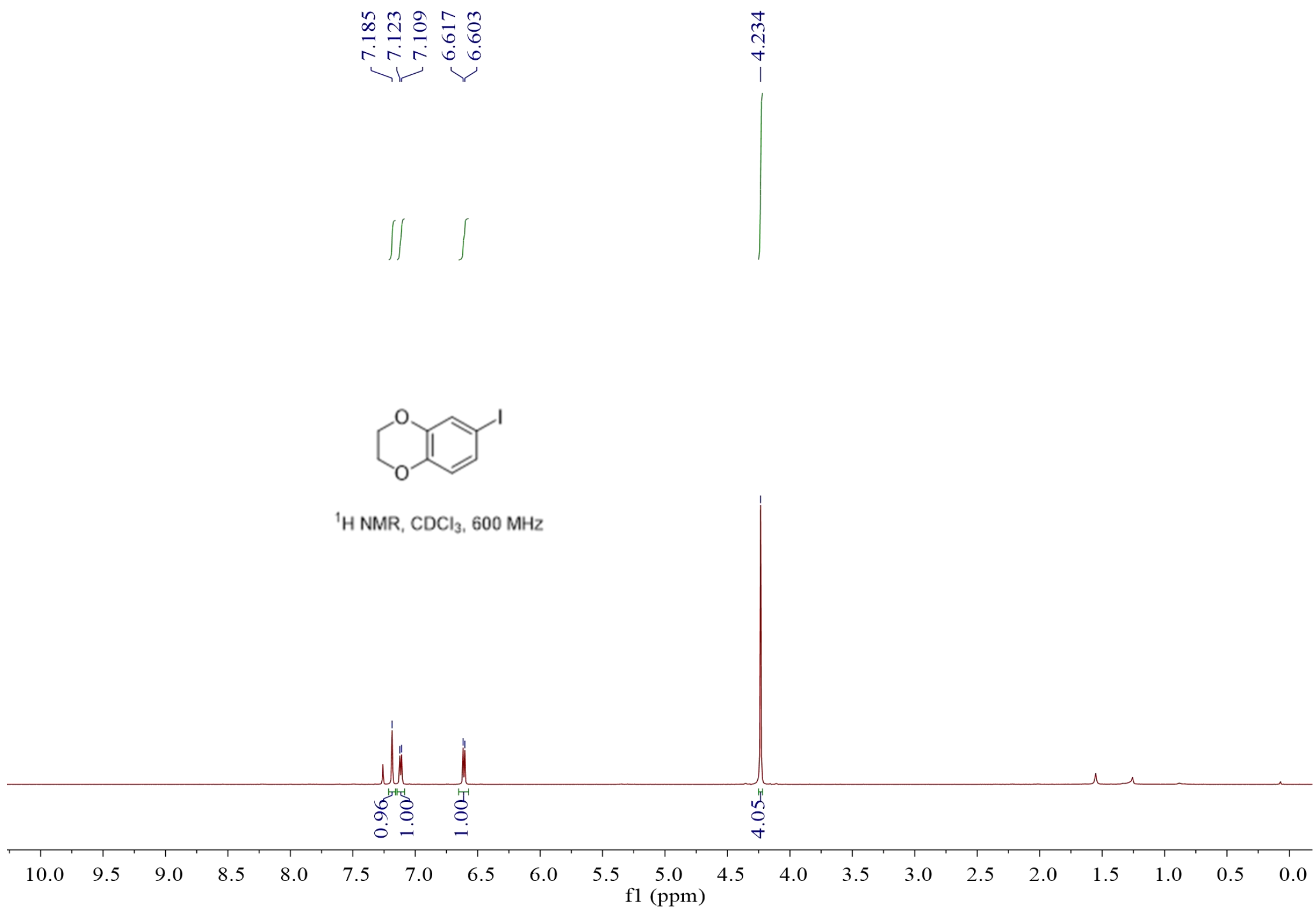
7.185
7.123
7.109
6.617
6.603
4.234
O
O
I
¹H NMR, CDCl₃, 600 MHz
0.96
1.00
1.00
4.05
10.0 9.5 9.0 8.5 8.0 7.5 7.0 6.5 6.0 5.5 5.0 4.5 4.0 3.5 3.0 2.5 2.0 1.5 1.0 0.5 0.0
f1 (ppm)

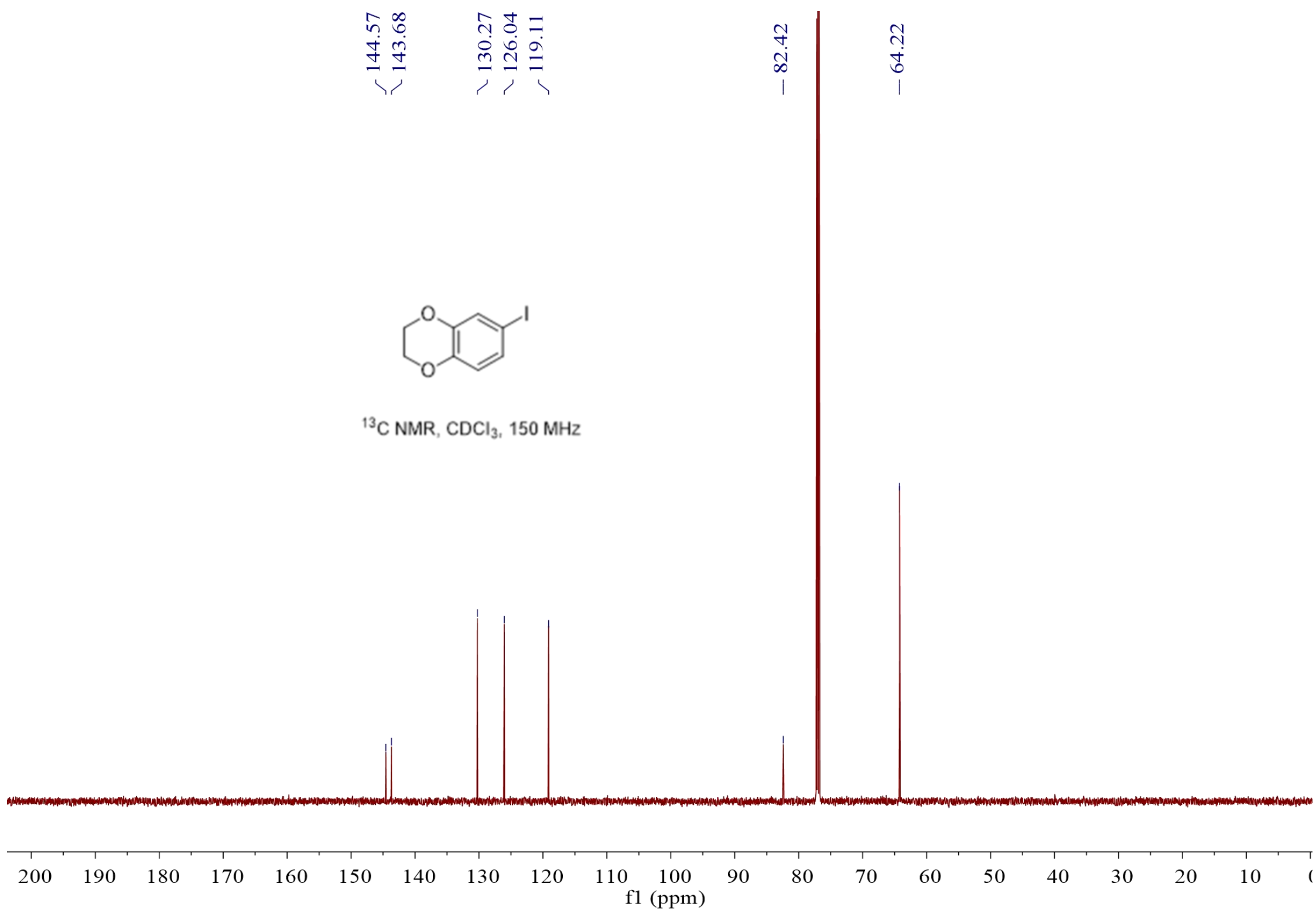
144.57
143.68
130.27
126.04
119.11
82.42
64.22
O
O
I
¹³C NMR, CDCl₃, 150 MHz
200 190 180 170 160 150 140 130 120 110 100 90 80 70 60 50 40 30 20 10
f1 (ppm)

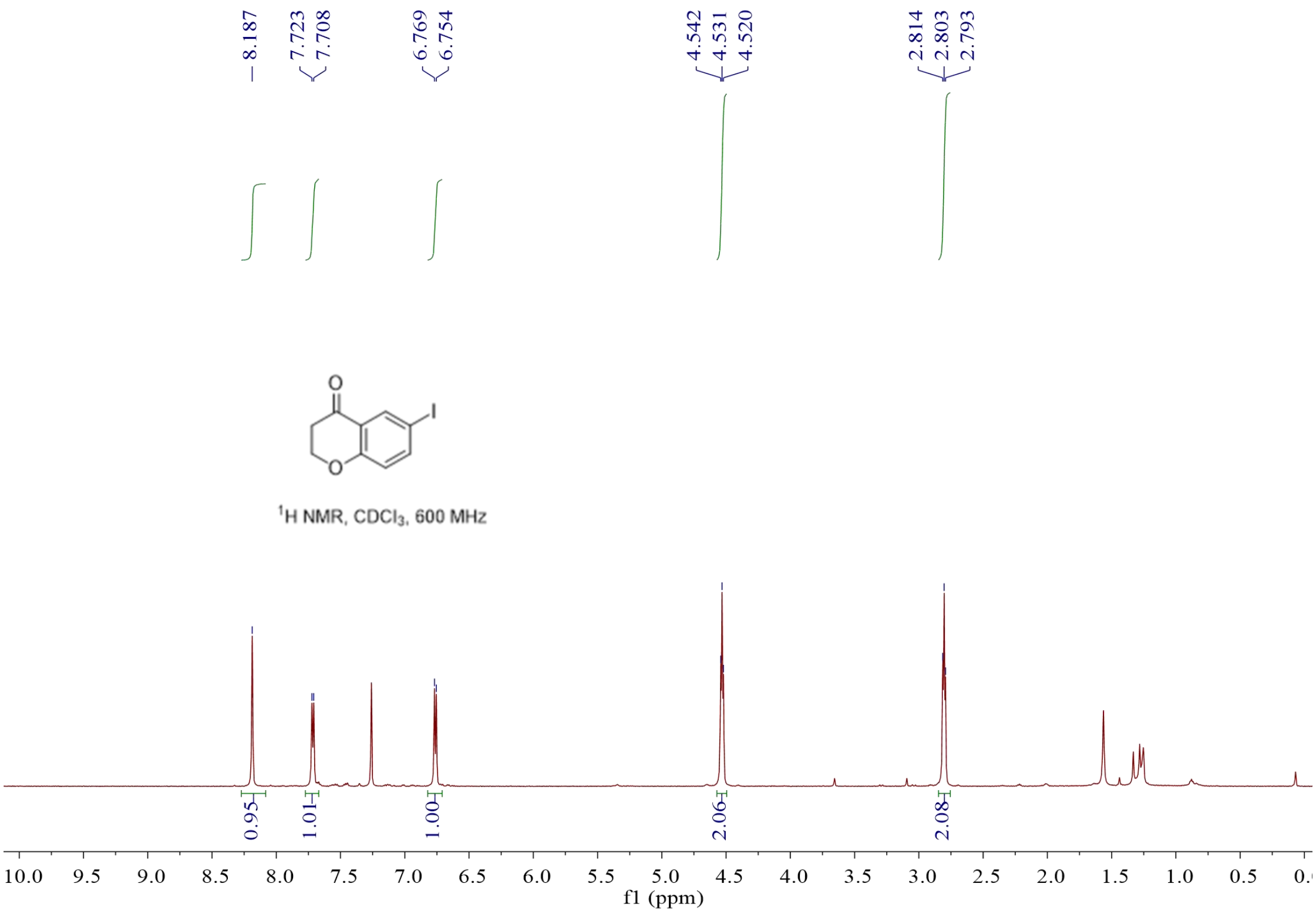

8.187
7.723
7.708
6.769
6.754
4.542
4.531
4.520
2.814
2.803
2.793
O
I
O
¹H NMR, CDCl3, 600 MHz
0.95
1.01
1.00
2.06
2.08
f1 (ppm)

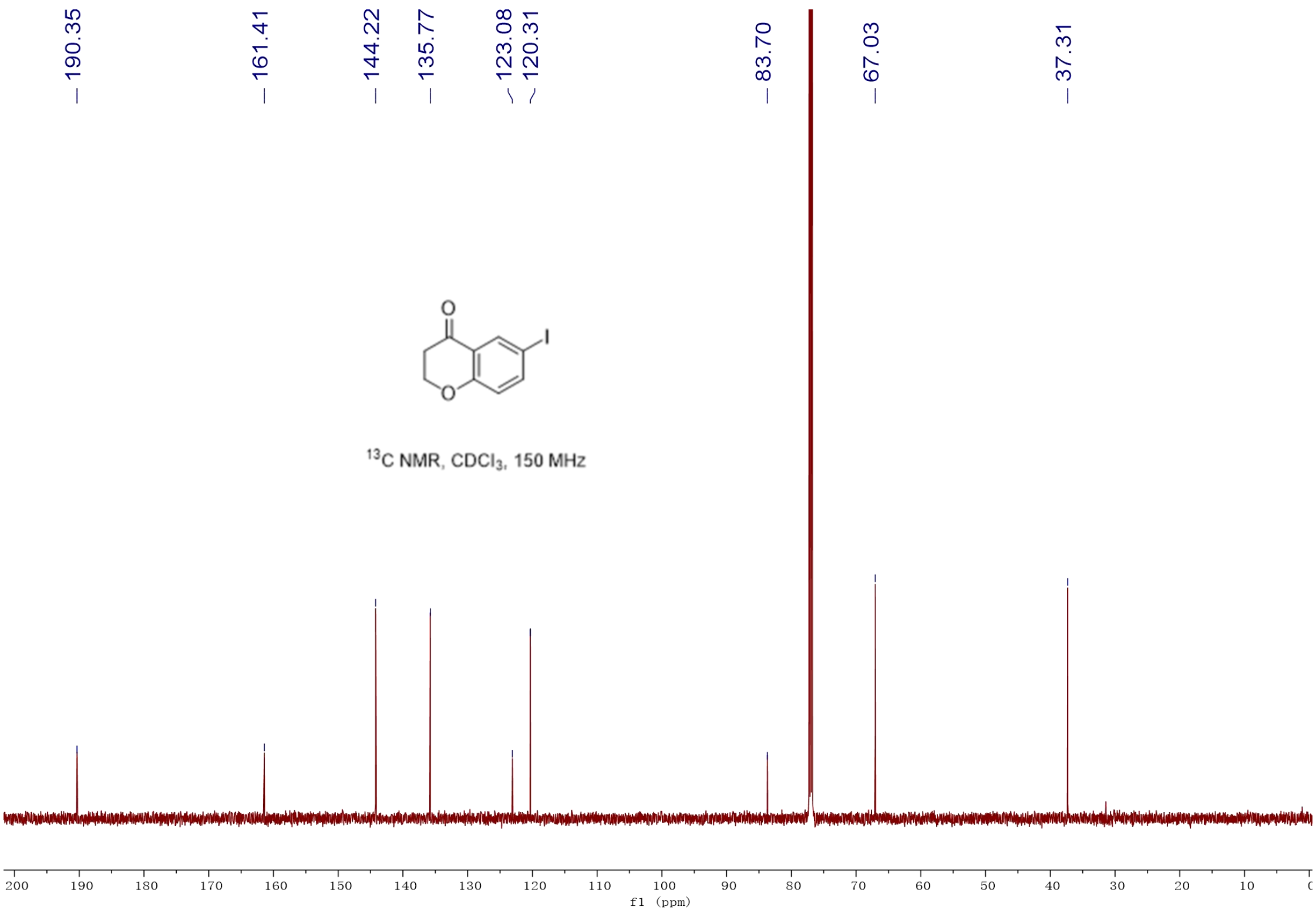

190.35
161.41
144.22
135.77
123.08
120.31
83.70
67.03
37.31
O
I
O
¹³C NMR, CDCl3, 150 MHz
f1 (ppm)

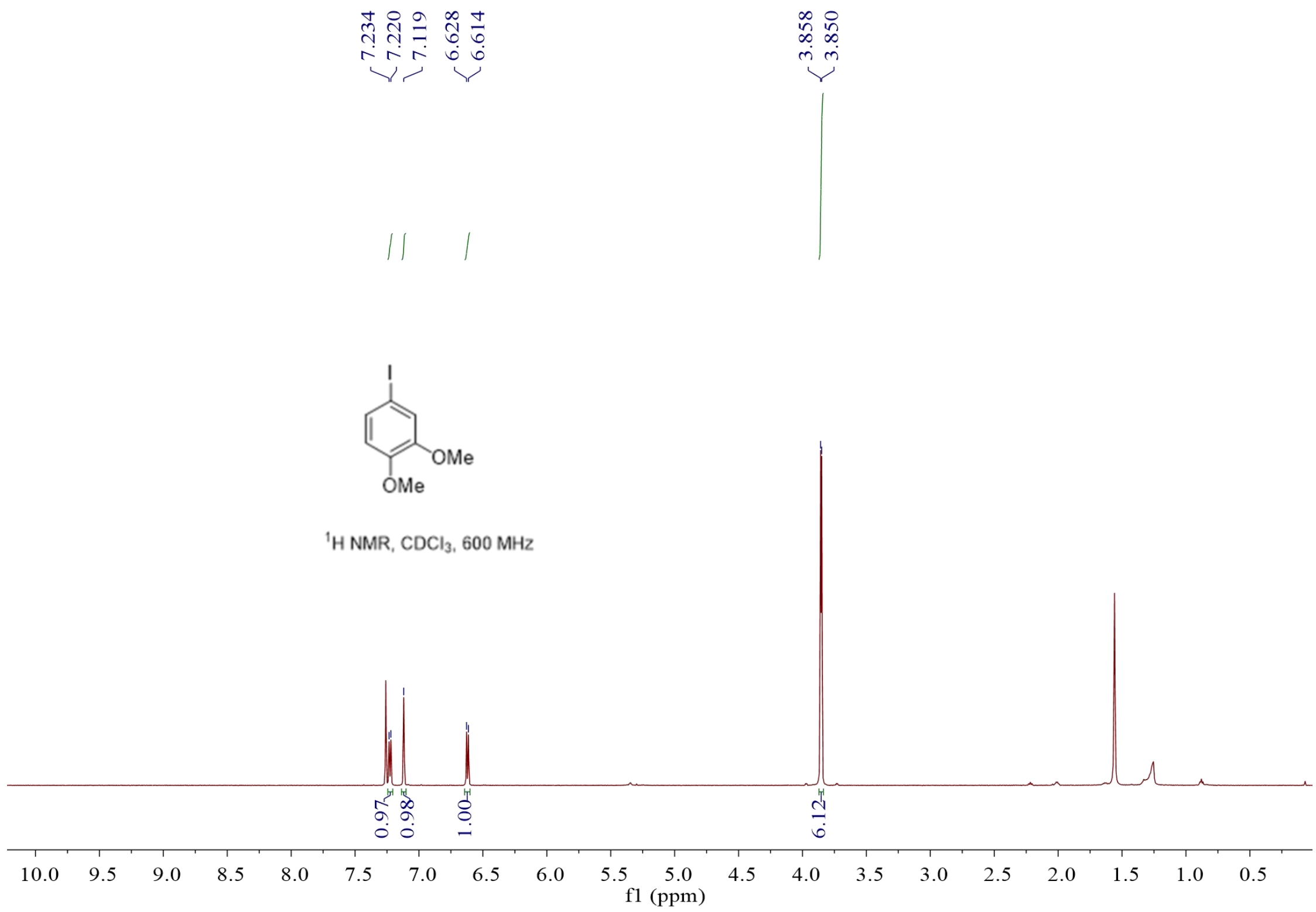
7.234
7.220
7.119
6.628
6.614
3.858
3.850
I
OMe
OMe
¹H NMR, CDCl₃, 600 MHz
0.97
0.98
1.00
6.12
10.0 9.5 9.0 8.5 8.0 7.5 7.0 6.5 6.0 5.5 5.0 4.5 4.0 3.5 3.0 2.5 2.0 1.5 1.0 0.5
f1 (ppm)

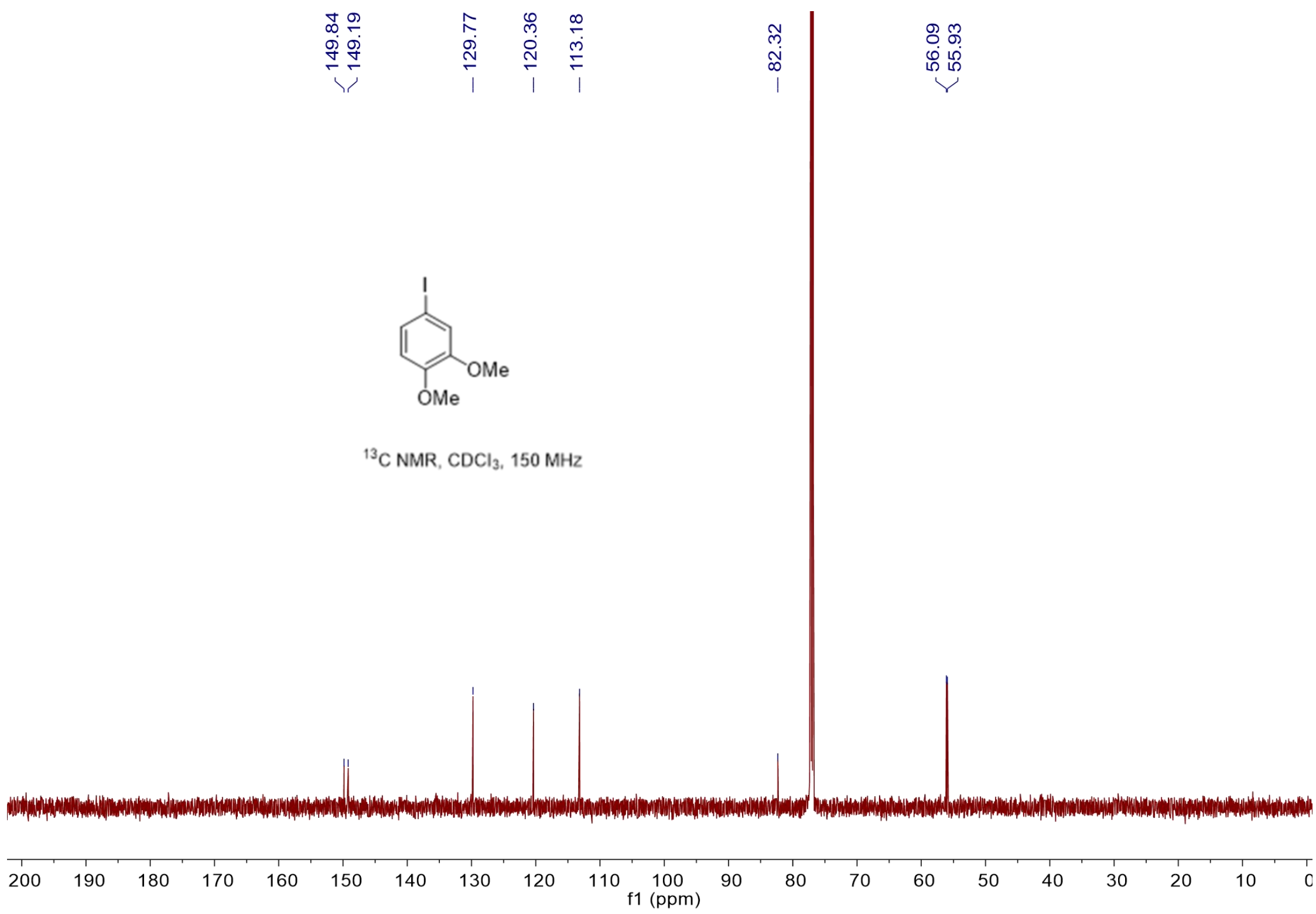
149.84
149.19
129.77
120.36
113.18
82.32
56.09
55.93
I
OMe
OMe
¹³C NMR, CDCl₃, 150 MHz
200 190 180 170 160 150 140 130 120 110 100 90 80 70 60 50 40 30 20 10 0
f1 (ppm)

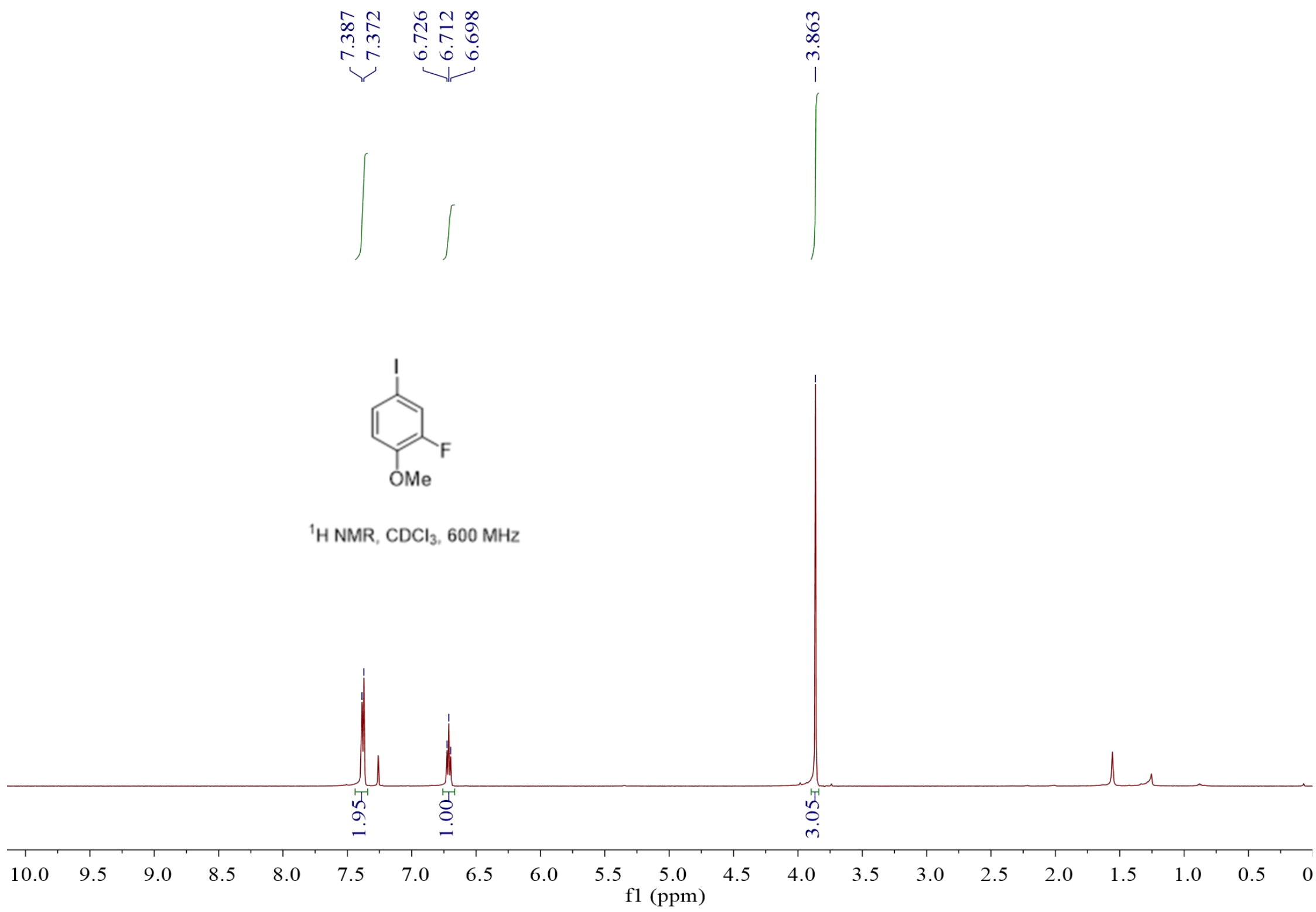
7.387
7.372
6.726
6.712
6.698
3.863
OMe
F
I
¹H NMR, CDCl₃, 600 MHz
1.95
1.00
3.05
10.0 9.5 9.0 8.5 8.0 7.5 7.0 6.5 6.0 5.5 5.0 4.5 4.0 3.5 3.0 2.5 2.0 1.5 1.0 0.5 0
f1 (ppm)

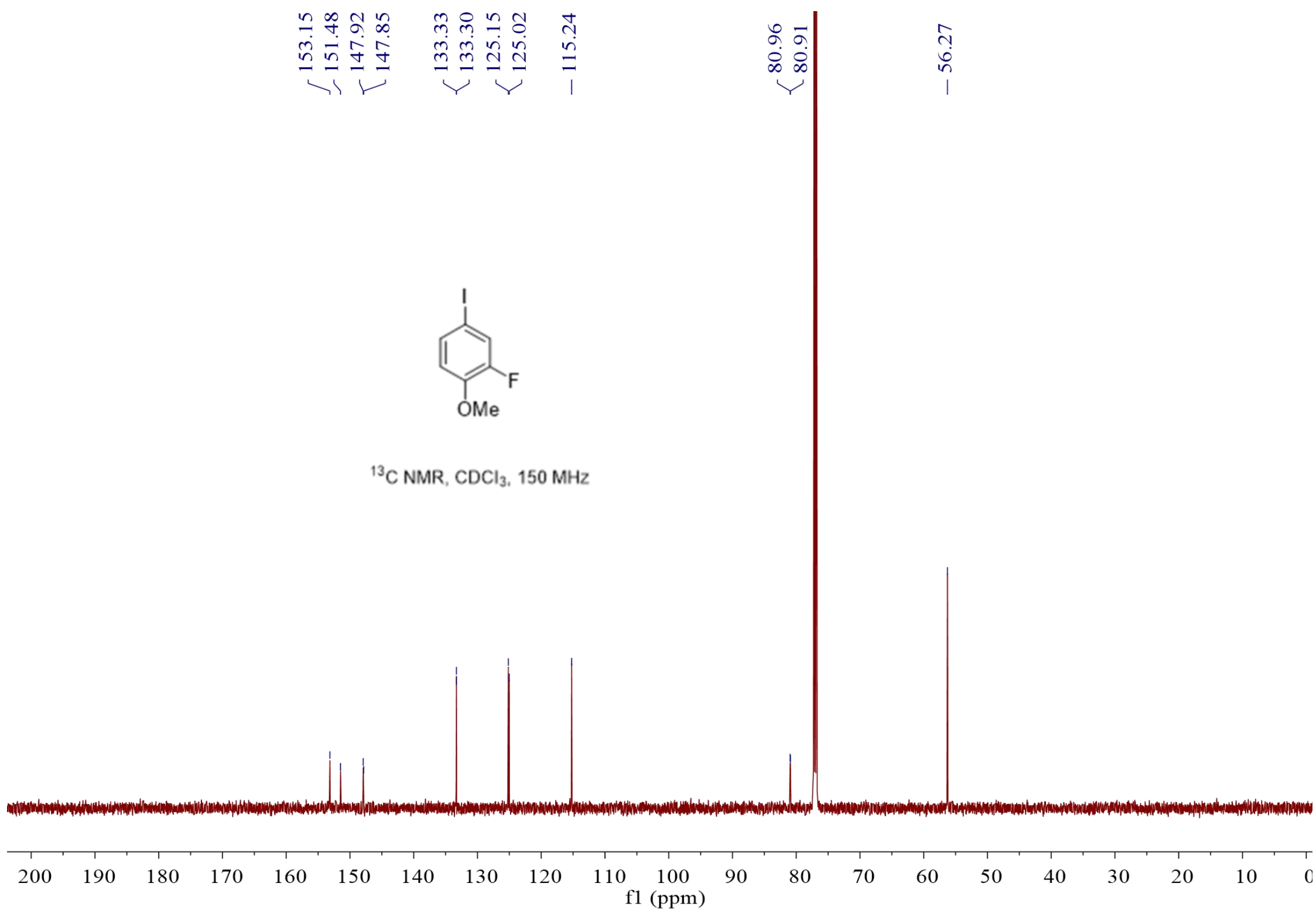
153.15
151.48
147.92
147.85
133.33
133.30
125.15
125.02
115.24
80.96
80.91
56.27
OMe
F
I
¹³C NMR, CDCl₃, 150 MHz
200 190 180 170 160 150 140 130 120 110 100 90 80 70 60 50 40 30 20 10 0
f1 (ppm)

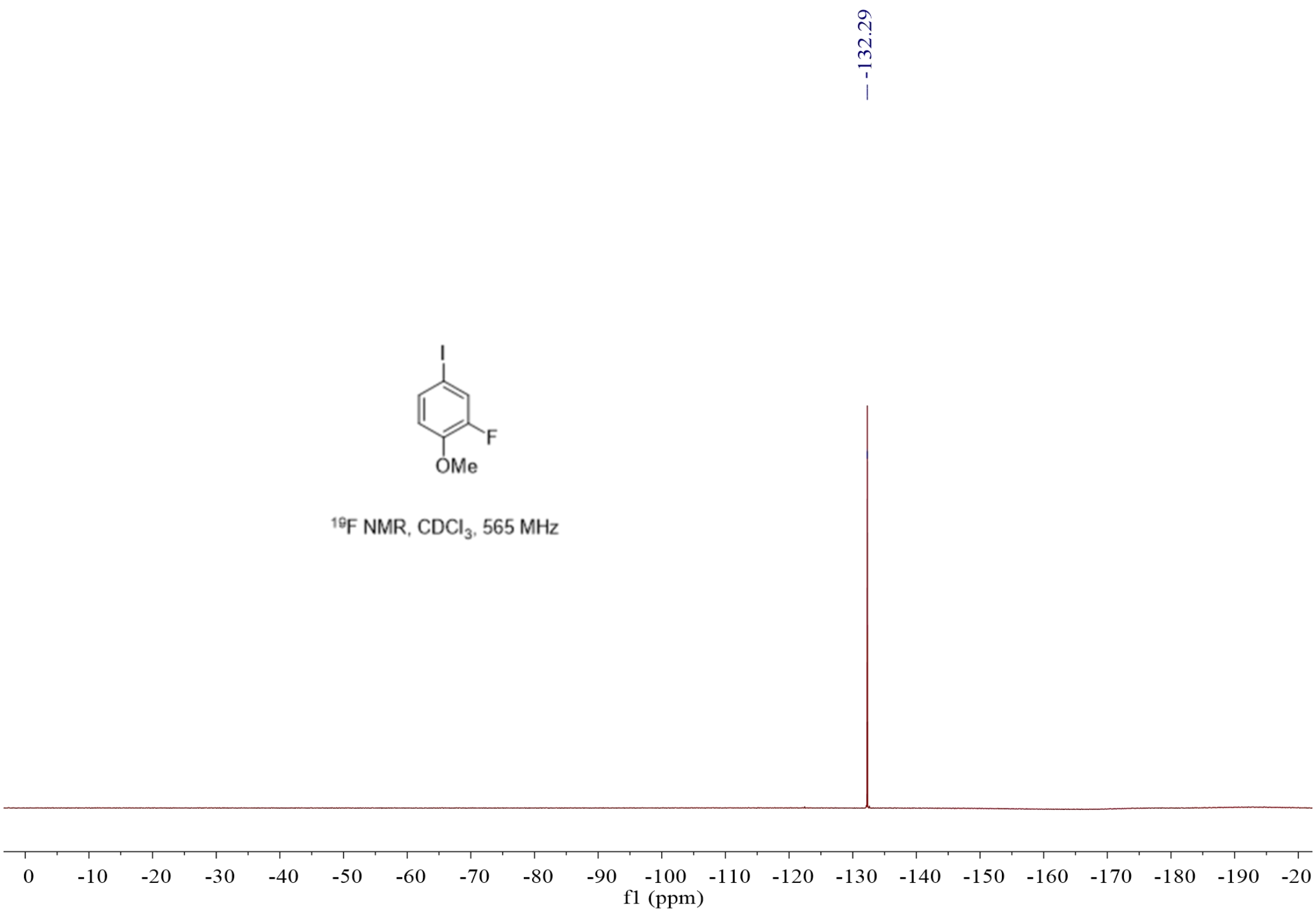

-132.29
I
F
OMe
19F NMR, CDCl3, 565 MHz
0 -10 -20 -30 -40 -50 -60 -70 -80 -90 -100 -110 -120 -130 -140 -150 -160 -170 -180 -190 -20
f1 (ppm)

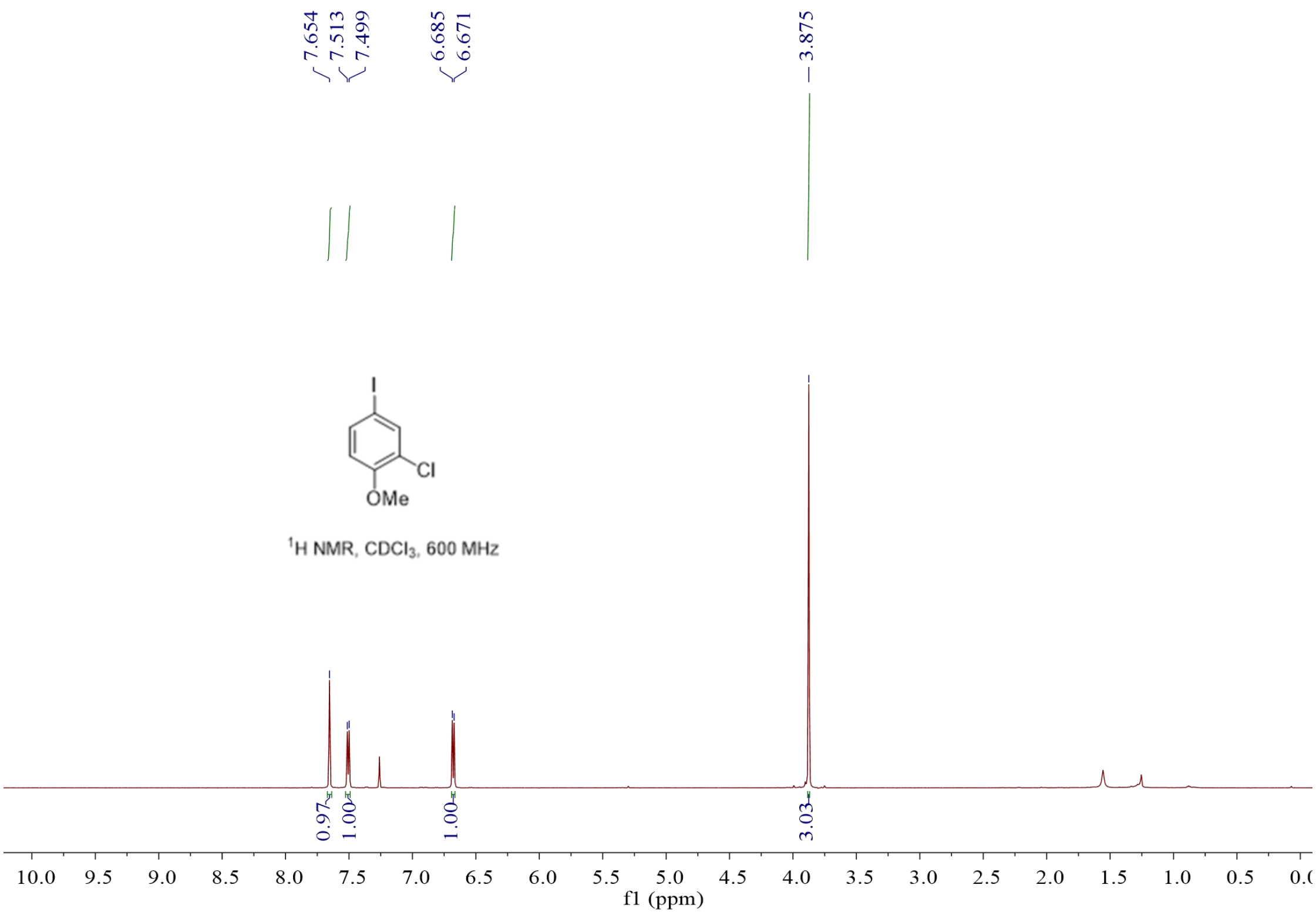

7.654
7.513
7.499
6.685
6.671
3.875
I
Cl
OMe
1H NMR, CDCl3, 600 MHz
0.97
1.00
1.00
3.03
10.0 9.5 9.0 8.5 8.0 7.5 7.0 6.5 6.0 5.5 5.0 4.5 4.0 3.5 3.0 2.5 2.0 1.5 1.0 0.5 0.0
f1 (ppm)

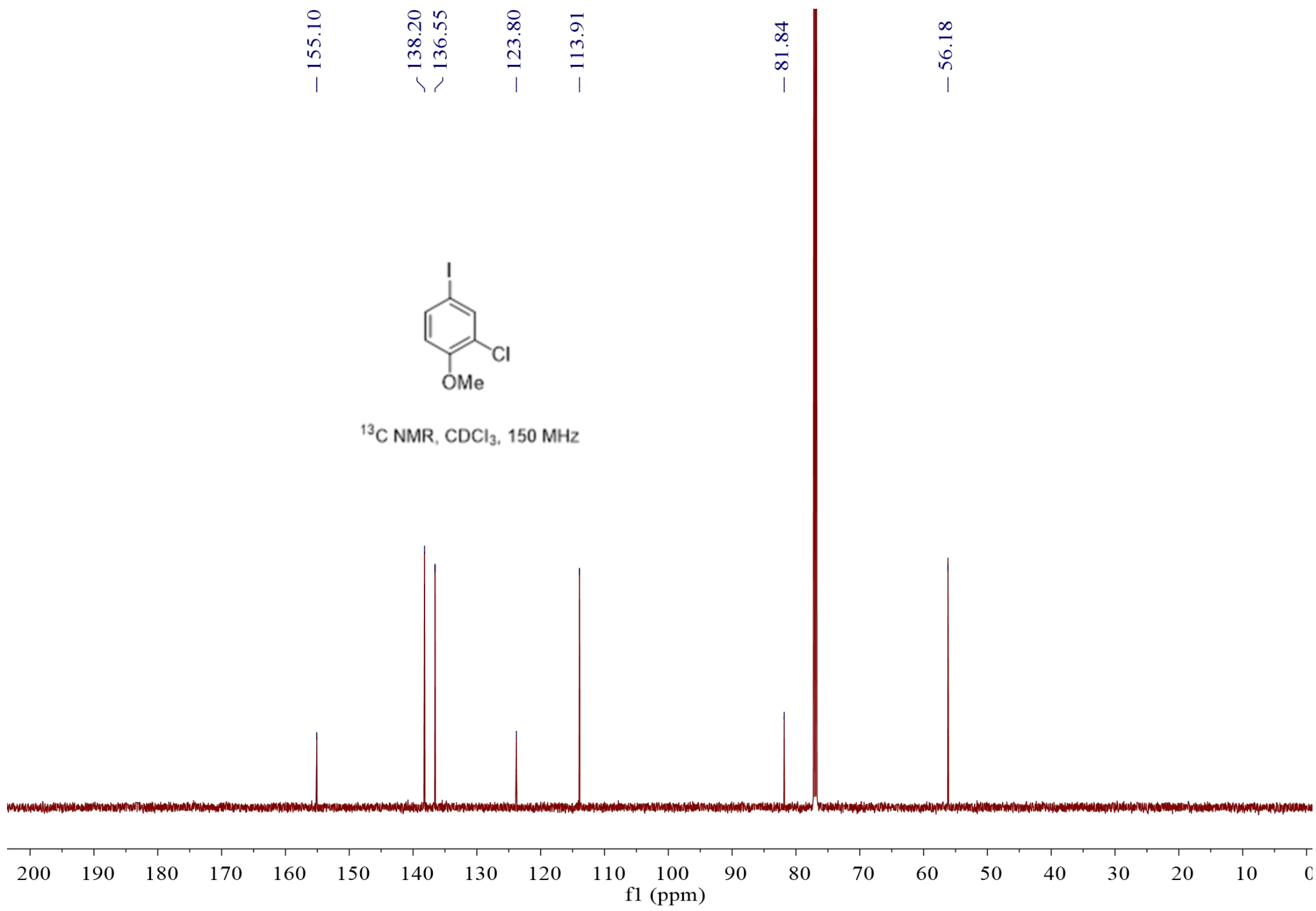
155.10
138.20
136.55
123.80
113.91
81.84
56.18
I
Cl
OMe
13C NMR, CDCl3, 150 MHz
200 190 180 170 160 150 140 130 120 110 100 90 80 70 60 50 40 30 20 10 0
f1 (ppm)

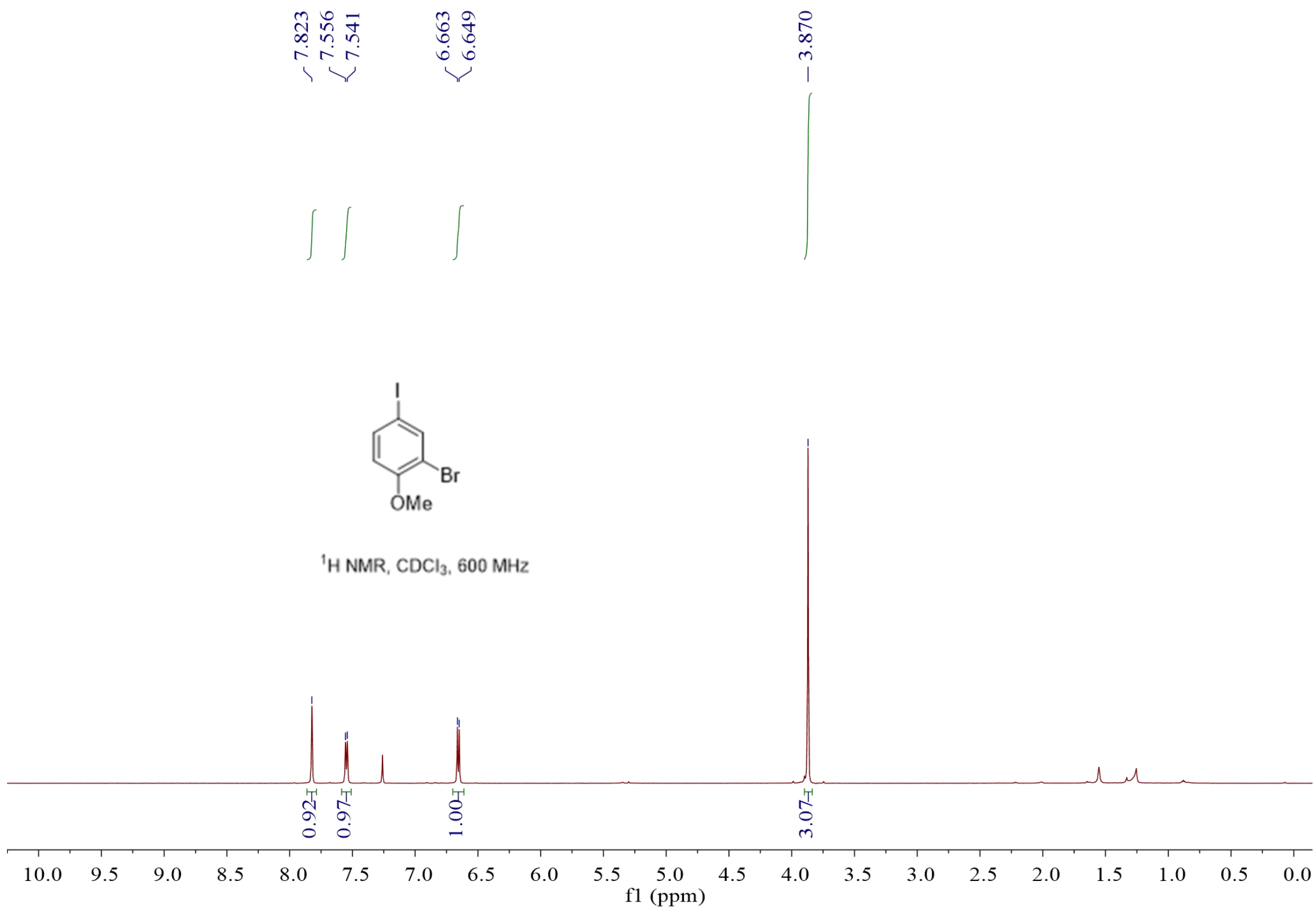
7.823
7.556
7.541
6.663
6.649
3.870
I
Br
OMe
1H NMR, CDCl3, 600 MHz
0.92
0.97
1.00
3.07
10.0 9.5 9.0 8.5 8.0 7.5 7.0 6.5 6.0 5.5 5.0 4.5 4.0 3.5 3.0 2.5 2.0 1.5 1.0 0.5 0.0
f1 (ppm)

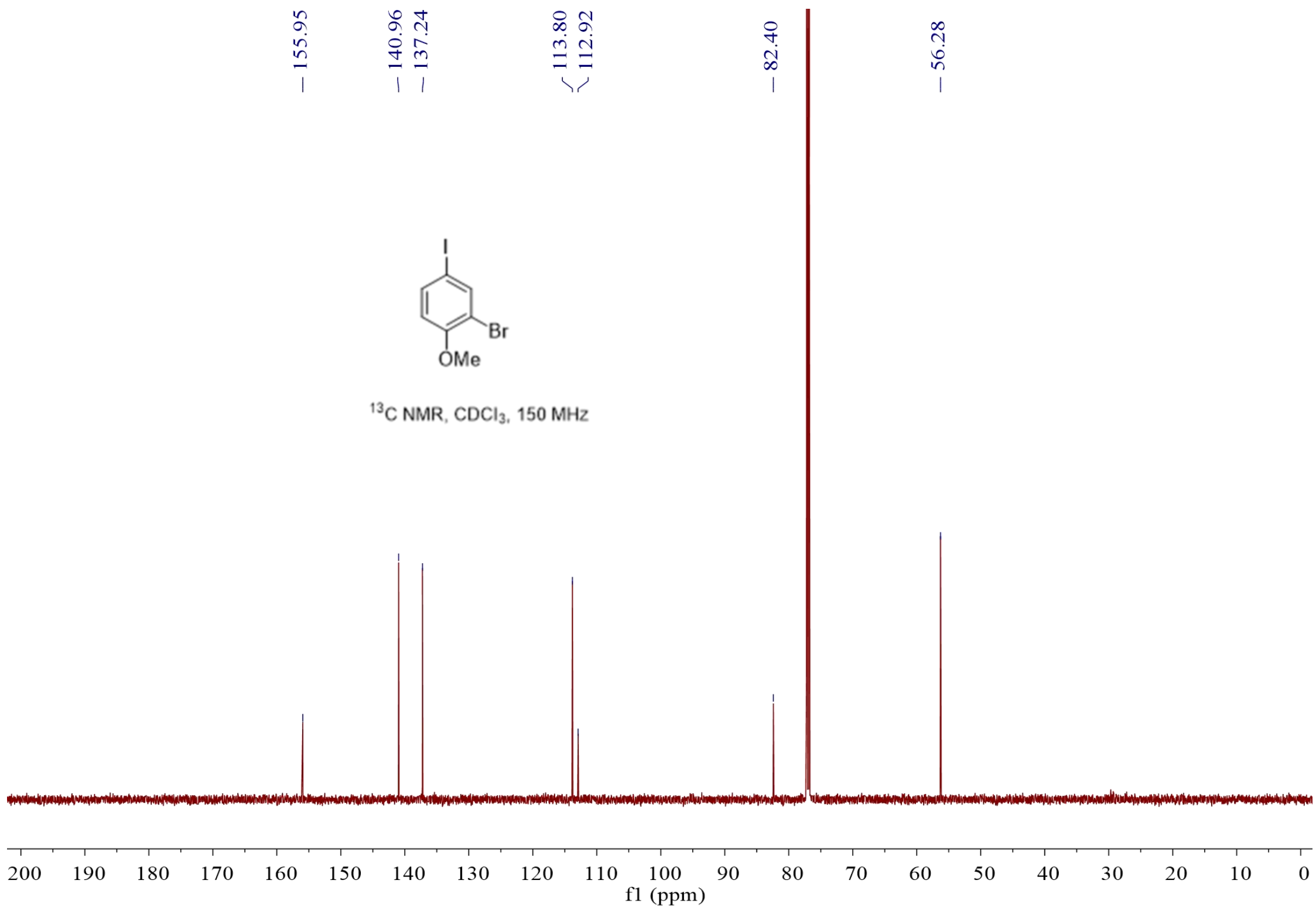
155.95
140.96
137.24
113.80
112.92
82.40
56.28
I
Br
OMe
13C NMR, CDCl3, 150 MHz
200 190 180 170 160 150 140 130 120 110 100 90 80 70 60 50 40 30 20 10 0
f1 (ppm)

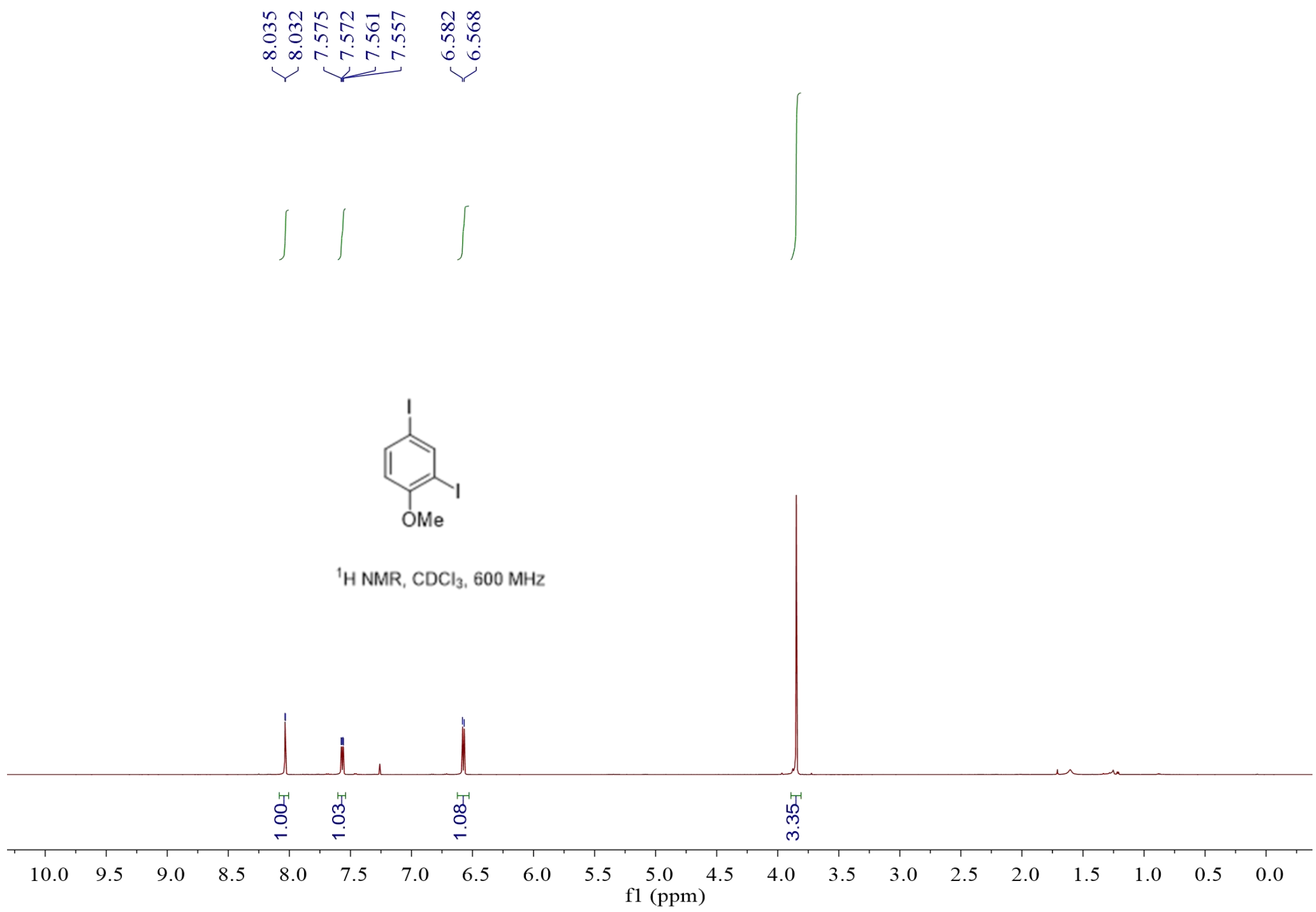
8.035
8.032
7.575
7.572
7.561
7.557
6.582
6.568
I
I
OMe
1H NMR, CDCl3, 600 MHz
1.00
1.03
1.08
3.35
10.0 9.5 9.0 8.5 8.0 7.5 7.0 6.5 6.0 5.5 5.0 4.5 4.0 3.5 3.0 2.5 2.0 1.5 1.0 0.5 0.0
f1 (ppm)

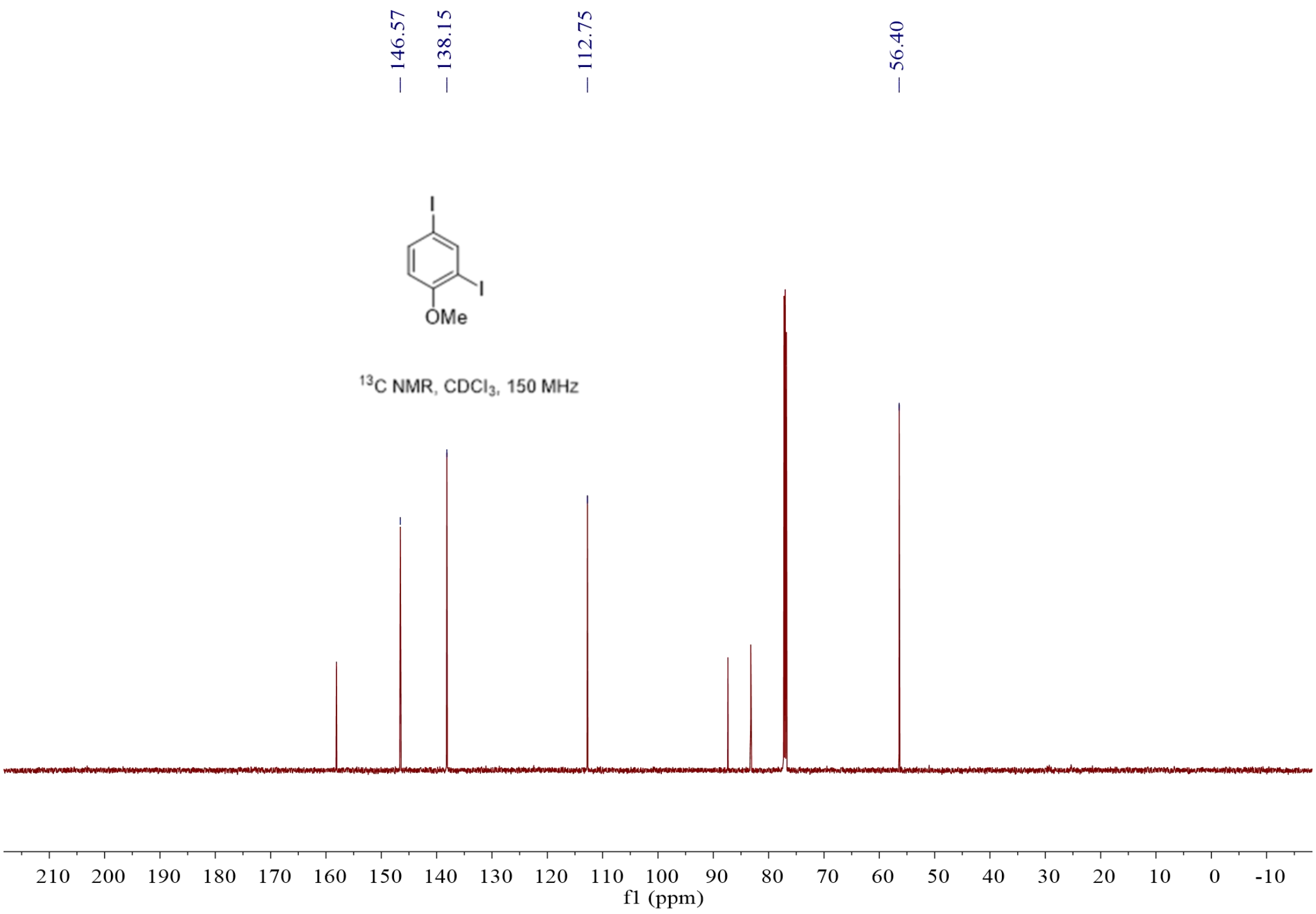
146.57
138.15
112.75
56.40
I
I
OMe
^{13}C NMR, $CDCl_3$, 150 MHz
210 200 190 180 170 160 150 140 130 120 110 100 90 80 70 60 50 40 30 20 10 0 -10
f1 (ppm)

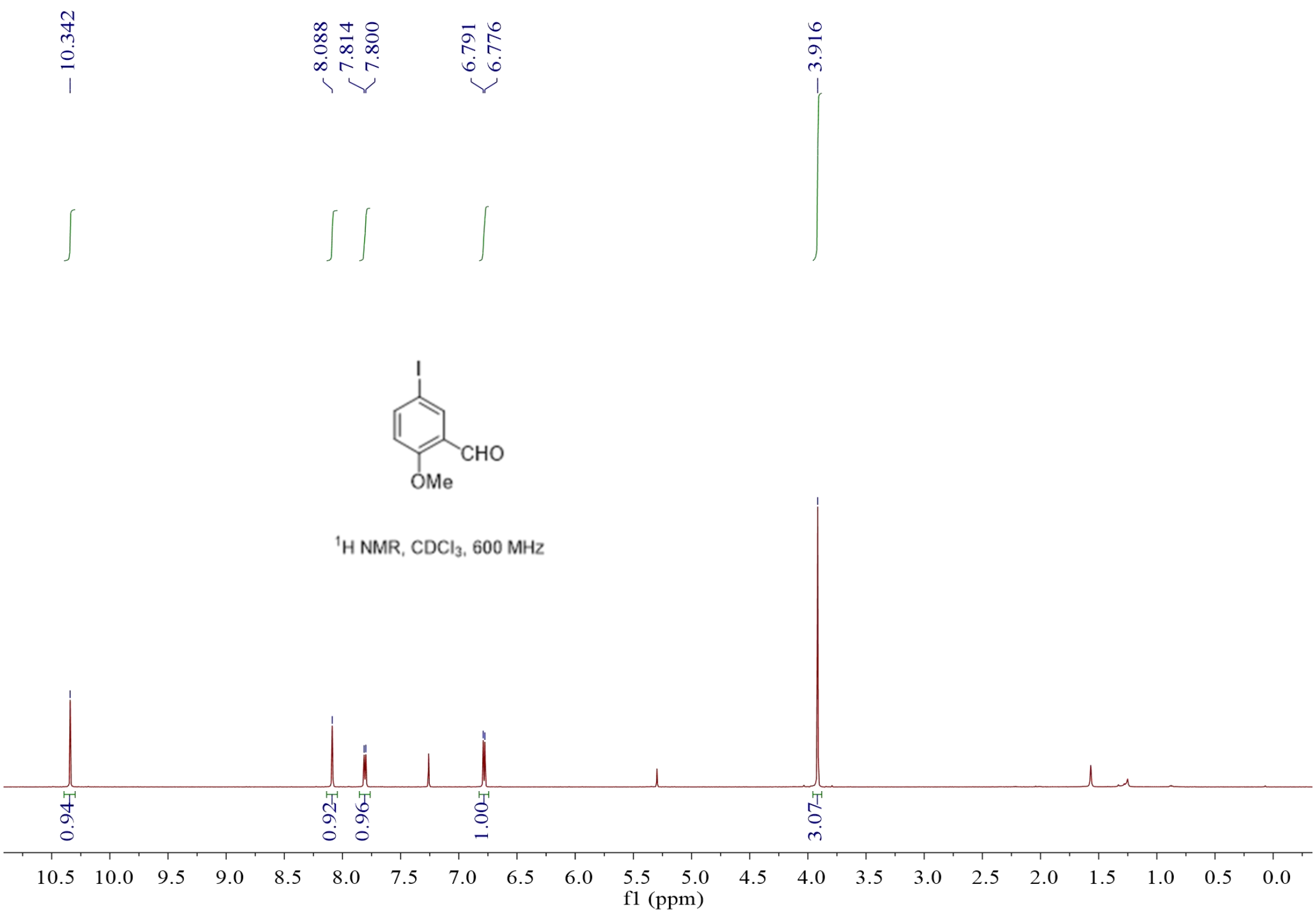
10.342
8.088
7.814
7.800
6.791
6.776
3.916
I
CHO
OMe
^{1}H NMR, $CDCl_3$, 600 MHz
0.94
0.92
0.96
1.00
3.07
10.5 10.0 9.5 9.0 8.5 8.0 7.5 7.0 6.5 6.0 5.5 5.0 4.5 4.0 3.5 3.0 2.5 2.0 1.5 1.0 0.5 0.0
f1 (ppm)

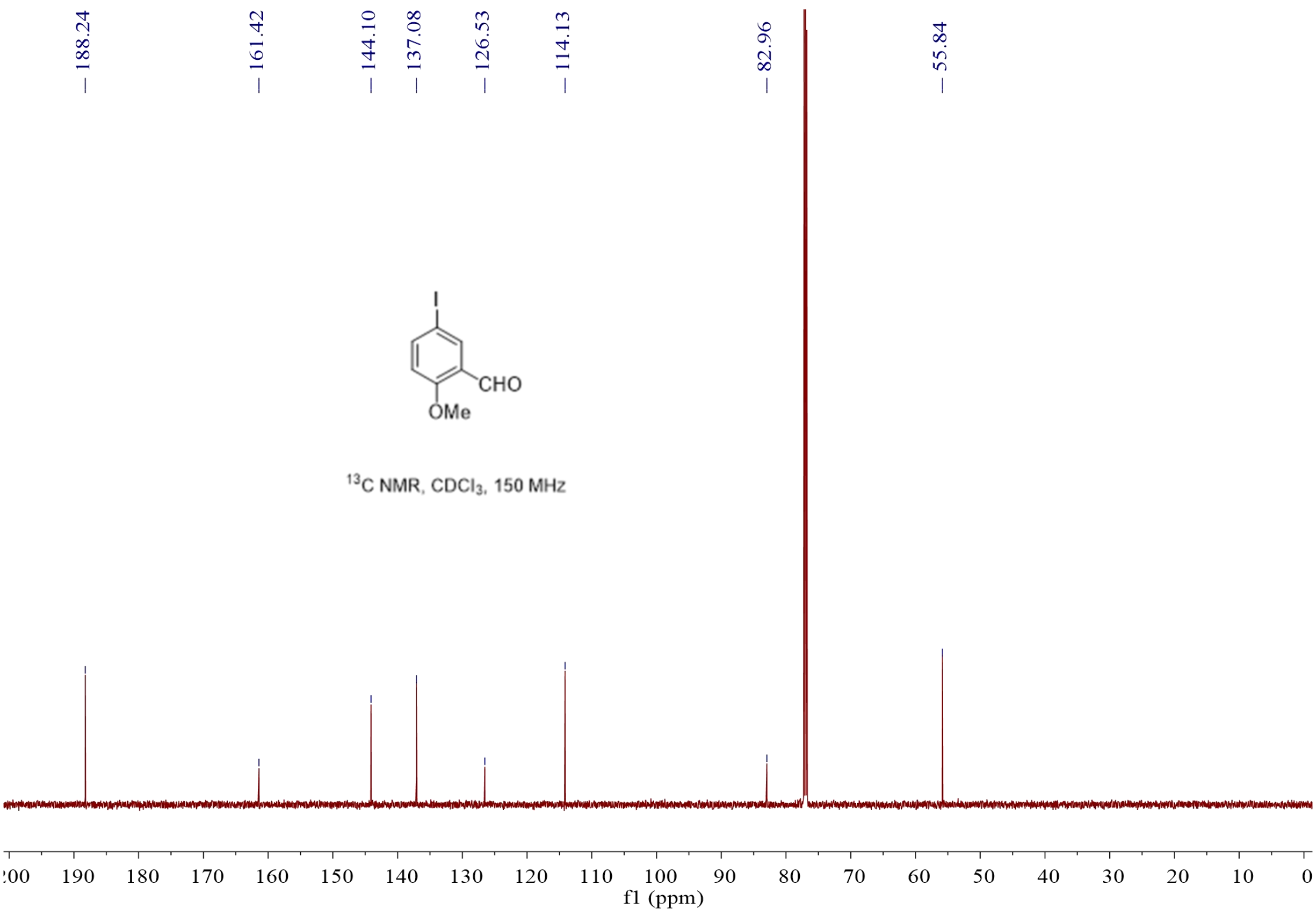
188.24
161.42
144.10
137.08
126.53
114.13
82.96
55.84
I
CHO
OMe
13C NMR, CDCl3, 150 MHz
f1 (ppm)

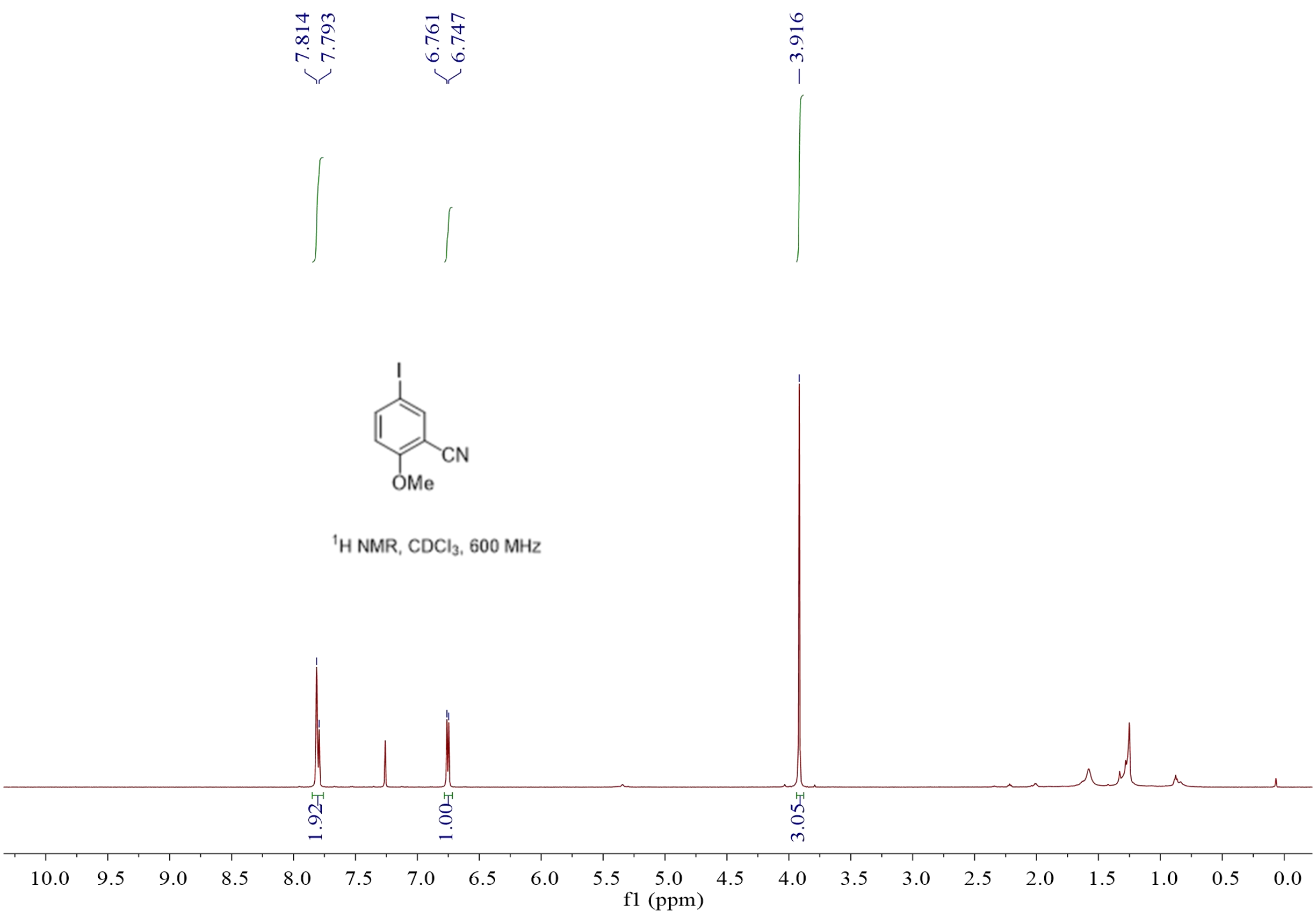
7.814
7.793
6.761
6.747
3.916
I
CN
OMe
1H NMR, CDCl3, 600 MHz
1.92
1.00
3.05
f1 (ppm)

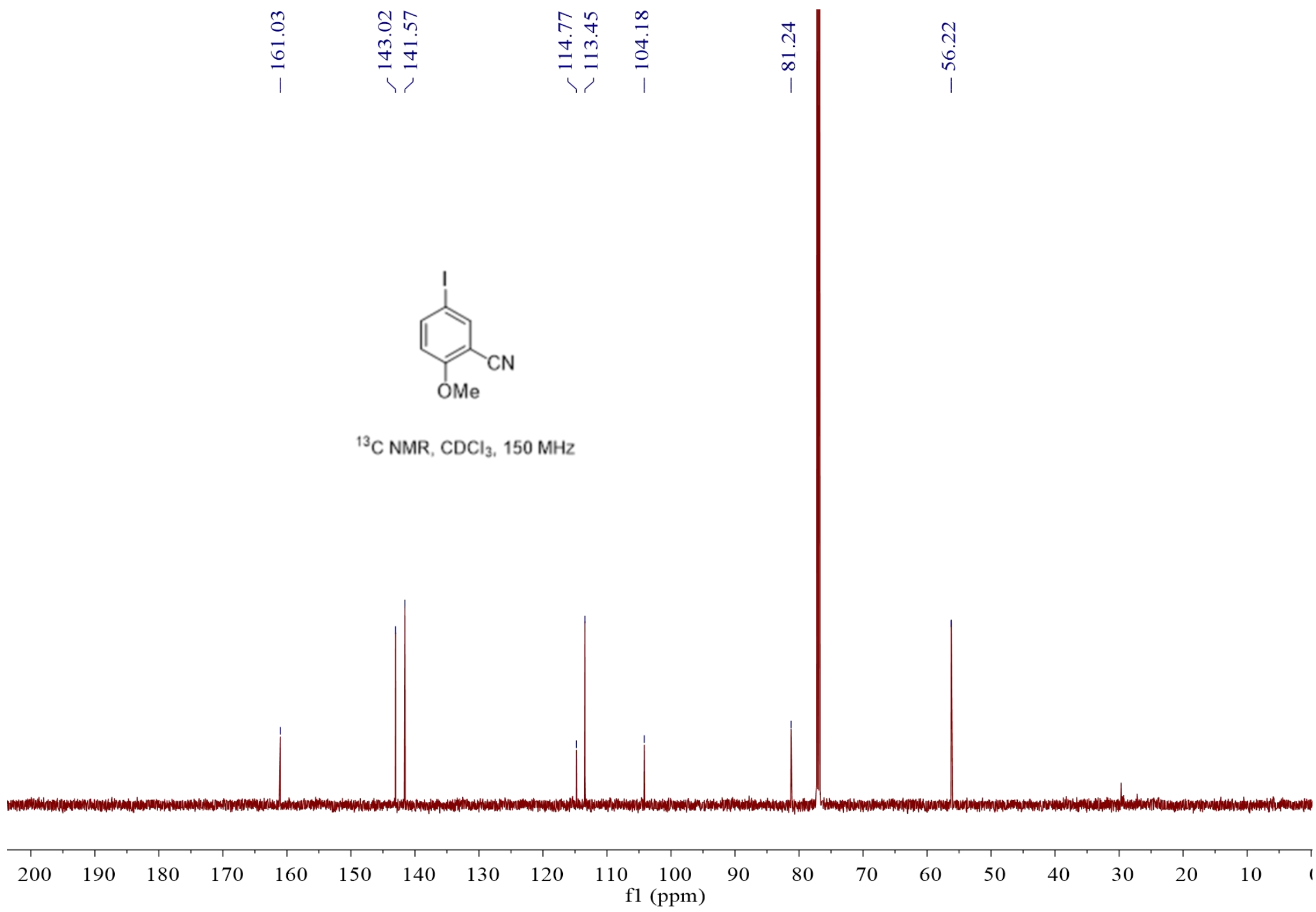

161.03
143.02
141.57
114.77
113.45
104.18
81.24
56.22
I
CN
OMe
13C NMR, CDCl3, 150 MHz
200 190 180 170 160 150 140 130 120 110 100 90 80 70 60 50 40 30 20 10
f1 (ppm)

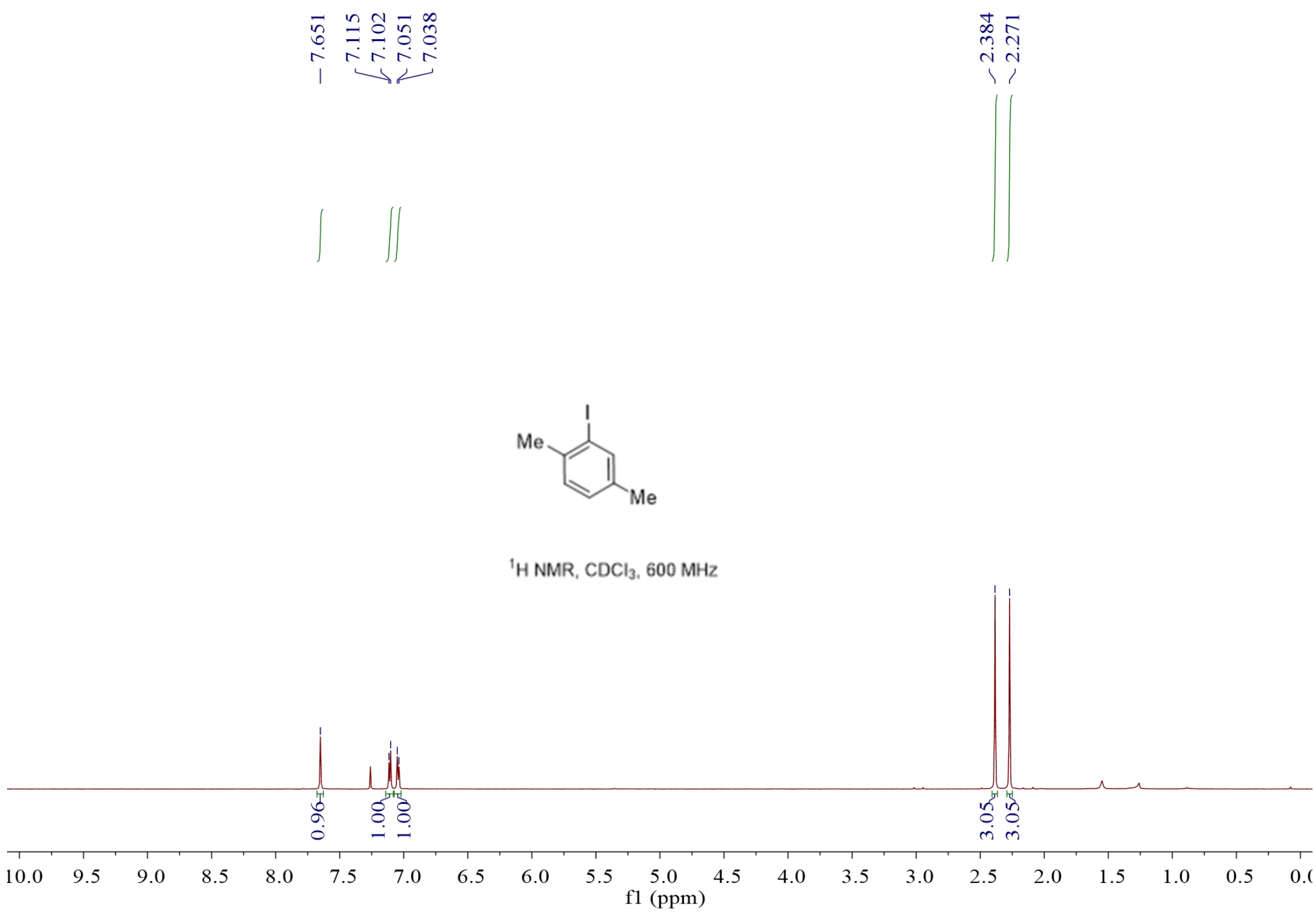

7.651
7.115
7.102
7.051
7.038
2.384
2.271
I
Me
Me
1H NMR, CDCl3, 600 MHz
0.96
1.00
1.00
3.05
3.05
10.0 9.5 9.0 8.5 8.0 7.5 7.0 6.5 6.0 5.5 5.0 4.5 4.0 3.5 3.0 2.5 2.0 1.5 1.0 0.5
f1 (ppm)

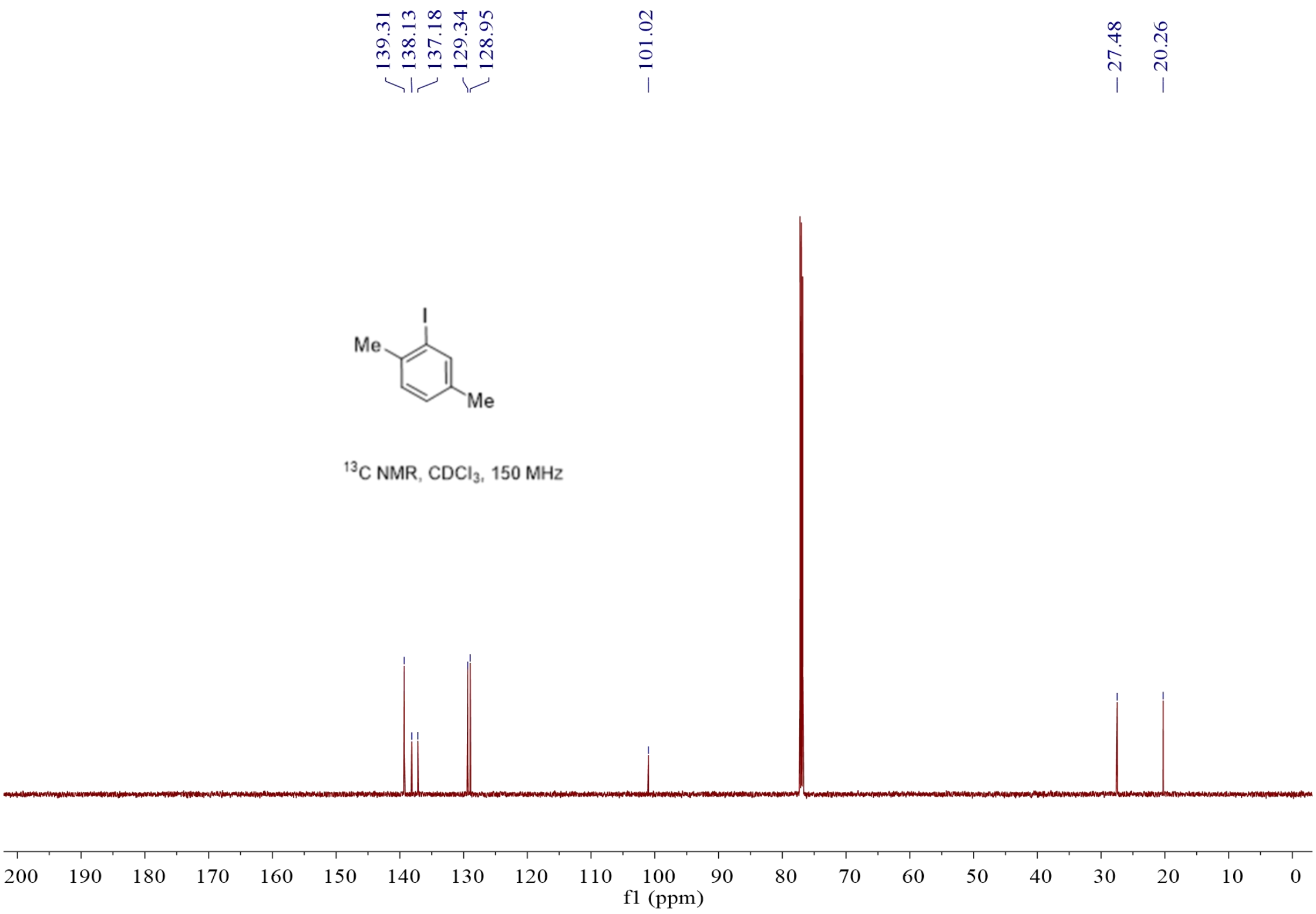
139.31
138.13
137.18
129.34
128.95
101.02
27.48
20.26
Me
I
Me
13C NMR, CDCl3, 150 MHz
200 190 180 170 160 150 140 130 120 110 100 90 80 70 60 50 40 30 20 10 0
f1 (ppm)

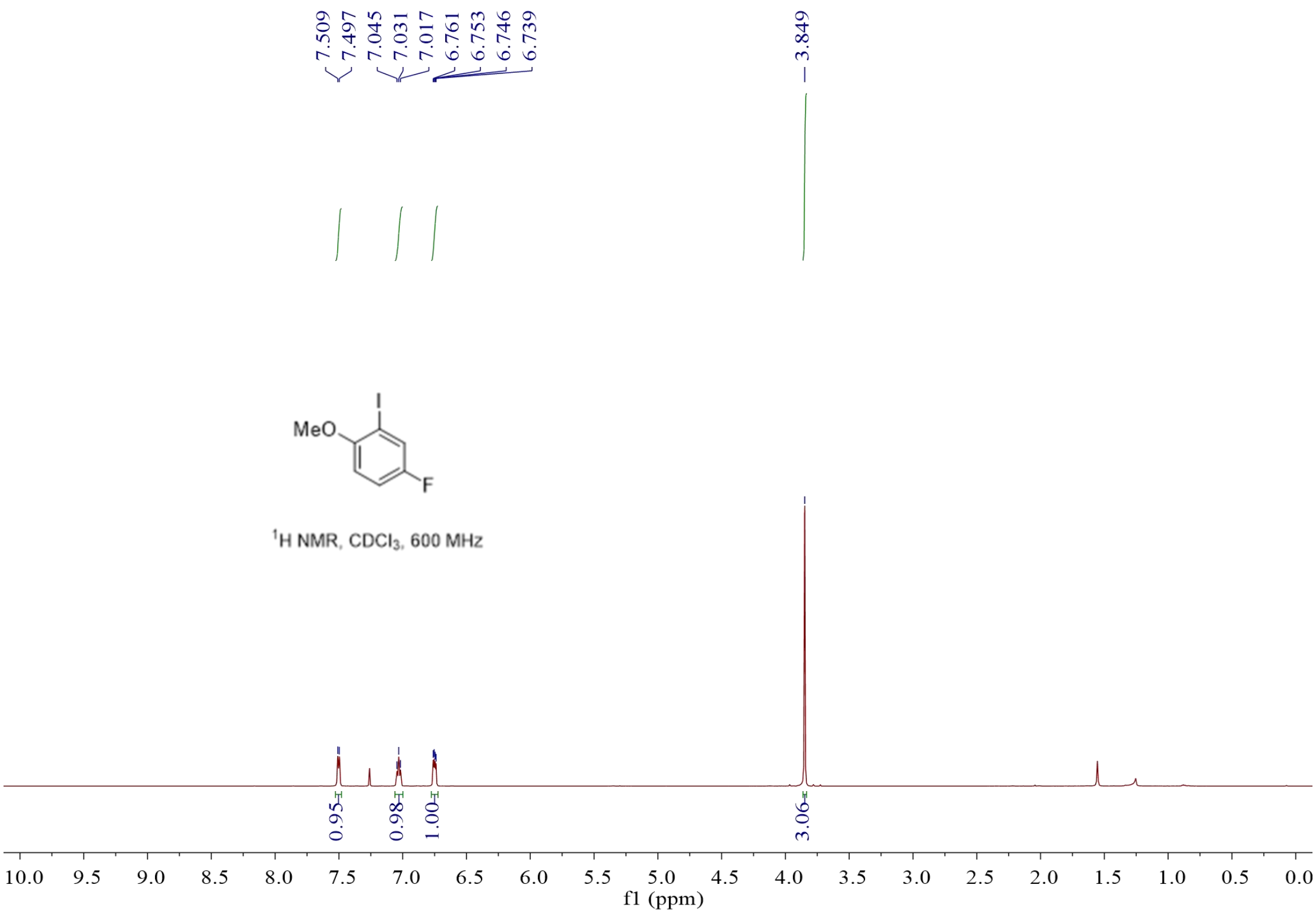
7.509
7.497
7.045
7.031
7.017
6.761
6.753
6.746
6.739
3.849
MeO
I
F
1H NMR, CDCl3, 600 MHz
0.95
0.98
1.00
3.06
10.0 9.5 9.0 8.5 8.0 7.5 7.0 6.5 6.0 5.5 5.0 4.5 4.0 3.5 3.0 2.5 2.0 1.5 1.0 0.5 0.0
f1 (ppm)

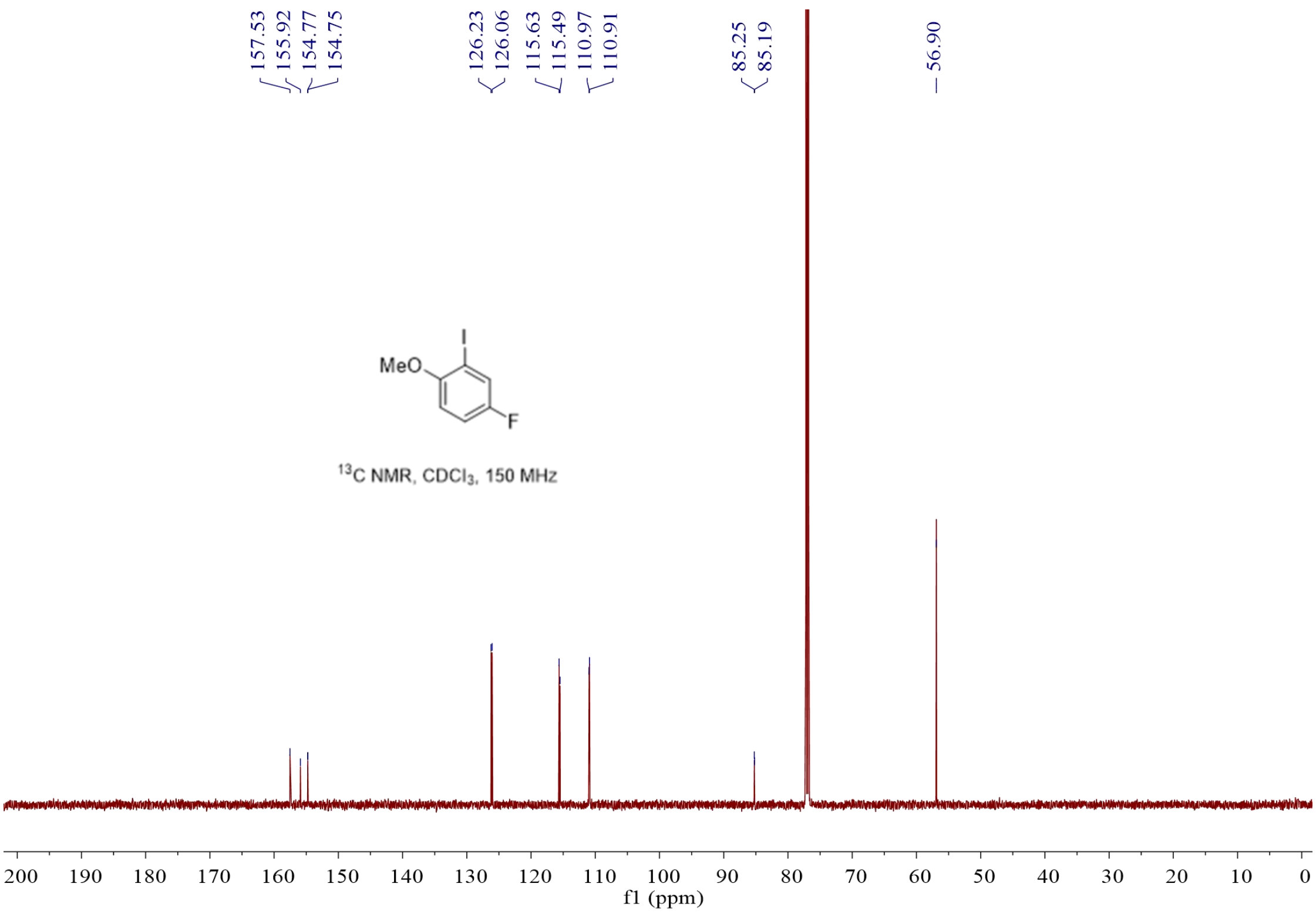
157.53
155.92
154.77
154.75
126.23
126.06
115.63
115.49
110.97
110.91
85.25
85.19
56.90
MeO
I
F
13C NMR, CDCl3, 150 MHz
200 190 180 170 160 150 140 130 120 110 100 90 80 70 60 50 40 30 20 10 0
f1 (ppm)

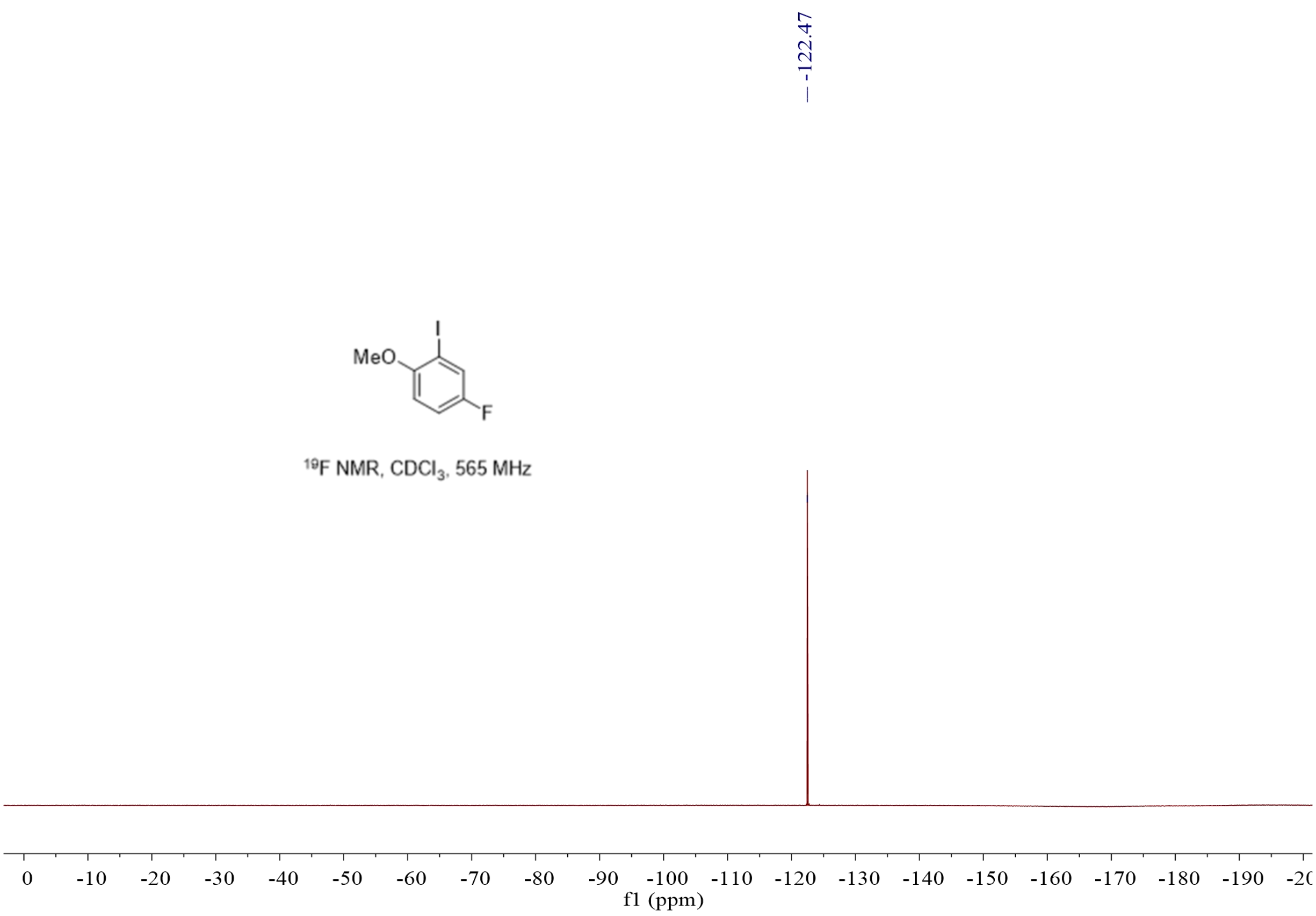
-122.47
MeO
I
F
19F NMR, CDCl3, 565 MHz
0 -10 -20 -30 -40 -50 -60 -70 -80 -90 -100 -110 -120 -130 -140 -150 -160 -170 -180 -190
f1 (ppm)

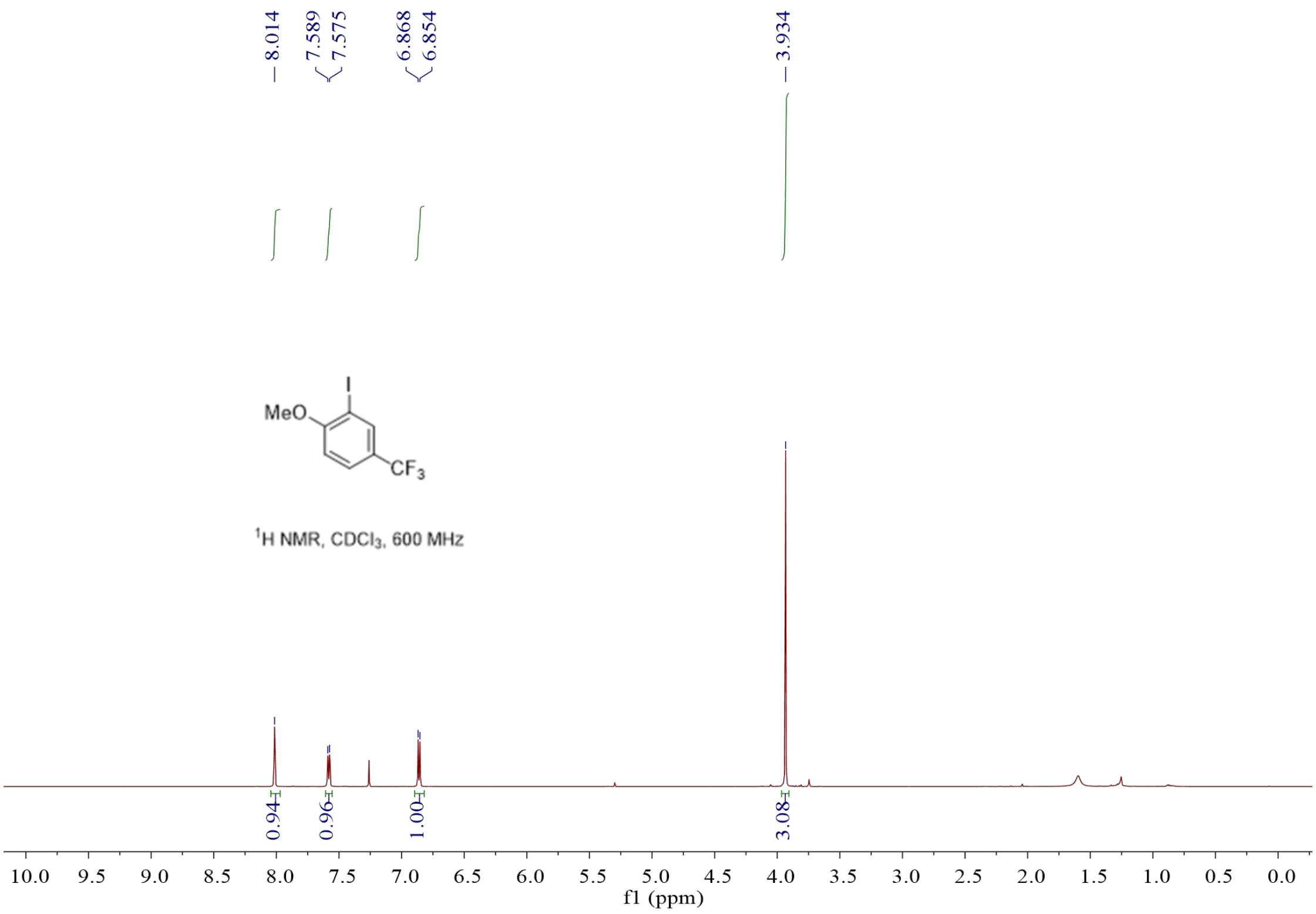
8.014
7.589
7.575
6.868
6.854
3.934
MeO
I
CF3
1H NMR, CDCl3, 600 MHz
0.94
0.96
1.00
3.08
10.0 9.5 9.0 8.5 8.0 7.5 7.0 6.5 6.0 5.5 5.0 4.5 4.0 3.5 3.0 2.5 2.0 1.5 1.0 0.5 0.0
f1 (ppm)

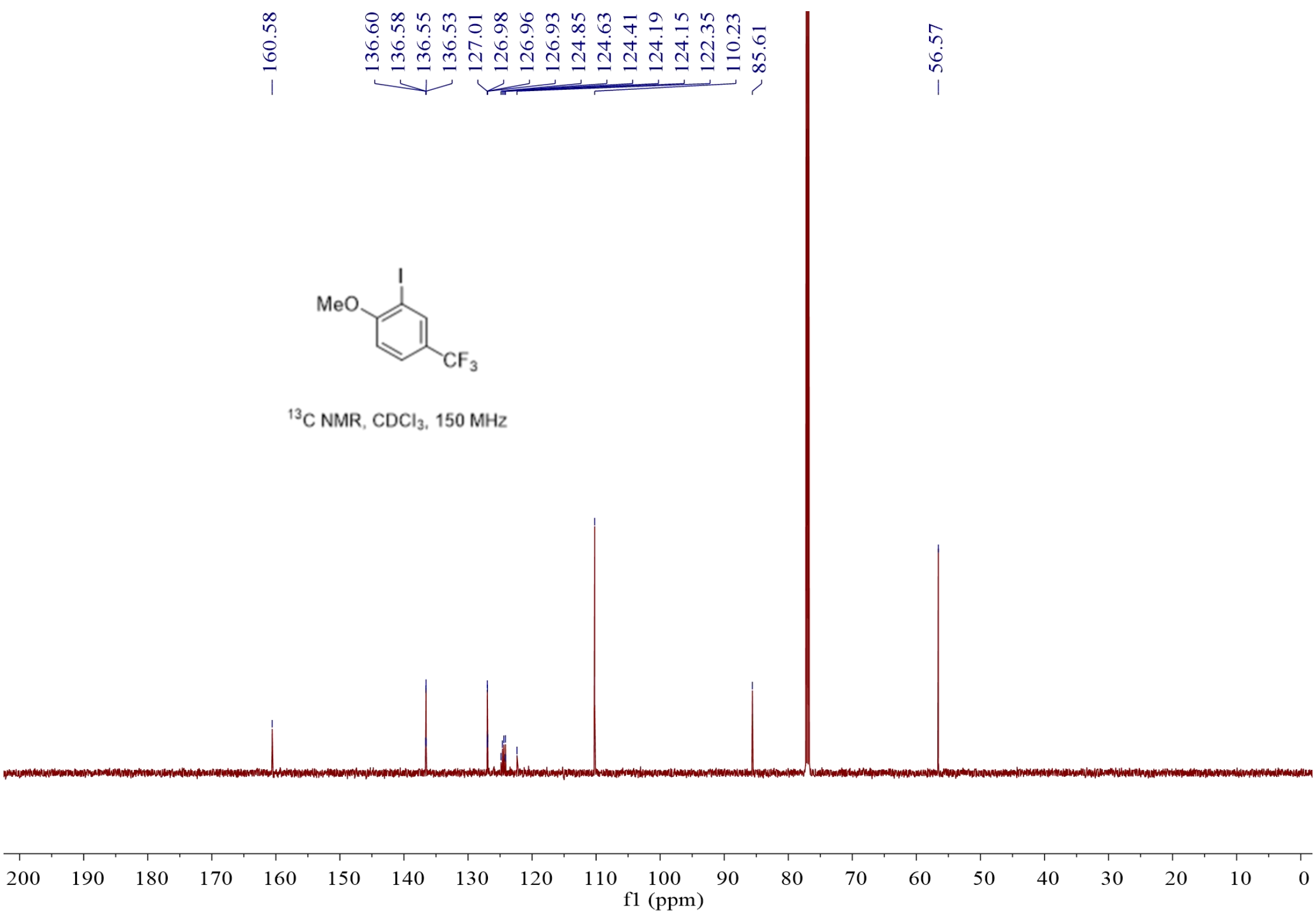
160.58
136.60
136.58
136.55
136.53
127.01
126.98
126.96
126.93
124.85
124.63
124.41
124.19
124.15
122.35
110.23
85.61
56.57
MeO
I
CF3
13C NMR, CDCl3, 150 MHz
200 190 180 170 160 150 140 130 120 110 100 90 80 70 60 50 40 30 20 10 0
f1 (ppm)

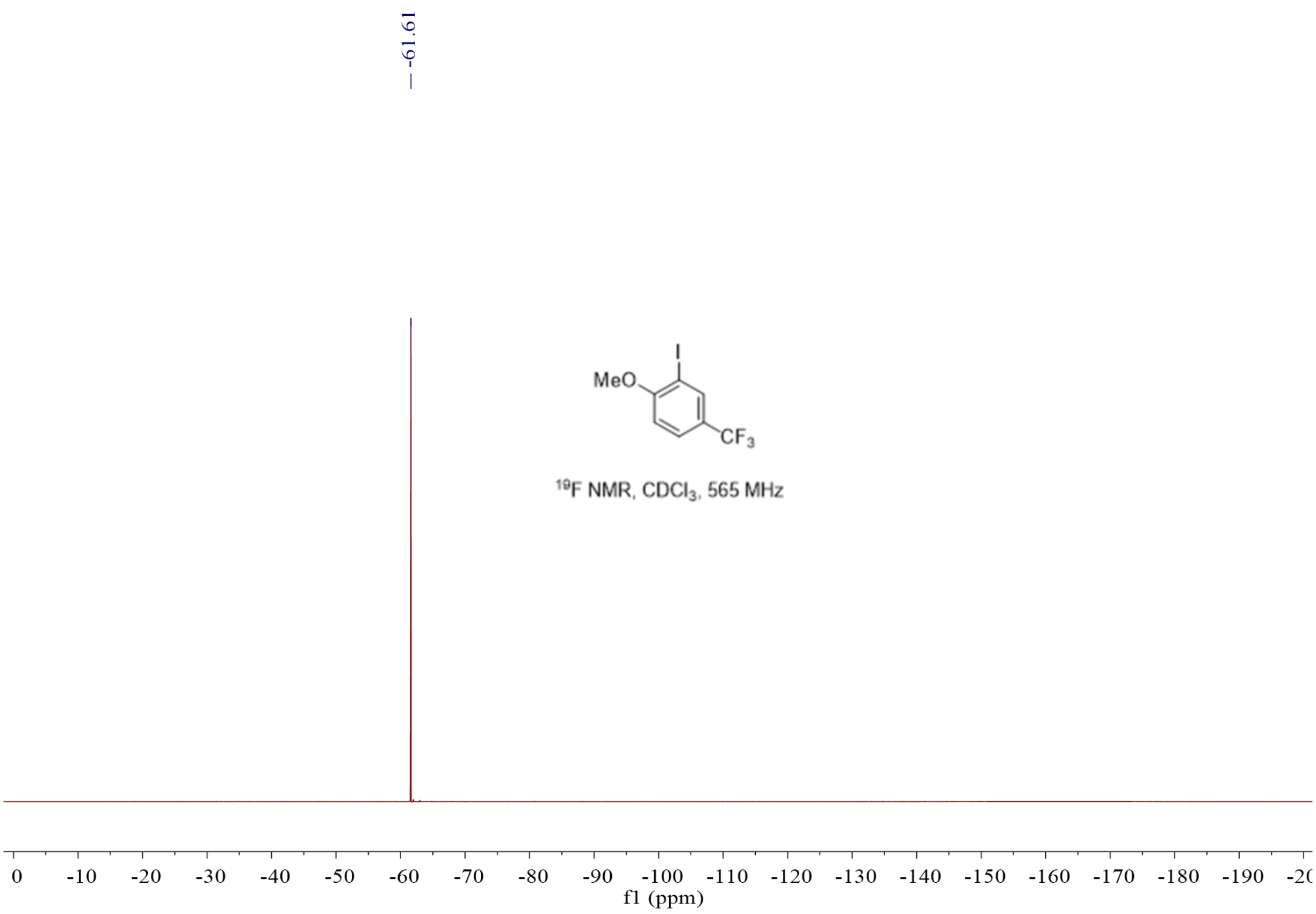
-61.61
MeO
I
CF3
19F NMR, CDCl3, 565 MHz
0 -10 -20 -30 -40 -50 -60 -70 -80 -90 -100 -110 -120 -130 -140 -150 -160 -170 -180 -190
f1 (ppm)

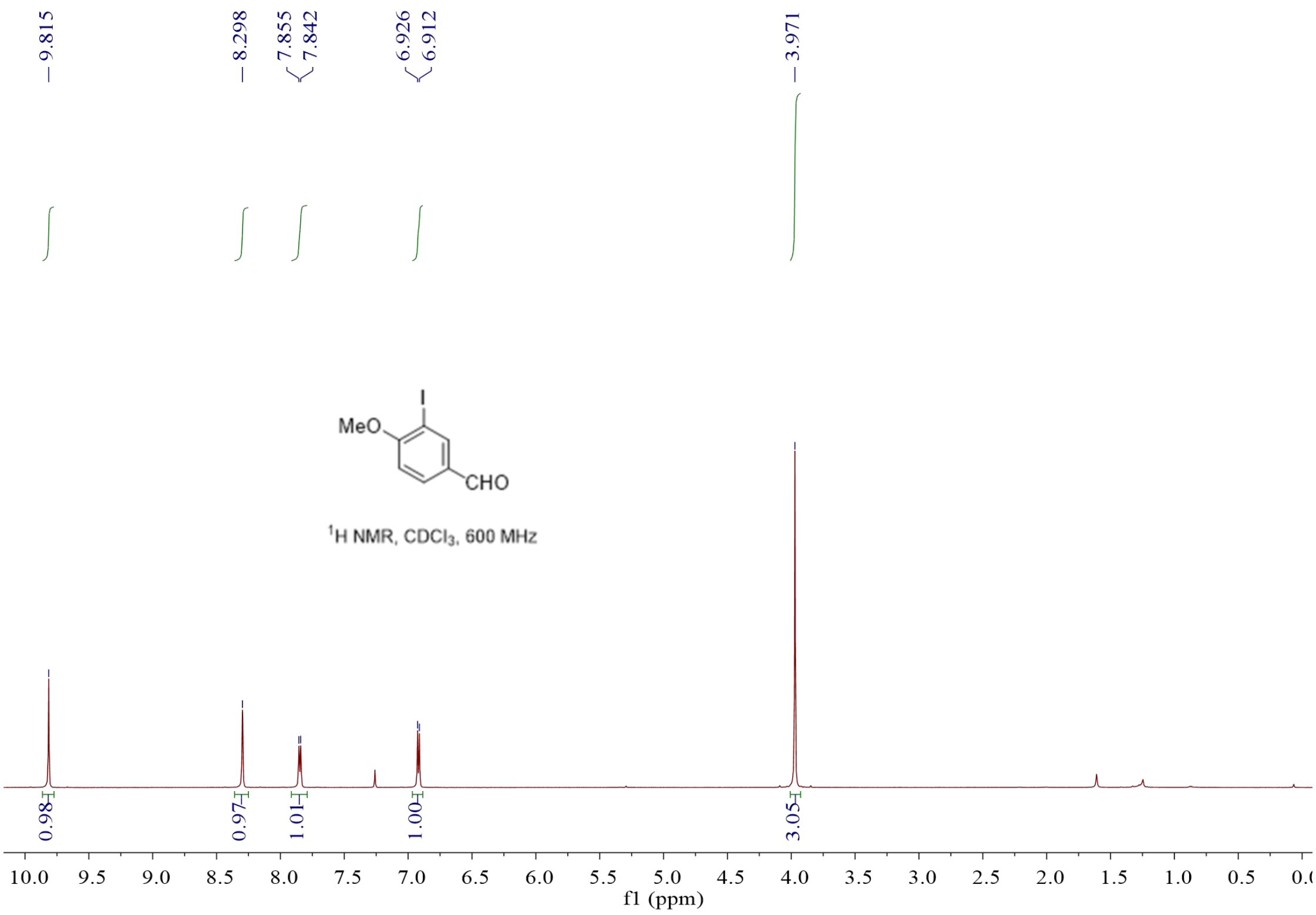
9.815
8.298
7.855
7.842
6.926
6.912
3.971
MeO
I
CHO
1H NMR, CDCl3, 600 MHz
0.98
0.97
1.01
1.00
3.05
10.0 9.5 9.0 8.5 8.0 7.5 7.0 6.5 6.0 5.5 5.0 4.5 4.0 3.5 3.0 2.5 2.0 1.5 1.0 0.5
f1 (ppm)

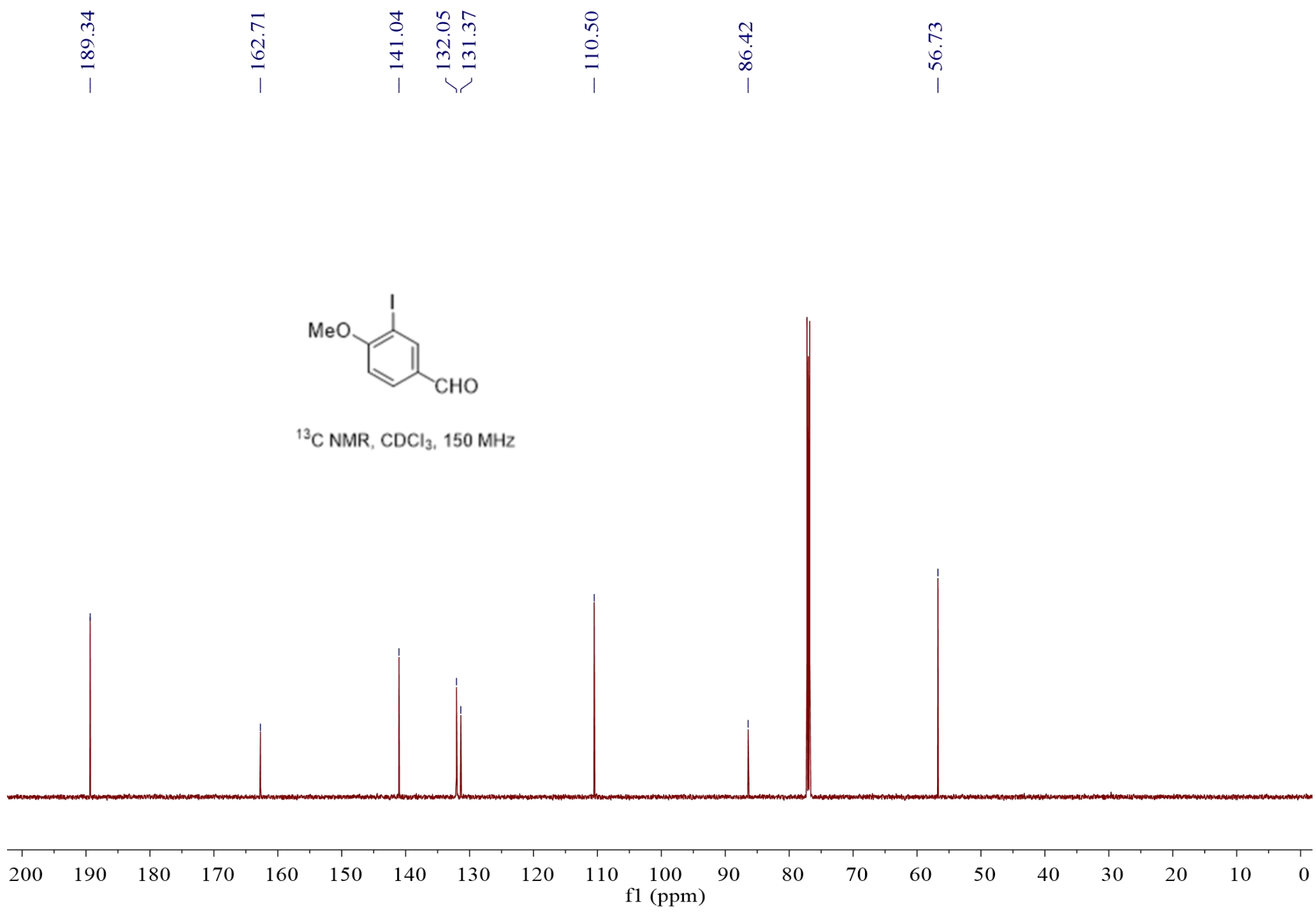
189.34
162.71
141.04
132.05
131.37
110.50
86.42
56.73
MeO
I
CHO
13C NMR, CDCl3, 150 MHz
200 190 180 170 160 150 140 130 120 110 100 90 80 70 60 50 40 30 20 10 0
f1 (ppm)

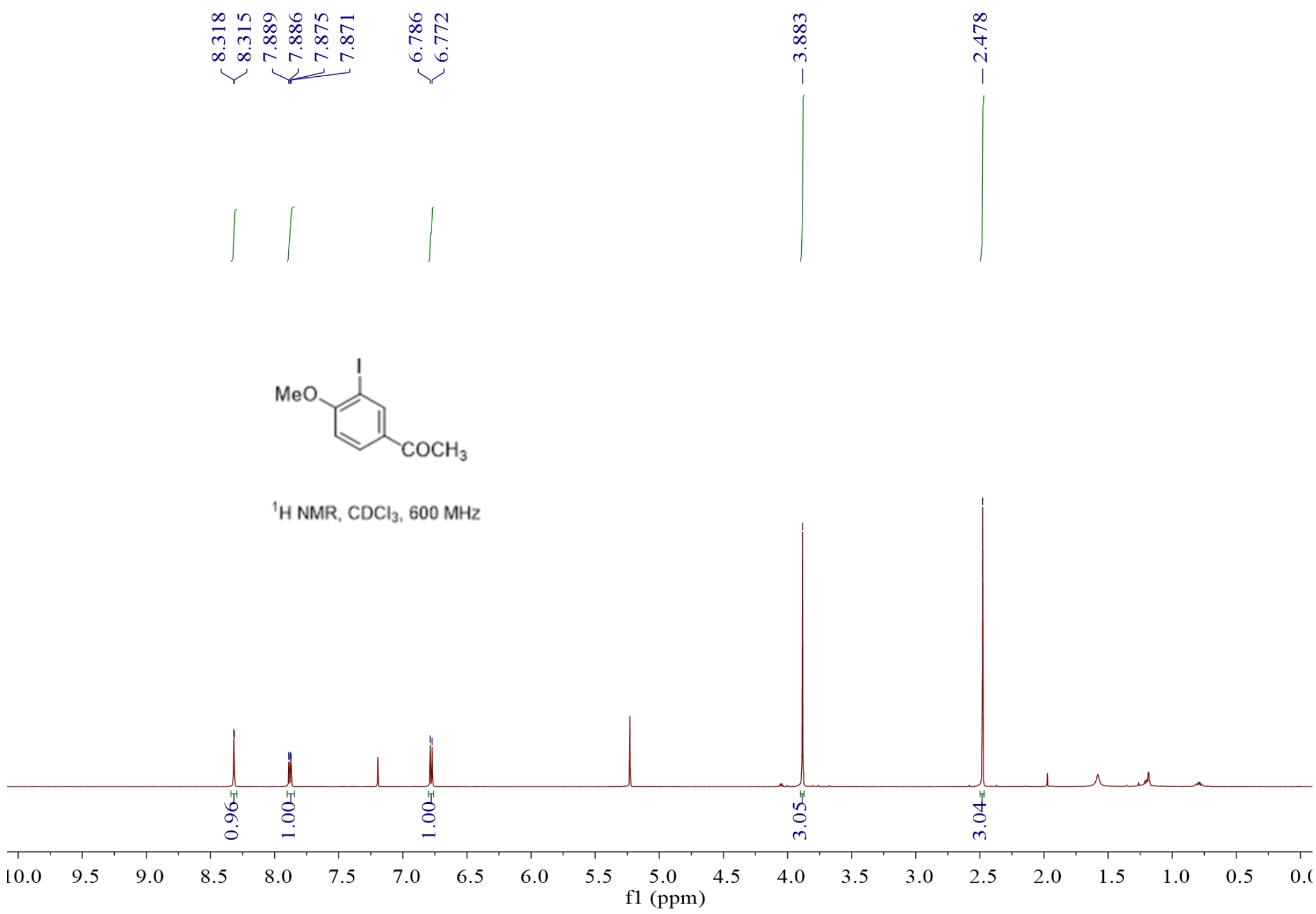
8.318
8.315
7.889
7.886
7.875
7.871
6.786
6.772
3.883
2.478
MeO
I
COCH3
1H NMR, CDCl3, 600 MHz
0.96
1.00
1.00
3.05
3.04
10.0 9.5 9.0 8.5 8.0 7.5 7.0 6.5 6.0 5.5 5.0 4.5 4.0 3.5 3.0 2.5 2.0 1.5 1.0 0.5 0.0
f1 (ppm)

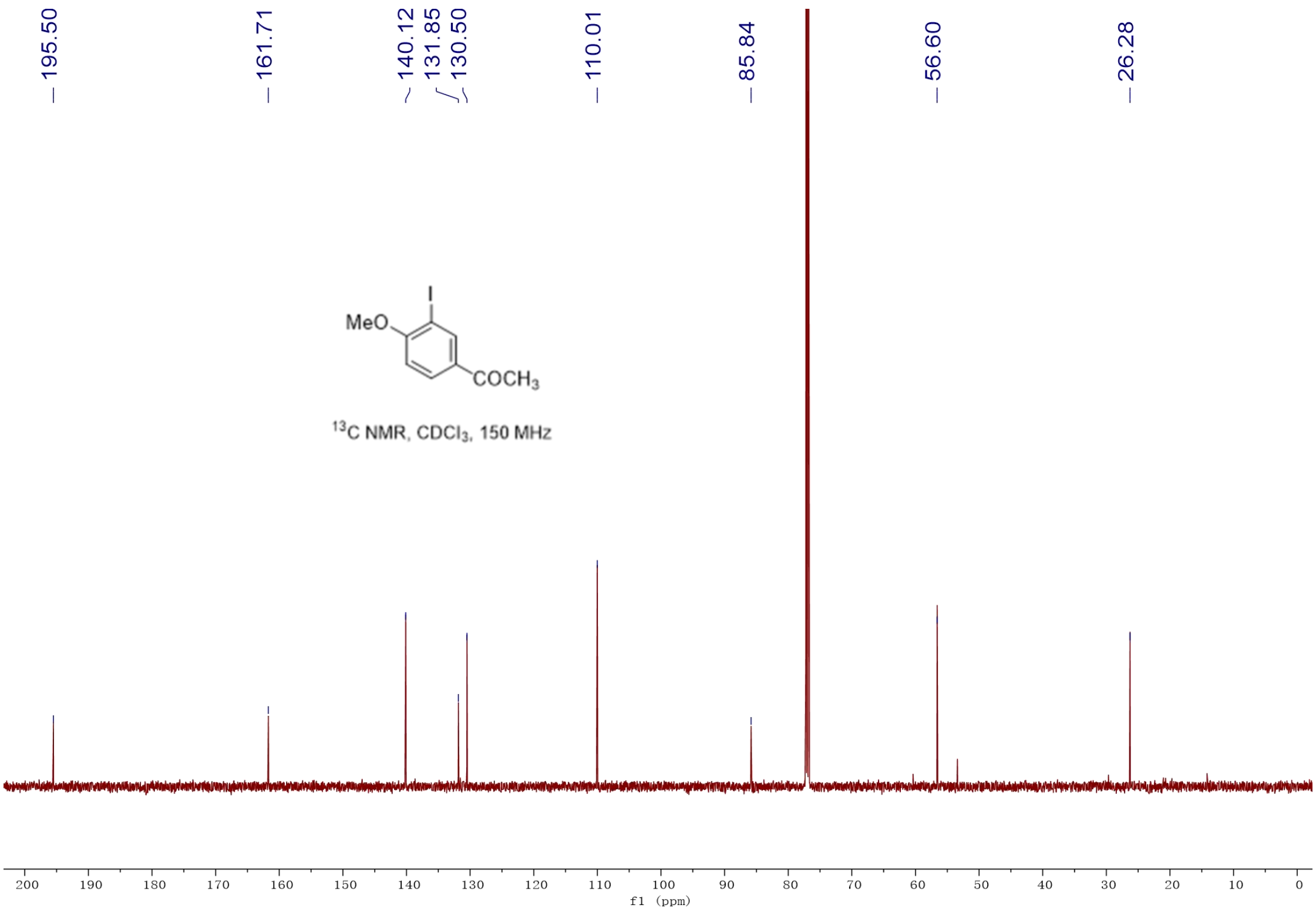
195.50
161.71
140.12
131.85
130.50
110.01
85.84
56.60
26.28
MeO
I
COCH3
13C NMR, CDCl3, 150 MHz
f1 (ppm)

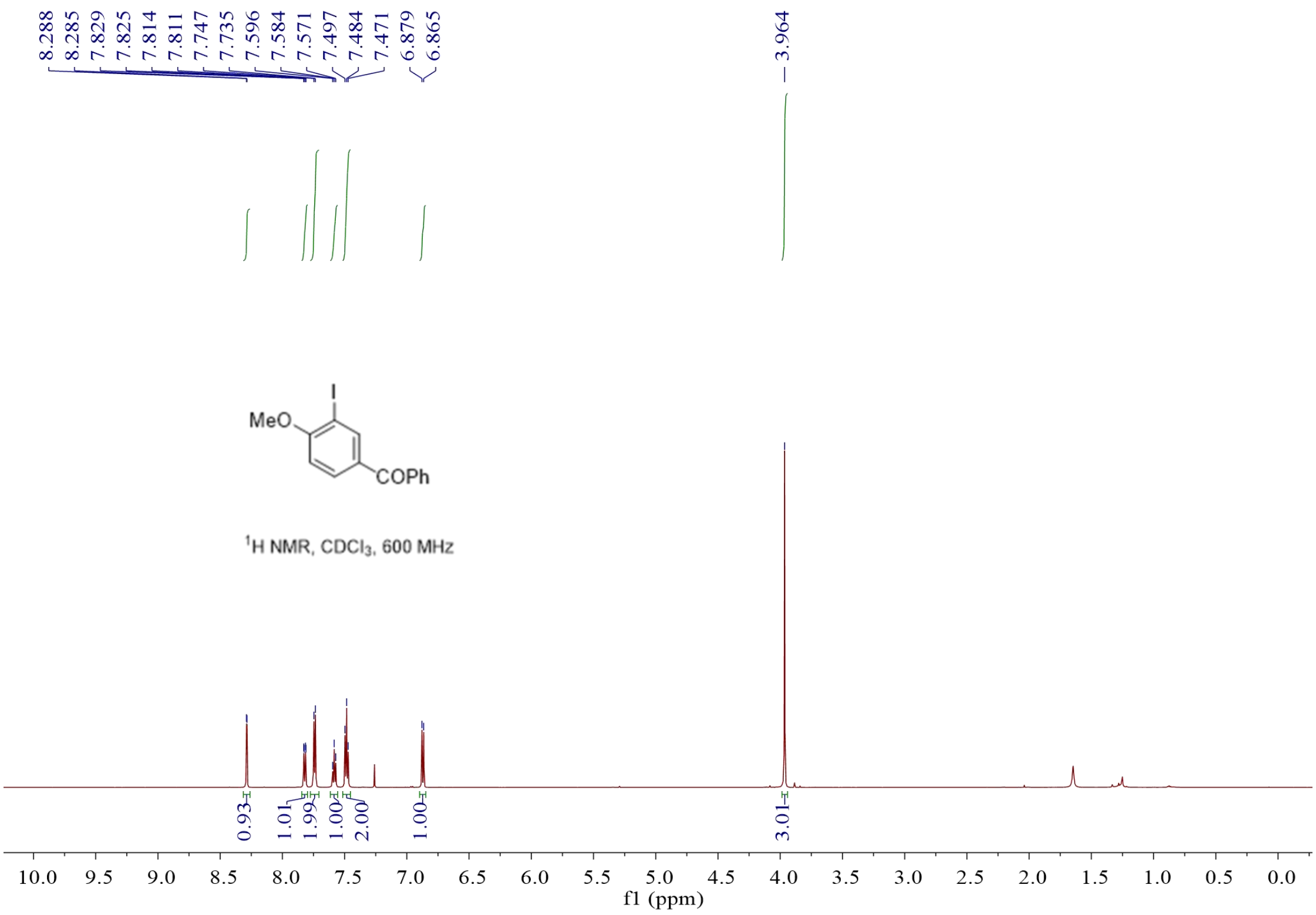
8.288
8.285
7.829
7.825
7.814
7.811
7.747
7.735
7.596
7.584
7.571
7.497
7.484
7.471
6.879
6.865
3.964
MeO
I
COPh
1H NMR, CDCl3, 600 MHz
0.93
1.01
1.99
1.00
2.00
1.00
3.01
f1 (ppm)

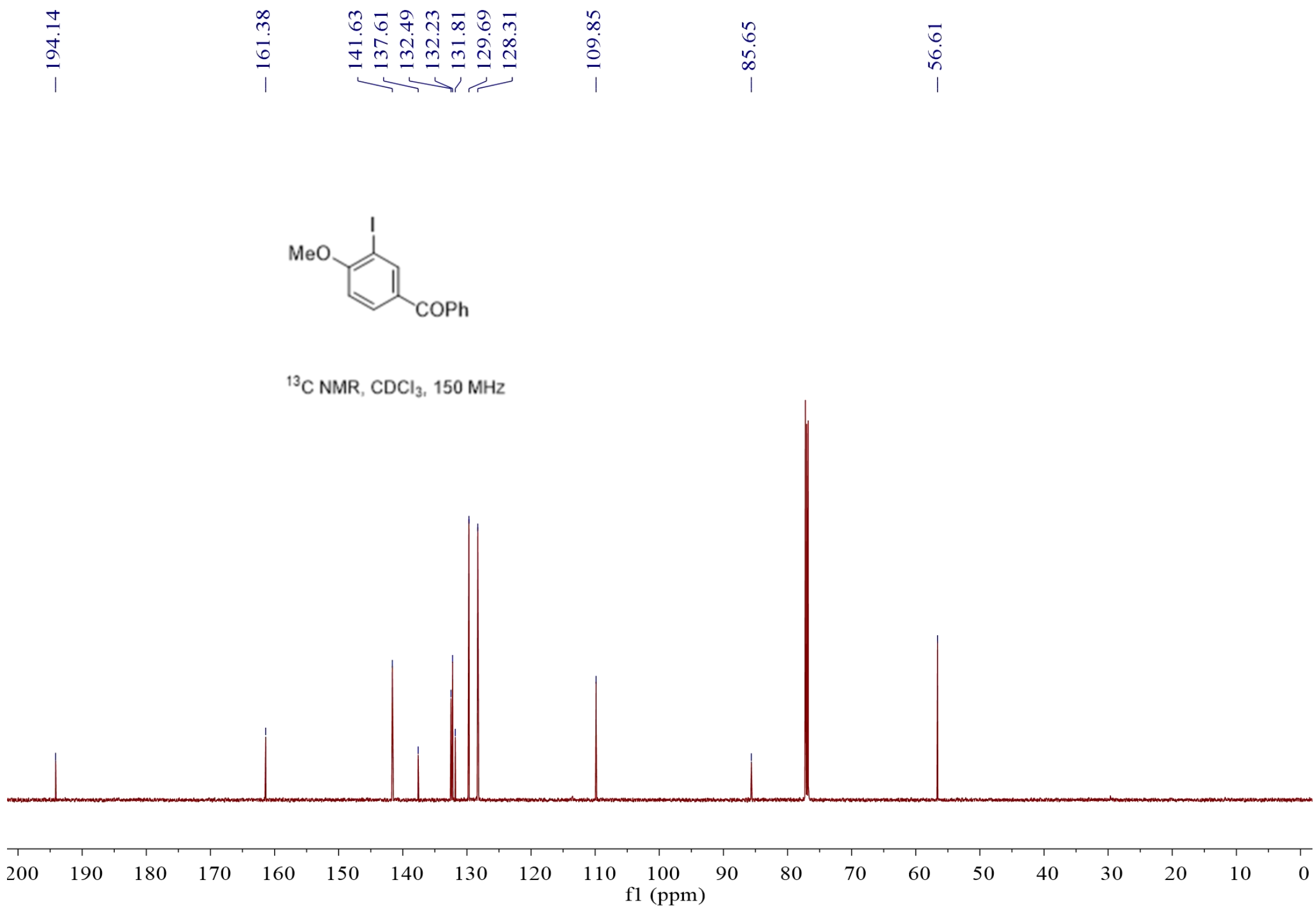
194.14
161.38
141.63
137.61
132.49
132.23
131.81
129.69
128.31
109.85
85.65
56.61
MeO
I
COPh
13C NMR, CDCl3, 150 MHz
200 190 180 170 160 150 140 130 120 110 100 90 80 70 60 50 40 30 20 10 0
f1 (ppm)

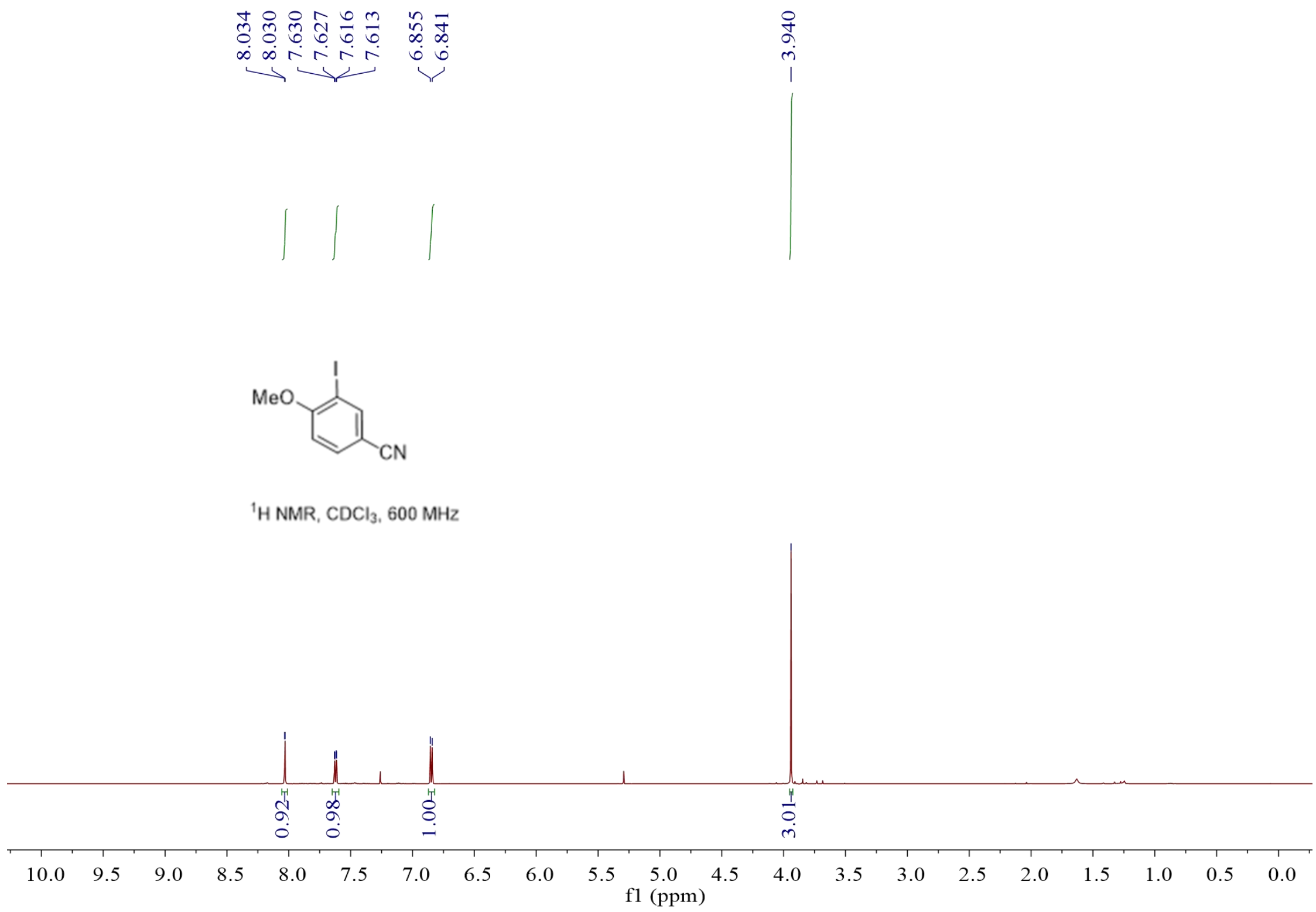
8.034
8.030
7.630
7.627
7.616
7.613
6.855
6.841
3.940
MeO
I
CN
1H NMR, CDCl3, 600 MHz
0.92
0.98
1.00
3.01
10.0 9.5 9.0 8.5 8.0 7.5 7.0 6.5 6.0 5.5 5.0 4.5 4.0 3.5 3.0 2.5 2.0 1.5 1.0 0.5 0.0
f1 (ppm)

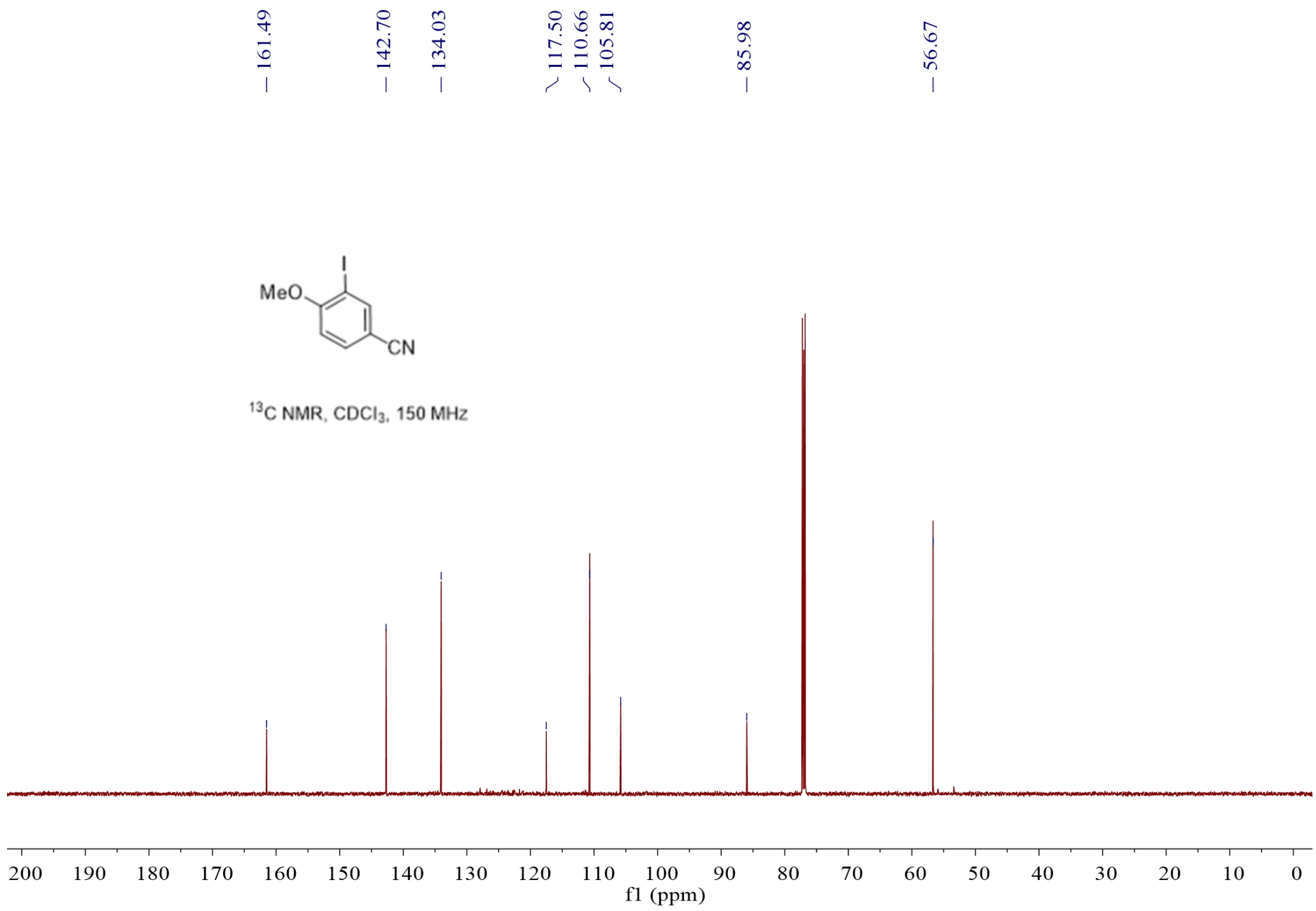
161.49
142.70
134.03
117.50
110.66
105.81
85.98
56.67
MeO
I
CN
^{13}C NMR, CDCl$_3$, 150 MHz
200 190 180 170 160 150 140 130 120 110 100 90 80 70 60 50 40 30 20 10 0
f1 (ppm)

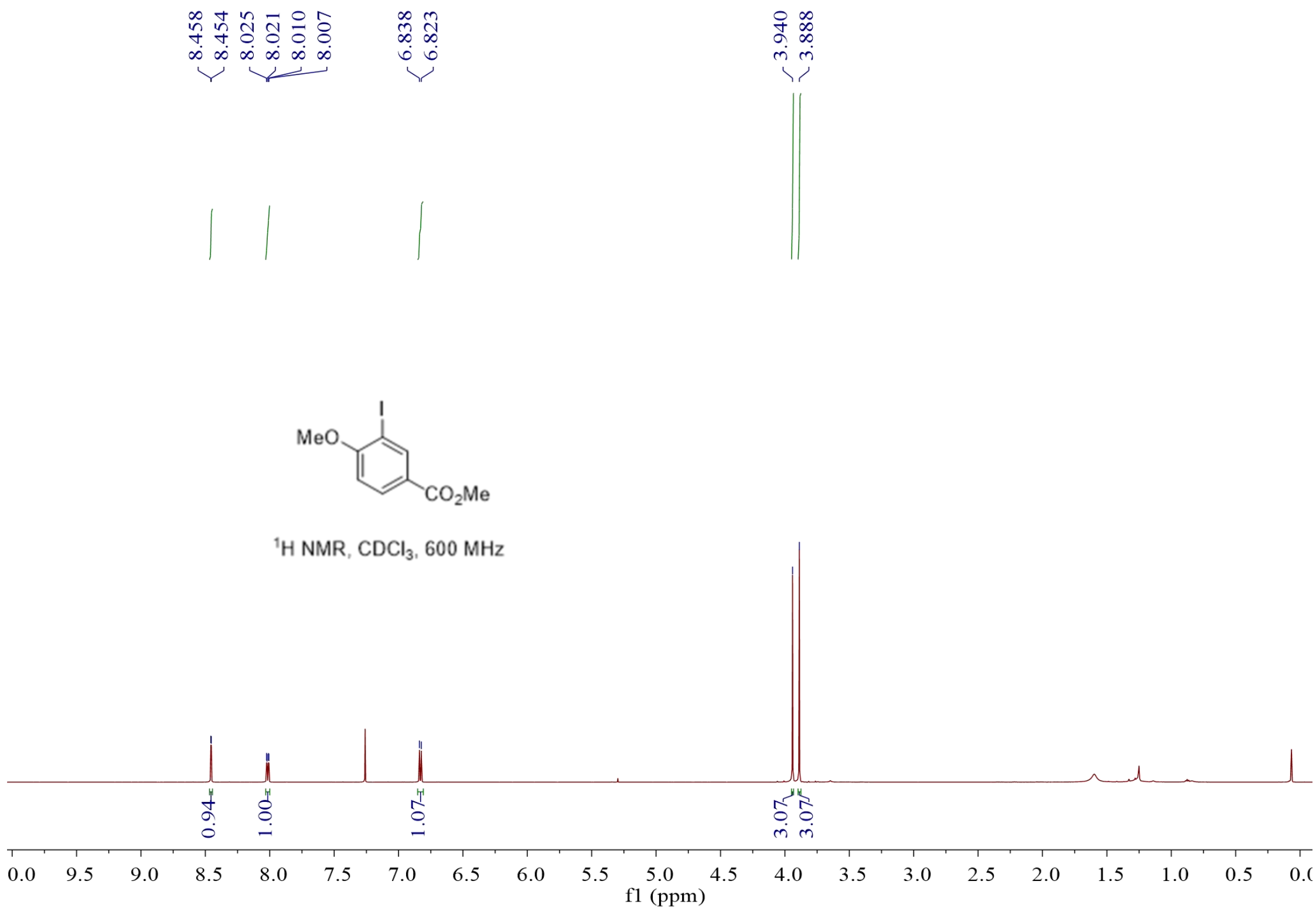
8.458
8.454
8.025
8.021
8.010
8.007
6.838
6.823
3.940
3.888
MeO
I
CO$_2$Me
^{1}H NMR, CDCl$_3$, 600 MHz
0.94
1.00
1.07
3.07
3.07
0.0 9.5 9.0 8.5 8.0 7.5 7.0 6.5 6.0 5.5 5.0 4.5 4.0 3.5 3.0 2.5 2.0 1.5 1.0 0.5 0.0
f1 (ppm)

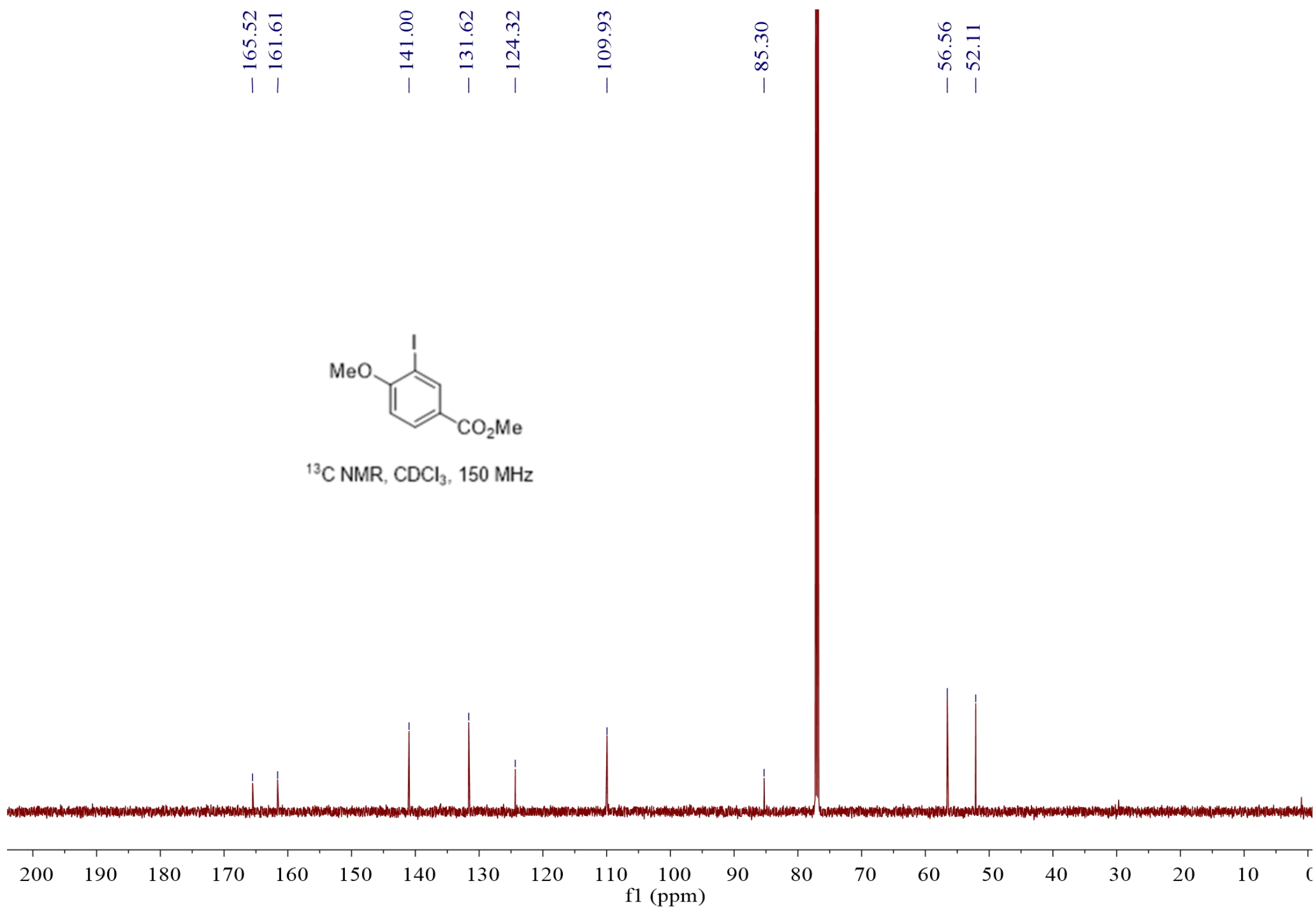
165.52
161.61
141.00
131.62
124.32
109.93
85.30
56.56
52.11
MeO
I
CO_2Me
^{13}C NMR, $CDCl_3$, 150 MHz
200 190 180 170 160 150 140 130 120 110 100 90 80 70 60 50 40 30 20 10
f1 (ppm)

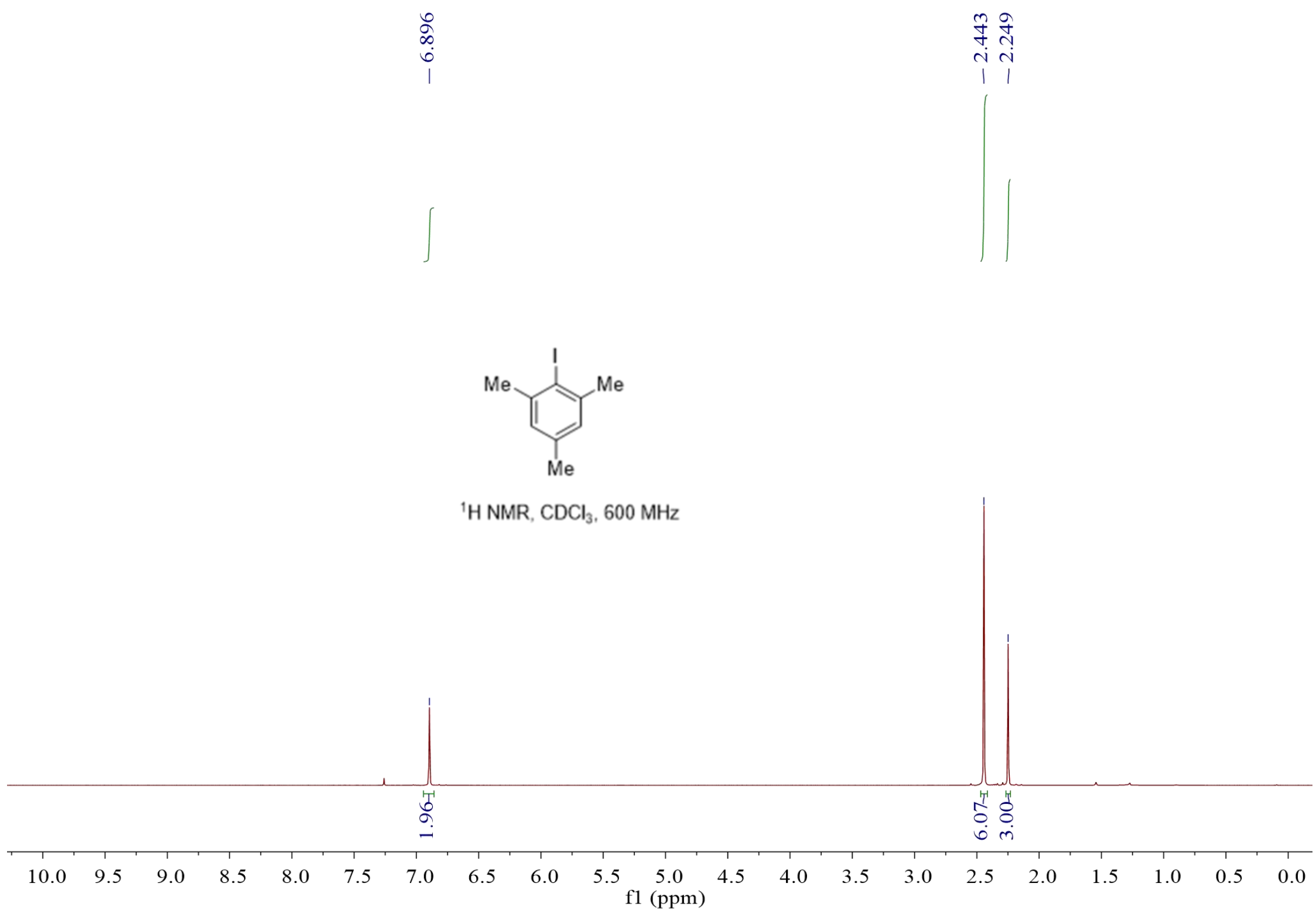
6.896
2.443
2.249
I
Me
Me
Me
^{1}H NMR, $CDCl_3$, 600 MHz
1.96
6.07
3.00
10.0 9.5 9.0 8.5 8.0 7.5 7.0 6.5 6.0 5.5 5.0 4.5 4.0 3.5 3.0 2.5 2.0 1.5 1.0 0.5 0.0
f1 (ppm)

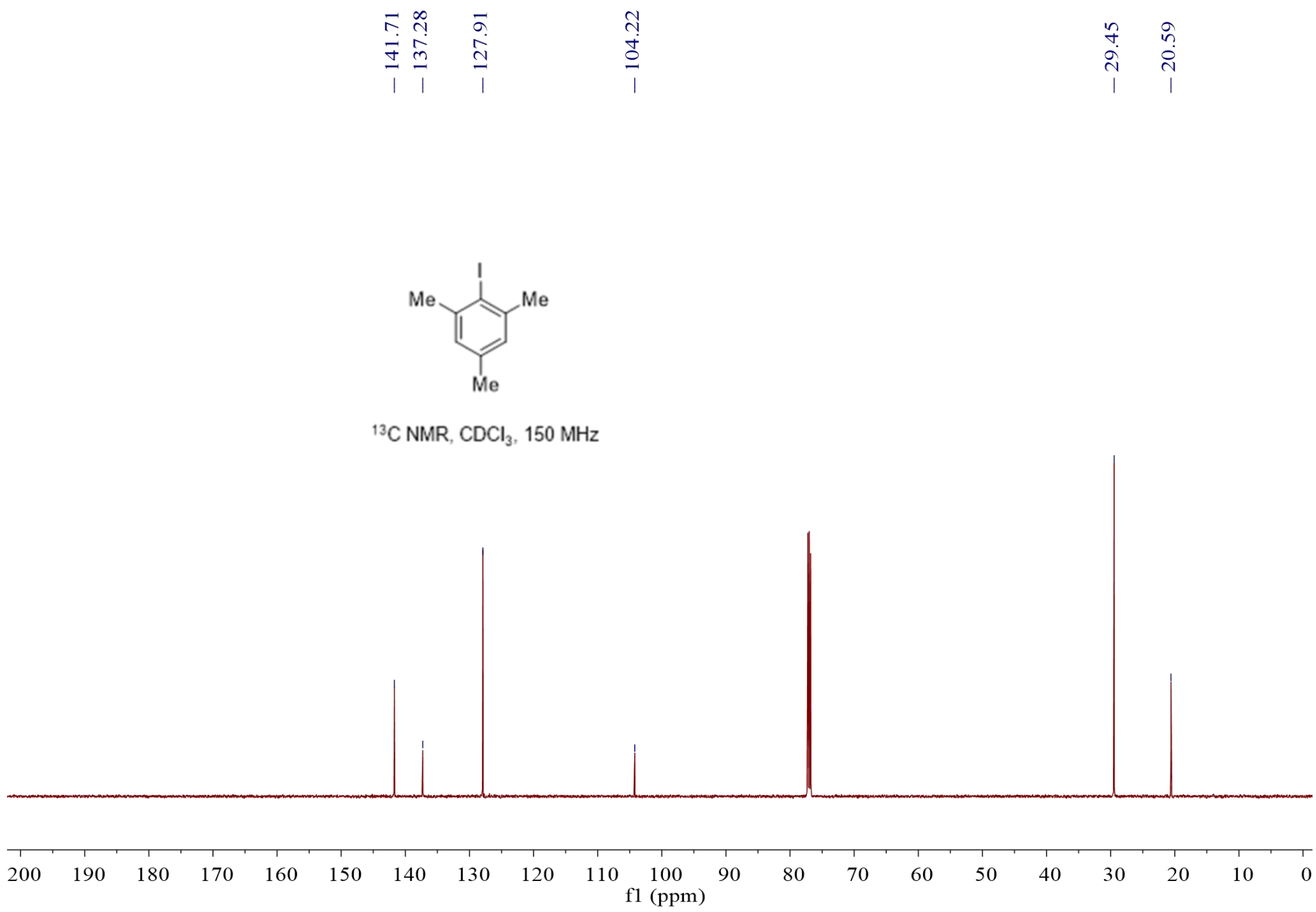
141.71
137.28
127.91
104.22
29.45
20.59
I
Me
Me
Me
^{13}C NMR, CDCl$_3$, 150 MHz
200 190 180 170 160 150 140 130 120 110 100 90 80 70 60 50 40 30 20 10 0
f1 (ppm)

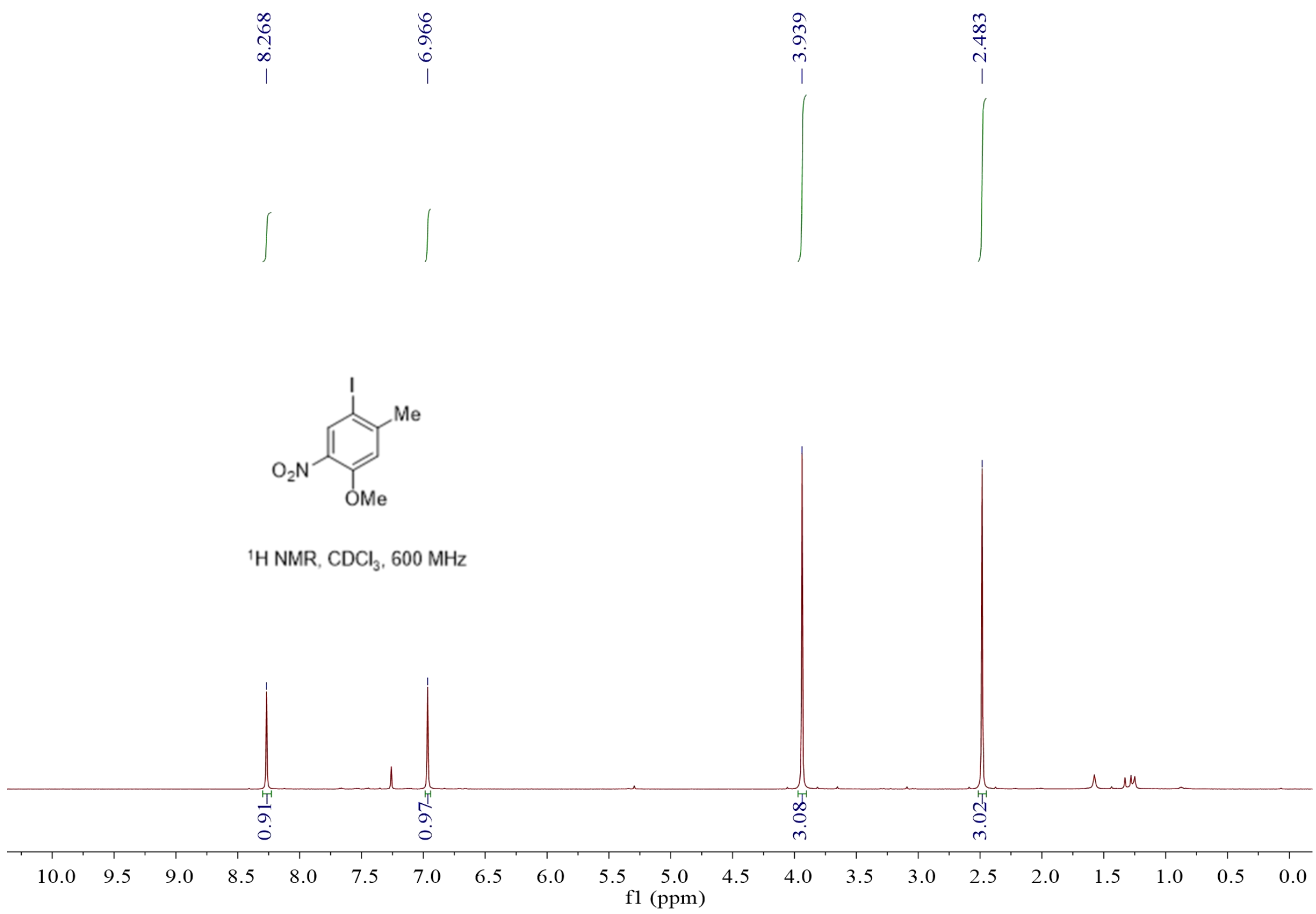
8.268
6.966
3.939
2.483
I
Me
O$_2$N
OMe
^{1}H NMR, CDCl$_3$, 600 MHz
0.91
0.97
3.08
3.02
10.0 9.5 9.0 8.5 8.0 7.5 7.0 6.5 6.0 5.5 5.0 4.5 4.0 3.5 3.0 2.5 2.0 1.5 1.0 0.5 0.0
f1 (ppm)

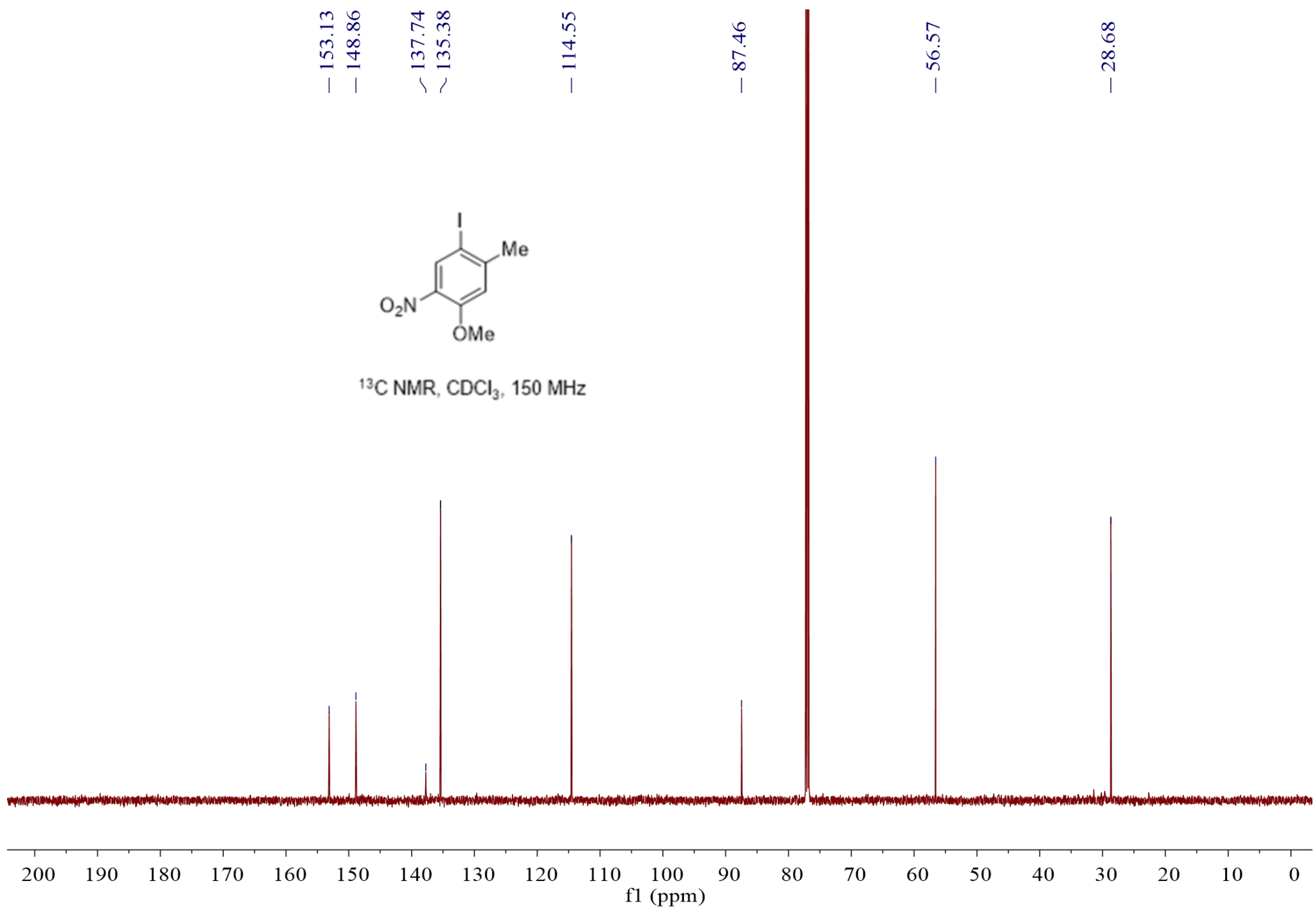

153.13
148.86
137.74
135.38
114.55
87.46
56.57
28.68
I
Me
O2N
OMe
13C NMR, CDCl3, 150 MHz
200 190 180 170 160 150 140 130 120 110 100 90 80 70 60 50 40 30 20 10 0
f1 (ppm)

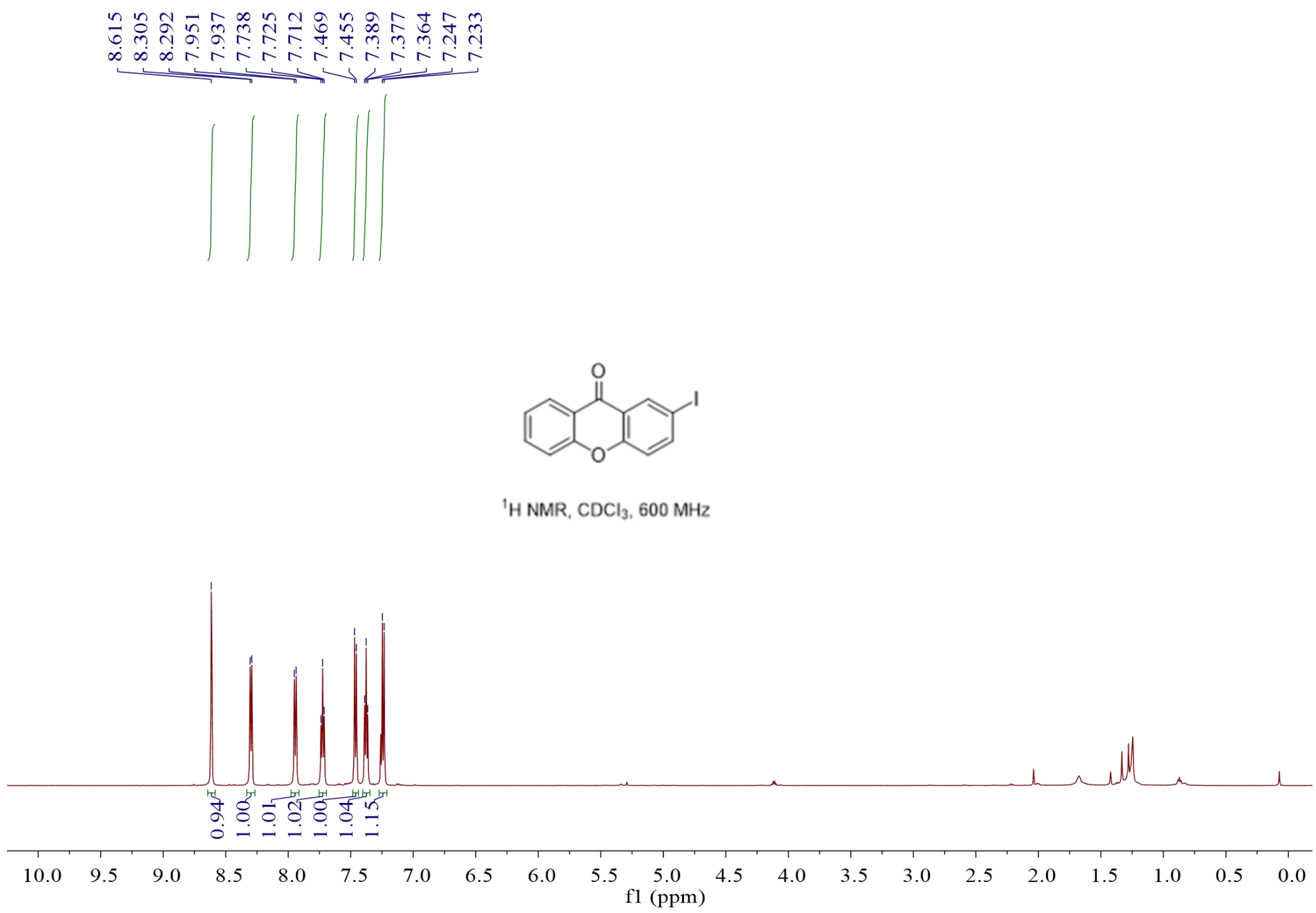

8.615
8.305
8.292
7.951
7.937
7.738
7.725
7.712
7.469
7.455
7.389
7.377
7.364
7.247
7.233
O
I
O
1H NMR, CDCl3, 600 MHz
0.94
1.00
1.01
1.02
1.00
1.04
1.15
10.0 9.5 9.0 8.5 8.0 7.5 7.0 6.5 6.0 5.5 5.0 4.5 4.0 3.5 3.0 2.5 2.0 1.5 1.0 0.5 0.0
f1 (ppm)

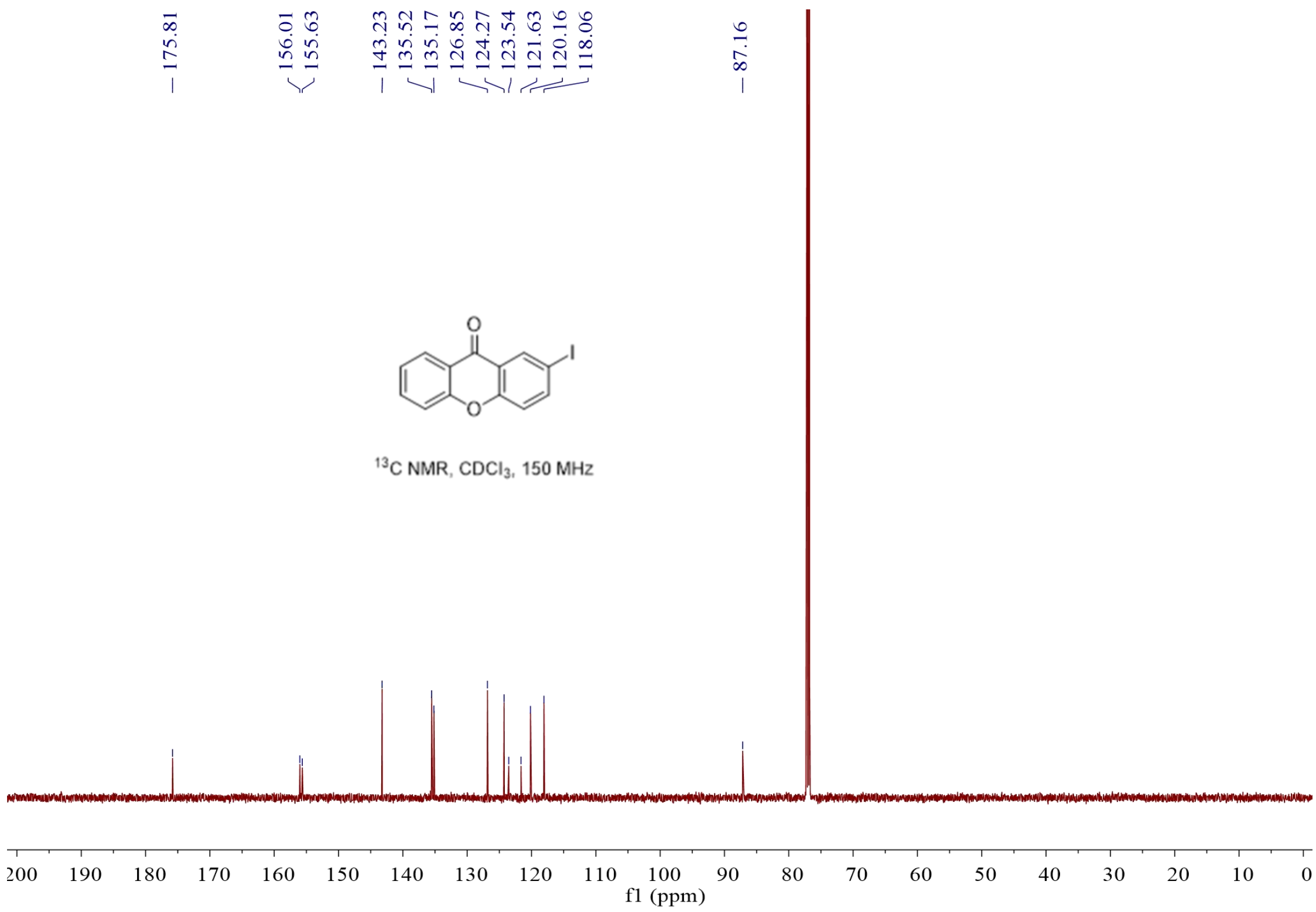
175.81
156.01
155.63
143.23
135.52
135.17
126.85
124.27
123.54
121.63
120.16
118.06
87.16
O
I
O
^{13}C NMR, $CDCl_3$, 150 MHz
200 190 180 170 160 150 140 130 120 110 100 90 80 70 60 50 40 30 20 10 0
f1 (ppm)

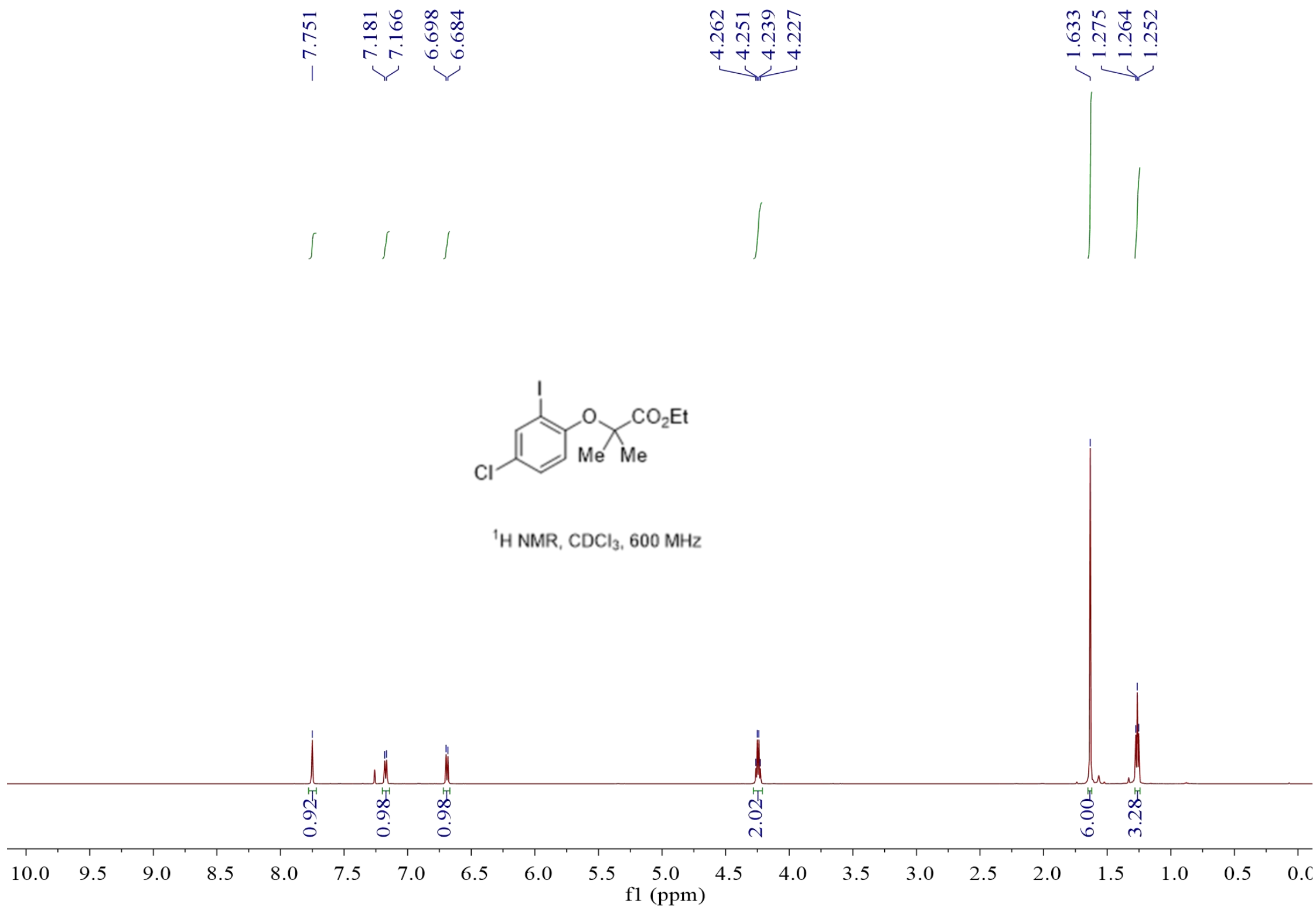
7.751
7.181
7.166
6.698
6.684
4.262
4.251
4.239
4.227
1.633
1.275
1.264
1.252
I
O
CO_2Et
Me Me
Cl
^{1}H NMR, $CDCl_3$, 600 MHz
0.92
0.98
0.98
2.02
6.00
3.28
10.0 9.5 9.0 8.5 8.0 7.5 7.0 6.5 6.0 5.5 5.0 4.5 4.0 3.5 3.0 2.5 2.0 1.5 1.0 0.5 0.0
f1 (ppm)

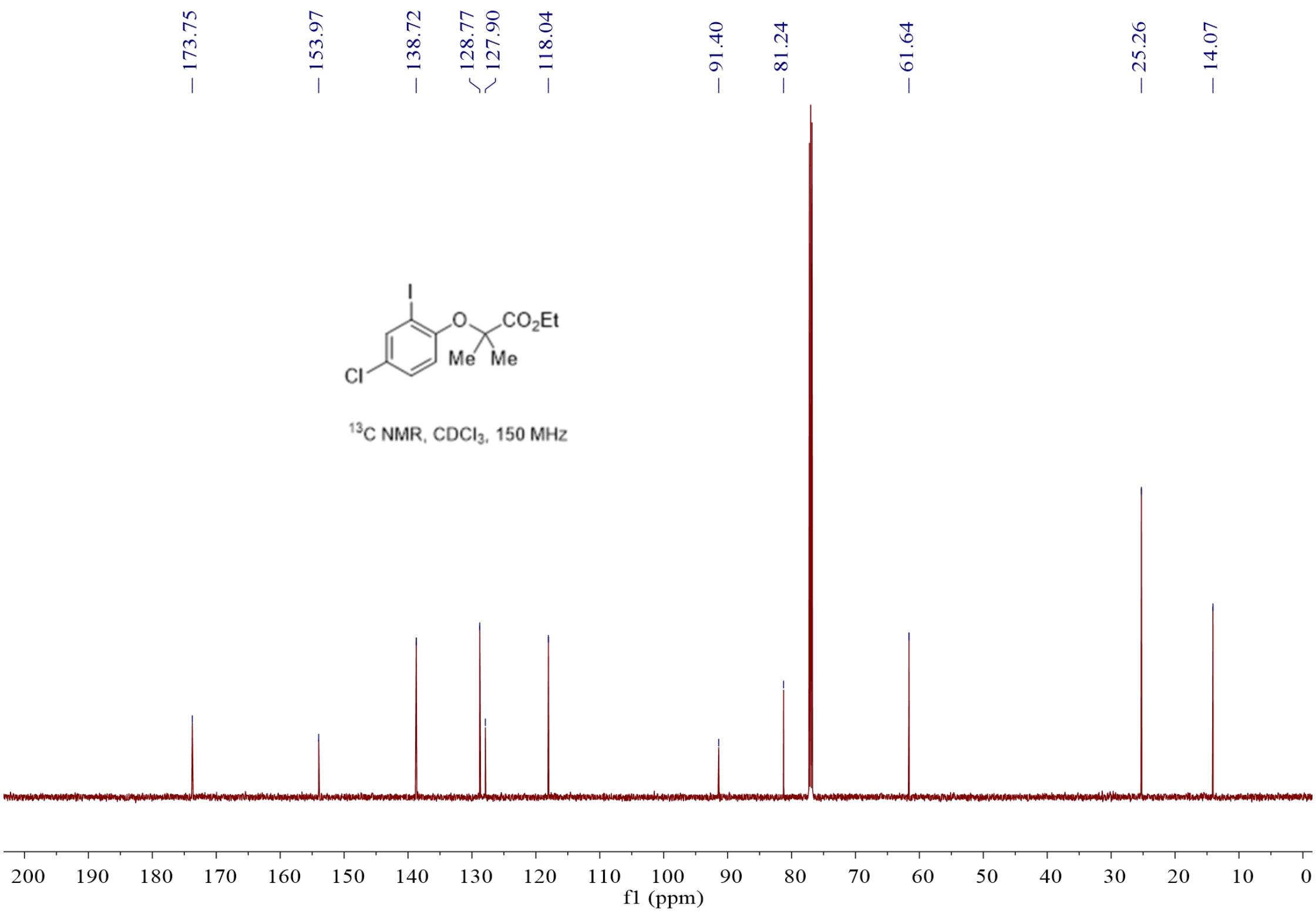

173.75
153.97
138.72
128.77
127.90
118.04
91.40
81.24
61.64
25.26
14.07
I
O
CO2Et
Me Me
Cl
13C NMR, CDCl3, 150 MHz
200 190 180 170 160 150 140 130 120 110 100 90 80 70 60 50 40 30 20 10 0
f1 (ppm)

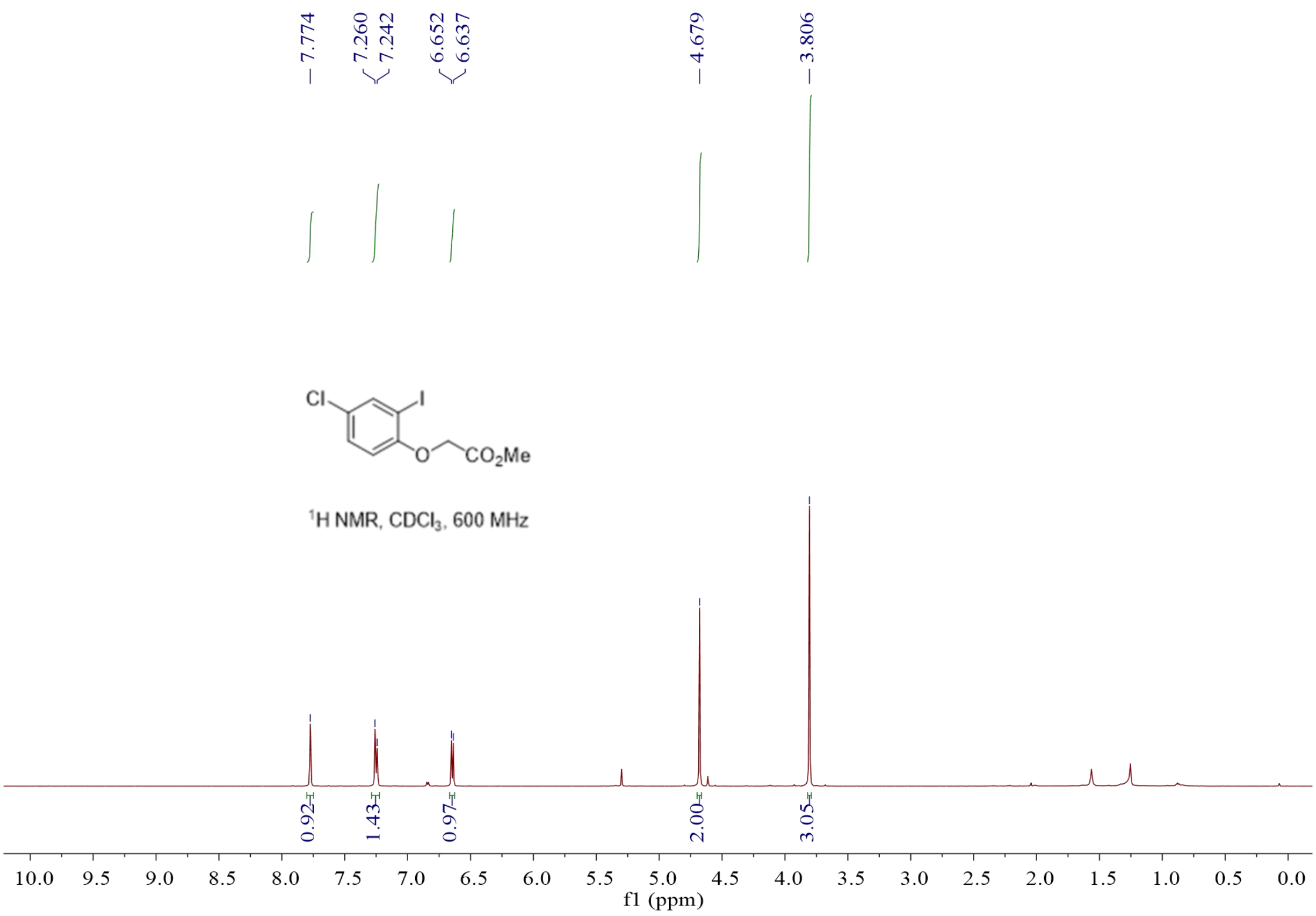

7.774
7.260
7.242
6.652
6.637
4.679
3.806
Cl
I
O
CO2Me
1H NMR, CDCl3, 600 MHz
0.92
1.43
0.97
2.00
3.05
10.0 9.5 9.0 8.5 8.0 7.5 7.0 6.5 6.0 5.5 5.0 4.5 4.0 3.5 3.0 2.5 2.0 1.5 1.0 0.5 0.0
f1 (ppm)

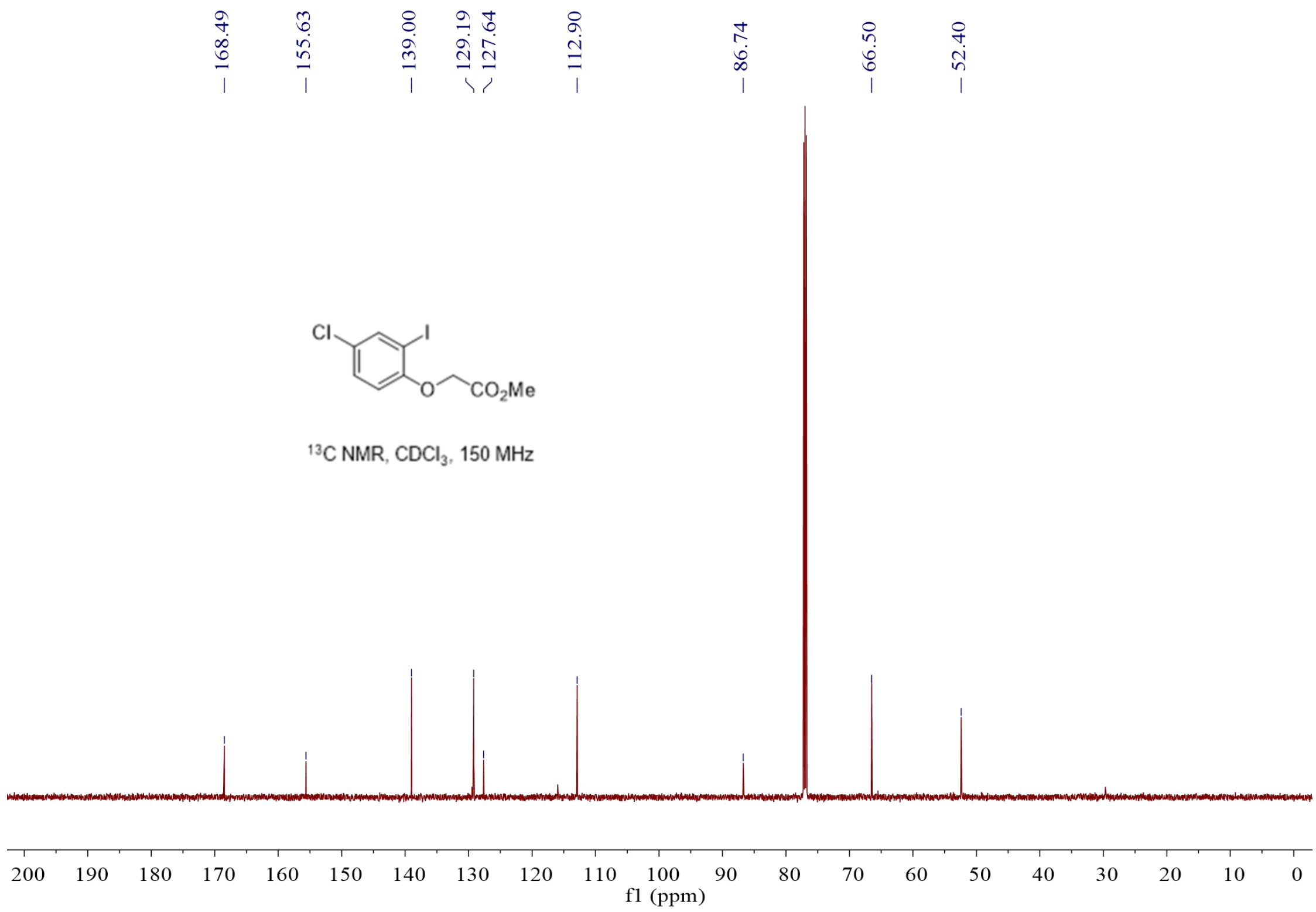
168.49
155.63
139.00
129.19
127.64
112.90
86.74
66.50
52.40
Cl
I
O
CO2Me
13C NMR, CDCl3, 150 MHz
200 190 180 170 160 150 140 130 120 110 100 90 80 70 60 50 40 30 20 10 0
f1 (ppm)

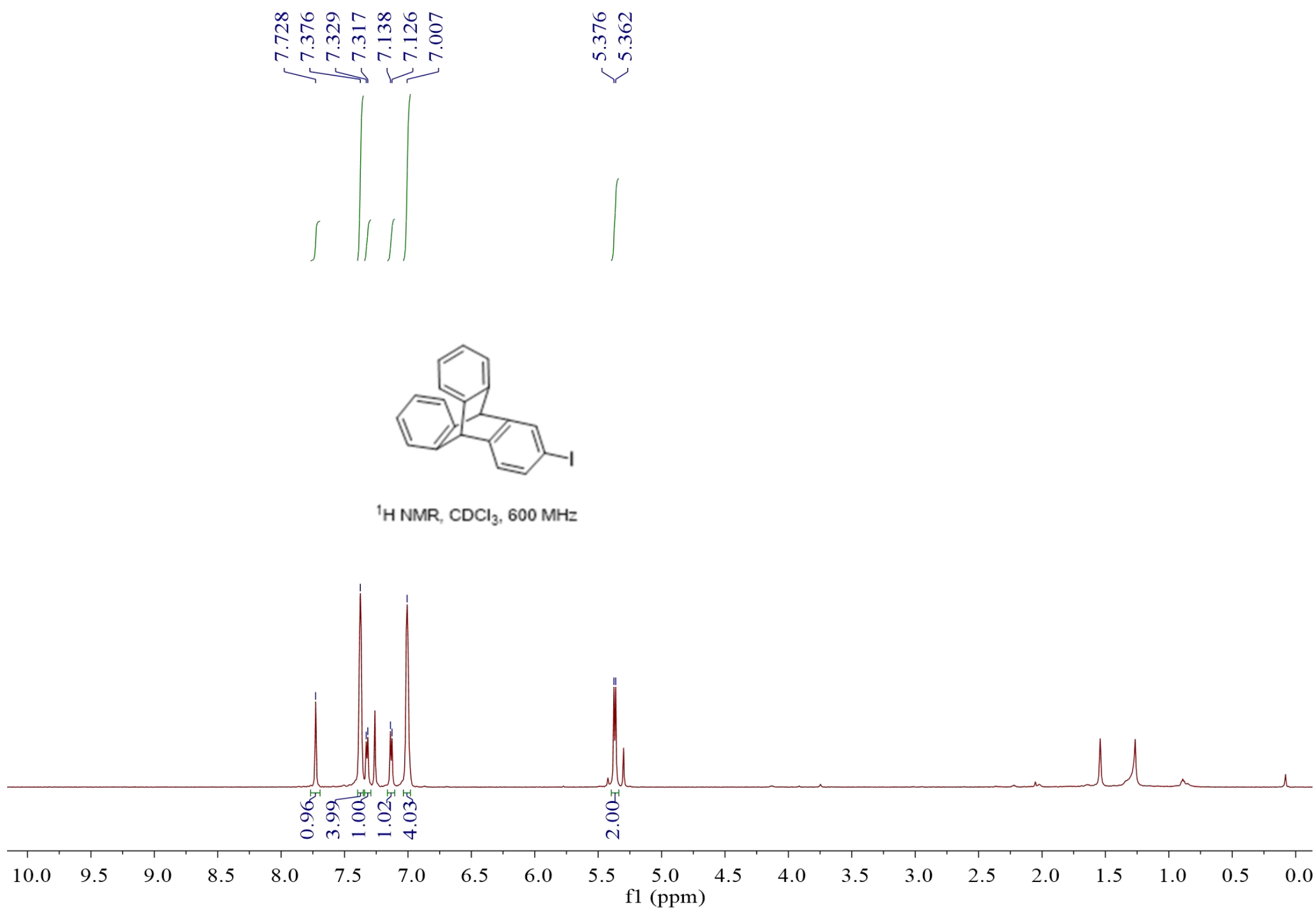
7.728
7.376
7.329
7.317
7.138
7.126
7.007
5.376
5.362
I
1H NMR, CDCl3, 600 MHz
0.96
3.99
1.00
1.02
4.03
2.00
10.0 9.5 9.0 8.5 8.0 7.5 7.0 6.5 6.0 5.5 5.0 4.5 4.0 3.5 3.0 2.5 2.0 1.5 1.0 0.5 0.0
f1 (ppm)

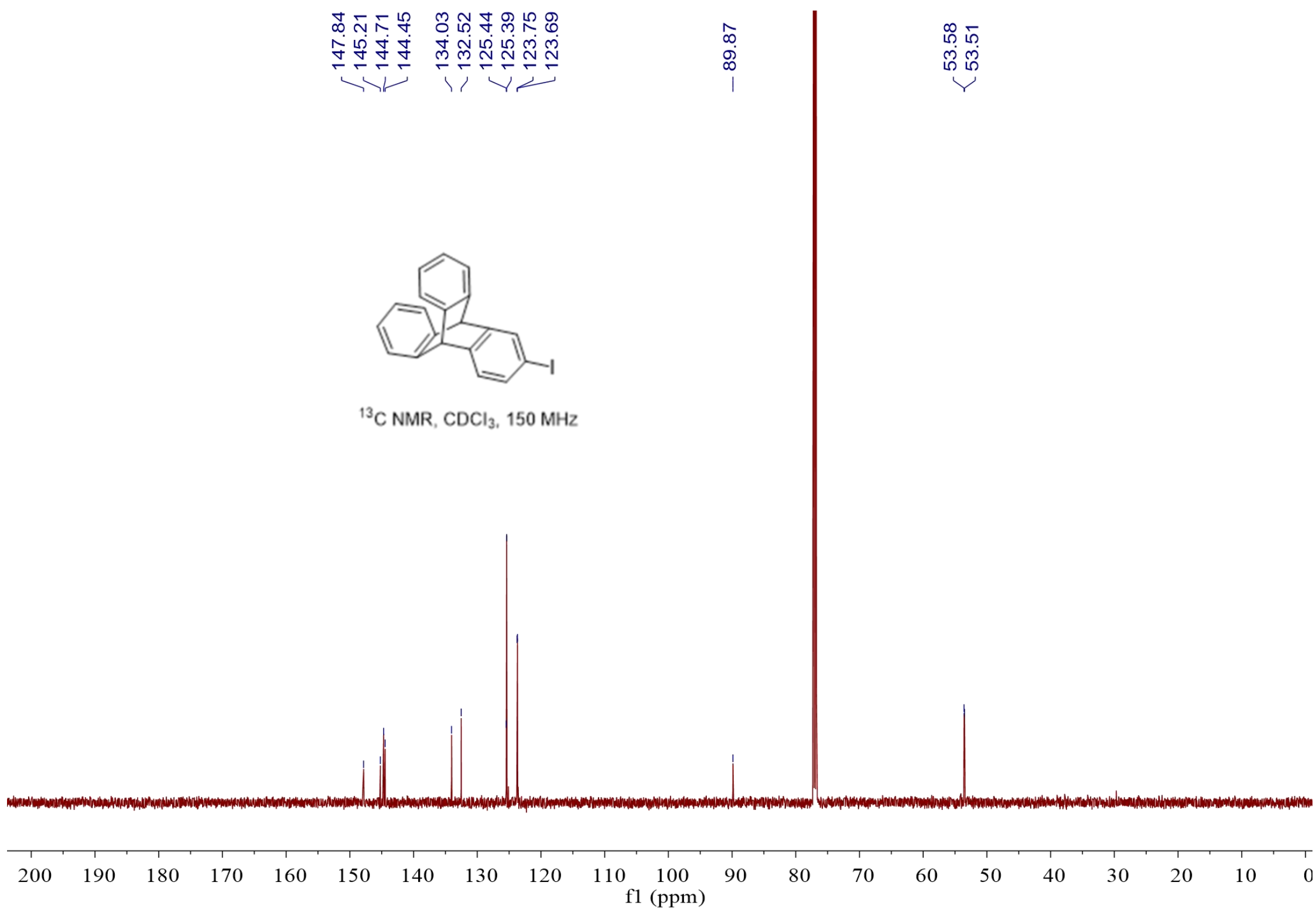
147.84
145.21
144.71
144.45
134.03
132.52
125.44
125.39
123.75
123.69
89.87
53.58
53.51
I
13C NMR, CDCl3, 150 MHz
200 190 180 170 160 150 140 130 120 110 100 90 80 70 60 50 40 30 20 10 0
f1 (ppm)

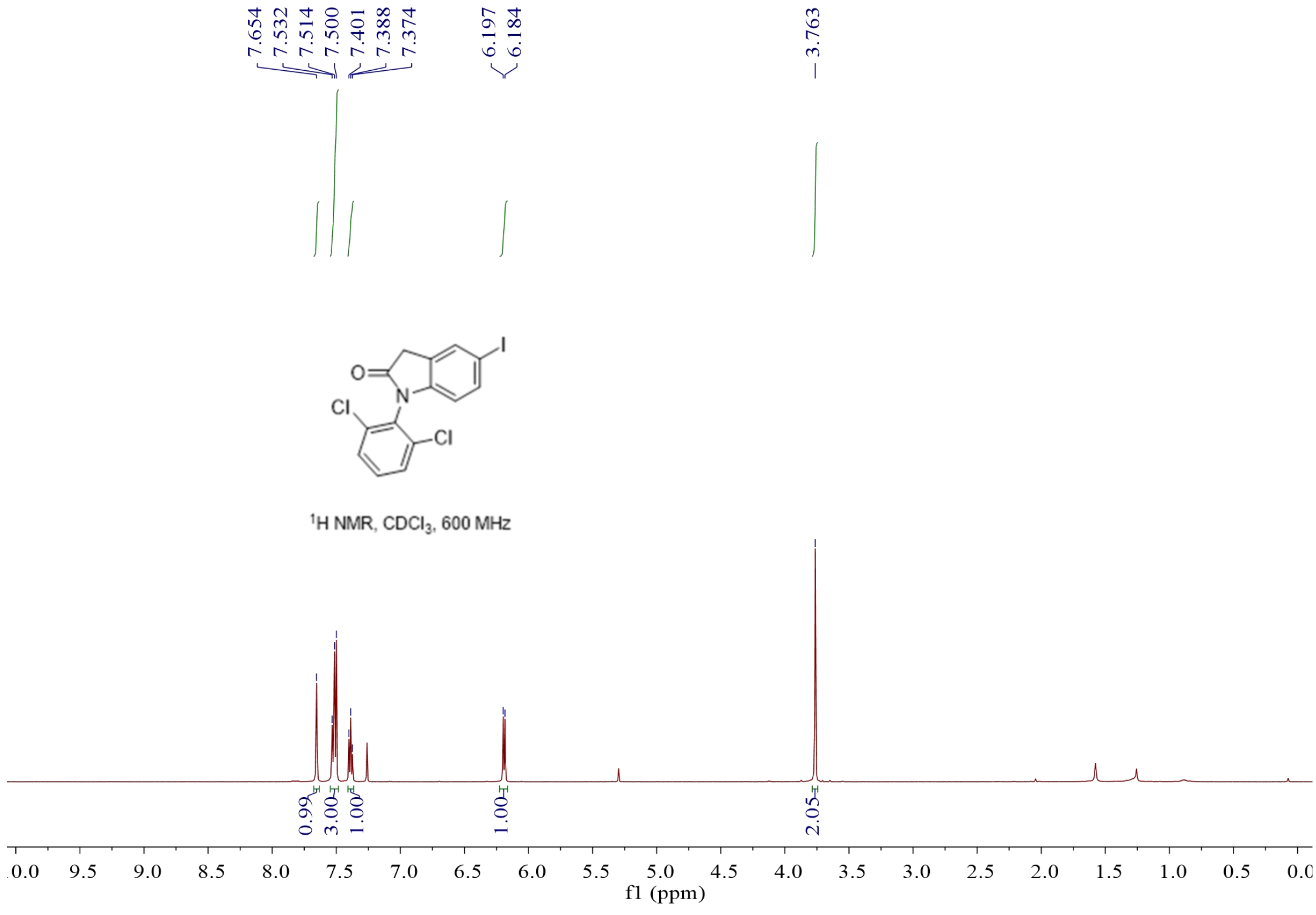
7.654
7.532
7.514
7.500
7.401
7.388
7.374
6.197
6.184
3.763
O
N
I
Cl
Cl
1H NMR, CDCl3, 600 MHz
0.99
3.00
1.00
1.00
2.05
10.0 9.5 9.0 8.5 8.0 7.5 7.0 6.5 6.0 5.5 5.0 4.5 4.0 3.5 3.0 2.5 2.0 1.5 1.0 0.5 0.0
f1 (ppm)

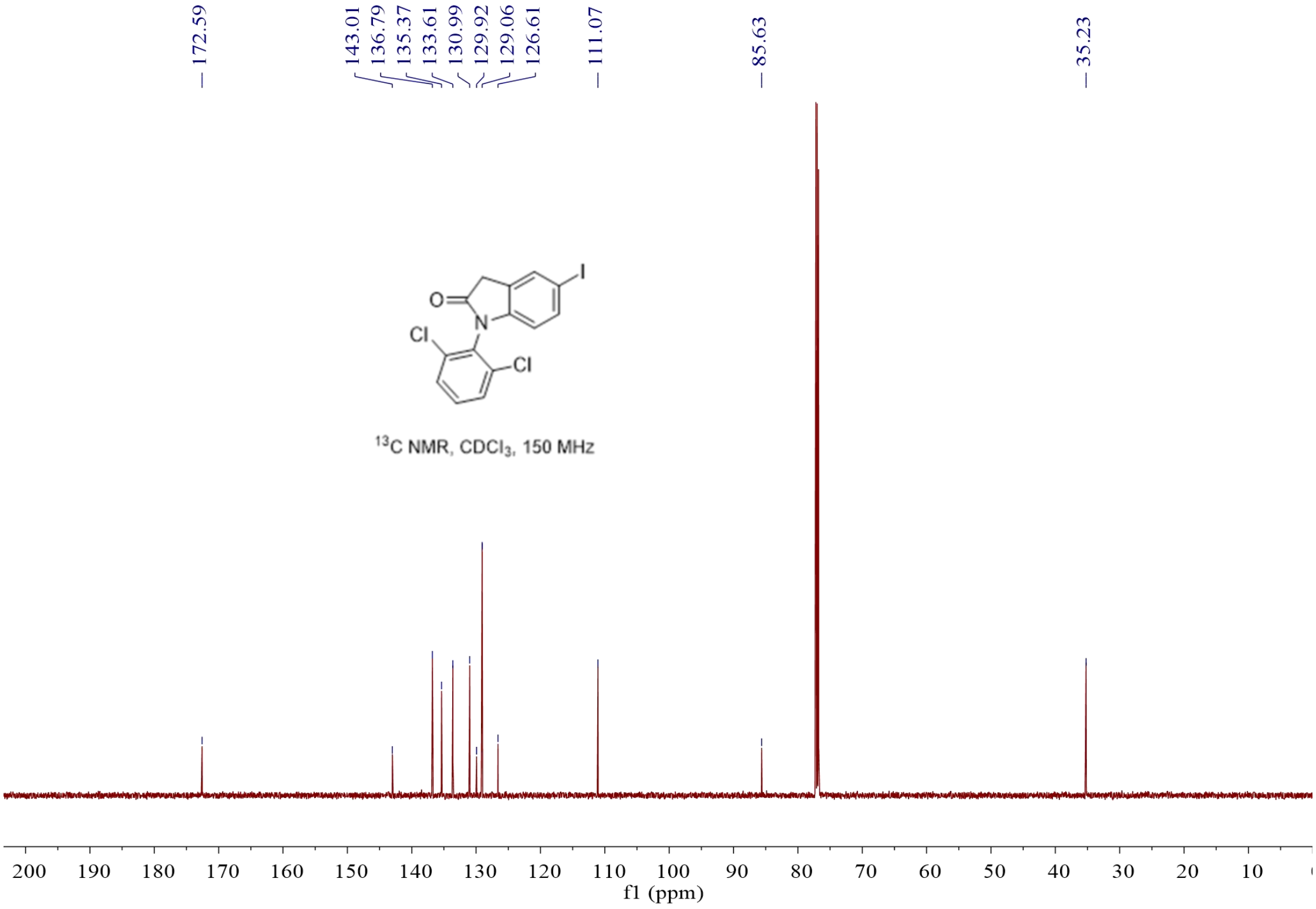
172.59
143.01
136.79
135.37
133.61
130.99
129.92
129.06
126.61
111.07
85.63
35.23
O
N
I
Cl
Cl
13C NMR, CDCl3, 150 MHz
200 190 180 170 160 150 140 130 120 110 100 90 80 70 60 50 40 30 20 10
f1 (ppm)

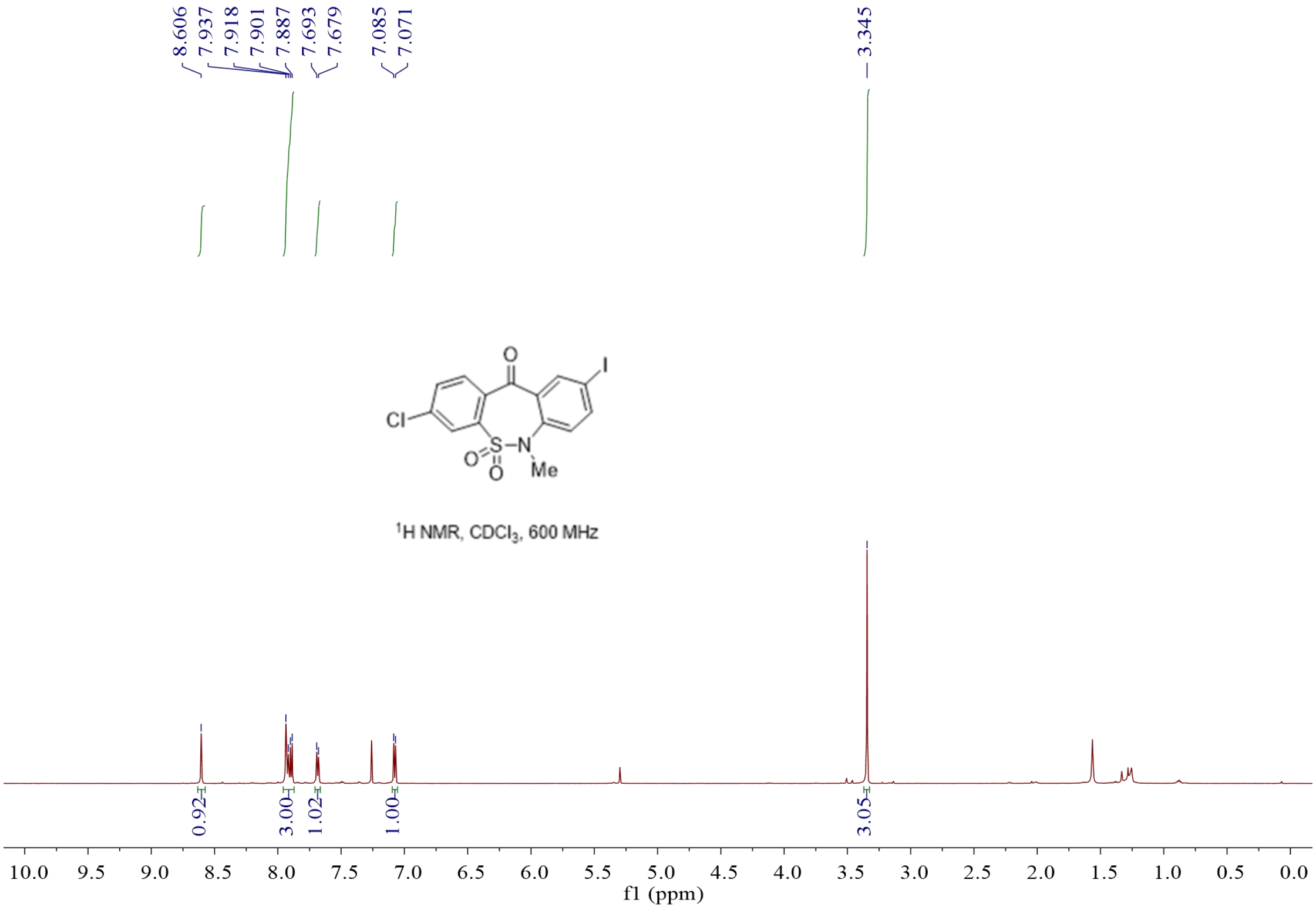
8.606
7.937
7.918
7.901
7.887
7.693
7.679
7.085
7.071
3.345
O
I
Cl
S
N
O
O
Me
1H NMR, CDCl3, 600 MHz
0.92
3.00
1.02
1.00
3.05
10.0 9.5 9.0 8.5 8.0 7.5 7.0 6.5 6.0 5.5 5.0 4.5 4.0 3.5 3.0 2.5 2.0 1.5 1.0 0.5 0.0
f1 (ppm)

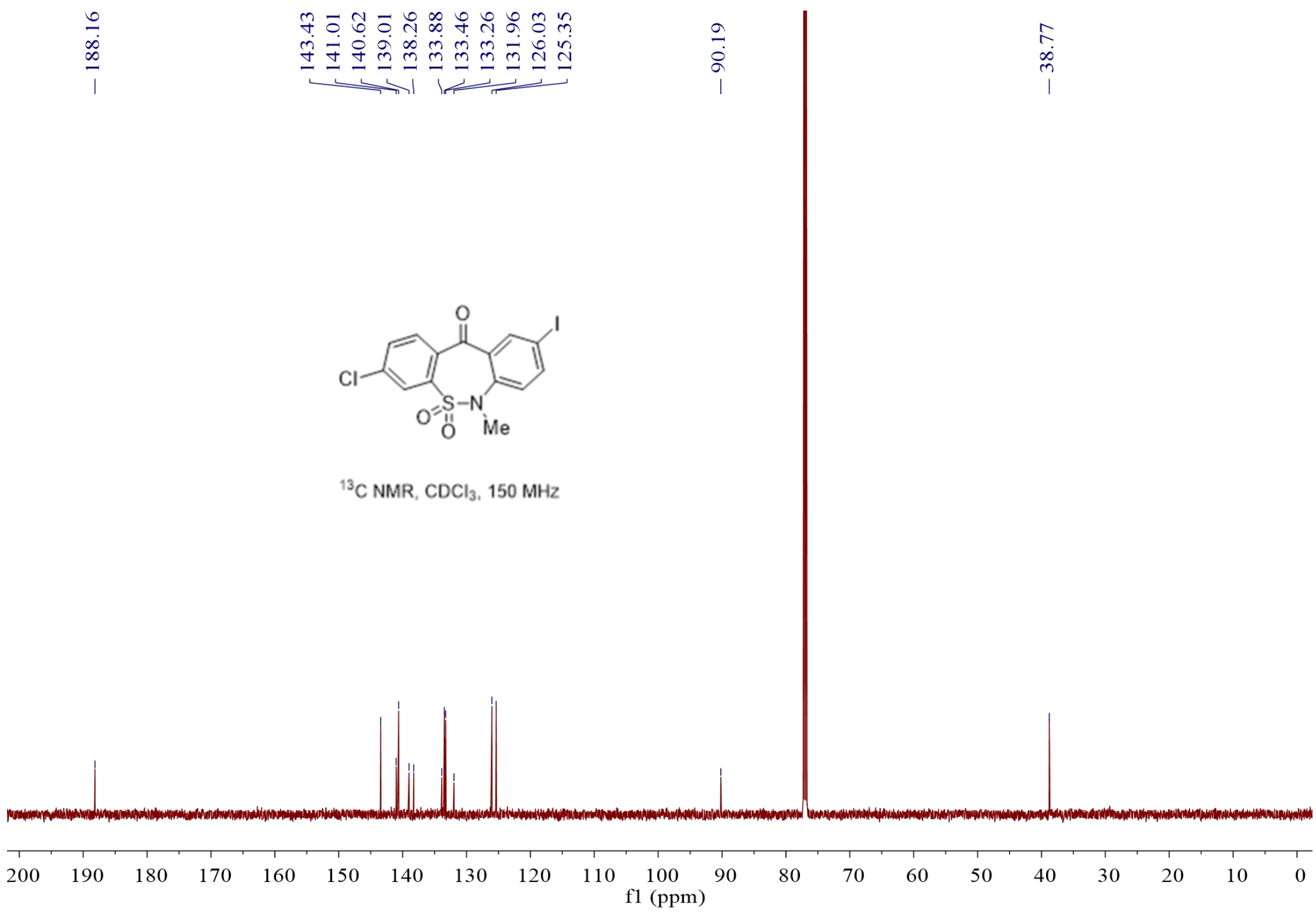
188.16
143.43
141.01
140.62
139.01
138.26
133.88
133.46
133.26
131.96
126.03
125.35
90.19
38.77
Cl
O
I
O
S
N
O
Me
13C NMR, CDCl3, 150 MHz
200 190 180 170 160 150 140 130 120 110 100 90 80 70 60 50 40 30 20 10 0
f1 (ppm)

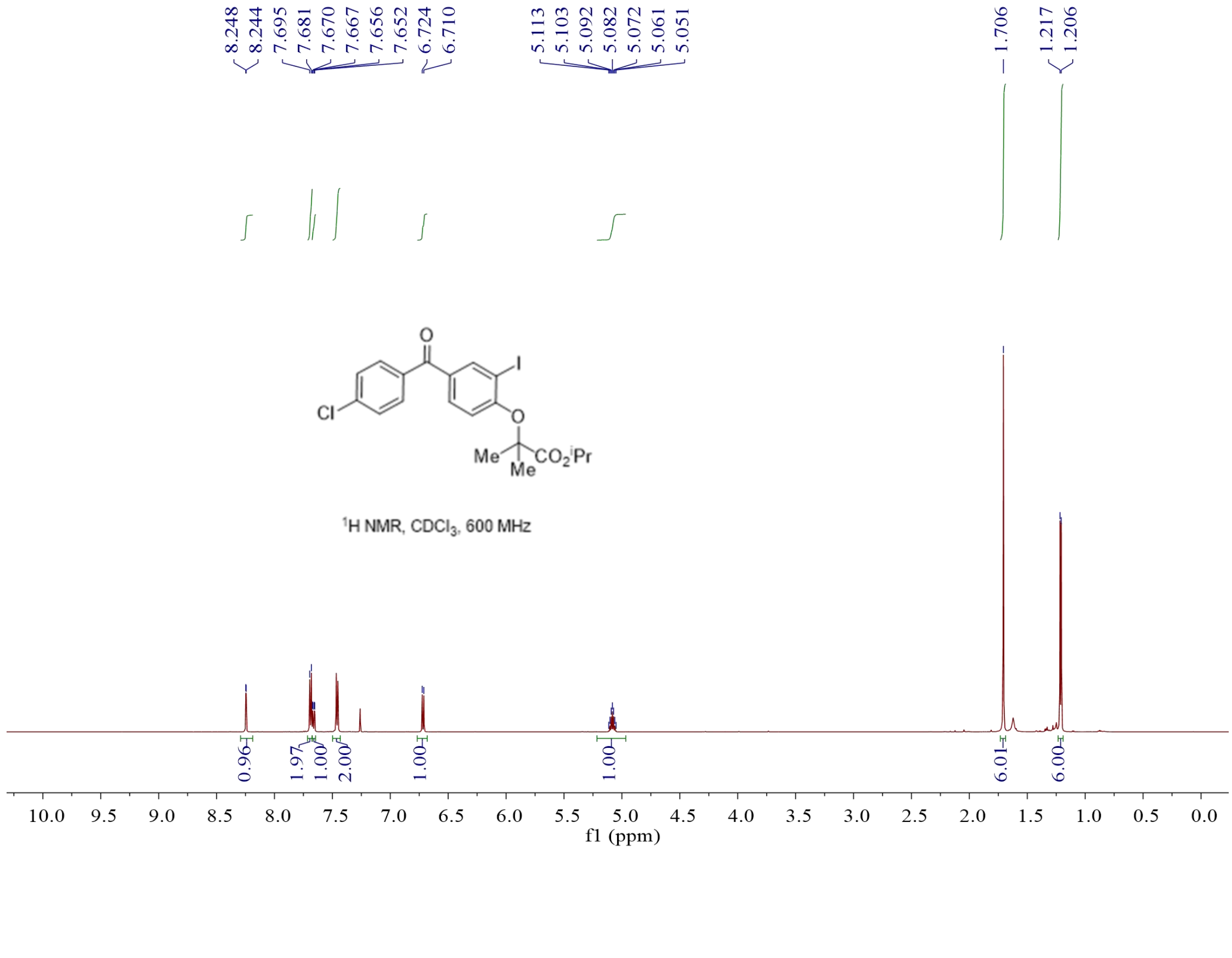
8.248
8.244
7.695
7.681
7.670
7.667
7.656
7.652
6.724
6.710
5.113
5.103
5.092
5.082
5.072
5.061
5.051
1.706
1.217
1.206
Cl
O
I
O
Me
Me
CO2iPr
1H NMR, CDCl3, 600 MHz
0.96
1.97
1.00
2.00
1.00
1.00
6.01
6.00
10.0 9.5 9.0 8.5 8.0 7.5 7.0 6.5 6.0 5.5 5.0 4.5 4.0 3.5 3.0 2.5 2.0 1.5 1.0 0.5 0.0
f1 (ppm)

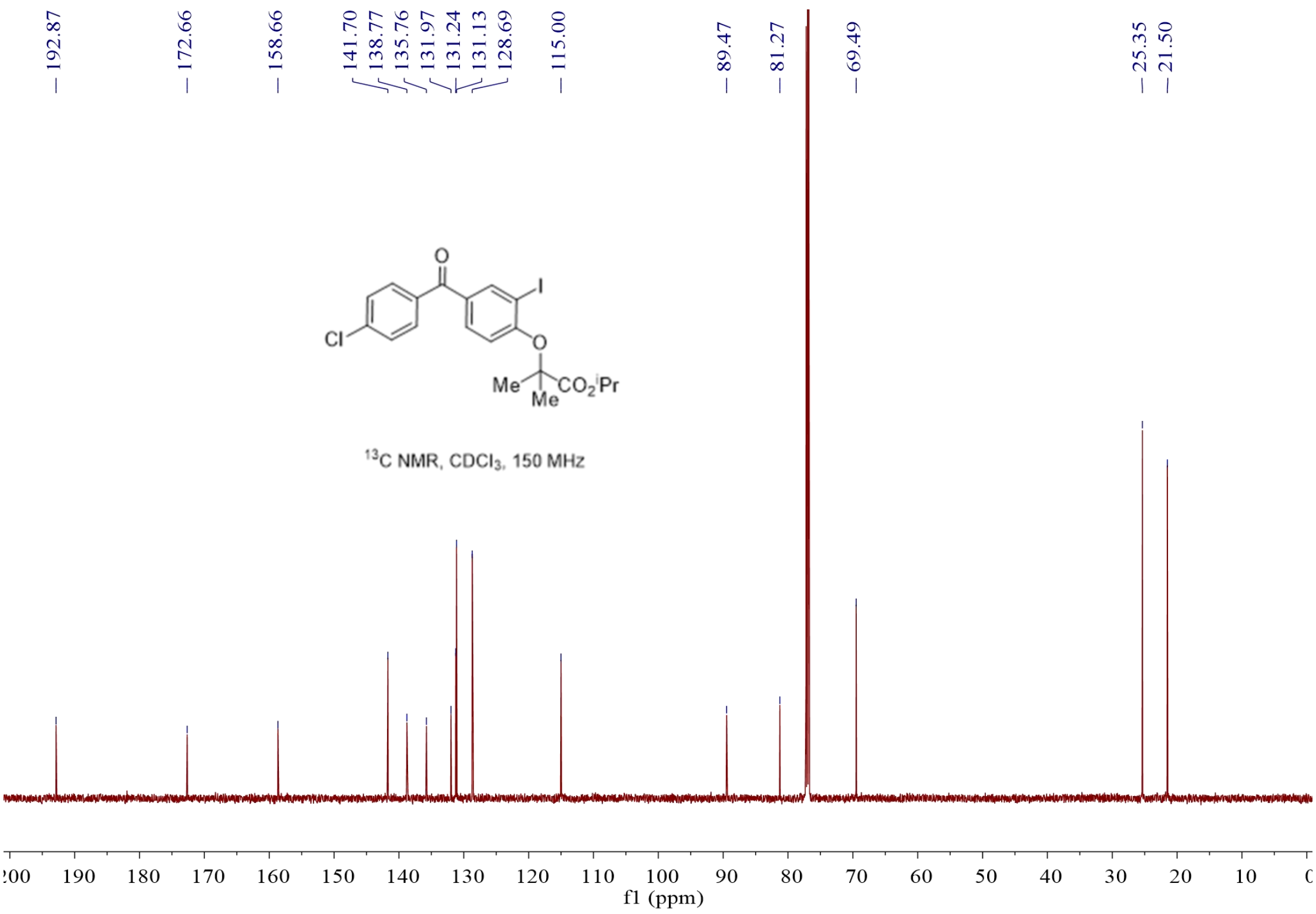

192.87
172.66
158.66
141.70
138.77
135.76
131.97
131.24
131.13
128.69
115.00
89.47
81.27
69.49
25.35
21.50
O
I
Cl
O
Me
Me
CO2iPr
13C NMR, CDCl3, 150 MHz
200 190 180 170 160 150 140 130 120 110 100 90 80 70 60 50 40 30 20 10 0
f1 (ppm)

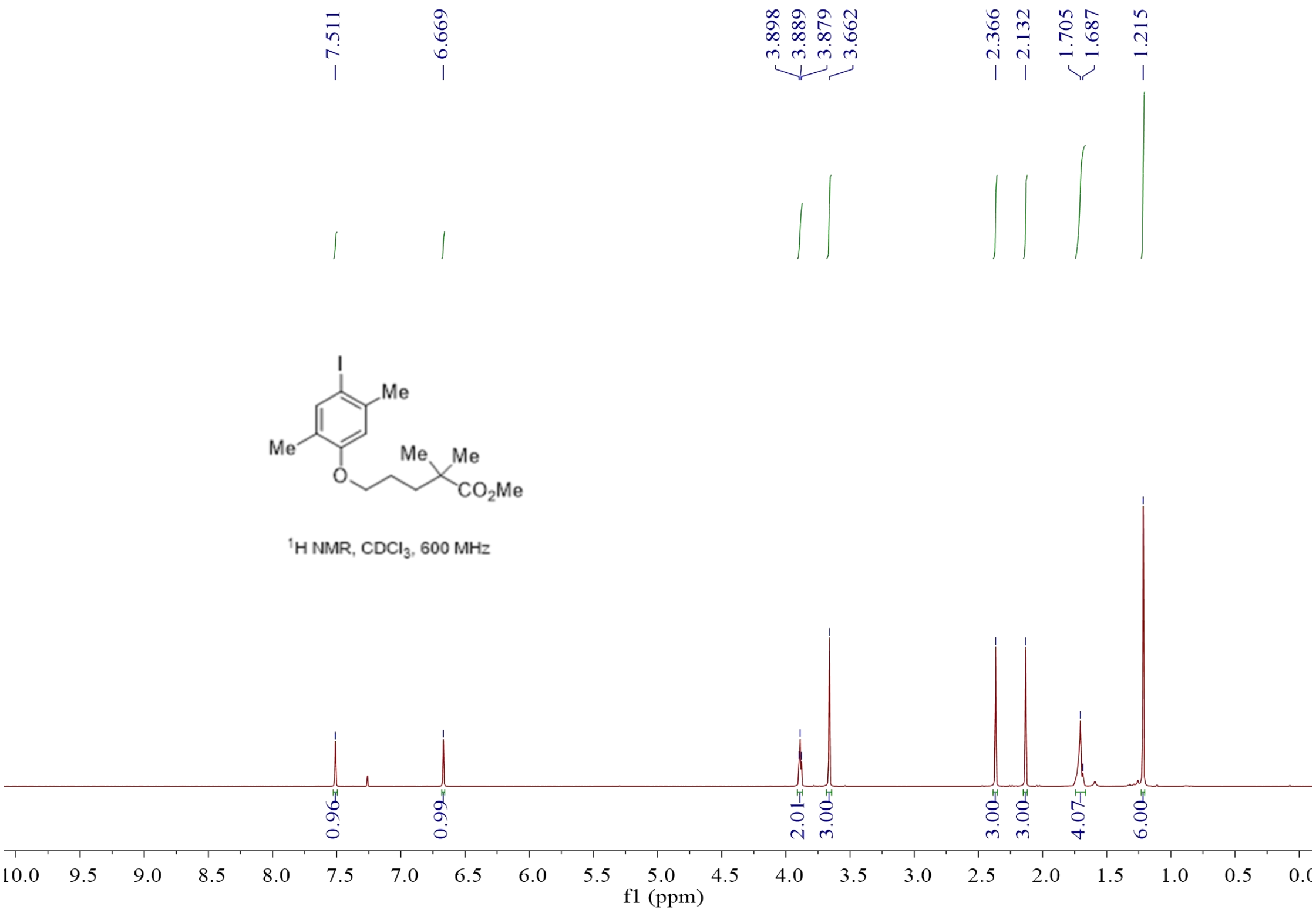

7.511
6.669
3.898
3.889
3.879
3.662
2.366
2.132
1.705
1.687
1.215
I
Me
Me
O
Me
Me
CO2Me
1H NMR, CDCl3, 600 MHz
0.96
0.99
2.01
3.00
3.00
3.00
4.07
6.00
10.0 9.5 9.0 8.5 8.0 7.5 7.0 6.5 6.0 5.5 5.0 4.5 4.0 3.5 3.0 2.5 2.0 1.5 1.0 0.5 0.0
f1 (ppm)

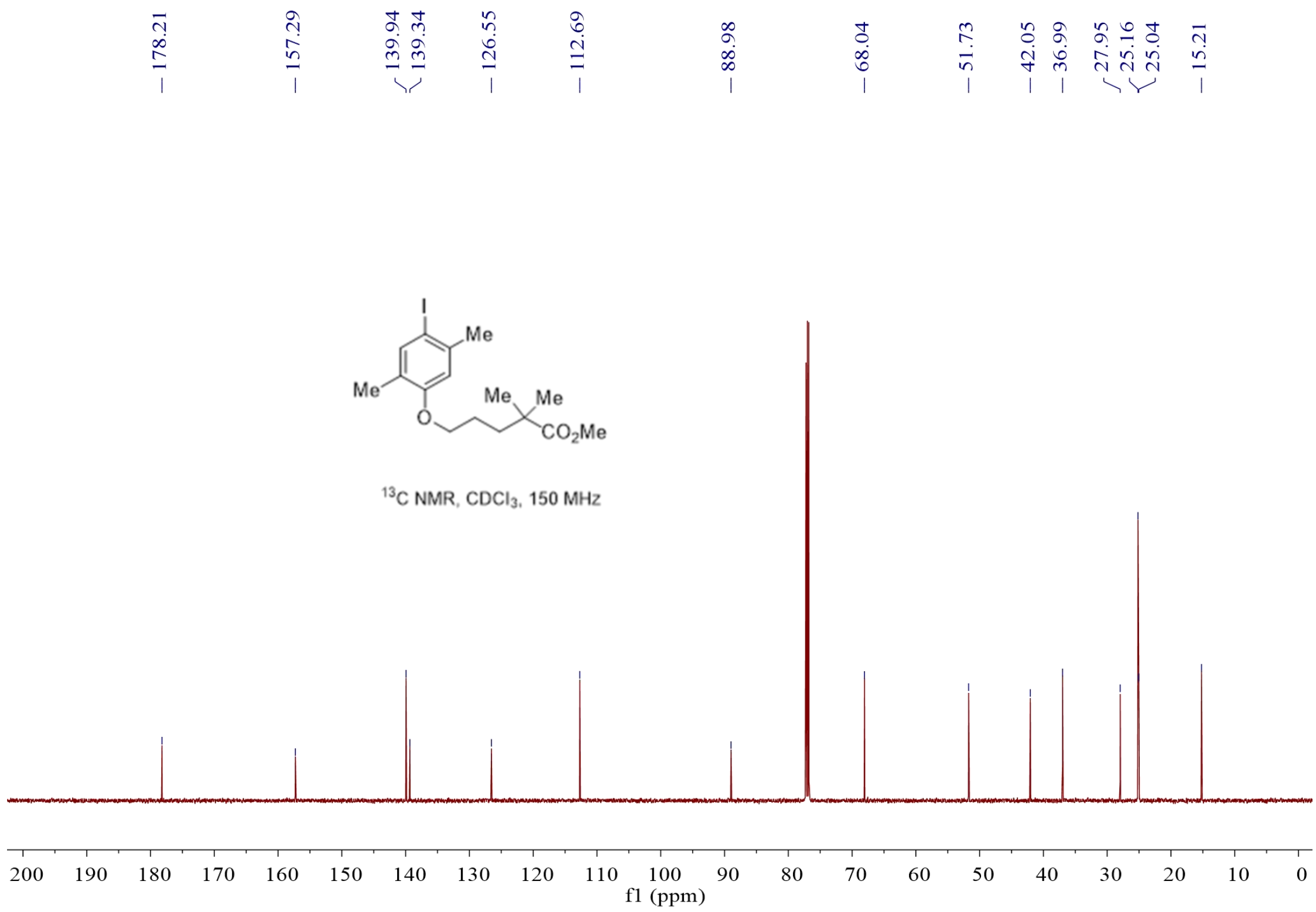
178.21
157.29
139.94
139.34
126.55
112.69
88.98
68.04
51.73
42.05
36.99
27.95
25.16
25.04
15.21
I
Me
Me
Me
Me
O
CO2Me
13C NMR, CDCl3, 150 MHz
200 190 180 170 160 150 140 130 120 110 100 90 80 70 60 50 40 30 20 10 0
f1 (ppm)

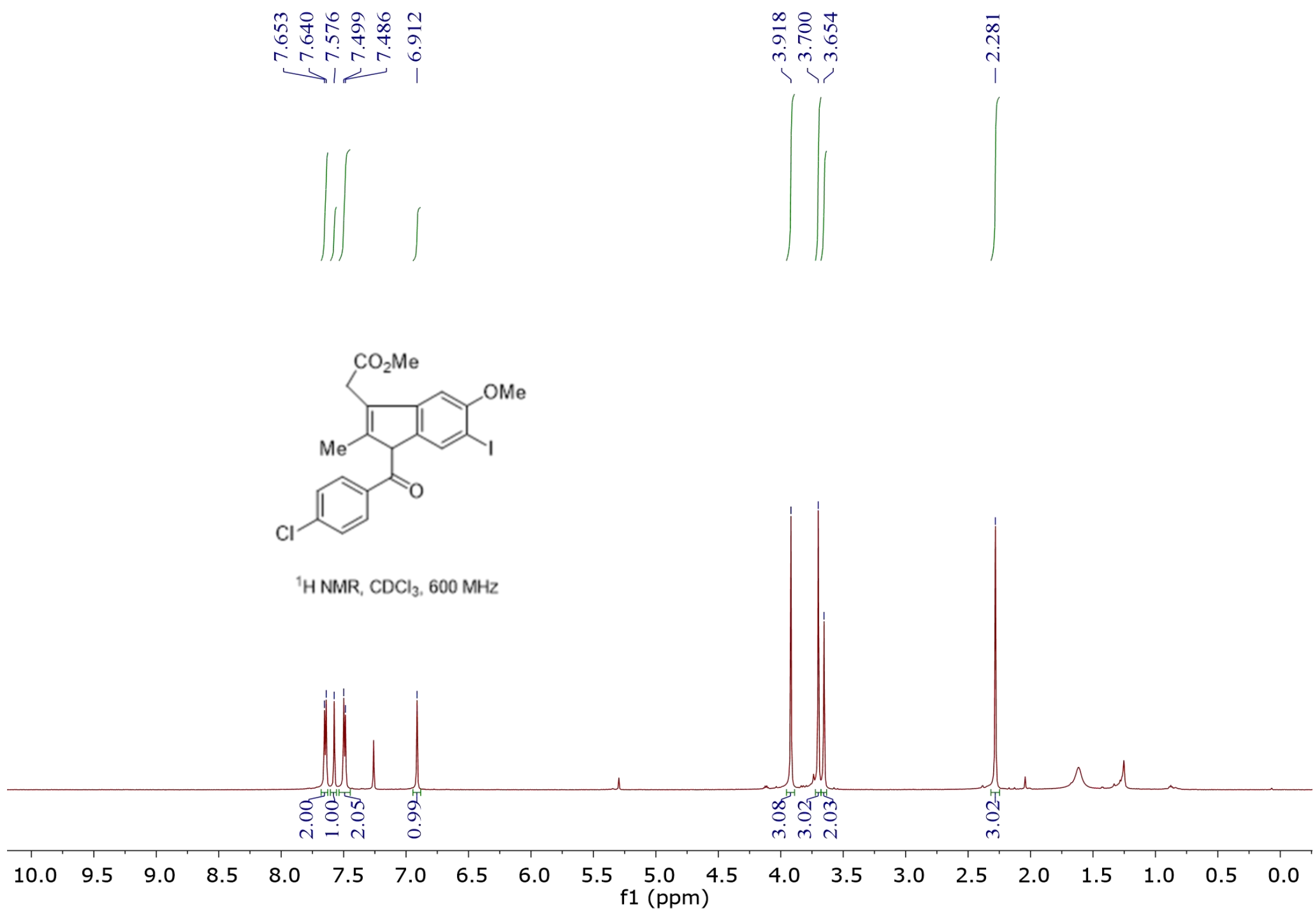
7.653
7.640
7.576
7.499
7.486
6.912
3.918
3.700
3.654
2.281
CO2Me
OMe
Me
I
O
Cl
1H NMR, CDCl3, 600 MHz
2.00
1.00
2.05
0.99
3.08
3.02
2.03
3.02
10.0 9.5 9.0 8.5 8.0 7.5 7.0 6.5 6.0 5.5 5.0 4.5 4.0 3.5 3.0 2.5 2.0 1.5 1.0 0.5 0.0
f1 (ppm)

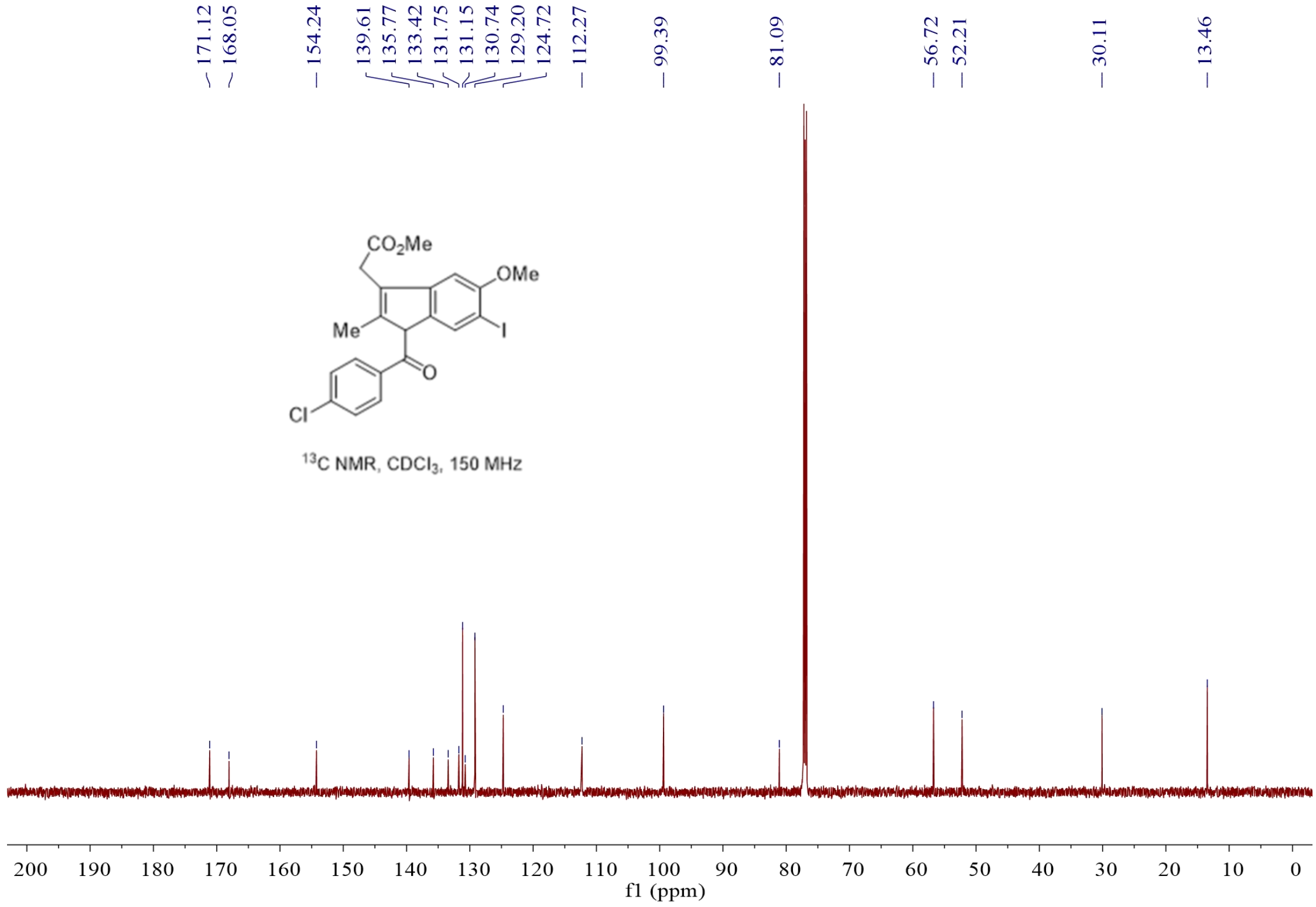

171.12
168.05
154.24
139.61
135.77
133.42
131.75
131.15
130.74
129.20
124.72
112.27
99.39
81.09
56.72
52.21
30.11
13.46
CO_2Me
OMe
Me
I
O
Cl
^{13}C NMR, $CDCl_3$, 150 MHz
200 190 180 170 160 150 140 130 120 110 100 90 80 70 60 50 40 30 20 10 0
f1 (ppm)

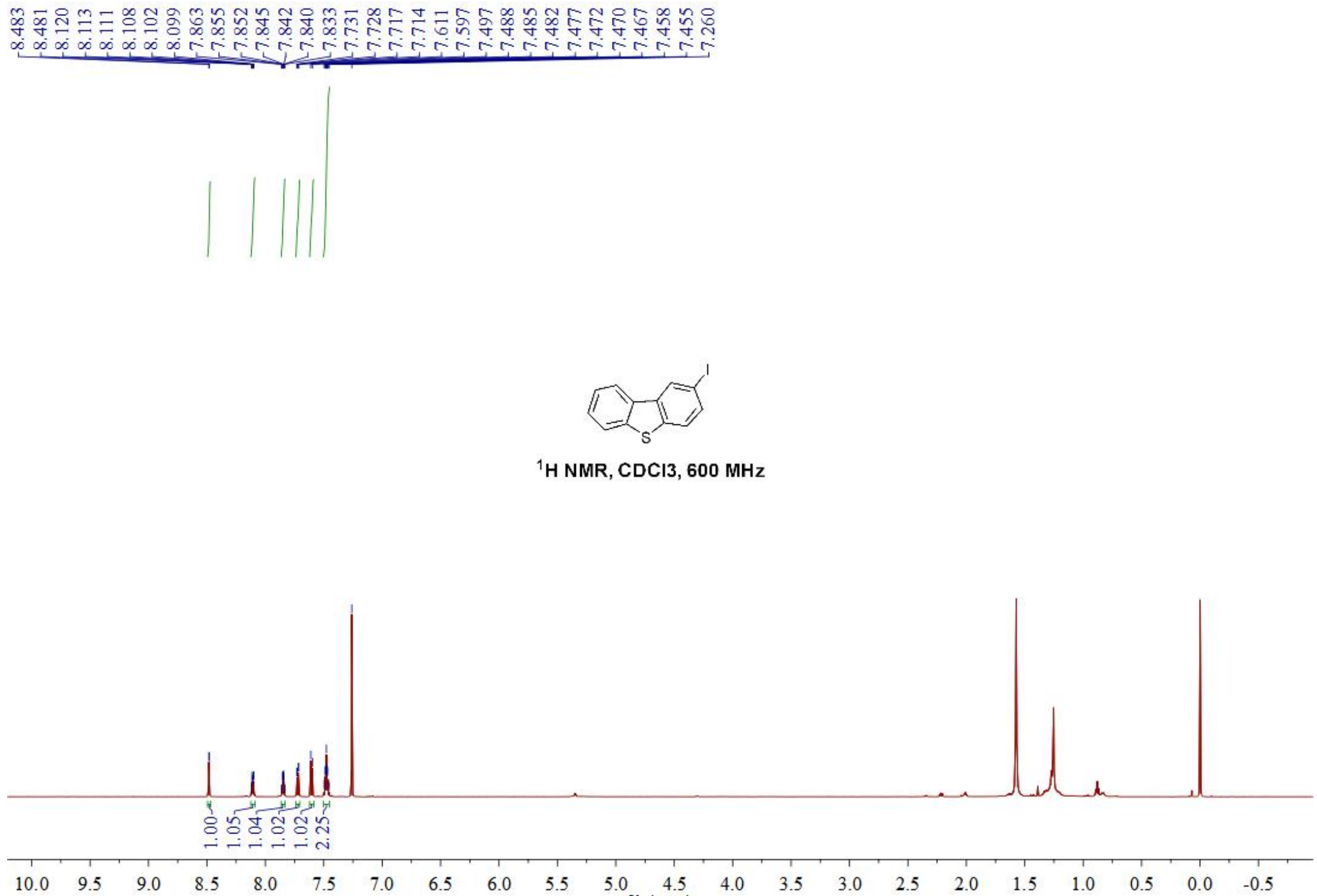

8.483
8.481
8.120
8.113
8.111
8.108
8.102
8.099
7.863
7.855
7.852
7.845
7.842
7.840
7.833
7.731
7.728
7.717
7.714
7.611
7.597
7.497
7.488
7.485
7.482
7.477
7.472
7.470
7.467
7.458
7.455
7.260
I
S
^1H NMR, CDCl3, 600 MHz
1.00
1.05
1.04
1.02
1.02
2.25
10.0 9.5 9.0 8.5 8.0 7.5 7.0 6.5 6.0 5.5 5.0 4.5 4.0 3.5 3.0 2.5 2.0 1.5 1.0 0.5 0.0 -0.5
f1 (ppm)

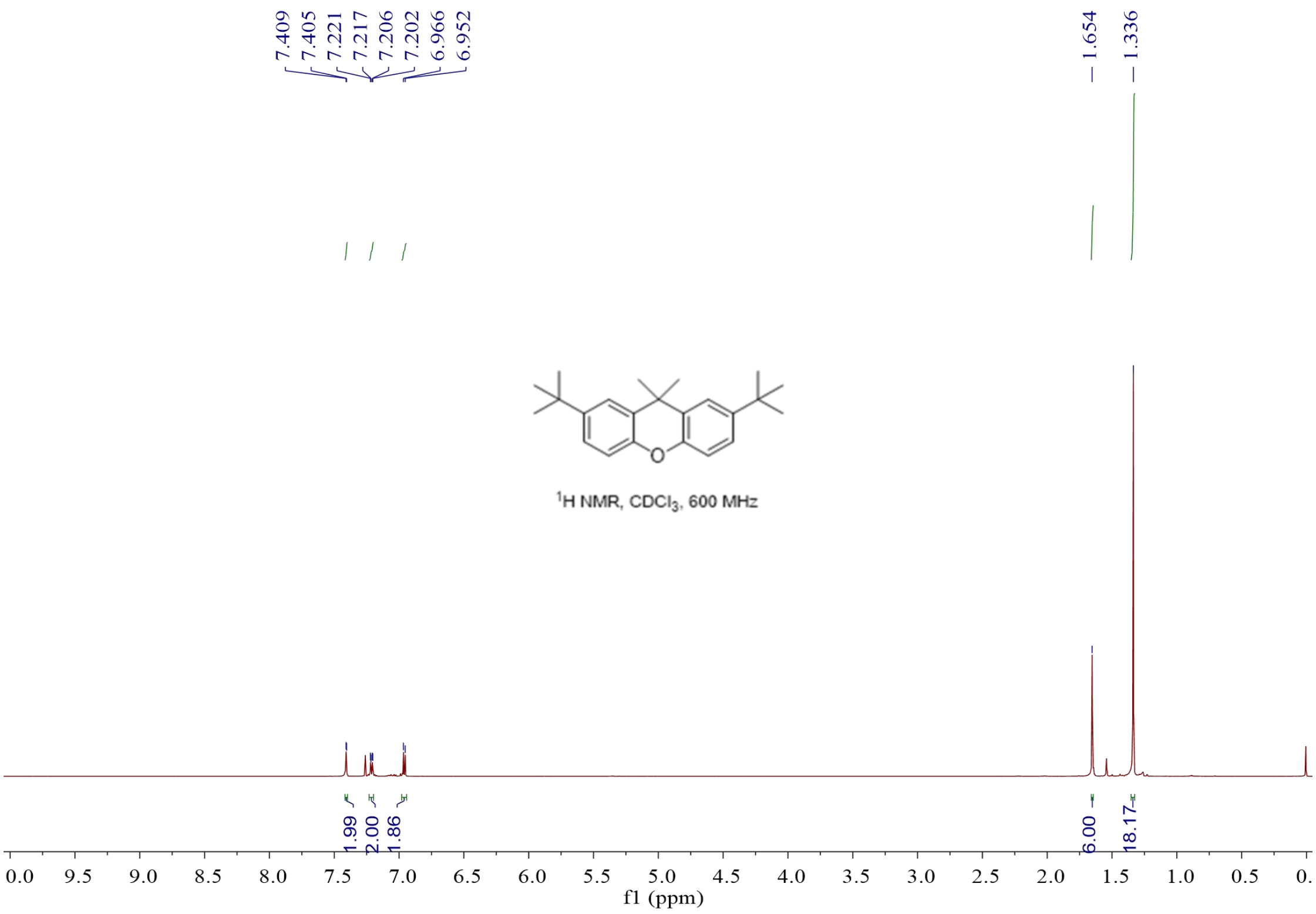
7.409
7.405
7.221
7.217
7.206
7.202
6.966
6.952
1.654
1.336
O
1H NMR, CDCl3, 600 MHz
1.99
2.00
1.86
6.00
18.17
0.0
9.5
9.0
8.5
8.0
7.5
7.0
6.5
6.0
5.5
5.0
4.5
4.0
3.5
3.0
2.5
2.0
1.5
1.0
0.5
0.
f1 (ppm)

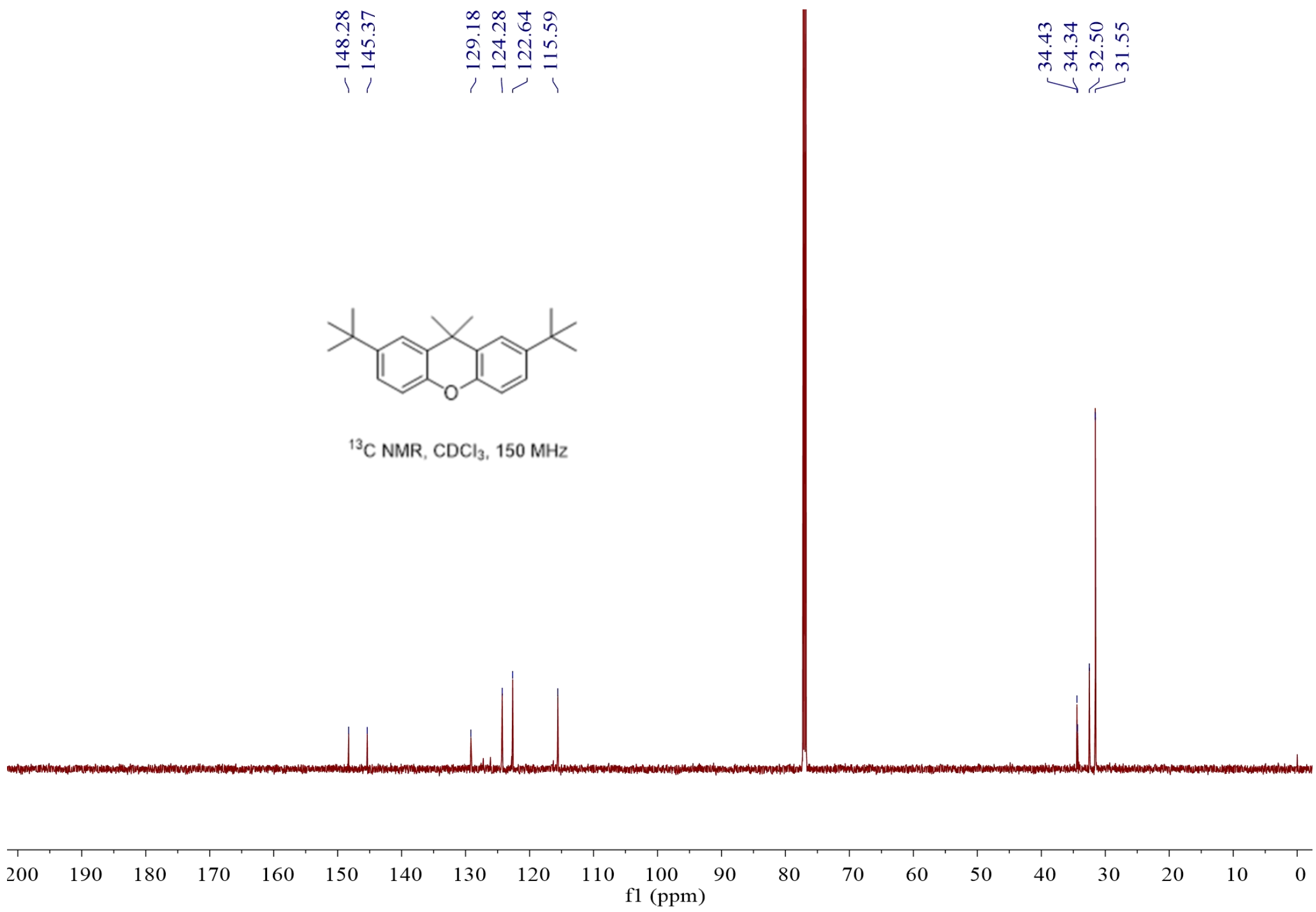
148.28
145.37
129.18
124.28
122.64
115.59
34.43
34.34
32.50
31.55
^{13}C NMR, $CDCl_3$, 150 MHz
200 190 180 170 160 150 140 130 120 110 100 90 80 70 60 50 40 30 20 10 0
f1 (ppm)

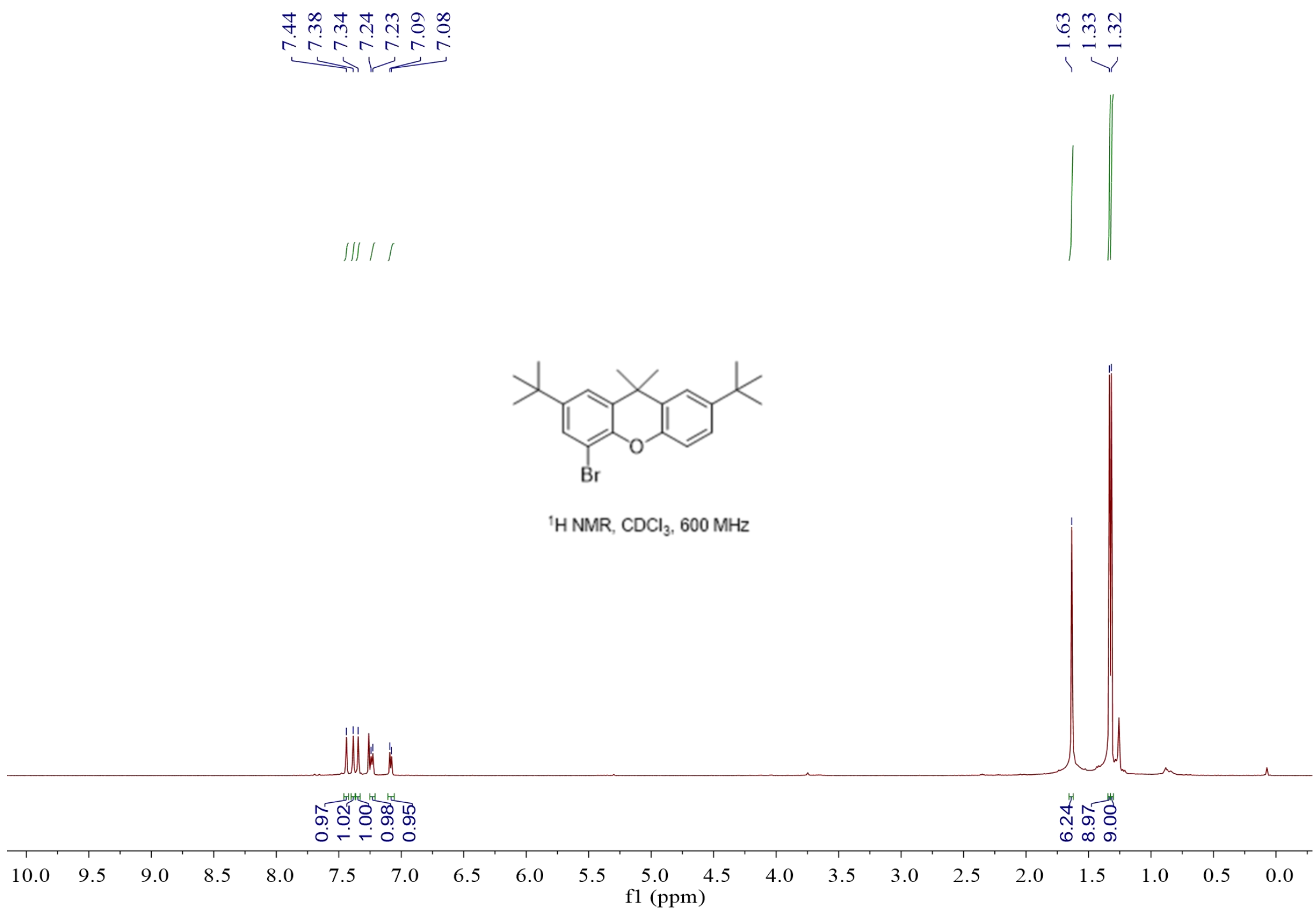
7.44
7.38
7.34
7.24
7.23
7.09
7.08
1.63
1.33
1.32
Br
O
^{1}H NMR, $CDCl_3$, 600 MHz
0.97
1.02
1.00
0.98
0.95
6.24
8.97
9.00
10.0 9.5 9.0 8.5 8.0 7.5 7.0 6.5 6.0 5.5 5.0 4.5 4.0 3.5 3.0 2.5 2.0 1.5 1.0 0.5 0.0
f1 (ppm)

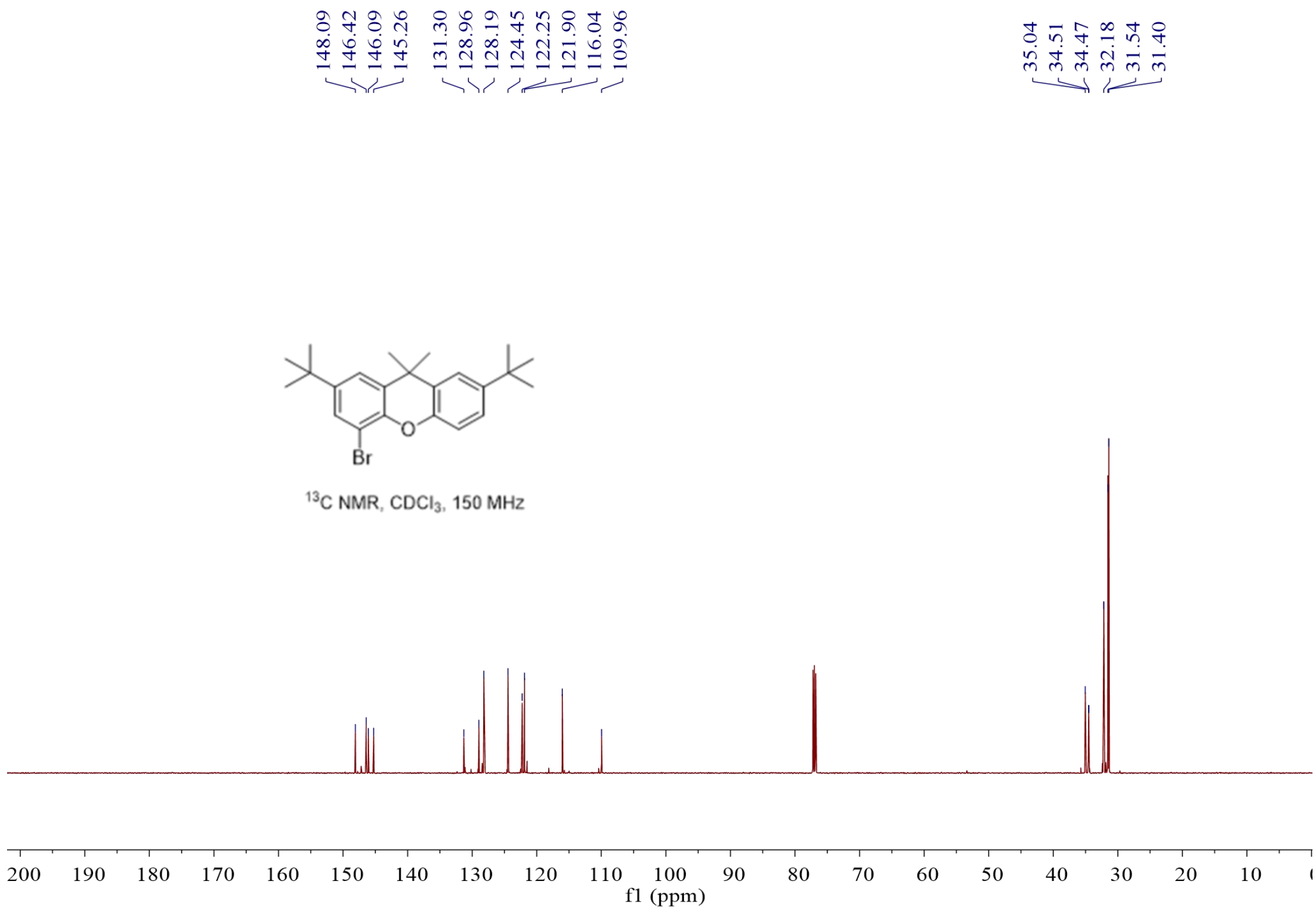
148.09
146.42
146.09
145.26
131.30
128.96
128.19
124.45
122.25
121.90
116.04
109.96
35.04
34.51
34.47
32.18
31.54
31.40
Br
O
13C NMR, CDCl3, 150 MHz
200 190 180 170 160 150 140 130 120 110 100 90 80 70 60 50 40 30 20 10
f1 (ppm)

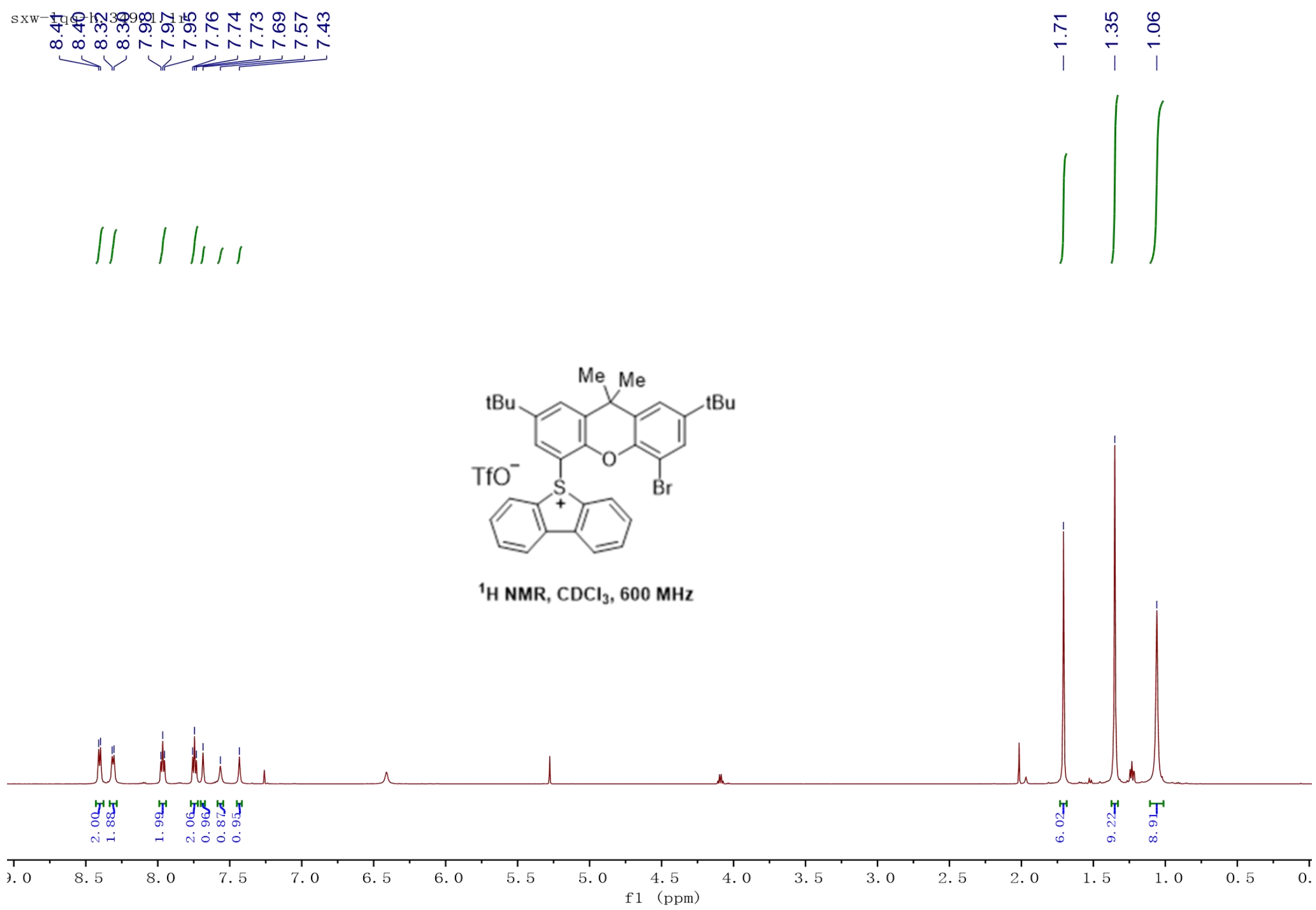
8.41
8.40
8.32
8.30
7.98
7.97
7.95
7.76
7.74
7.73
7.69
7.57
7.43
1.71
1.35
1.06
Me
Me
tBu
tBu
TfO-
S
+
O
Br
1H NMR, CDCl3, 600 MHz
2.00
1.88
1.99
2.06
0.96
0.87
0.95
6.02
9.22
8.91
8.5 8.0 7.5 7.0 6.5 6.0 5.5 5.0 4.5 4.0 3.5 3.0 2.5 2.0 1.5 1.0 0.5
f1 (ppm)

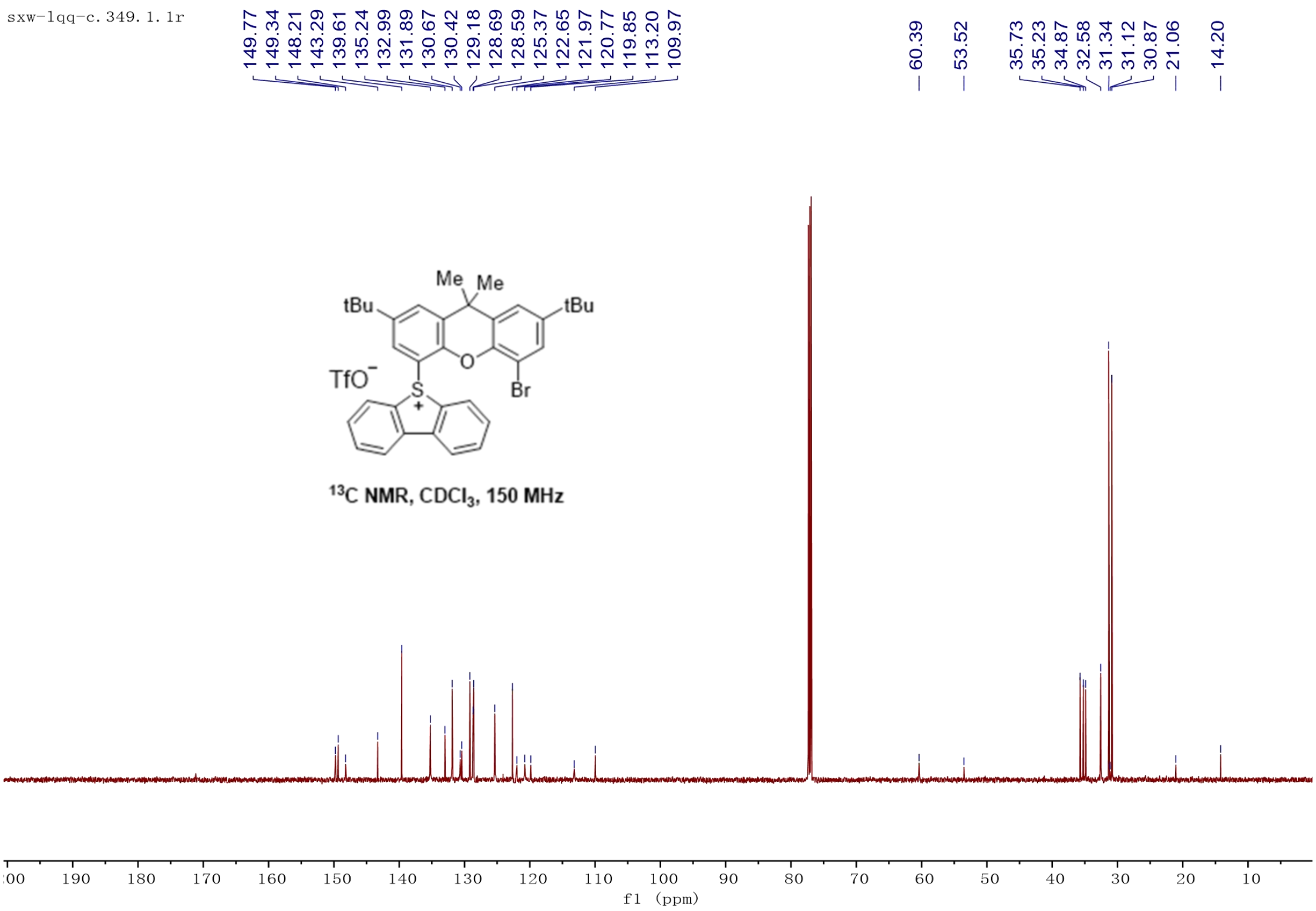
sxw-1qq-c.349.1.1r
149.77
149.34
148.21
143.29
139.61
135.24
132.99
131.89
130.67
130.42
129.18
128.69
128.59
125.37
122.65
121.97
120.77
119.85
113.20
109.97
60.39
53.52
35.73
35.23
34.87
32.58
31.34
31.12
30.87
21.06
14.20
Me
Me
tBu
tBu
TfO⁻
O
S
+
Br
13C NMR, CDCl3, 150 MHz
f1 (ppm)

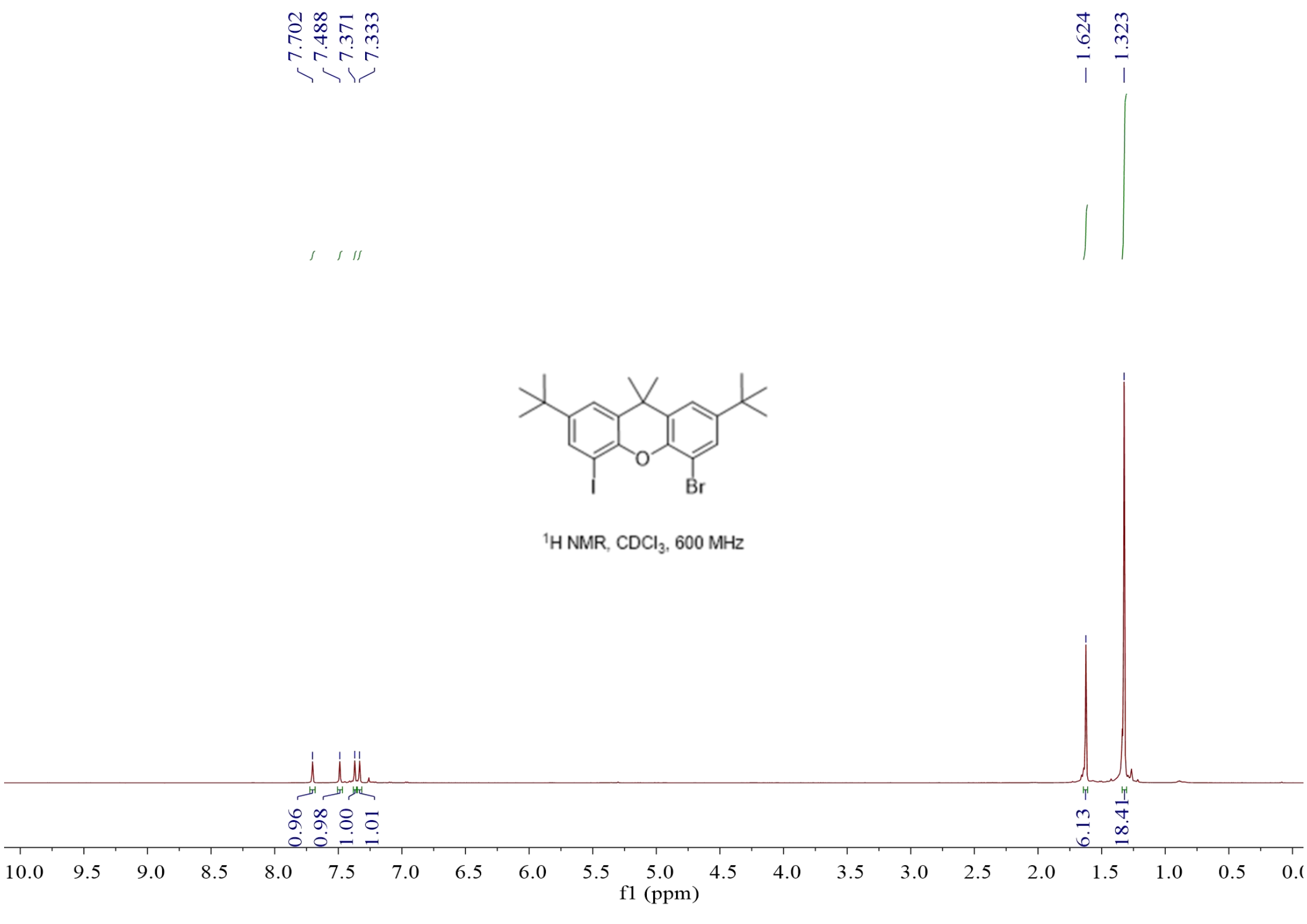
7.702
7.488
7.371
7.333
1.624
1.323
O
I
Br
1H NMR, CDCl3, 600 MHz
0.96
0.98
1.00
1.01
6.13
18.41
f1 (ppm)

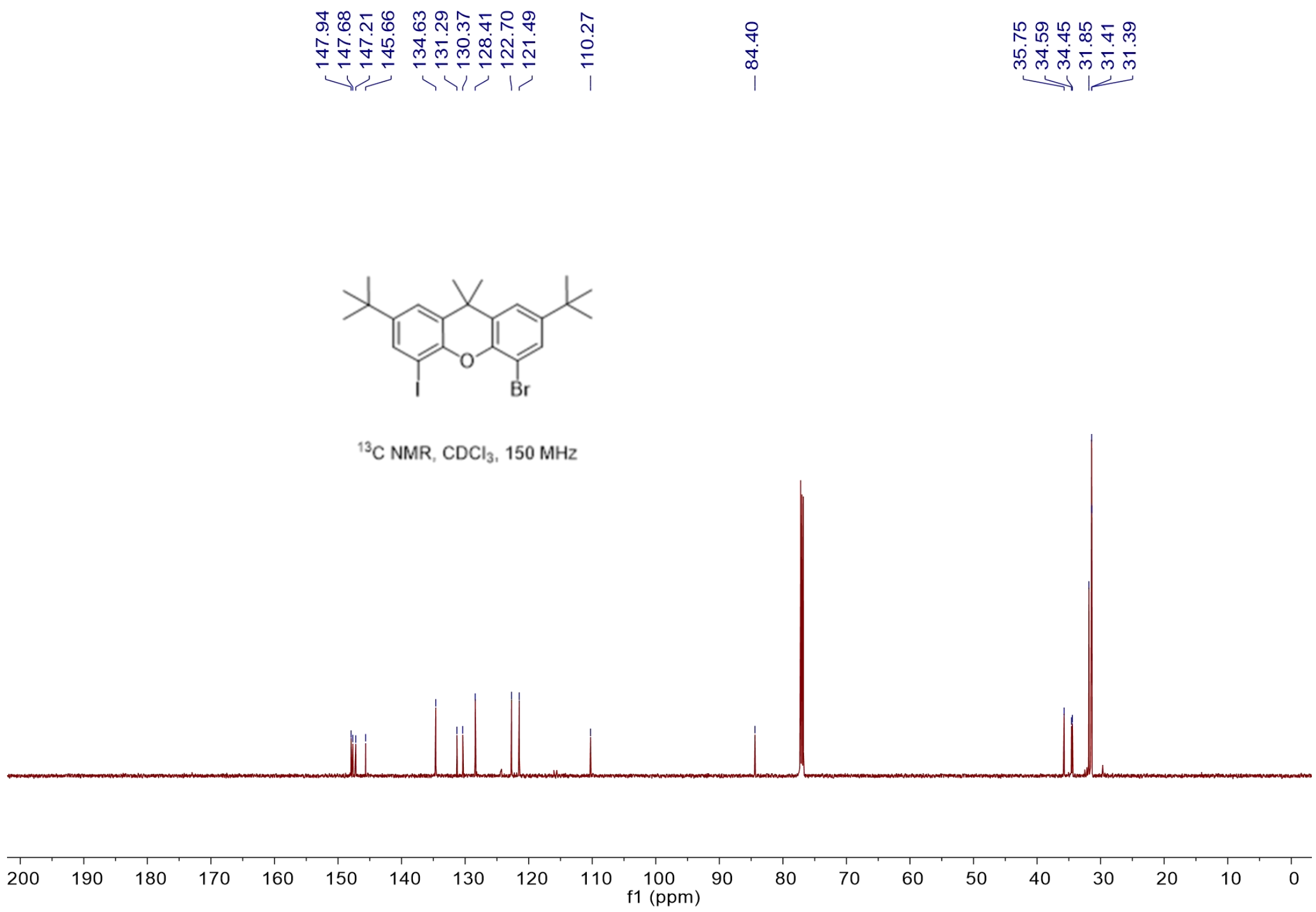
147.94
147.68
147.21
145.66
134.63
131.29
130.37
128.41
122.70
121.49
110.27
84.40
35.75
34.59
34.45
31.85
31.41
31.39
O
I
Br
^{13}C NMR, $CDCl_3$, 150 MHz
200 190 180 170 160 150 140 130 120 110 100 90 80 70 60 50 40 30 20 10 0
f1 (ppm)
